# Gene Expression and Functional Analyses of Odorant Receptors in Small Hive Beetles (*Aethina tumida*)

**DOI:** 10.3390/ijms21134582

**Published:** 2020-06-27

**Authors:** Yuanzhen Liu, Alexis Beaurepaire, Curtis W. Rogers, Dawn Lopez, Jay D. Evans, Lars Straub, Peter Neumann, Steven C. Cook, Qiang Huang

**Affiliations:** 1 Institute of Bee Health, Vetsuisse Faculty, University of Bern, Schwarzenburgstrasse 161, Liebefeld, 3097 Bern, Switzerland; alexis.beaurepaire@vetsuisse.unibe.ch (A.B.); lars.straub@vetsuisse.unibe.ch (L.S.); peter.neumann@vetsuisse.unibe.ch (P.N.); 2 Agroscope, Swiss Bee Research Center, Schwarzenburgstrasse 161, Liebefeld, 3097 Bern, Switzerland; 3 USDA-ARS Beltsville Bee Research Laboratory, Building 306, Beltsville, MD 20705, USA; curtis.rogers@usda.gov (C.W.R.); dawn.lopez@usda.gov (D.L.); jay.evans@usda.gov (J.D.E.); steven.cook@usda.gov (S.C.C.); 4 Honeybee Research Institute, Jiangxi Agricultural University, Zhimin Avenue1101, Nanchang 330045, China

**Keywords:** invasive species, odorant receptor, olfaction, phylogeny

## Abstract

Olfaction is key to many insects. Odorant receptors (ORs) stand among the key chemosensory receptors mediating the detection of pheromones and kairomones. Small hive beetles (SHBs), *Aethina tumida*, are parasites of social bee colonies and olfactory cues are especially important for host finding. However, how interactions with their hosts may have shaped the evolution of ORs in the SHB remains poorly understood. Here, for the first time, we analyzed the evolution of SHB ORs through phylogenetic and positive selection analyses. We then tested the expression of selected OR genes in antennae, heads, and abdomens in four groups of adult SHBs: colony odor-experienced/-naive males and females. The results show that SHBs experienced both OR gene losses and duplications, thereby providing a first understanding of the evolution of SHB ORs. Additionally, three candidate ORs potentially involved in host finding and/or chemical communication were identified. Significantly different downregulations of ORs between the abdomens of male and female SHBs exposed to colony odors may reflect that these expression patterns might also reflect other internal events, e.g., oviposition. Altogether, these results provide novel insights into the evolution of SHB ORs and provide a valuable resource for analyzing the function of key genes, e.g., for developing biological control. These results will also help in understanding the chemosensory system in SHBs and other beetles.

## 1. Introduction

Airborne chemical stimuli can provide animals with diverse information such as food sources, mates, predators, and oviposition sites [1,2,3]. In insects, these chemical signals, including sex or aggregation pheromones as well as interspecific kairomones, are usually received by olfactory sensory neurons within specialized antennal and olfactory sensilla [3]. Additionally, mouthparts and other body parts (e.g., maxillary and labial palps) are also reported to be involved in insect olfaction [2,4]. The perception of olfactory signals is typically mediated by three different multigene families of chemosensory receptors, i.e., ionotropic receptors (IRs), gustatory receptors (GRs), and odorant receptors (ORs). These three families focus on different targets: while IRs detect humidity, acids, and alcohols [5,6], GRs are primarily responsible for detecting tastants [7], and ORs mediate the detection of pheromones and volatile food odorants [8,9]. In addition, the chemosensory proteins (CSPs) and insect-type odorant-binding receptors (OBPs), two soluble binding protein families, are known to mediate the transport of hydrophobic ligands through the aqueous environment of the sensillar lymph and to enhance the sensitivity of the insect ORs [10,11]. Some evidence showed that OBPs and CSPs may participate in insect olfaction, for example, differential expression of OBPs to pheromone stimuli [12], and extremely versatile CSPs performing olfactory tasks but also others [13]; however, their precise role in modulating olfactory responses remains unclear [10,14]. These made them less attractive as targets for identifying key genes. In contrast, ORs have received the most attention as they are considered to be the crucial chemosensory gene family [15] and are involved in a broad range of traits, such as the detection of most volatile stimuli associated with food and mates in insects [3].

Our knowledge of the neuronal circuitry and molecular basis of insect ORs has improved greatly over the past decade [3]. As an insect-exclusive expansion of seven-transmembrane domain proteins (7tm_6), ORs bind diverse environmental odors and typically form heteromeric complexes of ligand-gated ion channels when bound to conserved co-receptors (ORcos) [16,17,18]. These complexes transform the chemical signal into the activation of neurons in the brain, mediating the behavior of insects [18]. To dynamically adjust odor sensitivity to strong and/or maintained odor, the insect olfactory system has to be able to downregulate the response in a use-dependent manner [19]. Use-dependent, i.e., odor experienced/naïve, expression of specific OR genes might reflect the chemical sensitivity of olfaction to environmental odors [20]. Despite the antennae being the major chemosensory structure of insects, chemosensation is not restricted to the antennae. Differential expressions of ORs have been discovered among different body parts, including mouthparts, legs, and even abdomens, which ORs were suggested to be involved in olfaction or female oviposition [4,21] and chemotaxis by sperm cells [22]. Additionally, sex-biased OR expression is detected in some insects such as silkworm, *Bombyx mori* [23], and scarab beetle, *Holotrichia oblita* [21]. This finding suggests that some ORs may detect odors critical to female or male behavior, for instance, host-seeking or female-produced sex pheromones [24]. Furthermore, the recently characterized functional insect chemosensory receptors/proteins can be utilized in biosensors [25,26]. For example, ORs from *Drosophila melanogaster* have been integrated into lipid substrates and used as sensing elements for bioelectronic nose technologies [27] ranging from agro-food sectors, e.g., food safety, to the clinical diagnostics [25].

Studies on the diversity and evolution of OR genes that code for olfactory receptor proteins in different species can provide insights into the evolutionary relationships between organisms and may reveal how organism-environment interactions have evolved [28]. Recent discoveries pointed out that the OR gene family appears to be highly divergent across insect species [29,30]. Notably, the evolution of OR genes may be influenced by interactions between hosts and their parasites [14]. For example, most *Drosophila* species are attracted to decaying fruits, whereas *Drosophila suzukii* females parasitize ripening fruit, particularly, a variety of berries [31]. In contrast to its closely related species *Drosophila biarmipes*, gene duplications and evidence of positive selection are found at the OR loci of *D. suzukii* [32]. It seems that the behavior and the host breadth may shape the evolution of ORs [3,15,18]. However, the links between gene evolution and ecological specialization are still not well understood.

**Figure 1:**
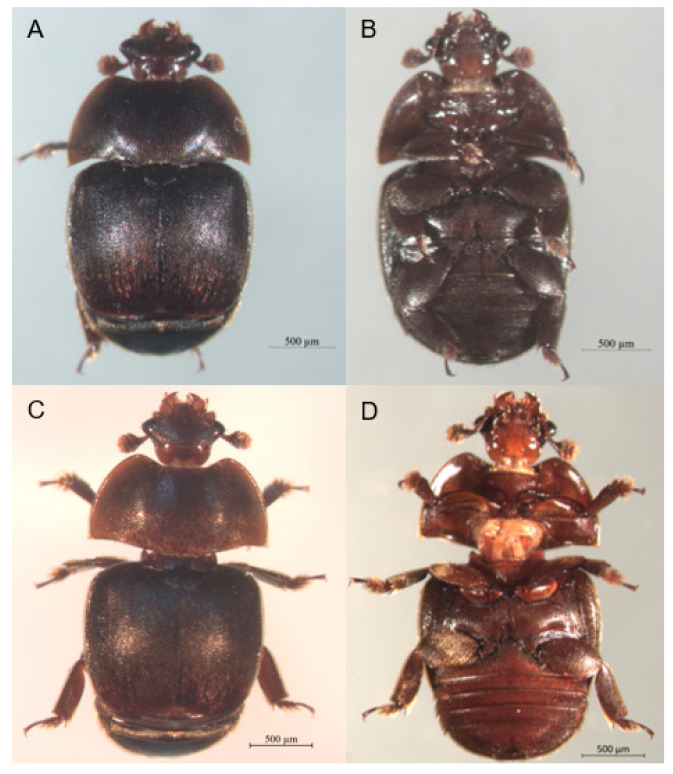
Photos of small hive beetles (SHBs). (**A**,**B**) Dorsal and ventral views of a female SHB; (**C**,**D**) Dorsal and ventral views of a male SHB. (photograph—Francisco Posada).

Small hive beetles (SHBs), *Aethina tumida*, are parasites of social bee colonies [33]. Native to Africa, SHBs have recently become an invasive species, and cause much damage to their hosts in their introduced range [33]. SHB adults and larvae feed on pollen, honey, and bee brood, but the larvae are the most destructive stage to bee nests. The adults emerge from the soil and invade bee colonies, where they oviposit in gaps and on combs. After hatching, larval SHBs feed on honey, pollen, and developing honey bee larvae. When they reach their final instar, SHB larvae leave the hive and burrow into the soil to pupate, where they complete their life cycle and emerge as adults before they invade bee nests again [33]. Remarkably, SHBs prefer to fly around dusk and are attracted to volatiles emanated from both bees, bee products, and symbiotic microbes, *Kodamaea ohmeri* [34,35,36,37,38], indicating that olfaction is involved in SHBs’ aggregation behavior and attractiveness to hosts [33]. The isopentyl acetate is a component of both bee’s alarm pheromones and volatiles produced by *K. ohmeri*, and thus appears to be a kairomone [30,31]. SHBs seem to differentially employ a few major compounds from the volatile extracts of colony materials and honey bees as cues, with some compounds (e.g., ethyl linolenate and palmitate) attracting SHBs, while others (e.g., tetracosane and oleamide) are repellent [39]. Besides these common behavioral traits, striking differences exist in the behavior of males and females (Figure 1). For instance, observations show that SHB males tend to infest colonies before females [40]. It seems that males are more sensitive to detecting host volatiles, however, prior study also shows that females are more responsive than males to the colony-related volatile sources [35]. However, the attractiveness of specific chemical components to both sexes remains unknown. 

Given these particular interactions with their hosts, SHBs are a promising model to investigate the evolution ORs and their role for host–parasite interactions. Even though SHBs appear to use olfactorial cues to find host colonies, the evolution of their ORs and their role in the interactions between SHBs and bee hosts are still poorly understood [33]. Yet, this knowledge is instrumental in understanding key features of the biology of the parasite such as its colony invasion behavior, as well as developing novel control strategies against this invasive pest. For example, the intervention of key SHB ORs can be applied to disturb their chemical communication and to develop effective biological agents against SHBs in apiaries, which may represent efficient and sustainable alternatives to the use of pesticides [33,41]. 

Since SHBs have high host selectivity and use colony volatiles as cues to search for host colonies [37], their OR genes might have evolved in response to odor cues from their host, as illustrated in other insects adapting to specific chemical environments [18,30]. To test this hypothesis, we here investigated OR gene evolution in SHBs and compared the OR gene expression in different body parts of female and male SHBs under two different statuses (colony odor-experienced/-naïve). To identify relevant candidate genes, we conducted phylogenetic analyses of ORs of SHB along with other beetle species (red flour beetle, *Tribolium castaneum*, and Asian long-horned beetle, *Anoplophora glabripennis*) and its native honey bee host, *A. mellifera.* We then performed tests of positive selection for OR orthologues in two OR clusters of interest to see whether SHB ORs have been positively selected because of the association with olfactory adaptation to host. Finally, we tested the expression of selected OR genes across different groups of SHBs reared in the laboratory to compare OR gene expression between body parts, sexes, and to test the effect of contact with colony odors. Altogether, these data highlight the evolution of SHB ORs, the parasite’s olfactory adaptations towards the volatiles of its host colonies, and the relative function of ORs in different body parts.

## 2. Results

### 2.1. Selection of Candidate OR Genes

**Figure 2:**
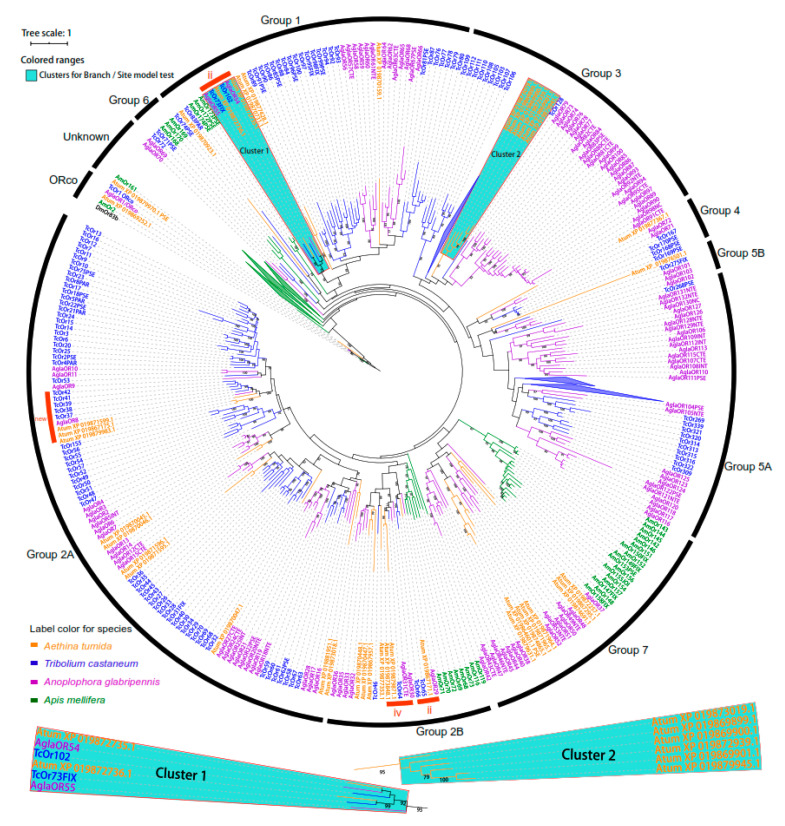
Dendrogram of the odorant receptor (OR) gene family of five species. The sequence similarity tree was reconstructed using maximum likelihood methods with RaxMLv8.0 using amino acid sequences of OR genes (>200 aa). The OR coreceptor (Orco) orthologue was used to root the tree [28]. ORs of *Aethina tumida*, *Tribolium castaneum*, *Anoplophora glabripennis*, and *Apis mellifera* are shown in orange, blue, violet, and green, respectively. The numbers above branches indicate bootstrap support value bigger than 75 (RaxML; *n* = 1000). The tree was divided into nine major groups (Group 1, 2A, 2B, 3, 4, 5A, 5B, 6, and 7) in black arcs [30], plus one unknown group and the group of Orco including members of Orco from other species, namely, TcOr1/ORco, Agla OR1/ORco, Atum XP_019869252.1, AmOr2, and DmOr83b. Names of odorant receptors for other species were kept as originally described [28,43]. Conserved lineages of ORs were shown in red arcs and kept the names of ‘ii’ and ‘iv’ given when they were previously identified, and a ‘new’ conserved OR lineage was discovered in this study. Several clades that have lineage-specific expansions were collapsed (see triangles in the tree). Branches marked with Cluster 1 and Cluster 2 were tested for positive selection with tests of branch models and site models in PAML [44]. The scale bar displayed the branch lengths of the tree. The dendrogram was visualized in iTOL [45] and edited in Adobe Illustrator^®^ CS6 (Adobe Inc., San Jose, CA, USA).

**Figure 3:**
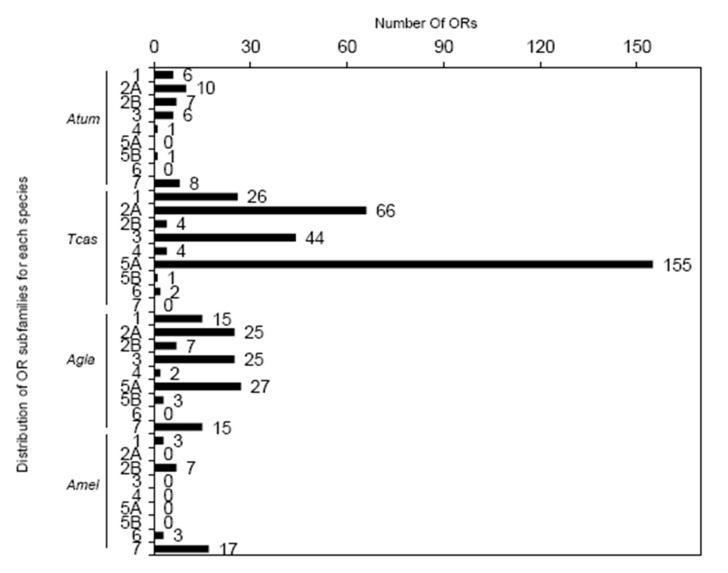
Distribution and count of odorant receptors (ORs) among OR subfamilies. The numbers or numbers with letters on the right of the y-axis (1, 2A, 2B, 3, 4, 5A, 5B, 6, and 7) represent the nine major OR subfamilies. The numbers on the right of the bars indicate the number of ORs for each gene subfamily. Atum, Tcas, Agle, and Amel stand for *Aethina tumida*, *Tribolium castaneum*, *Anoplophora glabripennis*, and *Apis mellifera*, respectively.

After Pfam filtering and removing redundancy based on the results of the HMMER profile search against SHB protein database, we obtained a total of 41 putative functional ORs (each having >200 amino acid). Coleoptera ORs used in the phylogeny were grouped into nine major OR subfamilies (Group 1, Group 2A, Group 2B, Group 3, Group 4, Group 5A, Group 5B, Group 6, and Group 7, Figure 2). Among these groups, common origins were observed for Groups 2 + 7 and Groups 3 + 4 + 5, respectively, but with weak supports. Order-specific diversification that most ORs from *A. mellifera* clustered in separate branches were observed, but twenty-five genes were associated with Group 2A and Group 7. Species-specific expansions were also observed for most groups except for Group 4 and Group 5B. One obvious SHB-specific expansion (gene duplication) of six ORs was presented in Group 3. SHBs also suffered gene losses, for instance, Group 5A had OR orthologues from both *T. castaneum* and *A. glabripennis* but not for *A. tumida* (Figure 2 and Figure 3). The possible function of the two OR subfamilies with gene losses and duplications were unknown, but being suggested to have the function for olfaction based on the search of functional domains. We confirmed three conserved lineages within Groups 1, 2A, and 2B (Group 1-ii, Group 2B-ii and iv, Figure 2) [30], and also identified a new group marked with the red arch ‘new’ and supported by strong bootstrap values (Group 2-new, Figure 2). In addition, ORs in Group 2B-ii including XP_019867171.1 were orthologous to the characterized pheromone receptor McarOR3 of the beetle *Megacyllene caryae* [42,43].

For the tests of branch models on OR orthologues from three species (Cluster 1, Figure 2), the alternative model (*ω*1 = 0.103, *ω*2 = 0.003, *ω*3 = 0.004) fitted the data better (*p* < 0.01). The greater *ω*1 for SHBs reflected SHB ORs experienced stronger selection pressure, which led to more non-synonymous nucleotide substitutions. The site model test could not reject the null model M7 (*p* > 0.05) testing whether the evolutionary forces operated on the duplicated OR subfamily (Cluster 2, Figure 2). Nevertheless, the test of the alternative model (M8) reported five potential signatures of positively selected sites of amino acid residues, though not statistically significant (Bayes Empirical Bayes analysis <95%). These potential positively selected sites were not presented by the more stringent site model tests, M2a (selection).

### 2.2. Expression Patterns of Odorant Receptor Genes

The expression level of gene ribosomal protein S5 (AtumRp311) was the most stable among the three candidate reference genes (data not shown). Five out of nine OR genes were successfully measured by quantitative real-time polymerase chain reactions (qRT-PCR) including one Orco, one OR orthologous to another species, and three ORs from Cluster 2 of duplicated SHB ORs (Table S1). Notably, although we optimized the PCR conditions and designed new primers for the other four OR genes, the primers can be aligned to multiple genes.

**Figure 4:**
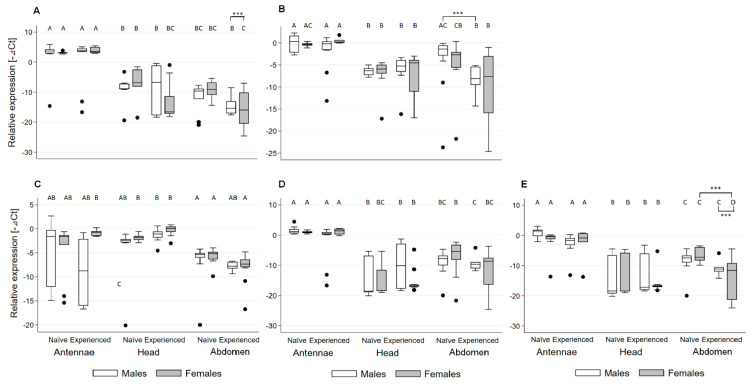
The relative expression pattern analysis (−ΔCt) of six odorant receptor (OR) genes for samples of different sex (male and female), status (colony odor-experienced/-naïve), and body parts (antennae, heads, and abdomens) in SHBs. (**A**–**E**) shows, respectively, the relative expression patterns of OR genes coding proteins: OR coreceptor (ORco) (XP_019869252.1) and ORs (XP_019872736.1, XP_019869899.1, XP_019872939.1, and XP_019869900.1). Ct values reflecting target gene transcripts were subtracted from Ct values of reference gene AtumRp311 ((−ΔCt) to show relative differences in gene expression. All analyses were performed using 15 biological replicates. Different capital letters above boxes indicate significant differences (*p* < 0.001) determined by STATA 15 software (more details please see data analysis).

Gene expression analyses revealed that the five OR genes were predominantly expressed in antennae (*p* < 0.001, Figure 4). In contrast, low expression levels were detected in both heads and abdomens. The status (colony odor-experienced/-naïve) and sexes (male/female) of individual beetles showed no significant influence on the relative gene expression in antennae and heads (*p* > 0.05, Figure 4). However, significantly decreased gene expression was observed for the OR coding XP_019872736.1 in colony odor-experienced male abdomens compared to naïve male abdomens (*p* < 0.001, Figure 4B). Likewise, abdomens from experienced females revealed significantly decreased relative expression when compared to the naïve female abdomen for the OR gene coding XP_019869900.1 (*p* < 0.001, Figure 4E). Between males and females, no obvious sex-biased expression was detected for the five tested ORs.

## 3. Discussion

The phylogenetic analysis of ORs of *A. tumida* compared to ORs from its host *A. mellifera* along with two other beetle species revealed that SHB has suffered gene losses and undergone duplications. This analysis also discovered an OR subfamily of putative pheromone receptor, and a group of ORs with obvious gene duplications for SHBs. Additionally, evidence of selection was detected for both OR orthologues among species and the duplicated ORs of SHBs. Expression patterns of five OR genes were measured and showed that exposure to colony odors and sex had no significant influence on their relative expression in antennae and heads. However, two of these ORs (coding proteins XP_019872736.1 and XP_019869900.1) showed lower expression in the abdomens of beetles exposed to hive odors as opposed to naïve beetles.

The OR phylogeny showed that 99% of genes of beetle species were placed within the previously described nine subfamilies [30], suggesting that our phylogeny was well-matched with the previous study of ORs in beetles [30]. This analysis allowed us to study the evolution and diversity of SHB ORs among different species. Particularly, losses of OR genes were observed in Group 6 for SHBs because this subfamily had only OR orthologues from *T. castaneum* and *A. glabripennis*. Gene losses in Group 6 have been previously shown for other beetle species [30]. Since SHBs usually parasitize colonies during their entire lifetimes excluding the period of pupation in soil [33], it is conceivable that SHBs have lost OR diversity observed in other insects in part due to their preferred host and specific chemical environment [18,30]. In addition, the observed OR expansion (Cluster 2 in Group 3, Figure 2) in SHBs might be caused by gene duplications reflecting adaptation to bee hosts. The gene duplications of ORs in Group 3 seem especially strong candidates for playing a role in host finding. Unfortunately, the function of the OR subfamilies and patterns of gene losses and duplications remain unknown, and no orthologues were functionally characterized, perhaps because the enormous diversity of ORs hampered their functional characterization [30].

Apart from the expected conserved ORco genes, our analysis confirmed three conserved OR lineages (Group 1-ii, Group 2B-ii and iv, Figure 2) found in all three beetle species, indicating that these conserved ORs might be fundamental for chemical communication in beetles. Even though the function of ORs in most groups is unknown, the phylogeny of ORs across species/orders can offer resolutions of their potential functions based on ORs that were functionally characterized from other insects. Indeed, Group 2B-ii appeared to be a clade of pheromone receptors because AglaOR29 is orthologous to the characterized pheromone receptor McarOR3 of the beetle *Megacyllene caryae* [42,43]. A future functional characterization experiment will be interesting to examine whether this OR coding XP_019867171.1 has a similar function for SHB, e.g., recognizing their sex/aggregation pheromones. If this is verified, this OR could be used for the development of useful methods for pest control.

Olfactory adaptations associated with OR gene duplications and evidence of positive selection at OR loci have been described in *D. suzukii* [32]. In the site model tests of this study, signatures of positive selection were also detected for the duplicated ORs for SHBs (Cluster 2, Figure 2), which may result from the adaptation towards bee hosts [18,30]. Regarding the site model tests, even though the null model (M7, beta) was not rejected, the detected signatures suggested that positive selection might be shaping the evolution of the newly duplicated OR genes of SHBs. These ORs were likely to play an important role in host recognition or response to volatiles from honey bee hosts. Furthermore, for the branch model tests conducted on OR orthologues from three species (Cluster 1, Figure 2), a higher *ω* value (*ω*1 = 0.103) for the SHB OR (XP_019872736.1) among different species was found. This suggests that these ORs have experienced stronger selection pressure even though they were under negative selection (*ω* < 1) to maintain their functional conservation [44]. These results shed new light on the fundamental roles of these ORs for the chemical communication of beetles.

Our gene expression data were consistent with previous research, showing that most ORs were predominantly expressed in antennae of both sexes [4,21,23]. The results of the present study also suggest that the abdomen of insects may be involved in their olfaction. Among different body parts, we only found obvious differences between groups with and without exposure to colony odors in the SHB abdomen. Although the antennae are the major chemosensory structure of insects, mouthparts, legs, and abdomens as well as other parts (maxillary and labial palps) can also be involved in insect olfaction [2,4]. Moreover, the presence of olfactory sensilla in the abdomen of insects indicates that the OR genes in the abdomen might be involved in their olfaction [46]. Altogether, this suggests that insects may have evolved a wider variety of solutions for chemoreception than previously assumed [4].

The downregulations of the two ORs (XP_019872736.1 in males and XP_019869900.1 in females) for colony odor-experienced abdomens were likely the result of SHBs’ olfactory regulation of maintained exposure to bee volatiles in colonies. To appropriately process strong and/or maintained odors, the insect olfactory system has to be able to downregulate the response in a use-dependent manner, i.e., the adaptation of the OR response caused by long-lasting stimulation of sufficient strength [19]. Use-dependent expression of specific OR genes has been observed in other insects, and can provide crucial information about species’ chemical sensitivity to environmental chemicals [20]. For instance, a dramatic downregulation of an OR gene was observed in the mosquito *Anopheles gambiae* after feeding blood, which caused a significant reduction in their olfactory responses and sensitivity to hosts [20]. Similarly, the two ORs of SHBs may be involved in olfactory recognition and adaptation to host odors. 

The downregulation of ORco is considered as another mechanism contributing to the use-dependent manner [19]. In this study, we found the downregulation of ORco (XP_019869252.1) only for colony odor-experienced female abdomens when compared with colony odor-experienced male abdomens (Figure 4A). Such a causal relationship was not well reflected here because no significant difference between the abdomens of female beetles exposed to colony odors and the naïve ones were detected. Alternatively, these expression patterns of ORs might also be linked to other internal events such as oviposition [47,48], cell chemotaxis (e.g., sperm orienting to eggs) [22] and other uncharacterized events. Previous studies showed that some ORs can be more expressed in the egg-laying female abdominal tissue of flies when compared to the antennae [46,47], and in some cases, oviposition can be induced by chemical cues from hosts [48]. Although such results were not controlled here, it is important to expand our knowledge about the role of ORs for SHBs and other beetles in the future by comparing the differential expression of ORs in different tissues.

Females SHBs are generally more responsive than males to different volatile sources of colonies [35], and male SHBs tend to infest colonies before females [33]. However, no striking sex-biased expression was observed here for the five measured ORs. Nevertheless, the decreased expression of the two above mentioned ORs (XP_019872736.1 and XP_019869900.1) in colony odor-experienced abdomens were likely to reflect their role for the differential response of males and females to volatile sources from host colonies. 

Altogether, the identified putative pheromone receptor (XP_019867171.1) and another two OR genes (XP_019872736.1 and XP_019869900.1) will be the key candidate receptors for recognizing pheromones or kairomones, e.g., volatiles from host honey bees or associated microbes, *Kodamaea ohmeri*, which are carried by SHBs and generate attractants for SHBs [37,38]. Finally, even though the differential expression of OR genes were found only in the abdomens here, antennae remain the main olfactory organs which should harbor other ORs that play an important role in SHBs’ olfactory response to the host-related volatiles. Further studies are needed to understand the mechanisms underlying SHBs’ differences of olfaction towards bee hosts and to further assess the role of OR genes for host–parasite interactions. For instance, the suppression of OR gene expression by RNAi will be helpful to confirm the role of selected OR genes.

## 4. Materials and Methods

### 4.1. Small Hive Beetle Preparation

SHBs were reared to determine the putative function of distinct OR genes for detecting host volatiles. Adult SHBs used in the experiments were maintained using a variation on standard laboratory rearing methods [49] at the USDA-ARS Bee Research Laboratory, USA. In brief, rearing was initiated with 50 locally collected SHB adults. Beetles were then kept in 8.5 cm × 30 cm × 21 cm plastic containers in a dark incubator (30 °C, ~60% relative humidity) and fed honey and water [49]. The beetles were provided with BeePro^®^ pollen patty containing 10% fresh pollen and five oviposition slides constructed by sandwiching one coverslip in the middle of two standard glass microscope slides and secured slides together using a jumbo paperclip [49]. After three days, oviposition slides containing SHB eggs were placed in a new plastic container. Emerging larvae were provided with pollen patty as above ad libitum until they reached the post-feeding stage [50]. Post-feeding larvae were transferred to pupation buckets with sterilized sand (30 °C, ~60% RT) and held in constant darkness until adult emergence. After 28 days, emerging adults of this second generation were collected and sexed [36,49], and males and females were kept separately in two new containers under the same conditions as described above.

A week later, female and male SHBs from the second generation were divided into two groups: colony odor-experienced and colony odor-naïve. To do so, males and females were separately introduced into four small honey bee queen cages each containing 10 individuals. Two cages of each female and male SHBs were placed between honey frames in a honey bee colony for four days to expose beetles to the hive temperature and chemical environment. In parallel, two cages of each female and male SHBs were set separately in the laboratory to obtain colony odor-naïve beetles. All SHBs were supplied the same sugar water (2 mol/L) during these conditioning days. This way, four SHB groups were created: colony odor-experienced males, colony odor-experienced females, colony odor-naive males, and colony odor-naive females.

After four days inside honey bee colonies, the cages housing experienced female and male SHBs were removed from honey bee colonies and immediately placed on dry ice to freeze-kill the beetles. Meanwhile, naïve female and male beetles were also freeze-killed on dry ice. The beetle samples were stored at −80 °C until RNA extraction directly after sampling.

### 4.2. Sample Dissections, RNA Extraction, and cDNA Synthesis

The two antennae of SHB individuals were removed from the SHB head and quickly placed into a pre-chilled 2 mL tube containing 50 μL of TRIzol Reagent^®^ (Sigma, St. Louis, MO, USA). Additionally, for every individual, heads, and abdomens were separated and placed into tubes containing 100 and 250 μL of TRI Reagent^®^, respectively. Different body parts were immediately homogenized and kept on ice until RNA was extracted. Total RNA was extracted from each SHB body part (antennae, head, and abdomen) for 15 beetles per each of the four groups (Table 1) using TRI Reagent^®^ (Sigma) following manufacture’s protocol. Total RNA concentration and purity were measured with a spectrophotometer (Thermo Scientific NanoDropTM 1000, Wilmington, NC, USA). The concentration of total RNA was equalized across samples with ddH2O. A volume of 1.5 μg of total RNA was treated with DNase I to remove genomic DNA and was used to synthesize cDNA using Superscript II according to the manufacturer’s instructions.

### 4.3. Selection of Candidate OR Genes

Since ORs were never studied in SHBs, we first identified reliable predicted ORs in SHB to select putative OR genes involved in honey bee cues recognition. To do so, the curated profile HMM [51], constructed by multiple sequence alignment of the OR domain (7tm_6: PF02949) from Pfam [52] a database of protein domain models, was used to search as many as reliable OR homologs against the reference set of SHB protein sequences (project accession number: PRJNA361278) at default cutoff setting of HMMER version 3.1b2 [51]. ORs without functional domains ((7tm_6) were filtered out by searching against Pfam with the Web CD-Search Tool [53,54], and then repeated sequences were removed. The ORs with sequence length >200 amino acids of ORs from *A. tumida* and three other species (the beetles *T. castaneum*, *A. glabripennis*, and the honey bee, *A. mellifera*) were used to reduce the impacts of aligned gaps on the phylogenetic analyses as well as the sequence selection analyses (Supplement S1). Amino acid sequences were aligned with MUSCLE algorithm in MEGA 7 [55]. A phylogenetic tree was constructed using RAxML version 8.0 with 1000 bootstrap iterations (-N 1000 –m PROTGAMMAAUTO –f a) [56]. The tree was visualized using the online iTOL [45] and rooted with the conserved ORco orthologues according to [28]. We then grouped the ORs in the tree into different subfamilies referring to the previous OR grouping among Coleoptera and Hymenoptera [28,30].

Given host adaptation in insects often accompanied by olfactory adaptation and involved OR gene selection and duplications [14], to select candidate ORs involved in SHBs’ host-finding behavior and/or pheromone communication, we investigated the ratio of non-synonymous/synonymous nucleotide substitutions (*ω* = dN/dS) of OR orthologues among species, and duplicated ORs of the SHB were tested for potential positive selection events using PAML package version 4.9d [44]. This will provide information about whether these genes have undergone selection pressures, i.e., positive selection (*ω* > 1) or neutral/negative selection (*ω* ≤ 1) [44].

The branch model of PAML (model = 2, NSsites = 0) was applied to test if OR orthologues among species (Cluster 1, Figure 2) evolved under positive selection. This model detects putative positive selection acting on particular lineages [44,57]. Alignment files of codon sequences were prepared by web server PAL2NAL [58] with their mRNA sequences and protein sequences as guides. The same tree topology of this cluster was used in the tree file needed for PAML. Referring to the PAML manual [44] and a previous study [59], two SHB ORs in Cluster 1 labeled with “#1” and “#2” symbols on branches of SHB ORs were determined as the foreground branches, with the rest representing background branches to allow the *ω* ratio to vary among branches. The alternative model (model = 2, NSsites = 0, fix_omega = 0) and the null model (model = 0, NSsites = 0, fix_omega = 1, omega = 1) were tested for this cluster. Then, the likelihood ratio tests (LRT = 2(lnLH1 − lnLH0) and the degree freedom (npH1 − npH0) were used to perform chi-square tests to test which model fits the dataset significantly with the pchisq function in R version 3.6.1 [44,60].

Since most codon sites are highly conserved to maintain protein function [61], the site models of PAML, M1a (neutral) vs. M2a (selection) and M7 (beta) vs. M8 (beta & *ω* ≥ 1), were applied to test whether the positive selection was operating in duplicated SHB ORs (Cluster 2, Figure 2), which might be associated with unique olfactorial adaptations [59]. As in the branch model test, the alignment files of codon sequences were prepared by PAL2NAL [58] and the same tree topology in Cluster 2 was used in the tree file for PAML. The log-likelihood values (lnL) of 4 different NSsites models were computed, that is, pairs of alternative models (H1) and null models (H0): M1a (neutral) and M2a (selection), M7 (beta) and M8 (beta & *ω* ≥ 1). Then LRTs were conducted to test which model fits the dataset better.

### 4.4. Analyses of Odorant Receptor Gene Expression

To assess the function of ORs of SHBs detected in the tests of positive selection, we conducted analyses of OR gene expression. We tested three reference gene primer pairs, with two pairs designed from two conserved gene regions of the SHB genome including gliceraldehyde-3-phosphate dehydrogenase (GADPH) and ribosomal protein S5 (AtumRp311), and another pair from a previous study (*A. tumida* GAPDH-F and *A. tumida* GAPDH-R) [62]. Altogether, nine OR genes of interest were tested (Table S1). These included one ORco coding protein (XP_019869252.1), two OR orthologues to other insect coding SHB OR proteins (XP_019872735.1 and XP_019872736.1), and six duplicated ORs which were shown under potential positive selection. Gene-specific primers were designed with Primer-BLAST [63] (Table S1). Reactions were performed in 20 μL mixes consisting of 1 μL cDNA template, 0.4 μM each primer pair, and 1× SYBR^®^ Green Supermix (Invitrogen, Carlsbad, CA, USA). Negative controls were established without the cDNA template. qRT-PCR experiments were carried out in the iCycler (Biorad, Hercules, CA, USA) under the following conditions: 95 °C for 30 s, then 50 cycles of 95 °C for 5 s, 60 °C for 30 s, followed by melt curve analysis to confirm PCR product identity and integrity. Data were used for baseline correction and calculating PCR amplification efficiency.

The expression pattern of five out of the nine above-mentioned ORs was successfully measured in antennae, heads, and abdomens of SHBs (primer names in bold, Table S1). Samples were grouped to 12 treatments upon their sex (male/female), status (colony odor-experienced/-naïve), and body part (antennae/heads/abdomens) (Table 1). The threshold (Ct) values of the target genes were normalized with the reference gene AtumRp311 (Table S1). Differences (-delta Ct) of the relative expression of the five successfully amplified genes were assessed using generalized linear mixed (regression) models (GLMM) fitted using STATA15 (StataCorp, College Station, TX, USA). Multiple pairwise comparisons (Bonferonni test) for the five OR genes and treatment groups were obtained by using the mcompare (Bonferroni) function [64]. All figures were created using STATA15.

## 5. Conclusions

Olfaction is crucial for SHBs’ host recognition and chemical communication. Here we detected that the major chemosensory gene family of the OR gene has suffered gene losses and undergone gene duplications for SHBs, and identified three important candidate ORs involved in SHBs’ volatile recognition. Apart from the main olfactory organs of antennae, the abdomen also seemed to also play a part in the olfaction process. Our findings revealed SHBs’ olfactory adaptation towards volatiles from the host, which helps to resolve key component ts of the interactions between the parasite and its hosts and develop novel control strategies, i.e., chemical traps and gene intervention, against this invasive pest. Future functional characterization of the three OR genes or of more specific colony volatiles, using gene knockdown, RNASeq, and Y-tube olfactometric bioassay, may enhance our understanding of SHBs’ olfactory response towards honey bee hosts.

## Figures and Tables

**Table 1:** Overview of the 12 treatments used for the odorant receptor gene expression profiles. Total RNA was extracted from each SHB body parts (antennae, head, and abdomen) for 15 beetles for each of four groups (male–naïve, male–experienced, female–naïve, and female–experienced).

Sex	Status to Colony Odor	Tissue	Group
Male	Naïve	Antennae	1
		Head	2
		Abdomen	3
	Experienced	Antennae	4
		Head	5
		Abdomen	6
Female	Naïve	Antennae	7
		Head	8
		Abdomen	9
	Experienced	Antennae	10
		Head	11
		Abdomen	12

## Supplementary Materials

Supplementary materials can be found at https://www.mdpi.com/1422-0067/21/13/4582/s1. Table S1. SHB odorant receptor gene-specific primers for quantitative real-time PCR. Supplement S1. Odorant receptors protein sequences used for phylogenetic analysis.
