# Supplementary material for: Gene Expression and Functional Analyses of Odorant Receptors in Small Hive Beetles (Aethina tumida)
Source: Int J Mol Sci. 2020 Jun 27;21(13):4582. doi: 10.3390/ijms21134582 (PMC7370172; doi:10.3390/ijms21134582)
Supplement: Supplementary file 1 [file ijms-21-04582-s001.pdf]

Table S1. SHB odorant receptor (OR) gene-specific primers for quantitative real-time polymerase chain reactions. Gene-specific primer pairs were designed with Primer-BLAST. *Aethina tumida* 28S ribosomal protein, mitochondrial (LOC109601756), was used as a reference gene. Primer AtumRp311 was for amplifying the reference gene; Primer AtumORco was for the SHB OR co-receptor (ORco) gene; Primer 1 and Primer 2 were for SHB ORs that were OR orthologues to other species; Primer 3, 4, and 5 were designed for SHB duplicated OR genes which were under potential positive selection. Primer names in bold are those working for our experiments.

Names of Primer	Sequence (5'->3')	Product Length (bp)	Coded Proteins	Remark
**AtumRp311-F**	TGTGTGGCCTGGTTTGTCAG	78	28S ribosomal protein	Reference gene
**AtumRp311-R**	TCAGGATCCTCTGGCAGTTG			
GAPDH-F1	TTCGAGATCGTGGAAGGTTTG	76	gliceraldehyde-3-phosphate dehydrogenase	
GAPDH-R1	CAGAGGGACCGTCGACAGTT			
GAPDH-F2	GAGACCCAGCCAACATTCCA	85		
GAPDH-R3	GCCTTCTCGATGGTGGTGAA			
**PrimerORco-F**	TTAAGGTAGCCGGCCTTGTG	117	XP_019869252.1	ORco
**PrimerORco-R**	ATCGAAGAGTGTGCAAGGCA			
**Primer1-F**	TGGTGCGGTGTAGATTTCCC	207	XP_019872736.1	Orthologues among species
**Primer1-R**	ACTGACAACAGGGCACCAAA			
Primer2-F	TGTGCGCCACTATGTGTTCT	94	XP_019870735.1	
Primer2-R	TCCACTCTATCCCTGGTTGGT			
**Primer3-F**	TGCTTCACTTGCTGGGGATT	131	XP_019869899.1	Duplicated ORs
**Primer3-R**	CGAGTTGTCTTCCCACTCGT			
**Primer4-F**	GGCACCAATTTCGTTTGGCT	173	XP_019872939.1	
**Primer4-R**	TCCCCATTGTCCTGATGCTC			
**Primer5-F**	CGGATCATGTCTTTGCCCCT	193	XP_019869900.1	
**Primer5-R**	CCGAACTCTCGTCCATCACA			
Primer6-F	ACGTCGTGGCGATTTTGTTC	77	XP_019873019.1	
Primer6-R	GACATCTGCACTCAGGTCCA			
Primer7-F	TGCATTGGTATGAGCCCTGA	131	XP_019869901.1	
Primer7-R	GCGTCCATGTAACTTGCGTC			
Primer8-F	GGCCACTTTGGTTACGGTGT	82	XP_019879945.1	
Primer8-R	ACACGTCCTCCTCTTCGTTTC			

Supplement S1. Odorant receptors protein sequences used for phylogenetic analysis.

>XP_019869252.1

MMKFKVAGLVADLMPNIRLMQGAGHFMLNYHADNSGALHTLRLGYCCVHLVLMLFQYGTILGNLVVDR
EDVNELAANTITTLFFAHCITKFIYFALRSKLFYRTLGIWNQANSHPIFLESNNRYHALALKKMRRLLVIIVCA
TLFSGIAWTAITFVGDSVKSKKDPENENETITVEIPRLLIKSWYPWDAMSGTAYYATLVFQVYYVFFALAQAN
LLDSLFCSWLIFACEQLQHLKEIMKPLMELSATLDTYVPKSADLFRAPSANNDIMENEYNDKNEDLNLKGIY
STRQELGGHFRSGALQTFGQGGGGVGPNGLTKKQELMVRSAIKYWVERHKHVVRLVTAIGDAYGVALLLH
MLTSTVMLTLLAYQATKIDGVNTYAASTLGYLLYALGQVFHFCIFGNRLIEESSSVMEAAYSCHWYDGSEEA
KTFVQIVCQQCQKAMSISGAKFFTISLDLFASVLGAVVTYFMVLVQLK

>XP_019879970.1(PSE)

FAHCITKFIYFALRSKLFYRTLGIWNQANSHPIFLESNNRYHALALKKMRRLLVIIVCATLFSGIAWTAITFVGD
SVKSKKDPENENETITVEIPRLLIKSWYPWDAMSGTAYYATLVFQVYYVFFALAQANLLDSLFCSWLIFACEQ
LQHLKEIMKPLMELSATLDTYVPKSADLFRAPSANNDIMENEYNDKNEDLNLKGIYSTRQELGGHFRSGAPP

NLRSGGGGVGPNGLTKKQELMVRSAIKYWVERHKHVVVRLVTAIGDAYGVALLLHMLTSTVMLTLLAYQA
TKIDGVNTYAASTLGYLLYALGQVFHFCIFGNRLIEESSSVMEAAYSCHWYDGSEEAKTFVQIVCQQCQKAM
SISGAKFFTISLDLFASVLGAVVTYFMVLVQLK

>XP_019870046.1

MVTSVSYAVLTFSMNLVKLAFMYKNIDKILGMMSLLRSEPFRIKCREHEELAQASKTFYLRVFYFCLILGFNTD
IFWIVYPLTGAEKTTPFRAWFPFNYEVTVYFYVIYAFQITAAIMNTVICLNLDCFTSSMLIQAGLQCDMMCITL
RNLNKFVINENGKMIETDTEDEDEKEYSNRLLKNLIYCIKHHIQIKKWAKDIEDIHFISVFLLFVVGTFVLCASL
FEISLVDIMSMEFGMLLSYSSAILTELFLYCYFGNELIYKSEQIFHAIYDCPWLNCDIMFKKTIVLFLIGTQRPIVV
YVGGLFTMSLPVYVAILRSSYSYFTLLKNAWFPFNYEGTEYFKFVYGFQAMTAVFETMMCVNMDCFSSSLMI
QAGLQCDMMCNTLKNLEKFKVNDDGGMVESDAQDEDTEAYSNRLLKNLIYCVKHHIEIKKWAKDIQDIHS
ISICFQFGIAQKFILLSNYYLAILTEIFLYCWFGNELNYKSSQLYHSVYECQWMDCNIKFKKAMLIFMMGTEKPI
TVYVGGLFPMSLPIYVAILRSSYSYFTLLQNVFLK

>XP_019871595.1

MRDIIQNSFLVNLIIMRITGLYPVKFFNEYYVIYAWVFYALFTVPVPVLSLAELYIDKVTDFEMLSDTVFMDAQI
GIFIIKFLPFIVNVKGIDATFQTLKSKMFTDYLPEQQTFVDNAVYLSKQCFYLFWAFCVASVGNAANTSAAVD
TLIPGLLCHAAAQIQILKDNLIHLDERTNEEVNRTYKQLNMDRIQLKNKLLFEKVADCVKHHKAIRDFVDQI
EDSYNLVLFSQLFGSSLVEPLSITFFQMFVFMLTMNLEIFLFCYYGSVLYEESVTINEAIYMGNWYECDIKSKKA
LLILMEGAKRPMQITAGKILYVSLELFMGILKKSYSIFAVLKNFT

>XP_019870047.1

MAKILNRLAFGSVKNHAFEIVTSVLYLITQAGHDFLKISNTVFIVVEFIIVTFKQSVMKMSLSKFTSTWNMVDS
EMFNLPQTQSMCVANWALVPYITPERSLQTGIWLPFNPLDDLKSYVAATTYISISCLHASYFTASIDTLSAGLI
MQAVGQLELLNDNLKNLHKCAQTDDPDAVYKKIIECVKHNEYIYKYVKEVEECLSIIIFAQFLTSTIVICMTC
VILTLLEVSSFRFYMLICFLITMFFQLFFYCYYGTLLYQESNNVIDAIYSGEWYELDEKCKRALVVLMVRAQKPF
LLSAGKFFTVSLETFTVVLRRAYSLFAVLNNY

>XP_019871964.1

MSLMIIEMFKESDNPQIMGETLSYSTLYIVMYVSLLYIESKNVQDLYRAIDKKEEALLKTANSSIRDIYEKHTEIN
NVVGAQIFGVCGVFCCCCLVVHHVKVFEGEWAFIFFQWFPFNADNYYIPILLNQLCFLCVALFYMMYTKLA
VIAFSTYILAHIKVLQHFIVNIDHYAKYVLENDGLEDMHDARYVVLKQCVQSHNEIIEYVDLVRQNTSTFILL
DFMLTSLQLGSILISLMTEGLNERLVDKVVLWYYFTLNIHWMWVTYFYGNEIMVESVNISVLAYSEVEWNNF
DLKAQQGLIMMMAK

>XP_019879983.1

MSSEEHFNFREIVSLNIKALHIFGYFRPDTDSKLQRMLYEIYSIFAVGLLLFVLTLSEVANMYVIFGDFEQMIAD
SFLSMSHWIHYVKLYVLRRGRGDVIWKLIHDIERREYLPKSPTQLRILETNVRNSKIIFKTFFSMCIMCGICFTLI
PFLGAGPGNVGKKKLPYGGWYPESYFVASYIYQIFASPLTGLGNISIDTFITSMIMVASSQLEMLNDSVKNLKQ
FAMEKAAKNGRNLEEEMNLCLIECVKQHQYILDYTKTLNKVFTECIFGQFLFSVIIICFTLFKIALVPITDVQFL
TLILYLMCMLLEIFLWCYYGNEIIIHSNELTNSAYFTHWAGCSKRFGKNLVYFMTRSQLTLKMFAGGFFTLSL
QTFVTILRSSWSYFAVLNHMNSVKEEMGPLM

>XP_019867171.1

MSSEKNLLVSVPKEYFKVPIVLLQTIGISLKPIENEYLRFLYRIYSFILISFSIYIFLIIEFVECLILNPSVDNITFGLSYG
VTHILGSAKVTLFLIKKNSLIRIIKILESGVFAPNITRGGIKEFHLINDAVKRANSQAYIFFTMAFMIIGLRGVYA
MVDSGEDVEEFIYGTNETYIKHIKTMPYTTWFPFNKDKQPYYAIGFTYQIASTYIYGLFIGATDAILCGFLIHIQ
AQFRILKISLVTLLERSKQGSNVKDSHEDVKTITLPAGCIKVPLLTRQVNVELKKHCRKIVQHHQEILNLCIYIE

DEICYLMLMQFLSSLFIICFQLYQLSSISPANIGFISMSLYLSLMIYQLYNYCNNGDEVRELSESLGREIFAIEWITF
DEPVKKYLLMMIMRTHRASVFTAGKFALLSLQTFMAIVRGSGSYFMVLRQMNN

>XP_019871596.1

MFGSPFDAFHSTIVFTLVYAFVFIGVGNAANTSAAVDTLIPGLLCHAAAQIQILKDNLVHLDERTTEEVNRTY
KQLNMDRIQLKNKLLFEKVVDCVKHYKAIRRVKPLSITFFQMLVFLFTMNLEIFLFCYYGSVLYKESVTINEAI
YMGKWYEYDIKSKKALLILMEGVKRPMQITAGKILYVSLELFMGILKKSYSIFAVLKNFT

>XP_019870447.1

MHGYHANFFETIVLTLKLFGIWVPNNLSNFKEKLYHFYMCTFLGIAWLYFISEIIIIKDTHKSLFDLISNIGLLIT
HVVGVIKFSTLFFRRKKIEEMMQILQSNRFKYESVGDFKPSDMFNESKEISSKHTWCLFVVYGLVGISAHFSGLI
NMGGISSDEVINCSDYVAYYSYFPFNMDSIGKCHILFFLMDVPLIIFALYIAAFDSLFGTLANCLKCQLLIVCKA
FTSIRERVLTKLNLPQDFQIFRDEEHPMLEQALFEEMKRCTVHLSYLIRVHTGIEEIFTYVILTQSIVSLIVFATCL
YVGSSVPLMSPEFFSHMEYFVAVFMQFTTYCWYGSEIIFAGHSVGPSIFNSDWTSTSERFKKAMILNIIRMRNG
LHFSIGKFTPLTVATLITVLRGSFSYFAVFKSME

>XP_019872939.1

MITIVPLVTSRSLQMYVYVPEALGTNFVWLLESLIIPLITVSVFAFDYLIFTFLQLTIVQFQLSNSSIRKLNFGEHQ
DNGELRTQLVKLVKHHIFMLKFTKRLQRIMSLPLLVQLFNTVSAFCIEMYLMRYNVDLGQGIIGSLHSLVVFV
QICYFYCTPCQNVMDQGLEIAIQVYSINWYELEDNSLKMLLKMIIMRGSEPIDFSAAGFVQINLAALVMVFKT
SLSFYSFLVAIDT

>XP_019870448.1

MHGYHKNFFETNFLSFKMFGIWVPNNLSVVQRSLYDLYIVTLLAIAGLYFISEIIVIKDTRKNLFDLLSNIGMLV
THVVGVSKFATMFLKREKIEGDDGEIAMRQIQVRVSRMRQASSQDYINCNDYVPYYGYVPFTLDTIGKCHFL
FFFMDVPLVIFALHIAAFDSLFGAISNCLKCQLLIVSEAFTSIRIRVLTKLKLPETFQTFRDEEHPKLEQAMYDE
MKHCTQHLETLISVHQDIEEMFTYVILAQSLVSLVVYATGLYVGSYIPLMSPEFFPRMLYFLAILMQFATYCWY
GSEIIYAGSNVGPSIFNCDWTSASQRFKKAMILNIIRMRYGLYFSIGKFTPLTVATLIAVLRGSFSYFAVFKSM

>XP_019871599.1

MELVENSIIPDLDLREMISYSLNIIMFTGFLRPEKRSKYDIGYKASIVFFVGLFFILYMSTEFANIVVVFGDVEEMT
QASFLFLTHFVQFFKFFSITQYNDLLWDMILRLNMKAFEPRTLNQRQMLTERISLSKKIAKCFVYLGIATCGL
WAVFPFVDSGKTEVELPLSGWFPFRTDYSPVFELIYLYQVCGSTFNAVINISLDTLIAMLIFFVSCQLEILNDSLA
KAKEICERRVKENNGRRNFNEEIEVYLRELVNHHRYILNFADDFTKIFTGCIMAQFVVSMLIICVTLFKLTLVP
VASLEFLSMMIYQICMLMEVFLWCYFGNEVIINSMEITTSAYTCDWTNFPMQFRRNLLFFMTRSQFSLKLYAG
GFFVLSLPTFLGILKSSWSYFAVLNHMHSQ

>XP_019870731.1

MTPLFYISHVTLFYDLVYRIKNGIPLDVELCATMCSALVANYMLIVFLFNIDSVMSICHHLSSTEEFPLPPDFIPT
RDRVEKFAHIYRIYICVSSVIYSSSKAILTNPCQITKAALNVERDCALYLPTFFPFKIEYFPVYQLVFLVQGCSLL
HILIGASYVALLSYEIISYLGNRMNHLCLILETISTEKDEKLQKETIKFCVRYHEFIIRISTDTSSLYNKIFAIHAIAA
ALVFAGLSKQALNDLNAALHLIGWIGCIFLVCHTGQNLIHVSENLGEKIMFMCKWYKLNSSLQKDLQLILLR
SQRPLTLPAGPFATLNYELFIKVIKTAYSYFTFIANT

>XP_019871965.1

MGNKNHLNFLKKVRLVVCGFVDPSQLKYKNKLVKGFYRVYSLIATPLYLTNLTLMIIEMFKESDNPQIMGET
LSYSTLYVVMYVSLLYIESKSVQDLYKAIDEKEEALLKTANTSIKQIYEKHTEIKTTWWGRKYSESAASFWFPF
NPDNYYWLLILDQVCYLSVALFYMMYSKLAVIAFSTYILAHIKVLQHFVVNIEHYAKYVMENEGIEDIHDAR
YVVIKHCVRTHNEIIEYIDLVRQNTSTFILLDFMLTSLQLGSILISLMTEGLNPRLVDKLVLWYYFVLNIHWMW

VTYYYGNEIMVESVNISVLAYTELEWHKYDLKAQQGLILMMAKSQFPLSLSIGPLADLSLSAFLTVIRATFSYM
TFMKTIYE

>XP_019870045.1

MPPIDLTTIMSPNVFIFKLYGFWKPDTILSFLLKVLVLCILLTFWVTQLVFLFFNLNDLRLVTSVSYAVLTFSMN
LVKLAFMYKNIHKILEMMALLKTEPFRTKCREHEDLAQSTKKYYSSVFYFCLVLGLTVDLFWGIYPVTSVESII
PFRAWFPFNYEDTVYFNVIYALQTAAATMNTVICLNLDCFSSSMLVQAGLQCDMMSITLKNLDKFMVNED
GELLEAKVKDENKQEYSNRLLKNLIYCIKHHVEIKKVLKFFDSICESPWLDCDVKFKKTIVMFLMGARKPIVV
YVGGLFTMSLPVYVTILRSSYSYFTLLRNVYN

>XP_019874865.1

MLQIKLLLAEDLEMVAENLNYTIMYTVMAIATRYLNNPNCRSLLLDIEKFEKTISQNGDTDKRVLEISNKYSK
YNNRINRFMFCFTLSTCILLSYSYSHLPFRPKDRLKFIFPEWYFMDVEKYYWVIYIDNTLFVVVCLGPMFVYVR
FASLTFTTYINARLEMLQYFIKIVEPLSVKLSEKYDIPIQDARYCILRSCLKQHIYIINYVTVVKEYTNMFVLLDW
LGTSVQFGSMWLRVLLSFQEDDGFDKFMTGYHFVVIYTFMVFNYYYAQKVENESINISQHAYNLDWFEYDG
RTKMLVQMIIMRAQKPLNLRIGPFAFMRLFSLVDILKSSFSYFVFMHTLYDLYEKM

>XP_019877353.1

MGKVTVQEKIAPNLFSSYFDMTMRQVYLSGIWVKHYMKSKHKLFWAIHSPIVVACSIFLTYQEIYLICTSRTLN
EVVKNLRDHLNHVAGTAKLIAFVLMRHKFYECMRLLRTYDFYYEQVAGFDCKEIIRKQLRACFDVIFTTMCS
CVTAHIKILQGAFRTIRQRCLLRIKINEEEFLNDPPCLQEETIKEMRRVIIHLQAVISVCDRVEYVYNKQALVQT
SISLLEICFCLYLSSTRDKDIAMNGFYLMATMFQLMLYCWHGNELTYASSGLPYAVYDGDWLSTNKAFKQY
MIITMSRMNRPLYVTIGKITPMSFSTFLTIIRASYSVFTFLKQKENMNMKTNGNIDY

>XP_019872736.1

MKVQKQRVFLSTCVYVLHLMNLWPKENQYVYSKWRFIKDISLIVSIMPCAIPILADFSLQLYDGITDLTAIIDN
LIALTCIVEMIYMIICFINKRRLIKKLVTSICDFEKYGQSQELLQTDRKANLYSKMFLWYGILGNFVYMLMPQL
NVKHCVENRSQQLIDQGIPCGLVVRCRFPFKFDYTPVFEIVFVHQIYTCSMVTLVVLVLTMLLCGFLMHLEH
QLQHLKSYIERLDLVPCCQYIEQLHFCIKYHCAIIDYSSALNEAFSLMMLLHLSLTSIVISVLGFEVLSVDNLMD
SVRFCLHLLGWLVLLLLICYYGQKTCXXSTSLSLAEKIYSLSWFTRSTNAKLQMKMMIMRSQKPLTLSAAGMG
VMTLPTFLRVLSSAYSFLTLLLK

>XP_019872735.1

MEEETVDHEYLSFAINLLDKLQMWPASSNNKKNFLQKYFNNFMIALSILAVMADIVTAYHDNNLTIEHLME
QLIALSALFSVFYCSICFMSKKHEIKILVQNLTKFEKYTPKSLIQETDKRISFYSKCFIIYGVVGNMFYALSPLLSY
KSCLEERHFNKYGISCGTIVSYVLPFKHNYAPWSQIVFLQQYYTCNLGTVIIMTITMLLSGILMHCNTHLQYLE
SLILKTSTVDRRFIYEHIYYCVKFHTAIIEYTDEINDSFSLMMLLHICGTSFIISVLGFAILIEANYSNGVRFSMHLA
GWFGMLFVICYYGQTLMDKSSHLSIAAYDTLWYEVPFKVRESLCLVLLRSQRPLVLKAAGVNVMSFATFLGV
LSTSYSYFTLFLKIKP

>XP_019879631.1

MEESHLAFFWTVQALAGLKKWKNTTIKYKLYKIFIIGVYFYNLFMLQLQLITARTIEEIMENLSFTTMYTVMTI
GIYFLSKPKFTQLFQDIKDFEETLIKKQGIEKKVIEIADKYSNINNKTNRVIYFTTSIFCVFFIYDYSRLPLRPKNRI
KFIFPEYYFFDEEKYYWFVFIDNCIFLVGCLLPLFVYMRFSSITLITYIIARIKMLQHFIRSVETYAKHLSRTQKQSL
QEARYNILRSCLIQHIYIIEFVNTVKKYTNMFILMDSLASSVQFGSIWLRLLIGFEEDDLFDRVMIGLHFIVIYTF
MMVNYFYAQQIQNESENIAVETYNLRWFDFDSRTKIMVSMMVMKARVPLTLAIGPFSEMKLYSLVQVLKAS
FSYFVFMHTLYDLSLAQ

>XP_019864601.1(PSE)

MRFSSITLITYIIARIKMLQHFIRSVETYAKHLSRTQKQSLQEARYNILRSCLIQHIYIIEFVNTVKKYTNMFILMD
SLASSVQFGSIWLRLLIGFEEDDLFDRVMIGLHFIVIYTFMMVNYFYAQQIQNESENIAVETYNLRWFDFDSRT
KIMVSMMVMKARVPLTLAIGPFSEMKLYSLVQVLKASFSYFVFMHTLYDLSLAQ

>XP_019872257.1

MEEEINFLKLLKKLMILSGIWRLPLKTKSLQSFYDKYSILFQLYFYAFVLSSMAIAVGVVWKYSIDTVVECLSISI
LCSIVSMKTVVCQTESIKRLLRYILAKEDELKRSTDEESKRICIEHQNVAYFMTKLITSSTATVVQVASIFFQLVT
LKLSLLNLVFLTTYFGLTSLQIFSSAWFANEIKEQSLGVADAICEHNWHEQSLSIQKTLMLMMTRAQKPLTLT
VGPFSTMSNNTPITIMKASYSYVTLMTSKRE

>XP_019869900.1

MSYHETINCWFDSLKWIGMSPAKPRNYWAFIIILTSGMFVIGLAFADFFFNPDTNYMDGLETMVIFSHVTHTL
SKTGAVFVFSQGFKQLTEDLKHFWSFEKEELKLKFGNDQDNQILKMRLVKLIKHHIFLLNFTERLNRIMSLPL
FFQMCSTVSTLCIEMYLMKNNVDLGQGMIAGLLTMQIFIQICYFFCKPCQDVMDESSEVAMEVYSTNWYELE
DPFLKMFLRFIIMRSNKPVDFSGAGFVEINMAAVLMVFKATLSFHSFMVAMESK

>XP_019873019.1

MVYVYVPEILGRYFFLVLENVLIPLITLTVVSFDYLILTFMQLTIIQFQILHSEISKLYFGQDQHQSNLRSQFTKIIK
HHIFLLEFADKLKQIISLPFLFQYFTTVMAICMELYVSRKNFDLGYIILAMFYVVAILFQITLYCKSCQDVMDLS
ADVSNQIYTTKWYESQDNSLKMSIKFMIMRSDRSIAFSGSGFVPINLEALVMVFKTIMSFNSFLTAMDT

>XP_019874691.1

MMEQINCLKMLKRLMMISGIWRLPLETKSLQGLYDKYSIVFQCYFYLFVLSLGVATIIVWNISMDMVVENLG
VLILCVIMIIKIGICQTDTIKSLLRHVFKKEEELKASDDEESKQIYIRYQNVAFVITRIIVIHTISVGVSLLLTKYLFY
WNMEKNQSYPNVTLYRKKPLPYITWLPFDSDQHFITAFVLHSISAFMGVNYNSVTAIFFLSVMIFICGQLAILQ
HKIRILDTTLHNSNWKNVTNEYSEKFNNAVKMILLLDFTLNSLQVASLIFQLITMELNVVMIFLVIYLTLMVL
QIFLLSWQANEVKEHSLRIADAIYDHAWHEQIISIRKTLLMMMMRSQKPLTLTIGPLYAITNSTSLTSIKAAYS
YVTLMIRKRY

>XP_019873848.1

MLKRVGLSAAQDFFFLNRWIMKFAGLWLPDSQNPNVQTAYKIYAVFVFVFVNLYFTATEFVSLLYTYKDLN
EFIKNINFALTHLMGAVKVVFWYFNGYKMQRIIKVLEEADYYEESGQFKPKELYDDFRILGMKTTIVFFLFAH
STLSASYLPPTFIALKYLFADSWEGLPENLPYYCWMPFAYDTPKLYLLALGYQAGPMFSYAYSIVGMDTLIMN
ILNFISYHLSLIQRAFLTIAERTLEDPCRSKLTKEEDEMINREAVKICRHVQVIRRICMELEATQRYLTFCQGMA
TLFILCTCLYLVSTTPVFSEEFYAEVIYFVAMGFQVFLYCWFGNEVTLKAQEVPMYIWRSNWYATNKKFKET
MVYTMLIMKRSIYFSFGNFVPLTLQTFMNILKTSYSFFAVINNSEK

>XP_019873018.1

MIQVIMVGHSAYNNSSLDILTVKLITIGSASLDALCVSLSKIDYKDEAKTSLIKSLKFYNEILDYVDLIEKTFSMG
LFVQFLATVFVLCFTSFQLSLVDIGTNQSYLYISYMSCMLCQISMYCWYGHEIMFQSTGLSASCYLANWYESD
VTLRKMIILFMERTKKPVILTAGKFFTLTLTTLKELLKNSYSYFAVLNRLYK

>XP_019881951.1

MTNFALNKHQMCLVDDILNNPIFKTVSLGETEIIVNSMVSSKYLSVVFRFLCVGVVLFYGIYPVIDISSENMYPL
DLWYPFDPNSYHSTIILFEVSLIALGAWTNSNIDILTIKLISLGTAQIEILKKKLKSLVNNMEDVVTDEIVNTRLK
HCIEHYSLILRYVKTVQDLFSKGIFVQFASSVVVICMTGFQFIVVPLQSMQFILVLLYFQTMMCQVIMYCWYG
HTIMESSSGISMACYMSEWNRCSPKTCKMLMIFMERAKRPIRFYAGNYFVLNLATLMAILRSSYSYFAVLQRL
YKKFVNTVELAFADGVLVQFAATVMVICTTLFQMIVSRYIQAIEEVFSYGVLVQFLASCLVICFTGFQLVVTKP

QSLQFFILLLYLMILLTQVAIYCWYGNNVSIKQQELLVATLLSTWYESEMSVRKSLIILLERCKMPVSLTAGKFF
TLSLTTLLGILKSSYSYLAVLQQAYSDK

>XP_019869901.1

MYHQTINFWFTLLRCIGMSPDRPKNYFTFIFIVTLGNIIFGLVLADFFYNPDASYMDAIESIVLFSHMLSKTIAIF
MFSKGIRRLSENLKNFWSFEDHEHLREDCYNKLISLHKGAKMYLSYYVIGCSSILMLPLVTSRTLPMYVYVPE
ALGSNFIWALETFTVPLLDLNVFSFDYLTFTFLQLTIMQFQLLNSSISQLKFGKGQDNEKLKMQLAKIIRHHIFL
LEFVRTLKQVTSMPLLVQLFNTVSAFCIELYLMRNHIDMGHALVGILHSVVIFIQICYFYCGPCQDVMDQGLK
IADTIYSINWCQLDDNKLKMYLKLIILMSNKEIIFSAAGFFRINMATLVTVFKTSLSFYSFLLAMETKEGRVKTL
AF

>XP_019867175.1

MSSEEHFNFREIVSLNIKALHIFGYFRPDTDSKLQRMLYEIYSIFAVGLLLFVLTLSEVANMYVIFGDFEQMIAD
SFLSMSHWIHYVKLYVLRGRGDVIWKLIHDIERREYLPKSPTQLRILETNVRNSKIIFKTFFSMCIMCGICFTLIPF
LGAGPGTWAKRNCLMVDASSQLEMLNDSVKNLKQFAMEKAAKNGRNLEEEMNLCLIECVKQHQYILDYT
KTLNKVFTECIFGQFLFSVIIICFTLFKIALSNELTNSAYFTHWAGCSKRFGKNLVYFMTRSQLTLKMFAGGFFT
LSLQTFVTILRSSWSYFAVLNHT

>XP_019879945.1

MYLSYYVIGCSSILMLPLVTSRTLPMYVYVPEALGSNFIWALETFTVPLLDLNVFSFDYLTFTFLQLTIMQFQLL
NSSISQLKFGKGQDNEKLKMQLAKIIRHHIFLLEFVRTLKQVTSMPLLVQLFNTVSAFCIELYLMRNHIDMGH
ALVGILHSVVIFIQICYFYCGPCQDVMDQGLKIADTIYSINWCQLDDNKLKMYLKLIILMSNKEIIFSAAGFFRI
NMATLVTVFKTSLSFYSFLLAMETKRRTCKDAGFLS

>XP_019869899.1

MSYNQTLNFWFRCLEYIGMSSAKSQKSVGFFVLMSAGLFILGFAIADLFYNSKVKYTLETLVMLSNMLTKTA
AVFYYSTDFRQLSEELEHFWSFEHEKDLKDKFHKELANIHKAAKLYFYYTALLHSAVNKLNVEQGDDQTHL
GPQLLKIIKHHILLLDFADRLKRTMSIQLLIQFFITMTAICIELYVTRKKIDFGHSGIGLLHLLGILFEITLYCKPSQ
DVMDESENLAMQVYSVNWYEWEDNSMKMSLKFLIMRSQRSINFNAGGFVQINMAALVMIFKMSMSFNSFL
VAMDTKKD

>XP_019870159.1

MENIGMDITSILGFTGCLFFTCNIIKSIAKFIKVLRVLSDLKTHGIPNGFDEQNKKLNFYSKMFAMYMYIAMTII
TFSSTVNMGYCFDKPACGMLIPPVMPFDIDHYPGKLFYFILKLVALILIYHTGGILSFAVMEVLEHIIFRLDDVK
NKFVQALNPENPNRDKDLKEAIEYHNFVINVSQEYNEVIKPCMLVHVILTGVMLGATFWFTFVKFTLDSLVI
CLGYFTAMGCVSIGGQRLMEASESVADVVCTTCWYGVELKLQKALILVIARSRRPLYISAGTLGIVNFTLIIGV
GYL

>XP_019867295.1

MIFFITRVITVHTAIVGICHFLTKYLTYLELEKNQYPNVTMYRRKPLPYTMWLPFNSDEHYFGAFFLQSIAGFM
GCTYTAVTVIFYLTGMIFICSQLSILQHKIKNLATSTHQSEVEVKKKLFTLIIDHQTIIEYSEQFNNDINVILLIDFT
LNSIQVASIFFQLITVSIVAKVLHYNCFKKLQIDLPIVMAFVLNYLALIVVQIFFFGWFANEIKEQVTLTLTYFLN
L

>XP_019877428.1

MKNYLQYFFSMINVTCMIVQFMANIESMNSIRLDLSNMEFTMPSDFAKTRDTANRHSTFYGVSMYMFCISYA
AANIFGENPCGNQIKGSDISCGIFLPSIMPFNINFFPVFQLIFLAQFLSTVHLLVGGATVMFMLYEIVLYIGNRM
EHLCIILETIPNQRDEKVQKDTMKFCVQYHNFILSISKKTSRLFNKIFCFHIIFTGLVIAALINQALENINARFHL

FGWLIALFLMCHSGQQITYISENLGQRIMFMCKWYKLNTSLQKDLRFILLCSQEPLVFPAGPFADLSYDLFIHK
GNKLYSIKMENIMFYFTDY

>XP_019870923.1

MSRVSKVYESSMKMLLATGLWPQQDATFLDNSKAFLSYSNFFLMMTLLGIELYDSRTSIVRMSEILFVFSTFTA
YCVKMFSFRYKKTAFLNLIDSMQELDNYPKGFELESHMIGAENVQRRLATTYRWICASCMSCFALFPLLDED
KTFPLPFPYFHDGLMRIVVYVFMISGLGTCAWNNSCLDLLPTSFMAIAAASLDILNDQLANTRSSNVDERKVI
EHLLYCAERYDKIIK

>XP_019876501.1

MEEEINFLKLLKKLMILSGIWRLPLKTKSLQSFYDKYSILFQLYFYAFVLSMAIAVGVVWKYSIDTVVECLSISIL
CSIVSMKTVVCQTESIKRLLRYILAKEDELKRSTDEESKRICIEHQNVAYFMTKLIITSSTATVGTFLLMTKFLLY
WDMERHQTYPNVTMYRKKPLPYITWLPFNSDDHYISAFSLHSIAAFIGTVFNSVTAIFYLTCMIFICCRLRILQ
HRIKTMGTSMHQPELVVKAELSSLIVDHQTIIEYTEQFNNDINLILLVDFTLNSVQVASIFFQLVTVKLQVKLR

>XP_019877367.1

MLKMVWYDFWPADMVGKNIESELKQNTTQVVFCYLSVYVPAFMYHFGNFTIALIRNKLGFEAAFPFNSEISP
IYEILYVSQVWLNIYASVTSILGHDFLLLGLTGNVVAQVTLLEEALKQLGTGNEIELNEKIRVITKKLYYTNDSN
ESEEIKLMISCFNHHRMLLSKWYKVLDPRFRKLMLMSMQGSNKQITFTTFKIVTLNYGTFISVIKLTFSIYNLLS
SVQSKKY

>XP_019867957.1

MHLSSYGTVNDTRTCVEIFMFSTYIPFKPTSYLECHFFFALVDCQLAIFSVWISAHDTTYITLLHCLKTQFYILGE
AIQTIRERTLDKLGLPLDFEVFRDEDHLKLEKEMYKELRRCTKHLMLLIRVYNKLENVFCYVILGQSMVTLMV
LASSLYASSLIPFGSPVFFSQMEYLVAVLCQFAFYCWFGNEIVLAGEFVQDSMFQCD

>XP_019879871.1

MLKRVGLSAAQDFFFLNRWIMKFAGLWLPDSQNPNVQTAYKIYAVFVFVFVNLYFTATEFVSLLYTYKDLN
EFIKNINFALTHLMGAVKVVFWYFNGYKMQRIIKVLEEADYYEESGQFKPKELYDDFRILGMKTTIVFFLFAH
STLSASYLPPTFIALKYLFADSWEGLPENLPYYCWMPFAYDTPKLYLLALGYQAGPMFSYAYSIVGMDTLIMN
ILNFISYHLSLIQRAFLTIAERTLEDPCRSKLTKEEDEMINREAVKICRHVQVIR

>TcOr1_ORco

MMKFKVTGLVADLMPNIRLIQASGHFMLNYHADNSGALHTLRLGYCCMHLVFVLVQTFSCNFVNLVLERG
DVNDLAANTITVLFFTHCVTKFVYFAVRSKLFYRTLGIWNQPNSHPLFVESNNRYHGIALKKMRRLLYIIIIWT
SFSAIAWTGITFVGDSVHNIKDPENENLTITEPIPRLLVKAWYPWDAMSGMPYYITLVFQVYYVFFSLAHANL
LDSLFCSWLIFACEQLQHLKEIMKPLMELSATLDTYVPKSADLFRAPSATSQDQLIENGTNPAKKNEDLKGVY
STRQELGGHFRGGALQNFGSGGVGPNGLTKKQELMVRSAIKYWVERHKHVVRLVTAIGDAYGVALLLHML
TSTIMLTLLAYQATKITGVDKYAATVLGYLLFALAQVFHFCIFGNRLIEESSSVMEAAYSCHWYDGSEEAKTF
VQIVCQQCQKAMSISGAKFFTISLDLFASVLGAVVTYFMVLVQLK

>TcOr2PSE

FDWSTTIGINLFLLKCVGLWPEAEFYKPNFYTFYAIFNAIFIIGGHNLSQLILTYFVYSDLEALANTIFVLTTNIM
AAAKMYFFVRNLKMIKQMLAVLNTPQFLPKSDTQVDLVKPSLKLWKLSYTVFSVIVAMNVFLWSIAPFFNPE
QRFPFVAWYPFQTSTTLNYCIVYLYQVVCIWVITIANMNLDTVTMALMVYIGTQCDILCDDLSNLYGNSDYF
HKHLISCIKHHKKIVSYARKSNNIFNMIILGQFATSTMVLALTMFQLSLVNPLSGIAAIHLNYILGITTEILLYCY

YGNEVEVKSSRMSYSIYKSDWYEQPINIRRTILILCERCKRPIKFTAINLFALSLSTFMTIIRSAYSYFALLYNVNN
RZ

>TcOr3

MKLSSVTTCLFSSDFHTRMNFDWKDTIKLNFLMMKIVGLWPKEKYKINFYTLYTLISVNLFICGHVIFHTVAV
FVVGRDLKHLIGALYMSLTETLLLVKICYFIKNSRLVKSLLTSLDGDIFQPKNEKQLELTNPSLIFWKKVHKSFA
ILVANTVFLFVSLPILSKSTKLYRLPLEAWYPYNTQKSPNYEITYLYQFISTLFRGMASVSMDTFIAALNMYIGV
QCDILCDNLRNLNETNFMENLSLCIKHHKAIVSFARECNKFYNGIVLGQFFSTSIALGLAMFLLSLVTPLSTES
NTLLFYLGATTSEIFLYCWFGNEVDVKSSKIPYSAFESDWTGAPIEAKKNLLIFILRTQKPIKMSAINLFSLSLETF
TTILRTSWSYFAVLRQVNGQAZ

>TcOr4PAR

MYSENKFDFVLTIKPNILFLKIVGLWPVDNNDVYRIYTLIVTVFFMGVFDFTRIMNIFFVYTDLKVLTATIYLTV
TDITVLVKTCLFMSNIKTLKRLIVTINCDVFQPKTDHQKQLVQSGLKAWKVSYMVFWSLVFCCLVMWTVSPII
QATPAGRKPLPLPAWYPYNTDITPFYEITYVCQVISMWFLATANMNMDSLIAALMIYVGAQCDILSDNLKK
MKTFSKIKEEQAKFNQTLIDRIEHHKKILQFAYDCNASYNFIILAQFFTSSLAIALSMFQLTLVDPLSMESFPLLS
YAFGMALQIFLYCWFGNEVEAK

>TcOr5PAR

MIFFAWKSVIKTNLVILKFMGLWPRETKYRRNFYSLYAVLLTVMVASDNLFQTINLVLIHTNFSSLMGTVFVL
CTNWCASIKSFCFIYKVNLIKILLQDLDCEEFQPKNDTQMNLSRSILHLWKIVFYAMTSGVTATVLALAVTPL
LTGDLAQYELPFWAWYPYKTRTSPLFEIMYLYQVICVFCIAIISLNIDLLNMALMMYVGIQCDLLCYDLKKLN
NSKKDFNINLIKCIKHHKKIDSFAKHSNVFFNEILLTQFLTSTASLGLALFQLSLVDPLSLDGYTIVFYVLCIITEI
FLYCFSGNEVECK

>TcOr6

MNSFNWQESIKTNLKALRLVGLWPKSDFYKFDLYTFCTSLTVVVIVCGHNLSQIVYILQVYSDLKALTATIFV
ASINFLGAVKMYFFIKHIKTVKILFKMLKTYQFKPKNIHQTQLIKPFLNLWKILYVGYSINVYLIVAMWSLLPV
LNGWTWQKKLPFPARYPLDVTKSPYYELAYVYQFICIWYITVANLNLDTIIIALMMYTSCQCDLLCDDLKNL
TETRFFDKKLIECIKHHKAILVFAEKSNSLFNMIVLGQIATSTVVLALTMFQLSMVSPLSSEGLNHLFYIGGIIM
QILLYCWFGNEVEAKSSNILYAIYESTWSEASKNSKKNLIIFSIRCQRPIKATAVKLFALSLRTFITIVRSGWSYFA
VLYNVGSEZ

>TcOr7

MNKLQKFDWKATIRPNIAFLHYLGIWPEGEEYYKLNFYTLKTILYIIILVISTIVFQVINIFFTLDDLTSLTANIYV
LLTEILYFIKLCFLVKNMPALKLLMKTLDHKLFQPKANQIVIIQPLLNFWKLIFLAFVITCSFTVLFWAIFPILDS
SEEEKRLPLLAWYPYDTKISPNYELTYLHQVASYIYICYSHLNIDTFITALNTYIQCQFDILCDNLKNIKSDTKN
VDTKLAKCIKHHLLILMFANTSNEFFSWIIFFQFTSSAAITGMTLFQLTVVKPFTTEFYNFMAYVTAEVVQIFM
YCWFGNEVQVKSSNIPYAAFGSDWTEFSPNKQKSLLFLITRSQKSVKMSAFNVFDLTTDSFILKSAWSYFALL
NQVNSZ

>TcOr8PAR

MRSFDWKKTIKINIITLQAVGFWPKGDDSFKFNLYGLWTAISFTCIIFCHNFFQAVSIYFILNDLKAVTAVIFLN
LSELLGILKSYYLIKNMPKLKQLMITLNKPLFQPKNERQIKLIEPNLKFWKQMYNLYWVMSSGALFFWATFPI
FDNSIKDHRLPFIAWFPFDTTVSPFYEIAYVHQVVGIIFVAISTVGVDTLIAALSMYIGAQIDLLCDNLRHLTGP
NFNNELLNCIHHHKTILRFANNSNEFFNWIAFFQFFISATSIGITLFQMTVVTPFTSEYFSLISFELAIVVEIFMYC
WFGNEIEEKSKNIPYAAFESNWLEASKESKKNMIIFMIRCQ

>TcOr9

MSNFSWKAAVETNITTLKILGLWPKGDETYKLNFYTLYAVFGVIGLLCAHSFVQIFNIYFIVDDLEAFTSSIFVT
LSCLGTVAKTYYLLQNMQMLKELFISINKDIFQPKNNKQILLVEPSIKFWQRFYLIFRVLCYCTTFFWSSYPILD
KWTKDHRLPFLAWYPYDSTKSPFYELTYIHQVVSIWYLVSASLNIDMLIAALNMFVGAQCDLLCDNLRNIGQ
NSKEIGKNLVKCIEHHREILRFAVQSNVFFNLIVLLQFFTSAVSVGFTMFLLTIVAPFSSQFYSFICYGSSIITEMFI
YCWFGNEVEIKSNAIPYAPFESEWIGVPIEVQKNLIIFTIRAQRPLKMSALNLFYLSLDTFKAILKTSWSYFTLLN
QANSNZ

>TcOr10

MEDFSWEATLSQNINFLKVCGLWPPGDEAYKFNLYGIYAGFCVLGFLCVHTGTQTFNVYFILDDLEAFTSSIF
VTFSCVACVFKTYYLLKNMKLLKVLFININKEIFQPKNKEQQLLIQPSILFWKRFYLVFRILCYNTCFFWCAYPI
LDKRIKQHKLPFLAWYPFDSSVSPLYEITYFYQAVAIWYIVIISFNIDMLIGALNMFVGAQCDILCDNLRNLGK
SDINELNPDLIKCIQHHKAILSFVSKLNIFFNWIVLLQFFSSAVSVGFTMFELTLVAPFSGQFYSFICYGSAITTEM
FIYCWFGNEIEIKSSKIPYAAFECNWVGTPLGVQKSLIIFTIRTQRPMQVSALNLFYLSLDTFKTVLRTSWSYFTV
LNQVHSZ

>TcOr11

MEKYDWMQAIKTNILILKIVGLWPDSEDYKFDFYALHASVWLSTLLVASTFFQGINIIFILDDVKALTGTAYVL
LTEILAVIKTYFVVKNMKMLKHLMQSLNNNKLFQPRSHEQIKLIQPSLKFWKLLYNLFHSLVGGATLFWILFP
IVDKKEKRLPFLGWYIVDTKVSPYYEIVYGFQFCSCCYMSALIINIDTLIAALNVYIGNQIDILCNNLRNLKAGC
SIERDLITCIKHHQEILNFVQYANKFYRWIILLQFFVSAVSIGITMFQLTIVVPLSSEFYSFLFYANSIISQIFMYCW
FGNEVQTKSNKIPQAIFESGWTDFPLKTKKDLVFLLMKTREPIKVSAFNLFSLSLDTFMRILRTSWSYFALLNQ
VTZ

>TcOr12

MQKFDWRSMIKMNIVVLRCVGLWPSGEESYKPGVYTIYASTVLTLFLLGHIFFQAVNVYFIRNNLSAVTGTIYI
LLIEILLVFKVYYLVKNMTVLKQLLKMLETEMFQPRNSTQINEIQADMKFWQMLIRFLWVSVMCSNLFWAIY
PLVDNAGKEKRFPFLAWYPYDAQKSPYYEITYVYQTISVNYMSSIHVSVDALAGALNVYNGNQFDILCDNLR
NLHRLTKNGTIDAGRNFRYCLKHHKHILDFAKKCNNYLNWILFMQFFVSTISIGITMFQLTVVRPFSNEFYSL
FTYISAIIGQIFMYCWYGNEVEVKSSKIFYATFESDWIEFSEEVKKELIFFVMRTQKPVKLSALNLFYLNLDSFMR
ILKTSWSYFALLHQISNRNSZ

>TcOr13

MDYYDWKSTIKSNLFMLRLIGLWPKGEEGYKVDFYMLYASFLLLTFVLTHIFFQTINIYFIRTNLAAVTGTIYVL
LVEMLLLSKVYYLITNMTILKQLLHILDTEMFQPKNSAQILEIESNIWAWKLIYKSFTYSCFGVNMFWAMYPL
FDETDRRLPFIAWYPYNTRITPFYEITYAHQIISVSFITIIHVNVDALAAALNVFNGSQFDILEDNLRNLHKLKK
NGVVDVRQNLTYCVRHHKHILSFADKCNNYLNWILFLQFFISGVAIGMTMFQLTLVVSFSTEFYAFLTYGIAI
TMQVFMYTWFGNEVKFKSSKVSYALFECEWIDFSQKVKKDLIFFTIRLQKPVKLSAFNLFYLSLQTFMKILKTS
WSYFALLHQVNTRZ

>TcOr14

MLLKWSSVIEFNLFLLKWIGLWPGEDYQLNMYSFYGFSVIILILCGHTLSTGLTLILDSGDIDTFTETMFILNIEF
MTAWKALNFALNRKKFMQLLDAIDKTTFQPRNGKQVTLVLRNIDGWKVMFKMFGISLGLSFIFTGLLPIFSK
TYKDRKLPMEAWYPFDSTKSPFYQLCYVYQMAAVAVAVMVILNVDTLVAAMNICIGLQCDLLCDNLRNLH
TNTSKSMNQKLIECIKHHQNIISFAEKFRQAFNWSIFLQFFVSTTSLGIVMFKITRFSLYVSEYYRFISYACSVLV
QVFIYCWFGNEVIVKSSKIPYALFESDWTQDSLEMKKNMIVFILRTQKTLKITVCHVFDLSLPTFLTILKTGWSY
FAFMNRVTSPHZ

>TcOr15

MLVKWSSVIKINIFLLKWVGLWPGEKYQLNVYSFYAFTVIILILCGQTLSTGLTLILGSGDVDTFTETLFVVNIEF
MTAWKALNFALNRKKFIQLLNAIDKPMFQPRNDKQVTLVLRNIDGWRVMFKMFAISLALSLIFTGLLPIFTK
TYKQRKFPYEAWYPFDSSKFPIYQLCYMYQMASASTLVVVILNVDTLVAAMNICIGLQCDLLCDNLRNLHFD
TSKSMNQKLIECIKHHKSIIRFAEKFRQAFNWSIFLQFFISSTSLAIVMFKISRTTNYGSEYYRFISFACSVLVQVFI
YCWFGNEVIVKSEKIPYALFECDWTPEPLEVKRSMIIFIIRTQRILKITVSYMFDLSLPTFLSILKTGWSYFAFMN
QVTEVNTSKZ

>TcOr16

MDKYDWRSHIKINILVLRFLGLWPKNTYHGFYIFHLIFMMGTFLLGHLFFQAANIYFIRTNLEAVTGTIYVLLV
ESLVVFKVYHLVKNMAMFKQLLEILDTEMFQPKNKKQIVAIDETIHVWKTIYKSFLYTCFGTNAFWAIYPLLD
KSEGGKRLPFLAWYPYNTTITPLYEITYVYQIVSVSFITTVHVNVDVLVAALNIFNGSQFEILCDNLKNLHNGP
VKENLIECIKHHKEILKFAERCNNFLNWILLVQFFIFAVSIGITMFQLTLVIPFSTEFYSLLTYGMAIILQIYMYC
WFGNEVEIKSNKIPYAAFECNWVDFSPEVKKNLIFFIMRAQKPVKLSALNLFYLTLDTFMMIIKTSWSYFALL
HQVSSRKZ

>TcOr17

MDDFNWISTVKTNLLLLHIGGIWPRGDGTHKLNLYTIYAIFITFTFTTYHCFSQIINFFFVDDLQALTESIFISLIQ
SMALVKAFYILKNMRILKNILKNLETNKMLQPRNLKQIKMVQPSLTQWRLLSQMFWISAVFAMCLFGAFPIV
ESTYKEFRLPYLAWYPFDTKSSPFYEIMYLHQFVSSYTIAIVDIGADTLIAALNVFVATQCEILCDNIRNINGSVE
EMDSKWKECFTHHKEILKVARHCQKFFNWIVLMQFCASVICIGLTMFQLTLVVSFSSEFFSSLFYFGAITVQIF
MYCWFGNEVELKSSKILYATFEANWVEAPHQVKKNILIFAIRCQNPIKMSSLNVFYLTLETFMAIFRTSWSYF
AVLRQIQNRISEEZ

>TcOr18PSE

MEEFDWISTIETNLLLLHVGGMWPKGEGTHKFNLYSIYAIVVTLTFTFYHCFAQVLNLFFVQXILQAFYILMN
MRALKGTLNNLQKNEMFQPKNLKQIKMVQPSLNEWTTLFRMFWFSGFAAMFLLSLFPLVDGTYKEFRLPFL
AWYPYDTKSSPFYELMYLHQVISTYTVGVVDISADTLIAALNMYVGTQCDILCDNIRDIDGPVQEMDAKWK
KCFTHHKEILKFAEHCQKFFNWIVLTQFCASVISIGLSMFQLTLVVPLSSEFFMFIFYLGAITVEIFMYCWFGNE
VELKSSNILYATFEVNWVDAPQEVKKSILIFAIRCQNPIKMSSLNLFYLTLETFKAILRTSWSYFAVLRQVNARE
Z

>TcOr20

MNSFNWQESIKTNLKALRLVGLWPKSDFYKFDLYTFCTSLTVGVIVCGHNLSQIVYILQVYSDLKALTATIFV
ASINFLGAVKMYFFIKHIKTVKILFKMLKTYQFKPKNIHQTQLIKPFLNLWKILYVGYSINVYLIVAMWSLLPV
LNGWTWQKKLPFPARYPFDVTKSPYYELAYVYQFICIWYITVANLNLDTINIALMMYTSCQCDLLCDDLKNL
TETRFFHKKLIECIKHHKAILVFAEKSNGLFNMIVLSQIATSTVVLALTMFQLSMVSPLSSEGLNHLFYIGGIIM
QILLYCWFGNEVEAKSSNILYAIYESTWFEASKNSKKNLLIFSIRCQRPIKATAVKLFALSLRTFITIVRSGWSYF
AVLYNVGSKZ

>TcOr21PAR

MTDFKLTIKTNVNFLKWLGLWPKNNYNCSLYTFYSFILNAIIASDNIGQTANLFCIYVDFGSLMGTIFVLSTN
WCASIKAFCFIRNTRYIKTLMKNIDCAKFQPKNCHQMKIVLSTLKFWKMVYYGFQILVGVTIMLITTYPIIDGS
IRKLELPFWAWYPYNIREAPLFQIVYMYQFFCVWCIAFLSMNIDMSNTSLMMFVKVQCQLLCNNLRNLKNY
DNCFNAKLIECIKHHQQILSFSNIANSFANKILLTQFFTSTASLGLALFQLSLVEPLTVDSYIIVFYIIAIIDEIFLYC
WFGNEVEVT

>TcOr22PSE

MIFFAWKSVIKTNLVILKFMGLWPRETKYRRNFYSLYAILLTVMVASDNLFQTINLFLIHTNFSSLMGTVFVLC
TNWCASIKSFCFIYKVNLIKILLQDLDCEEFQPKNDTQMNLSRSILHLWKIVFYAMTSGVTATVLALAVTPLLT

GDLAQYELPFWAWYPYKTRTSPLFEIMYLYQVICVFCIAIISINIDLLNMAXQCDLLCYDLKKLNNSKKDFNIN
LIKCIRHHKKIDSFAKHSNVFFNEILLTQFLTSTASLGLALFQLSLVDPFSLDGYTIVFYVLCIITEIFLYCFSGNEV
EYK

>TcOr23

MANFNWTKIIETNFVVLKVIGLWPEKTFFACKLYNIYTHFMVTCLLVIHLLLQTIQLALIIDEFQLFLTALPLLL
QQYHLLIKLFYFMVKFPILRYILHSLNNHQVFQPQNQDQKIQMEDRLSFMKKIYFSFYSMAGVAISFLVAFPIL
DILNGGERQILFVCWFPYDYMTSPFYEFTYFYQSASIIYAGVIVLQIDTLVTLLMTYLGFQCDLLCENLTQVGY
NNSKENNTELEFVKCIKHHQELIKLKNHCVDFSSGLIFVQVATSSIAIGLTLFQMTLNVSTFNVIFLVLYGLSVT
FQMFQYCWFGSEVIHKSDKIAYSAFEMNFVDAPLSVKENLVIFMACTQKPIKMPVLKVTHLSLQTFTKVLRT
AWSYFALLVQVSKZ

>TcOr24

MEEDFDFLSSLQTTLFCLRCVGTWPSNTYKLDAYTLYATASITICLFGHNFFQTVNIFFIFNDLNTLTGVIFVAL
TCLVAILKSLLFIFNMRRLKKLLLVDIRQKLFKPRNRQQVVMVQSRVNFWKKIYFMFTGMGVATMFFWALFP
IMDGTVKEHRLPFLAWYPFSVNKSPFYEITYIYQIVSVFFIVIVNMNSDMLLVALMNILGVQCDLLCDNLKNI
QFRERINEEFLRCVNHHMQILSYASDCNKFFNTIVLAQFFTTVVSLGLTMYQLTIVTPFTSEFYSFIVYGGAVL
MEIFLYCWFGNEVEFKSLNIPFASFGFDWTIGSVGLQKNLIIFIAKSQRPIRMSALNLFHLSLETFVKILRTAYSY
FALLNNVNSLNZ

>TcOr25

MEKFDWRCPIRINLLLLRSVGLWPRGYGVYKRNYYLFYSIFTTITIVGGHNLSQVINIFYVYSDLEALTGTIFVA
TTNILALVKRYVFVRNLPLIKEILQTLNTYQFHPKTRQQLKIIQAPLRRWKLAYLCFSIIVYFNVAMWTLEPLL
DKMIKNRRLPFEAWYPFNSKQSPNYEIAYCYQFICIWNITIANLNLDTLIFAFMMFISAQCEILCDDLRSLDVG
FGPKLIQCIKHHKEILRLAKVTNNIFNFIILGQIATSTAALALTMFQLSLISSINTTALTHFAYMMGMLSEILLYC
WFGNEIEVKSYLIPNAAYESQWMHQDRSVAKNLLILGCRCRKPIKITAINLFTLSLPTFIAILRSAWSYFALLSTI
NGKZ

>TcOr26

MEGIVRNSFTLNLTIMKLMGIYPLENHSRLYKVFGYALYIFSIIPGSVLGFLQLFFKGDITGVGYKDLSSVVVIFL
SPKLCSMVFAADNVKKCIDYLDEGYFTIKNQNQEQIVTECVQICRRNSVIFLGGCTVSFITWSGTLLYRDDIKQ
LPLIAWLPFNSQDYSLLHYVLYCSHGFGVAYVAFAAGTVDPLIPGLICHASGQVQILKDNLQHLDDYIDRSDL
VYEKIKECIDHYGAIINFVFKYERSFSVVILCQLLESAIVIGICCLQISKLEAYDINLIIMGNYLVFLLIQVYFYCYY
GTALVEENNSLINAIYMNRWYEYKKESQKALIILMECSKKPLLITAGRVVDLSLETFTLILKRSYSLLAVLKNYZ

>TcOr27

MLETKQIVFHSFKLNVTVLSLIGLYPPKNYSILYKIYAVILFLAVHTPQLVLGLLHYFLMGDFTSIDYSDFVTVG
MMFYAFKLLPFVTSVTKIQKCINYFDTLGYKILKSEEKIIEDCVGSCRRNTNVFFVGCCLSWMGFVAQVFLRD
EPQQLPLKVWFPYSRDESPVLFYCIYILLIFGPGYSVLACGTIDPMIGGLAYHAAAQLQRLKRNLQYLDEYIKE
KNVGKSKENKRGVIYEEIISCVQRYQEIATFVDLFKDSFSQVVFSQFMGSVFLIGLCCFQIITATEVDINFVITAN
YIWVILFQIFFYCYYGTMLIEENYTLTNAIYLSNWYEYSIPEQKALFMLMERSKKPMIVTAGKILDLSLDTFTMI
LRRSYSLLCCLKZ

>TcOr28

MHSFFNIKVHNKMPVTVTDCFGVNLTVMGLFGMYHRSGNPTIFEKVIAYCMFFFFTIPIPILGSLYFFFQENVD
LEELHNNGFLIAEMVCNLAKYLSFIKNGHRIRKCIHYLELPIFATRRENQERIINASGWICKRNSRVFFVSVLA
ANVFWAGRPFMENKQKFPIEIWLPFDAKANAMAFYLIYLFLVIAVAYASIACAVIDPLIAGLASLASGQVEVL
KDNLENLSKYTREQMLEKGIICNKFDEVFYANVRKCVDHHNAILHFIKEYEECFSSSVFSQFLGSILVICFCCLE

LTKIEPLSFNFYSMTFFATCFSAQIYMYCYYGTVLYDESNSIASAIYMGTWYEYDVKSRKALINLMERSKKPIHL
RAGKVLEMSVETFTMILRRSYSLLAVLKNYZ

>TcOr29

MFNHPNYYTMSNLIEDSFGISLRVLEICFLYTQRKPKFLHRVVSYLLCSNVTFTVPVFGLLYIFLETNITMKRMA
DDAFVVAGMWCYGPKLWPLLTKRTQIEKCIRYFENPQFVTLRENQTEIIKTHSEICKRNSKAFAIFMTTAVLI
WATKPFLFGENNFPVDVWLPFDPKTDLKKYYLLYVFVASGVVYGCVANGAMDPLIAGLVCLAAGHFKVLK
DNLQYLYKYTREDLEKQRIKIPTNQIFKCKLFHQKLKDCIRHHIAILQFVKMYETNFSFIVFNQFLFSILILGFLIF
QLSILEPLSYEFLQAIIYMVCVVVQIYFYCYYGTILYEESNSLNDAVYMGNWYKYDIKSRKTLLILMERSKKPCI
VTAGKVLDLSLVTFTTILRRSYSLLAVLKNQKZ

>TcOr30

MSSLVQESLHINLRVLELLWLYTPGDQTNFQKVRSCILFFVIMFHVPVLGGLHFIFGKNNNPTKLADNAFAV
TAMWCYAVKLWPLIGNRSKINDCINYLDKPIVALRENQKVILQTCSKICRRNSSIFLFYMTIAVIGFVTKPFLFE
ERGFPVDVWFPISLTDRLDVYWGFYIYVAIGVAYPVIASGVLDPLIPSLLCLATGHLKVLNDNLKHLDEYSNE
ANGSKDNNLYKNIQKCIKHHTEILNFVEFHQNCFSYMVFSQFVSSSLVLCFTCWNVSILQPFSFEWFQSLGYF
LILLVQLYFYCYYGTNLSEECDKITTSVYMGKWYKYDVKSRKALILLMERSKKPTIVSAGKILDLSVETFTTILR
RSYSLLALLKNQNZ

>TcOr31FIX

MTSLVQESLHINLRVLELLWLYNPKEQSKFHKLRSYIVYFVFMVHVPVLGGINLIFGKNDNPSKLVDNVFGV
AGLSCYALKLWPLIVNRSKIKDCINYLDKPIIELRENQKIILQTCSKICRRNSKIFLYYMIISVIGFVTRPFLFEEKG
FPVDIWLPNVLRDRLDIYWGFYIYVAIGVAHPVIASGALDPLIPSLLCLATGHLKILNDNLKYLDEYSKEASKS
KNYVLYKNIRKCIKHHIEIMNFVENHQTCFSSLVFSQFMGSPLILCFCCWNMSILQPFTFKWFESFAYFLILLVQ
LYFYCYYGANLSEECANITTSVYMGKWYKYDVKSRKALILLMERSKKPTIVTAGKILDLSLETFTNILKRSYSLL
AVLKTKKLIIZ

>TcOr32

MCLSTSEQSFSINLKIMKLCRLFPPTEGKKFYKIQAYLLQFLLLLPIPILGNLHLLLDENLDMEKVNYNAVFLA
QVTCFVIKLMAIIANSEKIKKCITELDSPKFAAVRENHKIILQHCIKVCKRNTLIFVVFVICGASSWATKPLFWS
RRNLPLDVWFPLDTTSTPVYCSLYIYLLIGVYFTSFANMVIDPLIAGLAYHATSQIKILKDNLQHLNNVYANEE
ITSSKNKIIYMKIKRCVQHYDDILSFVKEFEECFSLAIFSQISASVFVICFSCLQLSKIKTFGYYFIQLVFYFGVILAQ
IYFYCFYGSTLFEESSSIINAVYSSKWYDFDVPCRKALLILMERAQTPITVAAGKIMDLSLVTFATILRRSYSLVA
VLNNYQZ

>TcOr33

MSDIIGQSFGPNLLIMQILRLYPSQRYTRLHQLQACIMYVFFIILVPTLTIYNYIIQENFDILQFNYTATFLAETVS
WIAKLLPFITNANRMKKCITYFGTRYFEEMLQRYTSAKIMKECISVCRRNSTVFLYGVICGMTSFITKPLFWKG
YQLPLDMWLPFDATSGPGIYYTTYSFLAIAISYCAFAGTLIDPLIGGLACHATGQLKVLKVNLQHLKEYTETEV
KQSDETSDQIMYEKIRQCIDHHNAILIFVKEFENCFSLVVLSQFTGSMVAICFCCLQLSMVDLVSMSALSSTIYIF
IILGQFFFYCYYGSRLFEENNSLTNGIYMGQWYEYNIKLKKALIILMERSKVPMLITAGKILPLNLETFTLLLKRT
YSLLAVLKNYQQZ

>TcOr34

MSSLIQESLHINLRVLELLWLYTPKEQSNFHKMRSCILFFLLMFHVPVLGGIHLIFGINDNPTKLADNALTIAG
VSCYALKLWPLIGNRSKIKDCINYLDKPIVELRENQKVIFQSCSKICRRNSKIFQIYMITALIGFVIKPFFFEDKSF
PVDIWLPKALRDRLDVYWSFYFYVSLGVAYPVIASGALDPLIPSLLCLATGHLKVLNDNLEHLDEYSNEISESK
NCVLYKNVQTCIKHHIEIMNFVENHQKCFSSMVFCQFLGSPLILCFTCWNMSILQPFTFKWFESLAYFLILLVQ

LYFYCYYGTNLSDECANVTTSIYMGKWYKYDVKSRKALILLMERSKKPTIVTAAKILDLSLETFTIILKRSYSLL
AVLKNQNZ

>TcOr35

MEGTHSIKPNSLSSFSLLMVMEGLVKKSFRVNLLVMQILGFYPPQKYKILYKTYAYVVYCAFTILIPVLAALNL
LLGEDVDLEQISDNAFLVCQTGCFIIKFLPFVNNVAKIRNSLFMIECPLFHIYTKSQEYIIDECVATCRRNCRLFL
TFCIITLINWSISPFFLPGNALPVEIWSPFEHKASRKFYFWSFVYVVAGVGNGAISSGVIDPLLAGLISHATSQLK
LLKNNLQFLDEHAEEKIASLNISFTERGTVKADFIYQQIKLCVNHHIAITEFVNVYENTYSSVAFIQFAASVVVI
CISCLRLSMVEPFTFTFFAMVLFLCTMLSEIFLYSYYGTILYEENQTLTNAVYMGKWYNYDTKSRKALVILMER
SKRPMLVTAGKILDLSLETFTTVLRRAYSLLAVLKNYZ

>TcOr36

MKGLVEKSFRVNLLVMQVMGFYPPQKYKSLYKIYTYVVYCAFTTLIPVLATLELFLAENINLEQISDNAFIVCE
AGCFIIKYLPFVRNADKIKKSLFLIERPMFHIYTKRQEHIIEECVAICRRNCRLFLTFCTITVINWSITPFFLPGNN
LPVEIWSPFEHKASRKFYFLSFVYIVAGVGNAAVSSGVIDPLLAGLISHATSQLKVLKNNLQFLDEHAEERIAS
RNISFIERKRFKADFIYQQIKLCVNHHIAITEFIDVYEDTYSSSVFIQFAASVVVICISCLRLSMVEPFTFTFFVMAL
FLWTMLCEIFLYCYYGTILYEENHSLTNAIYMGKWYNYDIKSMKALVILMERSKRPMIVTAGKILDLSLETFTT
ILRRAYSLLLVLKNYESTPTEZ

>TcOr37

MKANFRIKMDNDPIYKLDLRDLVKINVKSIEFFGYLSPDFHSFLAKILYFIYASTFVGFMFVLYTTSEVINMILVF
GDIEKMTGASFLLLTHLVQISKLYVLIFHKSKVRRLINSINREEFQPKNQEQYNFLINDIRTSKTVTILFLLAGFA
TCALWAIFPFLDKTEKTVKLPLSGWFPFDTTKSPVFECAFVYQTVGALVNGLGNISIDTFLSGIIMVVSGQLKIL
NNSLQTMKPNCKANDTERRQKLIDIIIHHRSIIQFAQEMTWLFTTCIMSQFVVSVIIICITMFQMSLVSPLSLQFL
SMALYQACMITEIFLWCYYGNEVILQSGKLTQSAYMSQWLSSSKKFKHDLMFFMTRSQNPLKLYAGGYFTLS
LETFMAIVKSSWSYFAVLNRVHTTDNVZ

>TcOr38

MAHNLDVDLTEFVRFNIKCIQFFGYFSPDFRNNSQRKLLFRIYAALFVGFAFILSLLSQIANMVDAFGDIEKMT
EASFLLFTNLVQCFKIYTFANHGKKVWNLVYSMNRSDFKPNNLAQYWIVVNEIKTSKIISKLFLLACTLTCVS
WAISPLLDKRGSDELRLPLSGWYPFSTEKSPAFEFAYTYQIFTTWVGGLGDISMDTFMSGTIMVISTQLSLLKD
GLENVARNIKHDKSSVNKNLIQCACHYRSIIQFAAEVTNLFTTCITAQFVVGVIIVCMSMFQMSLVSVLSFQF
AAMLLYQICVLMEIYLWCFYGNEVMLKSDQLTQAAYMSEWLDGTEEFKQNLLFLMTRTQFPLKLYASGYFT
LSLETFKAIVKSSWSYFAVLNRVHSEEKAQQZ

>TcOr39

MSNQHEIDLTEFVKLNIKNMHFFGYFFPRFGHNKTRKTLYTMYSTLFVGSTFVLTALSQIANMINSFGDMER
MTEASFILFTNVVQCFKIYSFLTYGPRVWNLIDGLNRNIFKPINTDQHRILVNDIYMSKKISKIFLLACTLTCMS
WAISPFFDKRGDVLRLPLSGWYPFNTDKSPAFELVYIYQILTTWIGGMGNISMDTFISGIIMAISSQLSILNNAL
KNITKNNELVRCVFHYRIIIKFSDEVIYLFNTCLTTQFIVGVIIVCISMFQMSLVPVLSFQFVAMLLYQMCILLEIF
LWCFYGNEVMLKSDQLTQAAYMSDWTKSPNHFKQNLLFFMTRTQFPLKLYASGYFTLSLETFKAIVKSSWSY
FAVLNQVHSRQTQZ

>TcOr40

MSSLIQESLHINLRVLEFFLLYTPGEPTNFQKLRSSILFFALMFHVPVLSGINLIVGKHDNPMKLVDNSFGFVGL
SCYIAKLWPLIGNRSKIKVCINYLDKPIVELRENQKGILQACSKICRRNSNIFLYYMIISVTGFVTKPFLFEERGFP
VDVWLPTSLKDRLDVYWGFYIYVSIGVAYPVIASGVLDPLIPSLLCLATGHLKVLNDNLEHLDEYSSEENGSK
DSNLYKNIQKCIKHHIEILNFVYNHQKCFSLMVFSQFLGSPMILCFTCWNVSMREPFSLEWFQSLAYFLGLLL

QLFFYCYYGTRLSEEFEHVTTAVYMGKWYKYDVKSRKALIILMERSKKPTIVTAGKILDLSLETFTIILKRSYSLL
AVLKNQNZ

>TcOr41

MDNTLDIDLTEFVRFNVNSIHFFGYFLPEFGKHPKKKIIYVIYAVIFVGTTFGLSLVSEIANMINAFGDIEKMTD
ASFLLLTNLVQCFKMYSFLTHGPRVWKLIHSMNNSDFKPKNLEQRNILVEEIKMSKRISKTFFMACTIVCSLW
GISPFIDRGNSEKLRLPLSGWYPYSTDTSPGYEITYAHQTLTTWIDGLADVGMDTFLSGVIMVIAAQLSLLNNS
LKNLTKNCKNDGKKANTNLIECVIHYRTIISFADEVTYLFTSCITAQFIIGVIIVCVSLFQMTLVSLRSFQFFSMF
LYQGCVLMEIFLWCYYGNEIILKSDELTRSAYMCEWIEESREFKKNLIFFMTRTQFPLKLYASRYFTLSLETFTA
VVKSSWSYFAVLNQVHTKZ

>TcOr42

MPNSEDLDLREFVTLNLNCIHFFGYFFPRFKKPSKKKVFYYIYATTFVGFAFILSFLSQMAKMIESFGDMEKMT
DASFLLLTNVVQSIKITVFNTYRSRVWQLVESMNREVFKPRNATQYNILVHEIKKSKFITISFFIACVLTCASWG
ISPLLDTTVTNRRRLPLSGWYPFKTEASPVFELTYAYQMFVTTVGGLGNISMDTFMSGCIMNLSAQISVLNNA
LENMEPKKLRTDEEINESLVRNVIHHRNILQFADEFTNLFTTCITGMFVVGVIIVCISMFQMSLVPVLSFRFLA
MLLYQICVLIEMYLWCYYGNEVILKSNKLTESAYKCQWIDGSKKFKQHLLYFMTRTQIPLNLYASGYFVLSLE
TFKAIVKSSWSYFAVLNQVHSKQIEZ

>TcOr43

MDDTPVKKFFKVNLTVMRLFGFYPPKKCNIVYKIYALIVYVCCTVTIPTLAALHLLVSENVDLAQVCENSFVI
FEVGCFMFKLKPFIFNVEKIRQSIYMLNWPIFNNHSKQQEQIINECAWTCRRNTWLFLVFCIITFITWAATPFFT
SVYKFPIDVWLPFEATADPKTFFSVYLFIVLGIGNTAISNGVIDPLISGMVYFAVNQLKVLKDNLEYLENNSET
QLCFNLTVKHKTIYKKITMCIEHHNAILDFIKYYENVYSPVVFTQFTAAVLVICISCLQLSMVEPLTFTFFAMIT
FIVTMFLEIFLYCYFGAALYEESNTIIRAVYMGNWYTYDIKSRKALIVLMERAKRPIIITAGKILEVSLETFTMLIR
RAYSLLAVLRNYQSZ

>TcOr44

MKQIVKPSFRIHLTLMQILGFYPTRSYKKLYKLYAYFVYCFLTIPVPTLATVYLLVEDNIDLLQLSNSGFLICQIG
CFIAKFVPFWKKPEKIKRSIYMLENQIFTNHAPEQCKIIDDCIRICNRDCWLNLALVLSALTSWAVTPIAKGISK
LPVEMWLPYDVNKDDKTYYLTYLFVVTGTANGAIASGVIDPLIAGLACQATGQLRILKNNLQYLWENVDKE
ISDSEKDNALREKIMYDKIRSCILHHNSILNYVELFEDTYSSVVFTQFTASVLVICNACLQISMVEPFTFAFFAMI
SFIVTMLAEIFLYSYYGTLLYDESNSLTNAIYMGRWYEYDIKSKKALIVIMERSKKPMIVTAGKILDLSLVTFTTI
LRRAYSLLAVLKNYZ

>TcOr45

MNNHVQKSFGVHLRLLHILGMFPSRRISKIYNIYTYFMYCIMITPIPILVIINLFLEKHVDIVKISNNAFLTCQM
ACLSVKLIPFLGNIESVWTTLDMLEKPPFTTYTKRQEKLVDECITTCKRNCWLFLVFCIFSLISWASKPMGLEG
RNLPLEIWVPVDFTENTELYCFAYIYVVLGVAAGALSNGVIDPLIAGLASHATTQLKILKNNLQFLSEDADQV
IAQQLTKARIIQDKIRFCIIHHNAILDFVDYYENVYSGIVFSQFIASVFVICLACFYLSIVEPFSVIFFAMVIFLVTV
LSQIFLYSYYGTLLFEENNTLTNAVYMGQWYTYDVTSRKALIILMERSKRPMTVRAGKVLDLSLETFTTILRRA
YSLLAVLKNYZ

>TcOr46

MSKSEKIHTLATYFDSNIAFLKLTAFWIYDDETTRRKKYLQHAYNIFWIFYLFVAYQPAELLYVYYSFNDLSVF
LRALRDIGNHVSLAYKAFNYFIMRRDILKLMETLQHGNYHYEDCGDFQPKLIVDEEKKEALKWTKYFLNFC
NAICLSMFANGVFTFIFLSDKQYVERNGQRVYHQEQPVNTVSPFGSGTKLRFFVTFIYTMIALTFYAWTIVALD
SLFITIMSCISSHLKILQGAFKTVRARFIKLCASLSKLLISVSGKLESIYSTQTFVQTFISLGEMCFSLYLLSETADQ

NIGNEITYLIATGFELLMYCWFGNRITEASLKISYALYESDWFPTSLSFKKQIIFTMTRMQKPINVTIGKITPLAFS
TFLTIARGAYSFFTFLKQRHGINHZ

>TcOr47

MYLDLNKGIADNTVLFRIMGHWPFGNPKLYRVYTHFVLINMYLYNLTSLINMLKNLDDTEEVTATIYNLLST
VAVIIKANIFHYHFNHVKTIVTMFESEAFQPKNKQQEKILKNGIFWARFIFYYFFLTADLTLVMWILFPIMDGE
RRFPSNAWFPYDYLSGRNYTLTYIWQSIFIIYHALSNVCMDTFFAMLMVQTGAQCDVLNNQVSLLGKESVDS
DTVRGELGKCIHHHKLILKLAETIGLVFRNIVLVQFATSVSVLCETMFLLSLVKTLNATFVMLLFYQVAIFTQIF
LYCWFGNEVVLKSAKLYYSAYESRWYECPQSFKKDLLFFMQRTQKPIVLFVGKMFPITVITFTSILRSSWAYFM
ALRKVHDKSZ

>TcOr48

MPHATLSKMVVQKIDLLEPFDNVTRLLKILGLWYSPNETIVYKIYKNFVMATCFLYTLTCTVYGFKFMSFETL
EIAFGAVEGVLKSLMFRLKFQKIAESWQQIRQQEFQPRNEHQRTVLKWYIEVTKSLFLVYFFGVYIGCISALTV
SSWLRHKDFPTDHWFPFNYRRPFLYQYIYVHITVGFYLTAFLNCASDSCFYLSLLHITAQCEILADTLKNVHD
LHKLNAAKKNSGQKGEDEVMNQILIECMKHYNLIKKYTSLVADCFKEIITLQFVPTIVMICIAMYKISTLEPSN
TQFWFFAFTELGAITQIFIYCFVGNLVTSTSQKLFYATFESQWYNASQKFKKNLITVMMAVQRPVIFYGWNIF
AINYATFKSIVQTSWSMCVAFRSTQDLZ

>TcOr49

MVVEKINLREPFENVTRLLKILGCWYFPNESLVYKMYKNFALITCCMYTVTSIIYSFKYMSIDYDKAYESLEIGV
GTAEGVLKGIIFRMKFQKITESWQQIQQPEFQPRNEKQKMLLRRYIYVTKFLFKVYFFVVYIVCVTGLIVSSLLR
HKDLPTDHWLPFDYRKPFLHQYIYLHLTAGLYLNSLTNCAVDSCFYLSLLHITAQCDVLADTLKNIHDLDKL
NAKNAPERENKDQVMNKILVECMKHFNLIKKFTNQITDCFKEILTLQFVPTVAMICMGMYKISTLQASSSQF
WFFVCTDLGATTQIFIYCFVGNLVTTTSEKLFYATFKSQWYNASQKFKKNLLTFMMAVQHPIIFYGWDVFAI
NYETFKSIMRTSWSICVALKSTQDLZ

>TcOr50

MVVEKINLLEPFENVTRLLKILGCWYSPNETAVYKIYKNFIIATCFIYTVTCNIYVFQKMFTDSDKAYETLEIAV
GSAEGVLKGIIFRTKFQKITESWQQIQQPEFQPRNEKQKSVLRRYIEVTKTFFKVYFSLVYVGCVTGIVVSSWLR
HKDLPTDHWLPFDFRRPFLYPYVYVHVTVGLYLNSFTNCVLDSCFYLSLLHITAQCDVLADTLKNIHDLDKL
NGKNVPERENVDEVMNKILTECMKHFKLIQKFTNVITDSFKEILTLQFVPTIAMICISMYKISTLHPSNTQFWF
FIFTDIGATTQIFIYCFVGNLVTTTSEKLFYAAFESQWYNASQKFKKNVITVMMAVQQPIIFYGWNVFAINYET
FKSIMRTSWSICVALKSTQDLZ

>TcOr51

MSVKINLLEVFDNVKFLLKFLGCWTFPEENALYKIHKYFIIITCFLYATTCNIYGLKHAFIDPNKAYETLPIAVG
TTEGVLKSYLFRRNFAKVTESWNQIQQQEFQPRNEEQKTLLKNYIAMTKFLFKIYVVGVYICCVSAIVISSWM
RHNELPTDHWIPFDYHRPFLYQYIYVHLTVGLYLCSFLNCALDSCFYLSLMHITAQCDMLANTLRNIHDLEK
LNAAHRVTHCKNKNQVMNEILTECMEHYSLIKKYTNLVTDCFSGILTFQFIPSVGMICICMYKISTLDISSSLFF
QFIFVEAGSITQIFFFCFFGNLVTVTSEKLFYSTFESQWYNSSLKFKKNLITLMMTFQEPITFFGWNIFAINYETFK
SIIRVSWSICVALKSTQDIZ

>TcOr52

MSQIDLKEAFKQNIVLLKAMGLWFFQNERFYKLFKCFVQGSLVFDSTSLIIYVALNIRIKNVTDTIYSLPGSLEV
VLQAILFRKNFHLIRKSLNNLKQKEFQPKNDTQEKILKDSIALSRRVFYSFFWLVFVMIGMWMVLPLTKKGKY
LPTKYWIPFDYRLPVVYELLYVFECSCIIFHAFSNVALDTFFSIAMIQIGAQCDVLCDTIRNMDEQEKTNTMDR
ILIECVHHYRLIEDFAKSIATSFKEILMVQFVCSSLMLCVSMYELSLSEPMSGHFFQVLLFQISATNEIFLYCWFG

NEVIIKSERLFYAMFESKWYDSAATHRKNLMIFAHQVQKPISLLVWNIFPVDLKTFGGLLQKCWSFFVAMKN
IQEIQEZ

>TcOr53

MGFNFQSRTNKILRRLRYVGTWFENDSYDLYFLYAVLLNIYYNLHNIAQTMNVFYHLDDIEEWSSSGLLTLTT
LLTNFKAYCVLTNKKRILKLNQILTRSVFQPRSDHQVKMATDKFKIFDTMYSLHSSGPTLTVVFFSLYSLAELE
NRKLPFNAWYPYDFKKTPNFELTYLFQFTACMVQALIHVNTDSLAFNFIAILVIQLDFLADNLRNMCQKAES
MEQSLLDCIRHHKEILACRNELYHILNVNLFGQFILSTTALCMTFLQMTVVNPTSTHFIAILVYGMALLVELL
MFCWWGNELIIKSQLIPQAAFESNWMEGSIFFQKNLVFFICRAQKEMMLYAVGFFRISLNTFILIIKNSYSFYCV
LSHTGASRVKLZ

>TcOr54

MNLQKLDPLEGFKPTISMLKIFSVWNSSNMFYKIYKNVTTLSLAITYTCVMICVVVNFNVSEINENFYYIPALS
TAPFKLVIFQKSFKKIQNLLFLLQSQYTKIRSEKQAKMVEDSVVLSKRVVKVFAVLVVPTCVGLFGMPLLKDEI
KLPLIIWIPFDYHEPVVFGLVYFVISFSGSFTAYINIGTDTFFYNCLIQIETQCNILSDTLRNLHEFGRFEAEIHTILI
ECIEQYKTILKFTKILSKTYQGILSVQFICSLLSLCLTMYRMSLADPGSEEFLRYFVFQWGVLPEIFLYCYFGHRV
LDSTKNLYYSTYELQWYNTSAKFKTNLLIFMGQIQNPIVIYVAGIFSLDLETFKKIMQKAWSFFTALRNIHEQZ

>TcOr55

MKAFSFNIKLFQIFGQWCYENESFYKIYKYTATVLLFLDWLFTMIFVLVNFQESEVVDSLYISPSMTTSMLKYVI
FRVNFSQVERMLKIVEEQYLKIDSRRVGFLVERGTKSSVFVIKACFYLVMATVVSLITQPLLQEDIDIPLVIWLPF
DYHRSGIFELIYVYVSVSYLYFAYVNVATDCFFYISAIQIGVQCEIVGFMLENLNEIAKQEEENVRRLFLSCVTY
YNNILECVKIISDCYREILIVQFFCSFVALCMTMYQLSIVEPFSDVFFKMCVFQSAVICEIFLYCFFGDLVLEKSG
KLFYASFSFGWYNGSAKFQKELLIFMNQLQKPIIFHVGNVIPVTCETFKSIMQKSWSFFIALKNTQNRZ

>TcOr56

MVSTPLTNPLFSVAKLNAFKNTTLLLKIVGMWKFKTESIFYKIYKHVMVLHASFACVLSFLYALKKYKDPEAI
FFISYLPAIFTVPIKLVMFPINSGKIKQLLELLAAEKAIIRSAQQEEILENAMKLSRQIFNLFGGSFVAAAIVILVTP
LSVRSHVMMYYEWFPVDYSKDSVYYGIFGGSLYFLLHLIAVNAAGDFFFYISAIQIEGRFDLIVDTFLNLDEISA
RDGNSTKYERMHEIVIECVQQYNIIIECSKLLIDCFKEILINQLICSCLSLMFSMYQLNAAEPLSLNFFRIIFYAIA
MGSEIFLFCFFGNRLIVKGEILYYSTFASGWYDAPLKIQKDLLIFMQQLQKPVMINVGNIFPLNYDTFKGIMQK
SWSFFVALKNTQDLRSKNZ

>TcOr57

MSHSNPLEAFKLNTFFLKALTVWHVENPTYRLYKIFVVFSFAVTFFSAWICALVNYNVSEISENFYYLPAMST
GPLKYAIFQKNFTNIVNLTHLLETQYAKIRTENQKKIFDESVIFERKVMKNFAILIIPTCVAMFIVPYFQDRREM
PLIVWFPFDYKQPVVFDLVYFILAFACISIAYTNVSTDAFFYTCLIQIETQCEIVSDTLRNLDKIVTNGFRNVAES
RKIFIECIEQYNVILRYTKIVSDTYQGILVVQFFCSLVALCLTMYKLSLADPGSQDFIKYFVFKLGVISEIFMYCYF
GHRVLEKTEDLYFAIYEMHWYDASKQIQNEVFIFMGQLEKPIVFYVANIFSLDLDTFKKIMQKAWSFFTALK
NMHDIRNNZ

>TcOr58

MPFTIKDYDLRNAFETERTLLTLSGFYPRRTKKYNFFYNTSALINLFIAYGQLFSMVVQMVIDRNELSKLSETLL
FFMTHFTFLCKLTNFVYYKKKMFEIEDNLSRKIFYGFELWQIKPKIDSCKFIAKIFRILCILVVLFYTLVPYLDDK
EDLSLPLPGWLPYNTKKYYYPTVIFQVMSVSVSAYNNSSIDVLTCMLITVASAEFNLLKGALKTIDFHPKGHN
TKQLIEAKFENCVNHHKEIVKFAYQIETIFSKGIFLQFFASIIVICFTGFQMIVVPIPSMQFIFLIIYFSCMMCQVA
MYCWYGHDIITTSDSIGQAFYMSNWYESDVKIRKNICIFLERTKKPVILTAGKFVTLSLTTFTTILRSSYSYFAVL
QHLYKEDSZ

>TcOr59

MDEEFLIGTFETEKKFLRYGSFYPCGKRIKFIFLGLFMFVYSWTEFLSMITVLFVERDNLTKLSETLLFCMTQAA
FLFKLVNFLYHNKTMLRIESILKNPILNCLDQFEKNIIEKYMIRVKYLARLFRILCILTVSFYGLFPFIDEDPDHM
LPLPGWFPFDVKTHQIELVIAQTCGIAIGAFLNSTLDILPTILITLGSAQFDILKIRLENITSVDTSKSWLVKKAIK
KCVIYHTILLNYITQIEILFHKGIFVQFTASVVVICLTGFQMLVISVRSIQFILLMIYFSTMTCQIALYCWYGNELM
YRSMGLSDACYMSEWNKCDTSVCKSLAIIMERGKRPVVLKAGNIFSLKLTTLMTVLKSSYSYFAVLQRLYATS
E

>TcOr60

MSEDYTFRNVFAREKKILTISGFYPLREYEKNYFHFFSGTIQWIISLGMLFSMIIQSVIKRNDLMVLSETLYFLTT
HLTFVCKLANLEYHKKLLLDIEDMLKTTRFQKTLSLDLIEKTGMNEKIRKFNLVAKTFRIVCVWCVVLYVLVP
YFDPGKSKTLPTPGWFPFNWTDKYYYGTYFFEVAGISITAHMDSSIDILSWLLVTIASFQCDILKENLKNIYYNY
DKEHDIRETFKDCIRHHEEIIKFTTKVEQSFSQGILLQFLCSALVICFTGFLMLVVPVLTFQFANTIMYFCCMMI
QLGMYCWYGHEIMTTSDEIGQYFYLANWYDSSLTLRKDFAIFLERAKRPITLTAGGFVVLSLNTFTRILRSSYS
YFAVLKHLYNKSZ

>TcOr61

MGDYDFRAAFAFEKAIFSLSGYYQRQAGFSSLIICAIASLITIAQFLSMVMQIIVAGNDLTVLSETLLFFMTHFTY
MCKLVNLLFYKSKLLHIEDLLSRPRFYGFSQNELTIIKDGIEATNTVANLFRIFCVLACIAYGLVPYLDHTKAM
ALPLPGWLPYDTTKYYYPTYFFQMVAVSITASVNSTIDILTWKLITIASVQFDILKRKLKDLDYKLETTSLQIQF
KTCVKHHKEIVNYVKNVEKTFSKGIFIQFFASVIVICFAGFLIIITPVLSMQFLYLTLYFMCMISQVAIYCWYGH
YVMTTSDEIGQDFYMSNWYESDVAFRKDIIIFMERVKKPVTFTAGNFITLSLVTLTRILRSSYSYVAVLQHLYNE
VZ

>TcOr62PSE

MIVTIKDFDLRTAFNTERKILSFPGYYPKRDTEKMAFXTIVTSVTTTMISFGQLLSMIMQIFVARHDLSKLSETLL
YFMTHVTYMCKLANFGYHRSKMLHIEDLLTRSRFYGFASNELKQVTQKVASTKMVGTTFRLLCVVCVFAW
GMVPYIDHTKAQALPLPGWLPYNTTKYYYPTFVFQMVALSITACINSTIDILTWMLITIASAQFDILKEKLKSID
YRHELVTIEFEFKACVKHHKEIVNFVHKIEHTFSKGIFLQFFASVIVICFTGFSIMIVPLFSMQFGYLLFYFLCMM
TQVGMYCWYGHDVMTTSNEIGQYFHMSNWYESDLRARRDLAIFLERIKRPVTLTAGGFITLSLTTLTRILRSS
YSYFAVLQHLYKNSZ

>TcOr63

MGFMIQDYDLRNAFSLERKLMLVVGFYPKRDNKHEILYWLSAFFNLLISYGQLTTMIIQMVFDRSDLSKLTES
LLYFFTHFTFLCKLLNFQYYSKDLIEIENFLTDPIFYGYSFEQLDIIKAKIRSCAFISNAFRICCTFTCSFYCLVPFID
ESRKKILPLPGWFPYDTTNYYYSTFFVQSLSLFISAYCNTAIDILTWKLITLASAQFEILKENLTKIDYEGGFNET
KGALVRCITHHAKIVNYTERVEAIFSKGIFLQLFGSVIVICTTGFQLIVVPIPSVQFAVLGTYLCGMTTQVATYC
YYGHEVMTTSDAIGMSLYLSNWYASHVKIRKIVMIFLEKTKKPTIVKAGNFITLSLATLTQILRSAYSYFAVLQ
RLYKDSZ

>TcOr64

MMSDEYVKDVFIANRWMLRCAGLWTPSTRSKLVQIPYKIYAIVVFLFVNVYFTSTEFLSLFYTHKNLYNFIKN
VNFFLTHFMGAVKVIFWFFKGHVLRDLMRTLESPEFHYEPCEGFQPGLIWRKYRRIGFKYSLGFLALAHMTLS
SSYIPPLLTKLPYFSWMPFSYSTPRSYLLALGYQAGPMFSYAYSIVGMDTLFMNIMNFIAAHLVILQGAFASSK
MRVLDPGQMNNEMKRNCRHLQTILRVSEDLERVHRYLTLGQLTATLFILCTSLYLISTTPASSKQFYAELVYM
VAMGFQLYLYCWFGNEVTLMASEIPVNVWKADWYDCDQSFKKSMIFTMTRMQKPIYMTVGKFAPLTLQTF
VYILRTSYSIFAVIKNTSIZ

>TcOr65

MTATKSLKEIPPIYLRVHLTVLQILGIDILPVESVPQNLFYTYTALIISTMCLFTIAEFLDMVLNYEDIYRLTFGLC
YCVTHVLGTVKMFLMLYLRKKLWGNLTTLEEGIFKPNPTRGGPEELQIVNDAITMCNRQGYVFYTLVFLIIGA
RLLYASLANWPYDKHNYFDGNVTVIVNTKEMPYTTWMPFDYNDSPLYETIFAFQIFSTTVYGFYIGAADAVI
CGFMMLIKAQFLIVKRELETLIERAQKAAIAENPDNEDNFGREIERIELLDKRTQDYVAKYANECVYHHQELI
ALCDHAEEDFCYLMLLQFISSLLIVCFQLFQVSTLSPDSVEFFSMVCYLLLMLFQLLCYCWHGNEVQIVSGELS
RYAFGINWIIMRESPKKTLLLLMMRAQRPCYFTAGKFSLLSLQTFMTIVRGAGSYFMFLRQMNIZ

>TcOr66

MSKNLKEIPPVYLKVHLTVLQILGIDILPNERIPQTLFYTYSVLLIATMVVFTTAECLDLVLNYEDIYKLTFGLCC
CVTHVLGAAKMFLMLYLRKKLWGYFTTLENGIFKPNPCRGGAEEFEIVTSAINMCKRQGYVFYVLTVGVTG
GQGLYAALANLPYDKHNYFDGNVTVVVNTKQMPYATWTPFDYNDSPLYEIMFAFQIFSTTLYGFYIGAADA
VICGFLMLIKAQFLIVKRELETLVERAQRAGNPDRGDFGGGINRIEMLDDGTQVFVEKCANECVYHHQELIA
LCEHAEEDFCYLMLLQFISSLLIVCFQLFQLSTLSPGTFEFFSMACFLLFILFQLLCYCWHGNEVQFVSGELSRY
AFSINWIIMRESPKKTLLLLMMRAQRPCYFTAGKFSLLSLQTFMTVVRGAGSYFMFLKQMNTZ

>TcOr67

MDFTIRDFDLRNSFSLERKLLLVLGFYPIRDKEKHRILHQLSAFLNLLLYYGQLLTIIIQMVIDRNDLSKLTDSTL
YFLTLFTFLCKLFNFQYYGKDLIEVEKSLTDPIFYGYSFHKLQIIKAKVRSCTLVCLAFRISCTCSCFIYSVVPFIDR
SGQKTLSIPGWFPYDTAKHFYITFFLQSLSLFISAHCNSATDTLPCKLISLATAQFELLKDNLRTIDYENSFEETK
HALVKCITHHRKIVNYTKRVETIFSKGIFLQLFASVLVICTTGFQLVIVPFGSLKFAIHGIYLCAMTAQIAIYCYY
GHDVMITSDEIGTSLYMSNWYASHIKIRKIMVIFLEKTKKPTIVLAGNFITLSLVTLTQILRSAYSYFAVLRRLYA
DDZ

>TcOr68

MPSLIEQSFKINFRILKITAFYPPTKNKRLNQMLAFVLYMLFVVSVPILEVLNLVLQEKITFKQIIDNAFMIAELG
CLIPKYWPFVRNNDRLVKCIHYFDSPAFQPTKKKHREILQNCVKVCRAITIFFVAAVSSGYVSWSSRPISWKN
HIFPTDLWLPYDPKVAPKLYNFLVYTYLIKILKDNLQHLGEDTEAEFNQQSVINRLPKSEIMYRKIVKCVEHH
NLILEFVKEFQYCFAQCAFSQIAGSVVVLCVSCLQLTIVDLLSFDCLAMILFLVSMLSEVYFFCHFGTLLYEESST
ISDAIYMGSWYDYDKKSKQALTILMERTKRPVIVIAGKLVQLSLITFSMILRRSYSLLAVLENYNIEINZ

>TcOr69

MPSIIDISFKFNLKVLRISGLYLPEKFKSLYQIYSYLAYFLLVIPVPILECTNLLVQEKITFRQIADSAFLIAELGCFI
PKNWPFVRHADRLKRCIHYFSAPIFKTKRKEHEEILQDCIKVCHRSTAFYFASVTVGFFSWAIRPISWKNHIFP
TDIWLPFDPHTASKVQVAGVYFYLVLGKGFSSKVLKFWFNFSCGIKILKNNLQFLGEYVDEELATQMHSLSPS
EKTQLTYQKIRQCVIHHKHILAFVKEYEECFSQVAFSQFIGSVVIFCVSCLQLTIVDVVSLDFLAMMMYLIATLS
EVYLYCHFGTVLYHESNTISEAIYLSKWYEFDIKSKKALSILMERLKRPMIIKCGKILDMSFITFTMILRRSYSLLA
VMENYNIELNZ

>TcOr70

MPSIIDISFKININVLCLAGLYLPDKFKSLYRVYTYLVYVFIVIPVPTLGCVYLLAQEKITFRQIADNLFLIAELGC
FIPKYWPLVRHAERIKRCIHYFSAPIFKTDRKEHQEILDDCIKVCHQWSAFYFASVTAGFVSWSIRPISWENHIL
PTDIWLPFDPHTASSAKVASVYFYLVLGKGFGLGIKILKNNLQHLGEYVDEELASLEPCRKAQLTYQKIRQCV
IHHEHILAFVEEYEECFSQVALSQFVGAVVIFCVSCLQLTIVEVVSLDFLAMMMYFIAMLCQVYLYCHFGTILY
DESDTISDAIYLSKWYEFDKRSKKALCILMERLKRPMTVTCGKIFTMSLVTFTMILRRAYSLLAVLENYNIELN
Z

>TcOr71PSE

MAELKYFKGIVLILKWSLLYPLVKSWPKKCLYYVWALLYVLSFILTFVQCSMYLYKTPFNLIEEAMIIMNTADX
IYFSLFINFTFLYTKRTKLSRFVKDFNQKCLDQTDNKIEKITMEKSSKLSDKFVFYWTFNCIASSLMPMIASLLG

GNKNLPMVVWYPYDPNKTPYFHLTYIWEIFCISNLGLIYAVLDLVFPCIAIVLGQQFKILASNFKNNVYRALV
DSEVSEKIVQTFSKNLHNDSFNEEIFEIMNSAKFKKNNARYLRKNVKHHQQLLQYCADVSDILSIFLMGKVSA
AIFNTLFMAFSLITNADIVGTLYESPWYMCDVHFQRTFHIVQMRASKIVNVKAGNYFTMSASSYITFMXNRSG
SYIALLKELTDRGKZ

>TcOr72

MAKLEYLTGATFTLKCAVLYPIDSNNPKIKKILYAVWAIFFILTFVTGFIQCFVFVCINPFDLVQEAMIIMSLVF
YSTTFFYFIVFYKNWQNMVALVTNINKNFHRATDNVIEKISMDQASELSDKLAYVWTSSLAVGSVVPVVLAI
ATGNLEMPMPAWFPYDYNKSPVFEITYLWQVFCLITLAIIYGASDMFFPCITIIIGQQFKILASNFKNNFYTSLIK
LGAEESIVQNFSKDIKTHEFRSFYIKYGNIFKILNNAKFQTLNRAFLKRNIKHHKLLLRFCEDLNKILNTFLLIR
VSAIVFNLIFIGFNIIINADFLWTLYECPWYLCDVTYQKMLILVQMRVKRMVSTKAGNFFTMIAPSFIAFQRAV
FSYITLLKEVTDLGKDZ

>TcOr73FIX

MTRKHIFLNFTVTILKLSFLWPSNDNYDQWRLVKDASLIVSLMPCALPILAHFVLQITGDVYNMVTITENLIA
LICIIGMIYMTICFVKNRKLVKTLVKNLPAFTKYSKTTDIILTDKKANLYTKIFVFYGVIGNVVYMIMPYLNIEK
CQQRQNNDVPCGLVTRCWFPFKFDYSPVFEIVFVHQFYTCLMVSVIILDLTMLICGFLMHITNQLKHLRGFIK
RFDCSSQKIAEDVIYCVKFHTAIITYSEKTNEAFGTMMMLHITLTSLVISALGFEILIVDNFNDSLRFTLHLLGW
LVLLLLICYYGQLLIDESIAVAEDIYYVPWHLAPVDVQKDIYMILMRSQKPLTLNAANIGVMSFPTFLRVISSA
YSYFTLLLNIKSZ

>TcOr74PSE

MTSFHPQTTQSSFPPKMSQVQHLKFNVLTLKLIMLWPPNGHMTLNYHLKSLSLLIIVYLSAWTCIYGILRPLFT
ESDGYDVLIQRAIATIDFIGCIYMRYCFLDKIQNVTSLIRELPTFEKFCGKVEIETTEKKVQKYSKAVTVYWFFG
NLMNCLAPLHERTKCENLRKSQLYIERDPCGLMARCFYPFDVSKKLFPMAYFIQVYTCVVISYYVIVLTMTLV
GIMMHILTQLKNCRNLIHNLHEEITERESLKKKIQRIIVYHIKIIN

>TcOr75PSE

ZKLNFNWTKIIETNIVVLKINGIWPETSFFAWKNXLHDDALVLQTIQLVLIINDFQLFLTRLTLLLQQYILLINF
MLZXLRELKNVFSLSTIPYFSHKIVTKNDFQPFNDKLNSRKKFYVSFYSMVGFALGFLFKLPTGIFLNGGERQLI
LTCWFPCDSZTSPYYEFTYFYQSLAVAYAEIIFLZTDTFVTLLMTYXZTSCDLLYENLRKLAYSSTKNNTEQEFT
RYIKHHQNLMKLKNQCVDVSHMLIFIQVATSAIFIAFTLFPMTVHVSTFDAILLVINTRRNFGNVLSKPHTDA
DTKTDSSFTKVLRTAWSYFALLVQLNEZ

>TcOr76

MMESTVTRLKRMYLWPTASVTSRKPAFFLITFSCFLLYGSVMHLIVNDISMEEVHVIETTAGQFGVLYYLTLFTI
YRKGILEIYADLSNFTKFGKPYNFDKRNKQLNQWSRWFSVVLYFFVISVFAWPGIFTQSCEDLNVALNKTEVC
GVVSPVWLPFRFDYKPMKQFVYFWQSFCCLYSNGGAGTISFAMSETIEHLILRVEDLKILFPKIVAERSPEVRR
KMLAKWVDYHLWLLSIGKLMNDTYRYSFSVIVLCAGTLFGCIGYTVMKNASTNFNSSFIFFGWMESVFVICV
CGQRLMDAFHSVGTTVYNSEWCDTDVDFQKGVILITIRAQKPVRIYAGPFSYVSHLLILTVFQTSYSYINLLNA
SSZ

>TcOr77

MKYILMKKTIAFLSVTGFWPKTKESTKTRAFCILFSSSFLLFGSLGYLIVYRKFGSDDIDSIETATSHFGVLYFMFF
WILKRDGLVHIVNLLSDFSKFGEPRFFNDRNRQLDYLLQYCIFVLSVATGGVFLCPIIFVKNCEMVKQEKNLT
KVCGLVSNVWAPFDYSEYPMKRVVSLWESYCCFINFGCGGIMSFTMIKTMEHLHIRVEQLKDMFPDVVNEK
NLAVRKQKLEKWVKYHLHLYDIGELMNNTYRYCLSVIVLCVGILFGCIGISTMQPGSSHNSLFLFMGWFQSIC
ILCMVGQRLLDVFLSVGVMAYDSAWYEKDVDFQKAVLMIMIRARRPVLIYAGPFTNLSHLLILGVLQTSYSYI
NLLNAKZ

>TcOr78

MGHAIMTEILTYLTLMGFWPRSPKSSKASAFLIILSTSFLFFGILFYLIVNRQFGSSEIDSIETITSQFGVLYYLILFT
WKRNDIVEIVELLSDFSKFGKPPFFDQRSTRLNYRLSCIVLILIVANIVVAALPVIYIDSCHKANEQLNLTKTCG
LIAPVWLPFDYNEYPRKHLVFAWEVYCCVMNYVGSGIGALTMVGTMEHVIIRIEQLKYIFPKILDQPNPRIRE
QMLKNWVRYHLALFEIGRLMNDAYKWSLSVIVLCVGALFACIGISMLQSTASQINSICLFFGWFPSIAFLCMW
GQRLLDSSLSVGTAVYSSRWYDMDVAFQKSVLMILIRSQKPIRISVGPFTHLSMLLLLGVFQSAYSYINLLNATS
Z

>TcOr79

MGHVIMNEILTYVTLLGLWPRSRKSTKTISYLIILSSSFLFFGSLLYLVVHRKFGSNEIDSIETVTSQFAVLYYMTF
FTLKREGTVRIIDQMSDFSKFGKPPLFDQHNKRLNYLLSYFVICLFVAIVGVVALPAIYTGSCHKANEQLNLTK
TCGLVAPVWLPFDYNGYPLKFLVFAWEGYCCIITYACSGISSLVLVGTMEHLIIRIEQLKLMFPEILNEANRHIR
EQKLKNWVQYHLALFGIGKLMTATYTYCLSVIVLCVGILFGCIGVSTMQSASSNNSVFLFLGWFQSLIVLSVC
GQRLIDTCLSVGIAVYNSRWYDMDVSFQKSVHMILIRSQKPILIYTGPFSYLSHLLILSVLQTAYSYINLLSARGZ

>TcOr80

MGHVIMNEILTYLTFLGLWPRSRKSTKTVAYLIISSTSFLFFGSLFYLIAHRKFGSNEIDSIETVTSQFGILYYWVL
FTLKREGTVEIVERLSDFSKFGKPRFFDQRNRRLNYLLSYFVLVLMVAIGGVVALPVVYIDSCHKANERLNLT
KTCGLIAPVWLPFDYNEYPRKNFVFAWEVYCCIMTYACCGIAALVLVGTMEHLIIRFEQLKLMFPEILDEPDR
HTRQQKLKNWIEYHLTLFDIGKLMTSNYTYCLSVIVLCVGILFGCIGVSTMQSASSHNSVFLFFGWFQSIGVLC
IWGQRLLDTCLSVGIAVYSSRWYDMDVSFQKSVLMILIRSQKPILIYAGPFSYLSHLLILSVFQTAYSYINLLGAK
GZ

>TcOr81PSE

MRPTLDFMSFCRIWPKRNEELDPSRWYYIQFVVVLLSTVINLIGSVLHLSKVSIPNRSFYHVAEDLITITAQIGV
YYYIIVYVPLQRVAARLYLQLSSFELFGKPPAFDDHSVKLKYLAIIYKSGLNIVVIAYFXLPIITKHICERQNAAR
HFDDICGLVTNSWMPFDIKKFPNKQLFYLWQTYSVYWTYEGSAAISYLIVATMEHVLMRIWHLQGMIQEAI
NTEDAQVRKSRLDKCLEYHNHVFLLAADVDRIYQRSLFVHVLFSGVLFGIMGFSILTVGISVKTLSLFVVWVC
AAIFSSLSAQRLYDGSIAIGEEVYNSKWYDRDYKFQRDLITIMKRTQKPITIHAGPFAEISNVFILTIFKTAYSYLT
LLKASNNZ

>TcOr83PAR

MISPRKLLISSVTCLQLKTSFIWPPKNEEMTPSRWFYIKLAVLLTVSVIILYVPVCIHIHKLFERDLDASEDFALM
FSYYGFLSTMIFYSKNHKKICGLIYNLCSFEEFGKPPQFDELNQRLDFYSKLASGYTIIGMAVYIGMKIYKIPDCK
AERGAIQVCGLTVPLWTPWTVDTFWSYIAHLIVTLFYSMVFTRATLFYSVQV

>TcOr84

MTEEKELRLCLWSCYYLKLSLMWPLKREEFKSSKGLYLRLLVFVIISGSTFTAMIFMHLYKSLKVGSYDVSEDL
AILASNIGYVLMMTMYVSRQKDLELLLLDLSDFKTYGKPPNFDKVRKRMDLYAHLIFFYSMFGSFVYNMDKII
LIDKCKEARRINEVCGSAIPFWTPFETEDLFTLTLVITYVLINIFVVVKVAMTVSVQVLEISSHINLRIEQLKIFIA
GCFDRDFKASRERLDFCIRYHNVIIDFSERFSRCFSYVMFIHLAITGIIIGCLENQIVQEHQPEAMLHMGGWST
ATFIACYGGQLLMDASTSIADEFYNCPWYEADVKMRKDLILIILRAQKALFVSTGPFNVLSFALFVSIMKLSYSI
FTVLSZ

>TcOr85PSE

MTEDTELQLCLSSCYYLKLSLMWPLKGEKPDSKWFYFRLILGVFATILTFAAPVLMHLZKLWKVKGMDISED
LGIFAAIIGLVTMMVLYVVKQKDLSSLLQDLSNFEKFGKPPNFDKVNKRLDFLVKFVFSYAIFGAVVYNLMRII
EIPHCKRNRKIREVCGVFVPLWTPFDADYPPVLVLVASVVFIVVILIDKVTLLVSLQVLEISCHIRLRIEQLNAMI

LNCFNGDLQSSRKRLNDCVQYHIDIISYSERFNNCFTNGMFIHLATTGIIIGCLENQNVEGATPGGVLHLFGW
IISLFTSCLAGQILLDSSTSVADALYNSQWYTADVKLRKNLILMIQRSQKPLFLATGAFNIMSFALFVTVMKMS
YSILTLLQZ

>TcOr86

MALNQEDAICSKSCFYLRYSFLWPEEAPTRSFYAKFILVLILSFLTAFLPLFIHFLILVERGLDPSEDLFVIISYTGF
ALIMIIYVIHVKKTSYLIVQLSDFEKFGKPRGFDYWDKKFRLISSGVYYYVLIASSGLNLGRWVGMAECRKERD
FQVCGIVIPYWLPWKVDSWLFFILLDLYVLKMTLVVNCALFLIIIQILEITTHLKLRIDHLKEMLVKCFDSDSQT
NRKQLVNCIRYHTYIINCSKLFKKCFTHAMFSLIVTMALSCGCLESQVVKFDLWALPPISAWIFILFIACMAGQ
ILMNASLSIGDAGYHSKWYQTDANFRKYLILVLMRSHKALVLSAGPFNILCFELFVAIMKFSYSVFMLLNQNZ

>TcOr87

MKHVIMDELLIFLTFLGLWPRTPTSPKIISYLMIYSTSFLFFGSSIYLILHRKFGSDEIDTIEIITSQFGVLYYLTLLVV
KRDGITKIVNLLSDFSKFGKPPLFDQRSRRLNLLLRLFVTVLLAATVAIVSVPVVFINSCNKQNLQLNATKICG
LAAPVWLPFDYTQNPRKYFVSAMEIYCATMNYAGSGSGAFLVIGTMEHLVIRIEHLKNMFPEILNEPDKQIRE
KRLKKWIEYHLSIFEIGELMNETYKWPLSVIVLCVGILFGCIGVSTMQSVSFQNSSVFLFFGWFQSIFVLCFWGQ
RLLDSCLSIRKAVYNSKWHEMDVSFQKSVLMILIRSERPVLIHAGPFSYLSNLLVLGVLQTAYSYINLLNARSZ

>TcOr88

MTEEKQLRICLSSCFFLKWSFMWPTKSEEFRTSKGLYFRLLAFVIISGLTFTAMIVMHLLKSVEAGDYDISEDIAI
LATNTGYILMMLLYIIRQKDLESLLVDLSSFKKYQKPPKFDEVNRKLEWCTRMVFGYCVFGSVFYNLVKILAIP
SCKKSRRINEVCGVAIPYWVWFDTENWSIKLPLILHTFLVIIIVDKVTLLVSLQVLEIACNIKLRLDQLNCMLVS
CFDGDVEASRRRLNECIKYHKEIISYSEIFSKCFSIEMFTHLTTTGIICGCLENQVVQEHRPEAILHIGGWITAIFV
SSFGGQILIDSSLSVAEAAYSSAWYEADVSLRKDLILVILRAQKALFVSTGPFNVLSFALFVSIMKMSYSILTILQ
Z

>TcOr89

MKEAVLQQSKKEMHLLNLWPKGHVKHFRFRYVITLIIVSPFTLGTLTHFINVLKENLDVDLSGDISVIAVVTG
LHFMLITFVWGHKKIAYLWENLGPHEYFGKPDNFEKRCKQLNFYSRLYAYYCYLGLTVYIIMKNRGGIECRR
LNVERNLTEICGLVTTFWAPFDIDFFPFRQILFVDQVFATYFIVKGGAAISFTTLEVGEYIILKIKHLKRLVKEVF
DDPREEVQRKKLVFCIKYHQYIISIQELYDGRYKHCNGCYILMVGIIIASLSNEIMKNHNIEALLHLVGWVFSF
YICCFSGQSLLSESLTIPDAAFESKWYEAPVYMQKDLLLMMLRSQKPLMLHATPIGVMSLSLFITLVKTSYSYFT
LLNQSTZ

>TcOr90

MAKDTSPVLRESIEVMKYLQLWPQNERTNLRRRYFIVIFLCSPLHLGLATHLVVCLKDNLDVDLSANIAVLSA
VTGLTYMLIVFVWSQDKLVHLLAKLDTHEIFGTPDNLTKRSRRLNFYAKLYSYYCYFGIVIYSLVQIIEMPQCR
KMNEEKGLSEICGMIVPFWAPFDIDWFPLKQIFWLNQLLGIYIIIKGGAAVSITTFEVAQYICLKIKHLNRLLRE
AFDDPCDVVVEQKLLHCIRYQQHIIRTNELFNVCFKHCNGCYVVMVGIIIASLLNQILKEKSVGALVHFAGW
ICSFFICCHAGQAVISESLTIPEAALDSHWYEAPVKYKKVLLLLLVRSQKAFNLQATPIGIMSFDLFIALLKTSYS
YFTLLHKSTZ

>TcOr91PSE

MAKDSSPVLQESIEIMKYLQLWPQNEHTNLRRRFCFVILLCSPLTLGLAAHLIVCIKDNLDVELSANIAVLSAV
TGLTYMLIVFVWSQDKVVHLLAKLGTHEIFGTPDNFKKRSRRLDFYAKLYSYYCYFGIVIYSLLQIIEMPECRKI
NEEKGLSEICGMIVPFWAPFDIDWFPLKQIFWFDQLLGIYIIIKGGAAVSITTFEVAQYICLKIKHLNSLLRQAF
DDPHEEVIEKRLLHCIRYQQHIIRTNELFNVCYKHCNGCYVVMVGIIIASLLNQIIKEKSVGAFIHFAGWICSFF
ICCHAGHFRXSLTIPDAALDSKWYEAPIKSQKMLLLLLVRSQKAFNLHATPVGIMSFALFVALLKTSYSYFTLL
HQSTZ

>TcOr92

MKNQEIKICRATLTVLKYSLIWPSEADEMNPGKWYYIRVVTFILFTCPWVLSVFMHLIVSIRNNADIHLSEDV
ALMVAFTGVYYMTIIYVKKQPKVAFLLRDLSYFQFGKPPGFDETERILGFLSKLTFCYSVMAVVIYNYIKYRQK
PECERMNKLKGLKENCGMLTPTWWPFEINYSPAFQLIFLYIFTSTQVMMKLSLMISFNVLEMAHHIILRINHL
KTMILESLDEQDYEASKRKIKTCILYHLEILGFAERMDDCFSNGMFAHLTITAAICGCLEKQFVDGDNQLGSL
LHIFGWILALFLACLGGQHLINASETISDAIWSSKWYDADLRLRKDLIFMMARSQVGLYLNVGGFGILSYALF
LSVIKMSYSILAMLTSZ

>TcOr93

MTNLEIKICRATLKILKYSLIWPNEADEMNPGKWYYIRVATFLLITSLWVLSVFMHIVMSIIHDADVHLSEEVA
FCVAFCGLYYMTMIYVKNQPKVALLLRDLSKFQFGKPPGFEEKERILGFLSQFFFYYCVMAVMVYNLVKLLQ
KPDCEKMNEIKGLKENCGLLTPTWLPFDINYFPAFHLTFLYVFISTQILMKLALIISFNALEMAYHVILRIDHLK
IMITECLDQRNYEVSRRKLKTCILYHLEILSLSNRLNDCFSNIMFAHLTITAAICGCLEKQFVDGDNRLGALLH
VCGWISALFVACIGGQHLLNASLSIPDAIWSSKWYEADVRIRKDLLFMMAKSQVGLHLNVGSFGVLSFSVFFS
VLKMSYSILAMLTSZ

>TcOr94

MAIKICKFTRKNMQISLIWPREFEEINPGKWYYIRIVIFLITYGVFPFCTFLHAVVVIHNNLDIRISEDIGAVVSNI
GISYMAIIYVQQQNQIAYLLKDLSDFKDFGKPPFFEEENKRLNFWSICTFIYPTCGASLYNLSKILEKSECNKINE
ENGLPATCGFIFPIWVPFNINYFPLFHIMLISTWFCTTMFVRLHLSISYNAFEIAHHIILRIKHLNGMIITCFDCQ
DYKISRQKFTTCVLYYKQILDLSNRLNQSFSSIMFVHFTMTSAVCGCLEKQFVDGEYVGGFIHLVGWIISLFIAS
VGGQDLVNASQSISEAIWSSKWYLADIRLKKDVLFMLMRSQKDLHMSVGSFGVLSYAFFVSVLKMSYSILAM
LTSZ

>TcOr95FIX

MVVKESEIKVSRVTRKILQYSLIWPKEGDEINPGKWYYIRIFTFLSFTSLWCIAICMHFIIVLKDKIDWDVTEEIAI
IIAIYGTYYMVLAYVKNQKKAARILRDLSNFERFGVPPGFEEEEKRLKVYIIGIFIYAFLTITFYNFFKLSQKGAC
ERFNEEHHLDENCGLLSPVWIPFKVDRFPQFELVFLYLFTCCHLLMKLPLVVSYNALEMVHHIILRINHLKIMI
TECFDEPEYEISRRKLTQCILYHIEILEFATRVDDCFSNCMFAHLTLTGAICACLEKQIVAGISRFGAILHFIGWI
LALFIGCLGGQHFINASDTIPESIWASKWYNANLRLRKDLLLMMMRSQRDLHITAGPFGVVSYALFLSVLKM
SYSILCVLTSZ

>TcOr96PSE

MNENVKIKISSINRKVLZYSLLWPDEKIELNPGLKYKISVLGFFLITGLLVLSVTIHFCITIKVWMWKNFIAGYD
SYYTISAYLNKPTQKGNSDARDLIVFKNFEKPPNFEKQNNQLNFVAKILVLYSLLATVFYNGEQVIKKKECKK
INSEXGLSDHYCRLLAPCWLPFEIDFPVFQLVLIYVSISGYFLVKIAMHVSYNAFEIVSHYVLRTEHLKSMILETF
NENQRDICHQKFLRCVLYHIEILDFAARLDKSFFYTMFGHYFNWRNVWLIGNTNNFXGVNVVARTLHFIGW
ILALFMGCVGDNILSMXSKILPSVIWVLKZYQAXLEIKEASIFMPARSQKSLFIRAGPFGILCYSLFLTIHNTZVL
KTSYSILGVLTSZ

>TcOr97

MNNQKIQISNMTRKVLRYSLLWPKTNEELNPGIEYQFSVLGFFLVTGVLVLCITIRFFITIKAVHEVDAEVLAIL
IASYGSYYMICAHLKNQHKVALLMRDLSVFNNFGKPPNFDKRNNQLNFVAKLLALYSFLATIFYNGEQLINK
TECKRINKEKGLSDHYCGLLAPCWLPFEIDYFPVFHLILIYAFTSGYLLIKMAIHISYNAFEIVSNIVLRIEHLKA
MILETFENRNKQVCHKKFLQCILYHIEILDFAARLDDSFFNSMFGHLALTGGICACLEKQIVSGVNVVAGTLH
FIGWILALFIGCVAGQYLINASEILPSAIWTAKWYDADLELKKKVLFMLARSQKSLFIRAGPFGILCYPLFVTVL
KTSYSILCMLTSZ

>TcOr98FIX

MVKKESEIKISRVTRKLLQYSLLWPTEGEELNPGKWFYFRIFAFLSFTSLWCIAICMHFIFVMKDKPDWDPTEEI
AIIIAIYGTYYIVLAYVKNQRKAAGILRDLSNFDKFGVPPGFEEEEQRLRVYIICVFIYGFITITFYNFYKMSQKKS
CERFNIEHNLHENCGLLSPVWIPFRIDKFPRYELVFLYLLTCCHLLMKLPLIVSYNALEMVHHIILRINHLKIMI
TECFDDPDYEISRRKLTQCILYHTEILEFATRVDDCFSNCMFAHLTLTGTICACLEKQIVAGFSRFGAILHFFGW
ILALFIACLGGQQFINASDTIPEALWASKWYNADLRLRGDLLLMMMRSQRDLHITAGPFGVVSYALFVSVLK
ASYSILCVLTSZ

>TcOr99PSE

MSVGEIRICRVTRKILQYSQIWPSDNYEMNSGKWYYFRVLTLMLTTSVWFFCIIMHIIMSTRSHLDLHVSEDVA
YIVVFCGLYYITFNYVKNQHKIALLLRDLSNFDKFGVPPGYENVETKLGFYSKFLFAYVTVAGVVYNFMRFIE
KGECENMTGKDFNEVCGLMSPLWIPISLNNNSLVYCAIFLYVLICSQMVIKVGLMISYHATEMAHHIILRIDH
LKLMIVKCFDLEYNELQRRKLRQCILYHTEILDFASRLDECFSTGMFTHIFMTGAIFACIEKQFIDGEHQFCAA
VHFIGWVLTLFVACLGGQHLINASESIPESIWFSQWYKADKELRKDLLFMLMRCQKSLKIRAGPFGVLSYPLL
VSVLKMSYSMLCMLTSZ

>TcOr100

MSPKDKIKICGITRKVLRYSLLWPVENDELSPGIRYKLTILAFFSITGILVFSISVYSVLEIKQGYDIDVEDVAILIA
VYGTYYMVSAYLNNQHQIALLERDLSQFYKFGKPPGFEQLNSQLNFAVKVLIIYSFLGTFVYNGTKMLLREEC
KKNSQEKGLSDNHCGLIATFMFPFRVDYFPVFYIVLVITFLLAHTLIKLCMHISFNAYEIVNHIVLRIEHLKEMI
LSCFNERNQTIVQKKLRVCILYHIEILDMAARLDKNFFNTMFGHFALTGAICACLEKQIVLGVNIVAGTLHFI
GWIIALFVGCVAGQCLLNASEIIPNALWAAKWYHADLRTQKTLLFMLARSQKELTIKAGPFGILCFPLFVSVL
KTSYSILCMLTSZ

>TcOr102

MQNQSKPCQLDMMDETYLQFFVKSFTYLNMLPEKTTFCTTIQQYYVSVIITITTFPILADLVSQFYEESISFTSVN
ENFVALSALFAVIYVSVCFINRKHKIRALIADLALFETFSSKAVITETDKSVKFYTKLFIVYGIVGNLCYGLLPIL
GYKKCHESKSVHMTRYGIPCGLVVRFLFPFKFDYSPLAELVALYEILVCILGTSVVIVVTTLICGVLIHITVQLQC
LRKIILDLSQVNDLEILEHKMKFCVKYHTAILDYGIRTDLAFNQMMLLHITWTGFIISVLGFEISTTDDYVEAFR
FFMHLLGWLGMLFVVCYYGQKILDESLAIADAVYTFLWYKKSVIVQRYVLLILLRSQKPLTLRACGVKVMSL
ATFLGVLYSAYSYFTLLLKLKPZ

>TcOr103

MKQALKLADVLGFNPLKNDNLTKLKKYSSLICMISVVVSAILEFVSNFSALETYESAPESLVPQFQTLAKISSLL
LSQKDITELIDEIKYFWKLDQFGDFHTRKLKKIYKYVTIFFYFYTLMLSGACVLFTITTVIFTPEKPLFLCYGGLH
GLPSPQFEIYFVVDLAAIVIMSFGVAAYDGIFFYFAFHVYAEFKLVKVAFKGKSTFIEAVKHHDFLLKYLRKLN
EIYSPIFLCQFFSNLLGICFCLFMLSRSGMPPELTSFSKYFISLVAFTVQTYIFCLIGDLVSELSLDISNVIFYVDWLD
DEVYKSKTARLVIMNKAQSPVKLTIGKFTGMDLRTFLLIVRNAYSFLAFVNNALDZ

>TcOr105

MKPALKLANVLGLDPLRNDNYTQLKKMFCALCIVSLFVSAYLEFFSNFTTFETYETAPESLIPHFQTMFKMYS
LIFSRTEIVELIQMAEQFYKFSQCDERKKLTKLYKRVDLFFYVYASLVAAACVLFAIVTLIFKPGKPIFLCYGGL
HGLESPEFEIYLVVDLIGIVIISVTVPAFDGLFFYFALYIYTEFKLLKIAFKTMSGQELREAVKHHDFLLKYIKKLN
SVYSPIFLYQFFCNLLAICFCLFMLSRSGIPPEMVSFSKYFLCLLAFLVQSYTFCSIGDLITELSEDVSNAIFYTDWL
DDEAYENKTARLIIMSRAQNPVMLTIGKFANMNLRTFILIVRNAYSFLAFVNHALNZ

>TcOr106

MESALKLIDIIGLHPLKSDKYSTMRKTISFLSLVVILISAQLEFLSHLSVFEVYNSGPHSTIPPLQSLLKMATLHFY
KNELIDLMEKSKSFWKLDKFGDLYKQELSKLHRLVTIIVYIYIALLTATCVQLAVLTLIFRRGKPIFLCYGGLYG
LESPHYEIYSILDAIGIGVISIAVSGYDAMFFFFALDIYTEFKMIKSAFKRHSDQTVSSYNKQFIEAVKHHDFLLQ

YINQVNDIFSPMFLFQFFSGLLGICFSLFMISRSGLQDINTLSIYSAGLLGFTAQSYTFCLVGEVISELSEDISNEIFY
TDWLDDEVYRNKTAILIVMNRAQESPKLTIGKFADMNLRTFIMIVRNAYSFLAFINNALDZ

>TcOr107

MENPLKLLHIIGLDPRQSDKYSTIKKVISFLIVLAVLLSALIEFFLHHNESQVYDTAPQSTVPNLQALLKMFALII
YKKELIDLFTKGNHFWKLDKFGDCHKQKLTKLHKYVDLFFYVYAVIITGAFLQLALLILIFEPGKPIFLCYGGL
YGLESPQFEFYAVLDFLAIGVIAISVTAYDSIFFYFALYIYTEFKMIKIAFKRENCAQFIEAVKHHDFLLQYISKV
NEVFSVIFLTQFFSGLLGICFNLFMISTQGTRDMKSFSTYFVGLVGYTAQSFTFCLIGELISELSEDISNEIFYTDW
LDDEVYRNTTARLIVMNRAQESPKLTIGKFADMNLRTFIIILRNAYSFLAFINEVLDZ

>TcOr108

MGSILLLNSVLKKMEKALKLVNILGLDPRKNDTFSKFRSIFCFTILISASFSSHLEFFLNFKGLETCERAAESIIPQ
YQTMCKMATFLLYKTEMLDLIKKSERFWKLDRFGDLQAKNLHSTYPIFQIFFYVYVVILFLTCAMFALVNWIF
DTGKPISLCYGESEGLETPWVEFYIVLQSVEVTIIFLGITGYDMVFLYYAGSVCIQFQMLKMAFAERKMNERQF
LKAVKHHEFLLQYVEQLGDIYSMWFLLQYFSSLFGICFGLFLISKEGLPTEPERLSKYFPYIFSFTMQSFTFCMTG
TMLSDWSSEISDEIFHSDWSDDQVYKNKTARLIVMNRAQRPAKISIGKFLDLNLRSFILLMRSVFSFLAFVNNI
LNRINZ

>TcOr109

MGKVKFTEPLEFLNVVGLNPENCSNFSLFRRVISLGFFLVVITLGLLELLLHFEGLETCSRASEAMIVQYQLFIKI
AVLLKHRKNLVVLMQKTRKFWPLDKFGQDAKIERPHKLLKAFFFAYKLIMILMALQYILRKFVSKNGKPLAI
AFGESKGLSPKVDHLYFVLHSTSTFVVLHAVTGFDRLFFFLIGHVLTELKLVKKSYRLTQNRREKFLETVQHH
AFALEFVRKLNRIYSQVLLNQHLSCLFGICFGLFLVSKDGIPPDLGHVTKYVPYVISFITQTFTFCFIGSLLITWSL
QVPDAIFYNDWGKNQAYKYKTDKIIAMIRGQRAAKLTLGGFGDLDLESFNLVVKNAFSFFTFVNAMNQKZ

>TcOr110

MDKVEFSDPLFFLNVIGMHPFKADKFSKFRLAFSIAVYFAVIFSGVLELIVNSQGLETYARASDTLIPQCQLVC
KIFVLAKYKKQIARLLNGSQRFWDLGQFGARYGNSFGKTHKYLKSFFLLYKVMLTFTCLQFLAVKIIFKIPKPI
AISFGETKGLEPLYDHLYLVLHAMITLVTINLVNGFDGLFFYFIGHVLTELKMVKVAFGDSPIETNWSEEKRFK
FAVRHHRFVLDFIEQFNIVYCTMLLVQHLTCLFGICFGVFLMTKDGVPPDLDRASKYLPYIVTFIFQTFTFCFA
GNLLLSWSLEIPNEIFYHDWAKKTTYENKLAKIISMKRGQRAARLTLGGFANLDLDSFRMVLKNALSFFTFV
NAMMNKKAVTSVZ

>TcOr111

MEKVRLTEPLFLLHIVGMSPHDSGTFARIRKIFSILVYTSTVVLSMAELFFNYKDLETVIRATESFFTQYGLAWKI
AVFVVYKTELAQIIRLCDNLWPLDEFGTGHNFQFLHKFLRRFFLLYTGNLALLCTQFAVTAFFDDQFKSVMV
YYGEKESRSQIYDNFVFTLQVIYLYVGCFVVAGFDCFFFYLLGHAVTELKMLTISFSCKEIGRNWGYEERFKCS
VKHHIHVLELLDKINKVYSVMLLNQHLCSLFGICFGIFLMTKDGIPPNVDHFSKWSTYIFTFILQVWTYCFAG
DQIMHWSLKIPDEIFYDNYWNKYSLKNGLNKIIAIQRGQKAAGVSLGGFAMLDIESFNVVIKNAVNFFMFM
DKMYKREZ

>TcOr112

MITRLMAQFAIKGRVGTGGYIMDKVKLAQPLAHLNIIGLDPLKNDRFSKIRTVITVAVFALCNVFSFSELFLHY
NNPHVIVRSSEVVFPFFQNDWKIAIMLVYKKNLAQLIQNTSRFWQIDAFGKNYQYSMGIKHKYVRIFYLVYR
LMLMFSCSQYILLTIGSDRPMILSFGETGGLGSGALLFYLIFHIVYLLIIFNVINGFDGLFFFLVAHVLSELQMVK
VAFSSSKVITFWNHKRRFKSAIQHHRFVLDYINRLNSIYSILLLNQHISCLFGICFGLYLFISDGFPPDYEHISKYV
PYVIYYITQVWVFCFAGQLIIDWSVNISDEIFYHDWTLNRTYENKTDKLIIIQRAQHAARLSLAGYGNLDLQSF
NLVLKNGLSFFTFVNAVIHKZ

>TcOr113

MLDAWERLTFYPFKWISLGGLHPQNDRLVAKFLFLYNFAGFGTILGLAITQIYLSYENIYYTIDSILTIVLYLHIA
SKYVNLHLHKDTLAMLIQERSKFWPIDTFEMTVRQKCVRILTKSLTIIKSYLGYSLLVVISFLIQPIITGQLPVFM
YVPRGTYYIFFVIFMTITPGIMSSIWGVDTLFFSITTPVSIQFKLLAHKFETIDLKMDSKRVRHEFRKLVDYHNFLI
NYCQNINRMSSGIFLTQYLVAIATSCMQLFITSQPEFGLLNKIKCLTYFIMQIIETGIYCFTAQLISESSENVGNA
VYKAPWYDFNCGTRRDIALVIVRSQKKVVFNGLGLVWIKMETFTKIFKTALSFYTYLNTMVYQNZ

>TcOr114

MNDPSYYSLKLLKVCGLHPNSNNKLLNVYFVVNYSCFLAILTLAIIGISKTYDSNVFEAIENLQTVFLYIHLLG
KYPTLFFKKKTLKELLERQKQFWPIDNVEPKLAKKFDQILTNTTKFIRYFIIVTFLVIMNFFLQPILTGDLPVRVY
VPSGWFHYINSVYWYLIPVIIGSIYGSDLIFCSLCVPVIIQFQLLAHKFEKFKPQQLKKLVDYHNFLIKYCNDLN
KYIEPIFLNQVIVATAIICMQLFIVSQKEFLLPNKLKCLGYCFGEVIETAIYCFNAEMISDAAEKVGIAVYNSQW
YKVPRKSVVLVIAKTQKKVVFNGLGLVAINLKTFTQIFKTALSFYSYLNTMVAFEKNZ

>TcOr115

MSNKQLDPTYYILKIFKAAGVHPDVKVTPFLVFFFWMNCTIVSAVIVLATIGAIFGALDNDINTVVECLQSTFI
YIHILGKHFVLFYSKPILSHLLAQRTHFLQLETFDLSTIEQFQQLLKKTSKFVNTFMFCTSCVVFSFYMQPYLTH
GDLPVSVYVPDGWYYYIHFGFWPLAPCIVASIYGSDALFCAISVPVIVQFRLLARKIQNWKIENTKLNNQKSR
KIFKKNLKELVDHQNFLFEYCNEMNKFNNGIFLNQFLLSVGIICVQLFVVSQKGFKLPNKIKCVGYSFMEIIET
AIFCFNAELISDASEDVGNAAYDSLWYESDDPEVRHAITLIIARSQNRIVFSGFGFVWINLKTFTQIFKTALSFYS
YLHNVVLNZ

>TcOr116

MPETLSFYPELMLKSAGLHPYTKLKIVKFFYHHYLNLFFFIFLLFLAILEVGVSVKCDIYRAIEALSSVLFMTLTL
FRYVVNYRNKPSLAWLLEKRSNFWLLEHFEGQIRTDCAKIMHTSSNFIRNYKNYAIVLAAVFYVQPFIFHELQ
MKIYVPEGWFYYLYLVYWYMTPPLFVSVYGVTSMFCAICIPVTIQFKLLAHRIQNLDFKSEKFQRDLKHLVDY
HNFLIDYCTRINRYSNGVLLFEFFITISVCCILIFIAANDYPFVDKIKYAGFIVSQFLDTAIFCYNCELISDASENV
GKAAYDSLWYESESKIRRSLILIIVRAQKKVTFSGYGLVRINMMTFTQVFKATLSFTSYLNTVTVDEKMLNNZ

>TcOr117

MDKNLDPDDLSAYPLKFLWYGRLHPGLNPWWTKILVPVNVSVAFLYLVLAIKGIFSSYNHDTFFTAECVQTC
ILVVHAIGKFSNFLVHKNSLLRLVAKKSQFWKLESFDGDLYNECVWISTFVKKITRFYYFLNLFVLISFDLQPFT
TGYLPTGCYVPEGWFNFLTGLLWYLSCAVLFGLPGTDGFFCSLATSLIIQFKLLGYKFKNTKLYKNEPDITLW
NNLKQLVDYHNYLLSYSKELDATFKTIFLLQFMISIGSASVSVFIFMQPGDWSNRIKFLLYFVATMVQTAFYCI
PLEFVVSSAKQIGDFVYESNWYQVKDIKFEKCFTLILARTQKNVVFSAYGLIWINLGTFLVICKTVFSFYTYLNS
VNKITSZ

>TcOr119

MLMSLEPDDISADSLKILWYTGLHPALSSRLINIYIYANLFLCSVLTILAIIGIVLSYTNNIFFVAECLVTIILMVH
AIGKFIALHWNKKSLINLLKKKSQFWKIGSFDGEIHNECLQISTFVKNIIRFYYALSFCGGVFFDLQPFTSGLMP
SGCYVPEGWSNILMGVMWYITFPVVFVVTGTDALFCSLSTSLIIQFKLLNHKFKTLKLTNKSQTQLWNDLKEL
VDYHNFLLSYCEELDATFSGVFLLQFIISIAPASVSIFIFMQPGAWANRMKFITYFLAVITETTFYCLPLDIVVNT
ASQVSDAIYESKWYEVDVLHFKKCLTLVIARAQKSVRFTGFGMVYINLRTFLIIWKTVFTFYTYLNSAKKITKZ

>TcOr120

MAKKFLLDISADSLRLLWLGQMHPLSPFRRFVTFLILNLAACWLMIALAIKGITISYKSDIFFVAECLQTCNLM
FHGVGKFLNLYFQKNNLKSLLENRSKFWQLDDFRSEKLYSQMQGITFVIKKVLRYYYLLVLCVVFLFDLQPFA
TGLLPTGCYLPEGWFKGLTLTLWFLSVSFFLNIQGTNGFFYSQSVSLIVQFKLLSHRFKTTQFDKKELKELVDY

HNFLTSYCKQLNQAFAAIFLLQFFTSITSASLSIFIFMQPGAWTNRIKFILYYSYTLVETSFYCIPAEILVNAASEI
GNSVYDLDWHKIRINRVKKCIVIILARTQKTMVFTGYGLVNMNLQTFVVYVKTVFSFYTYLNSVRKIZ

>TcOr121

MPKKFRSDDISADSLRLLWLGHMHPFFPFRRSFAFLIINLTACFLMIALAIKGITISYNNDIFFVAECLQTCNLM
LHGVGKFLNLYFHRNGLQALLENRSKFWKIDDFKCENIYEDLSGITSTVKRGLRYYYCGALVVIFLFDLQPFA
TGLLPTGCYVPEGWFKGLTLTLWLLSISFFLIIQSTDGFFCSLSVAIVIQFKLLSHRFKNMHLLYAESERKMWKE
LKGLVDYHNFLTNYCKQLNAAFAPIFLLQFLVSIVSASVSIFIFMQPGAWSNRIKFVLYYLAIMVETSFYCVPA
EIIVNAASEIGNAVSDLDWYKIKINKVKKCFIIILARTQKTMVFTGYGLVNMNLQTFVIYVMTVFSFYTYLNSV
RKIZ

>TcOr122PSE

MRKKFMYNDSDGIAAVPLMLFRWGNMHPSSPFRPSVISLIINVCVCGFLIALAIKGIIVSYNNDIFFTVECLQT
CNLMFHGIGKFLNLFFHRANLKILVESRSQFWKIDDFKGEDIYDEMEKIKTVMGKGLIYYYVAVISVGLLFDL
QPFLTGALPSGCYTPEGWFKGITLLTWLLTFSFLNGVQGTDGFYFSISISIVIQFKLLTHRFKNIRLFYNEHEREM
WZELKELVDYHNFLMNYCNLLNKIFSAVFLLQFFVSISSASLSIFIFMQPGPWSNRIKFMLYYMALIVETSLYCV
PSELIVNAASEIGNVIYDLDWYKIKINQFKKCLVINVKTVFSFYTYINSVRKIEKZ

>TcOr123

MAKKFRSDDISADPLRLLWLGQMHPFSPFRRSVAFLVMNVSACWLLAALAIKGIITSYKNDIFFVAECLQTC
NLMFHGIGKFVNLFLHRDNLKKLLKNRSKFWKIDDFQSEEIYQELSEITSTVKKGLRYYYCGVVVVMLLFDLQ
PFATGSLPSGCYVPEGWFKSLTVMTWLLSLSFLNGVQGMDGFFCSISISIVIQFKMLTHRFKNMRLFHNESERK
MWKELKELVDYHNFLTRYCKLLNTIFASIFLLQFLVSIISASVSIFIFMQPGAWSNRIKFILYYLAVVAETSFYCV
PAEIIVNSASEIGYAVSELDWYKIRINQIKKCFVIILARTQRTMVFTGYGLVNMNLQTFVVYVKTVFSFYTYINS
VRKIEKZ

>TcOr124

MFFEDEDIIEFPVKLLRISRFHPTLNPLWSRILWPLNLITSSVFIILAIKGIVMSIHDDLFFMSECIQTIILMLHGITK
FTNLHLYKSDMARLLSEHRKFWKIKNFKKSGDVYLECKRMDNIIKKIVRSYCVLCIITAIFFDLLPFSTGSLPTG
CYVPEGWFKYLTVILWFVSCFFCVIIMGTDCFFCSLGTSLTIQFKLLAYKFQNIHVFIRKSEEELWKEFTMLVDY
HNFLMRYCGTMNDAFKNVFLLQFLMSIASASVSVFIFVQPGDWTNRTKFLLYFVVIISESSYYCVPSEIIKNSAS
ELSKSLYESKWYNTDVSQFKKCIVLVIARAQKTINFSGYGLVYINLQTFIVICKTVFTFYTYLNSARKISSEZ

>TcOr126FIX

MYNNKDSLLYDPLRFFGFIGFHPIFNSLILKISFYSTTTLGLFIYTMAIIGIVKPEETNSFFTLECLQTCILLSHTIGK
QVNYYMNSNKIVKFLQMTTEFWEFETFQGTIHPESNFLFHTVRKMIRYYFLVTTFGFIFFLINPPVCYVPEGWE
LFLRIVRALTFWSYYTSTMATDAFFVACGTLLLIQFKLLGHKFKNLDTQSQEKWNNLRQNVKHQIFLHSCKL
LNQIFAVVFLIQFLNSIAALAISIFIFSKPGSWNNRFKTLFYLVVVLFENAFYCVPAELVSAEALKICDQLFASK
WYESNVAQFRKSLVIVLCCTQKVIKFSSFGLVEMNLQTFVLISKTALSFYAFLNQLKRZ

>TcOr127

MSPRTLEMIADLPKDIITDSLKMLRLGRHHPTGSIWWTIFFIPVNTFFCSLLIILSIVGIVRYHEDDVFLAVDCLG
TCTLMLHAISKQIFLHAQKNAINVLLKMKSQFWNLDDFDGEISKECEMILTSGKIAVRIYFSFTCAAATFYFLQ
PFTAHHLPSDCYVPEGWFPFLAISYMYLIPTLVPSVVGLDALFWALGLSLAVQFKLLAQKFKLLGTCHENETA
ILWNQLKELINYHRFLIDFCKKLNKLFSFIFFVQSFITITSASVAVFIVMQPGNLSTRVKCLLTFVSYILEMAFYCL
PAEMSVNAAIDVADSVYNSKWFRIKSTEFKKCLILIIGRAQIPFTFSGFGLIHINIRMFQLVCKTTFTFYTYLNTV
QNRQZ

>TcOr128

MEQQRAIIETSLTFFKVLGIHPLKTLRKIPVLCLISSHLILFALVCLRFFYEKITFGLISDTFESTFTIVHVLAKFITL
VIIKPTIKKLFETTNTFWLGKTFQKEFRHEVDADLRTLGLLLRLFLFFVSIVIVTFAIRPIFDDTLAIHCYVPEFIPR
AIFIVFNNAVFAVSAFAVGSFDMFVCVMVVLITVQFKILNYSILGLDLGKVVTENDEKLCQKKIKIIVDYHDFL
DRYLAKVSSIVSPALLVYFTIAPIVLCFELFFMSKSSNFAEIAKSCVYILGIVIELFFFFCLPATYLMVQTQLMITTV
YNSGWENCYFLSVRKSLIIMMQRMQKETLLTAGKMIKINLETCTNAFRMALSFYTSLQILDDKSREZ

>TcOr129

MTDSKILLFPLKLYFLFGYHPDCSPKFRKLALLAGLWMYAFPFYLSIKGILFYRKTDFLLMLECLEAAFLFGEPL
FRHLAIHYHVSNLKNVLNLRQKAQIDGLEGESLFYYNLGKTFTFNYMIAGFVVLFGFCVQPFISGKLPATTYL
PEGYFVTFCIYYTLSGCYVVVTVVTTDALFCSLCTTAIVNFKILKRKIRNIKRTNLRHEVKKIVDQQNFLLRYCE
ALGEMYSDIFVVYFCFSIGAICMQTYISTNDQLESAIRIKTSIYAVGLFAQSILYSISAENVLSAASEIGDAAYDSP
WYRFSDAQYTKSLILVISKAQRKVIFSGCGLVTINFTTLTVIIKTAVSFYAYLNSLGSGZ

>TcOr130

MSSKNYLSFPTGFLLGTGLLPESKFSRKIVSIGVFTPMTLFLVYLIVKKARNGENKDVLLWAELFESLTTCAHIL
SRKYVMYVHGGLISQIIKERGCYFWNYDIFGPKFGQTLERKMNICTKIVKIVVSGGVVTIVLFCLTTVFDKSKV
VPLVCWTPEDSLQTGIIYAMEVLIMFEIMWALLSIDCFYLLICTDLRIQFILLQRMIKSIKFGSNHDEKSFAKMV
HCTQHHKFLLHFHAKLNTIFSSYFIVLYLVTVACASMHTYIILSPGFGDSIKSACYLSGMLFQVGLYFVITSNVE
IEAENLSVEIYNLNWENTGSVKIRKHVLFMLMKSQEELSVTGGGMLHVKRNEYVGLVRLAYTIATILGGMTZ

>TcOr131

MEYLKYCQTYIRGCGLASNSSPFRKILAIYFFLPPCFLIIAFSVYELWDANNNDIFLVIEVLECISSYTQLTIRKYM
IFTQNELMVEIINDCDQLWSFDTCGPELGKKFKQRMKNCWIMVKALVTCGFTTFILMCISARADRDNLLPFL
CWIPDFPYATEVLFLSQFMLLMELLYYVMATDGFYLLVCMDIHIQFEMMQEMLKTIQFDVISEKESWEKLTEL
AKHHNRMLHQKLNQVFSKYYVVQYFMTVAAMTVQTYTLKYRMVNIQTALKSIMYTFSLMFQSAYYLFPAS
NIEIEAENFSTEIYFLNWQDHEDVKIRKHILFMLMKSQENLELMGEGMVHINRNEYLLMFRLAFTIATLLDGL
NQLZ

>TcOr133

MEYLKYCQTYIKGTGLASNSSLFRNILAKYFFLPPCFLIIAFSIHELWDTNNNDVSLVTEVLECVASYTQLIIRKY
IVFTQSDLMVEIINDCGKLWPFDMFGSELGKKFKQQMKTCWTLVKFLVVCGFATFFLMCISARAAERDNSLP
FLCWVPDFPYATELLFFLQFMLLLELLYYVLATDAFYILICMDIQIQFEMMGKMLKSIKFGEISEKECWDKLVE
LAKQHDRMLHQKLNQVYSKYYVVQYFMTVGSMTVQAYNLKYRMVNIQTALKSIVYTFSLMFQCGYYFFPA
SNIEIEAENFSTEIYFLNWQNIGNIKIRKHILFMLMKSQENLAMMGEGMVHVNRNECLMMFRLAFTIATLLD
GLNQVZ

>TcOr135

MEYLRYCQSFVVGTGLVTNSPSFRKFLGWCFLLPLSLIVFAFSIYKIRDTDNDIFLMIEVMESISSFTQLMIRKYII
FIQGELMLEIFNECENLWSFDLFGPQLSEKFKQQMKNCWTLAKVLITSGFITIVLMCISALTDKTKSLPFICWV
PNFSYAHELIFLSQFILLIELLYYVVATDGFYLLICMDIQIQYKMMGKMLKSVQFGVISEEESWEKLVELANHH
NKMLHEKLNKVFSKYYIIQYAISVSAMSAQVYTLMYSKVVIETALKSICYTISLLLQVAYYFFPASNMEIEAEKF
STKIYFLNWQDNADAKIRKHILFMLLKSQKSLEMWGEGMLHINRNEYLLIFRLGFTIATLLSGFKZ

>TcOr136

MEYLKYCQSYIIGSGLASNSPVFRQFLARYLFFPLSLILVSLSIYIIKDANNDIYFLTEVMESLASYTQLLIRKYMIF
TQSKLMVEIINDCENLWSLELFGPELGKKFKQQMKNCWTFVNVLVTSGFSTVLLICITTLTDKEKSLPFVCWIP
GFPHATELIFLTQFVLLMNGLYYIKLTDAFYLLVCMDIQIQFKMMGKMLKTIHFGLLSEKESWEKLVELAKH
HNKILHKKLNKVYSKYYVVQYVMSVTAMTAQAYTLKYIEVNIQLALKSIMYTCSLLLQGALYFFPASNIEIEA
ENFSTEIYFLNWQDHGDVKIRKHILFMLLKSQENLEMMGEGMMHINRNEYLMMFRLSFTIATLLGGLNQLZ

>TcOr138

MGASIHYFNYFYGHSQSSKFPTSSKSLKARIFNYVLYPISCTILAFMLYNFRYMNDNIFKIARNCQSITSFGQIFT
RHFIYAQHSFLLENLLKNTAKNWKYDPKNPLFGHKTWTRVILVKSLKIMSFFVVTFIIILPFFCQDIDLPQACW
VPGSGHKMQLLIYFLQSVCLLEPMILLDMVDSVFLLTGVELEIQFILLRKAIKNVQISEQENQNFCMGKLKLY
ASYHQFLLDQHTVLKQAFSSFFFLQYLVSVQGLCVEIFVIKQASTIEQIFFSATYIVANAMFLSFAFLAASYLEIE
AEALPHAIYSIDWYNGDEKLGKHVLFMLMRAQKPLILTGAGMFAVTRNALLQVYRLAFSISTLLKQVZ

>TcOr139

MSDHFRYVSTIFKLSGYLPSLKTLKTKILKYVFSPIMFTLITFIIYNFRYMHHDIFEIARTCEALSTHGHVFGRKL
AVLKHANLIEQVINDRNFFWSYEKFGEKLGQRFRQKLFFRDYVMKSLSAMSVLMLAYFYLTPVFVRSVNLPQ
ASWVPQFSHATALVYFCQVVCLSESIPVAVIIDGTFLLMGAELEIQFNLLKKTLKAVEIGQNSGQKHEEKCLK
QLKICASYHDFLLKEHVKMKTIFSEFFLLQYLLSIEGLCIELFVVNKAQTWGQFALGAIYVVGIIMQSSFTFLSA
SNLEIEAESLADSIYDVDWYNAKDPRIRKHILFMLMKAQEPLRLTGAGLFDVNRNMLLQIMRTGFSISTIMRQ
FZ

>TcOr141

MAFDASKNNYLEMCLILYDLSGLRASSKPFLKFIALYTLYPLMLIVCAMIHVNIWFKHGNIFEITEVFTSICIIAS
ITIRKTVLIYYGPLYEDVIQQHSQFWDYGLFGKTTELRLRKNMKFCVFLIKCFLTSGIASIVVRSISPLFVKDILLP
QECWIPGNNPVALKVIYVLEILFYLESTTYFPLFDGLYLIMTGNLKSQLILLQKAVESVDLVRDDDEISWRKLK
KCCQHHKLLLSVLKKINKIYSVFFLCTYVLTIIGICLPLFVIFNKSSTFTQVVESVLVANIMNTLLIMICIPGSEIEI
EAERLITQIYNINWHETSNLKIRKFVLFWLMQAQVPLRITGGGMLIVNRSLMFQIQRIAYSVSTLLTGLTSZ

>TcOr142PSE

TELFGIILTSRSRVVQMTFDVSQHNYLHLCLMKTTELRLCFLLLNSFTISGKHLTIVYEXSSWIPGNNSLVDFTLF
YNENMTHTSLFVGLKLVNFKLQLIFLQKAIESIDLNSLGKEASCRIVKGCCQHHKFTLRYKYYIDFSSVYFHYT
KVNSYFFVCTYLFTIIQICTIXYFNFSNIFIGHLISLKLYKVYILVVTEIKIEAYKLITQVYNLIWYETKVZNLQVYP
VKEEZRIKERVVFAVNRTIILQRIAVSVSTLLTRLEZ

>TcOr144

MAFNESQDNFKLCFIAFNLSGLGPSSKPFLKILSYVLYPWLCLLFVLVCVNIVFKHSNIWDIGEVTSSISIAVMM
VVRKTILIKYSSVFAEIIELHSRFWDYGLFGKATETKIRKKVDFFKFILKCYIVSGITATSTRSIVPIFDKNLTMPQ
DCWIPGNNSIVKHIIYAFQVIFYAESISYFTFFDGFLLIVTANLQAQFILLQKATGSINFETDSEETAWKKLVKCC
EHHKFLISVHKKLNTLYSYFYLVTYFLVITMGCVSLFVIFDKSSTFAQLLESAITMVVLNVMIAMICICSSEIEIEA
EKLLTQIYEVNWYETPNLKIRKFILFWLMQAQVSVETKGAGLLVVNRSLMLQVQRFSYSVSTLLKGMNEZ

>TcOr145

MSEDNYFRMVLNCYNLSGLRKSSKWFLRVLSVYILYPIMLIIFGMIIYNVRFNHSNIFEITEVFISICMFLHMVLR
KTMLIKNSSLYEEILELQFYFWKYGLFGETTGSKLRKSMKTCVSVLKFIVINGSMSVTLHSISPLLVKSLVLPQSC
WIPGNNPVAKNVIHIFLVIMYVECLNYLVVFDGLYLLTATNLKAQFILLQKAADSIKLTRGEEELSWMKLKKF
SQYHAFILSVHKKVNKIYSEFFLCTYILTIWGTCIPLFIIFDRTSSVTEIIESTFVGFIINILLVMMFVPASSIEIEAEK
LAVHIYNINWYETKNVKIRKFILFWLMQAQVPVRMTGGGMLTINRTLMLQIQRIGFSLSTLLTGLARZ

>TcOr146

MKYIREVENIKAGIFSSCGIVAEEQRVEMSEKNQQDYFKIPDKCYNWSGVRVSSNALTKFVSLYILYPLMLILY
FMIIYNIRFKKNISDITEVFISISTFTIITFRKTLLIRNGSIYEDILKKQSHYWKYYMFGKPTEMKLRKSMEFCVMVI
KFLIASTTGSIIFHSIISPIIEGKIVLPQPCWVPNNDPVANDIIFALENIFYMEGTNYLVVFDGLYLLMTANLKTQ
MILLRKAVASINFREDEKTTWAKLKEYCEYHKFLLRIHGKINKIYSAFFLMTYVFTIMGTCTPLFVIFYEEADM

VLLGKSVFIALILNTLLVMTFIPAGELEIEAEKLSFEIYSINWYETKNLKIRKFVLFWIMQTQIPVQMSGGGMLIV
NRPLVLQVQRIAYSLTSFLAGLSZ

>TcOr147

MSFNESEDNLKLCLACYNLSGLGPSSKLFLRLLSYILYILLWVFLIMVIINIAFKHDNIWDIAEIFTSVSITITMVIR
KTIMLKHSSSFEELIEMHSQFWDYSLFGKTTESKIRKSTGLYKFILKCYVVCGVISVSLRSLAPIFAKNLVLPQDC
WIPGNSTIARFIIYAFETVLYAEGVSYYTFFDGLLLMITANLKAQFILLQKAIQSINFETDSSETAWVKLVKCCE
HHKFLLSVLKKLNSLYSIFYLCTYILVITGVCVSLFIVFDKSSTFAQIVEGAITVVILNSMIFMICICSSETEIQAEKL
ITQIYDINWYDSPNLKIRKFILFWLMQAQVSVQIKGAGVLVLNRPLMLQVQRISYSVSTLLTGLNEZ

>TcOr148

MESKSLRSYFDTSLKCYHLYGLTTNSSRIRRFFTTYVLYPLMLSLYAMVLYNLRFKHHHIFEFAEVSVSATTFG
NILIRKSLVVFSGSLNENVIDKHDQFWKYDSFSKTIAARCYRSMDLCQMLINFIMIGTTISIVVHCSLPLFLKDL
LLPQSSWIPGNSSIARIVLYIMEIIVYIECLILMEMFDGLYLLMTVNLKVQFMLLRKAIESINVEKEDDEKCWQK
MKDYCKYHKFLLSMHKTINKMYSQFFLYQYLLTIWGTCTTLFVIYNKSSTLAQITESVFIGSIINTLLIIIFIPASEI
EIEAEKVAFAIYGIDWYNSKSLRIQKFVLFWLMHAQIPVQMSGAGMLNITRSQMLQIQRIGYSLSTLLSKLSZ

>TcOr149

MMRNEGETNYFQMCLTCYNLSGLRSSSKPFLKIISQFILYPVMWVLFGMMTYNIRFKHNNLSEITEVFIAVCTT
AYILLRKTILIKNSALYEDLIKEQSRFWKYDLFGEPTESKLRKNMQFCVSLIKLIVISGIMSTVVHCSSPFLVENID
LPQSCWIPGNNSIVKNIIYVAESAIHIECLNYLAVFDGLYLLTTTNLKAQFVLLQKAIESITFKSGDEETYAWTQ
LKACSQYHIYLLSRIHKKINKIYSEFFLCTYILTIWGTCVPLFVIFNNISNFAEVVESMFIAFLINALLVMMFIPAS
EIEIEAEKLALQIYFINWYETKNLKIRKFILFWLMQAQVPVRMTGGGMLIINRSLMLQIQRIGFSLTTLLTGLTT
Z

>TcOr150

MAFDASQHNYLHLCLILYDLSGLRVSSNSFLKFLSLYVLYPLLLIMFVMVHLNVWFKHANIFEITEVFTSICIV
ASMCIRKTVLIQYGSTFEDVIQKHSQFWDYGLFGTKTESRLRKNMEFCFLLLKCFIISGIASIIVRCFSPLFMKEL
LLPQDCWIPGNQPVAKKIIYVLQIIFYIESMTYTPLFDGLYIIMTGNLKSQLILLQKAIESIDLKRQDDETSWRRV
KECCQHHKFLLRSILKKINKMYSNFFVCTYLLTIIGICIPLFVIFDKSSNLTQIVESILVAIVMNTLLIMICIPGSEIE
IEADRLITQIYNLNWYETRSLKIRKFILFWLMQVQVPVQIKGGGVLAVNRALILQIQRIAYSASTLLTGLTSZ

>TcOr151

MPQAQKDAFYFVIDCYNKSGLRKTSPNLRKLVSIYLLLPLLFILYSMIIINIRYKNANIFEIAEVFEAVSTFGQLV
ARKSLLLLHRSLFEEIIQDRSHFWSYDSFGGKIGDKYRQEMQFSVSLIKKMWYASVWSLVFHFCTPFFVTNAV
LPDACWIPVDSKCLVIFLYALEMVFYIEVIFLLGSFDAFFLCMCTELKIQFNLLNRTLDKLQTNPETTDEMWLK
TLKKCSRHHCFILKVHAKLNRFFSEYFVCQYLINVGGVCIQFFIISNESSNLTQILRSVVYILIASLLGALIFLPSSA
IEEEAEKFENRIYDIEWQNTKDLNIRKFIIFWLMKAQKSIHMTGAGLFQVNRNVLIQIPRLAFSIATILSNVKZ

>TcOr152

MEYLKYCQTYIKGSGLAPDSPPIRKFLAKFFILPCFLIIALSIYKLRDSNSDIFLVIDVLECVASYTQLTIRKYIIYN
HGKLMEEIISDCENLWSFDMFGPELGKNFDQKMRNTWTVVKSLITCGFVTFILMCVSATTSKENPLPFTCWV
PNFAFATELLFLLQFLLLMELLYDVMAMDGFFLLVCMDIQIQFKMMKKMLHSIQFGVTSEEESWDKLVELVK
QHNKMLHQKLNRVFSRYFLVQYAMTVGSMTVQAYTLKYSEANIETTIKSIVYTISLMLQGASFFFSASNIEIEA
GNFSKEIFLNWEDQQDVKIKKHILFMLMKSQENLELTGEGMVHVNRRDYLKMFRLAFTIATLLDGLSHFZ

>TcOr153

MAPNKSSKKDFLDMCRKYMHGSGLGPTSPKIKRFISLWLLFPVSLLLDVLVIYDFHFLDNDIFKTAELLESVSS
FGQLPIRKFILTYHSKLIQNLLEDRKKFWSYEMFGETYGKFLRRKMVLATRLIQTMIFFGASVATLMFVSTLAD

DRKTVPLECWIPEFKHSTHVVLVMQFCSLCEIIYLVGAVDCLYVLTCVDIKIQFLLLQKKLKTIQVGVKPMEEC
LNELTICVKHHNLLLRSHKSLNRIFSEYFFVQYFVSVLAACVQLYILMYITASLEDIMKSIVYLSAVVFQVAIFF
MPASDIEEEAEQFAVEIYNVNWECTSGTKFRKQLLFMLMKAQKPLYMLGGGMIHANRNEYIVLFRLAFSIST
LLGGMNENGRTDKZ

>TcOr154

MSNNDETDYFKIPLKFYNWSGVRITSKKIPKIISVYILYPMMIVLYGLMIINIRFNIDSLAKFIEVGISVSTMTIIAL
RKTLLIKNGSVYEDLLETQKLYWAYNRFGQAFESTQRKRMYFCLLVTKFIIGYSCVSLSYHSIIAPIIMHEMILP
QPCWNPGNTTIGRNIIFFVENIFYIEGTSNFVIFDCLYFLLATNLRIQFALLRKELNSIDFKEDSEEKCFARLVKC
SQYHKLLLSVHRKINTIYSAFFLLTYVFTITSTCTLMFVVFYMEADAALLGKSIFIVIILNTLLVMTFIPAGELEIE
GEKLAMDIYFMNWYETSSLKIRKFILFWLMRAQIPLQMTGGGMLVVNRPLVLQAERVSYSLASFLANLZ

>TcOr155

MTEPLYNPFAYTILLLKILGMWKYKTESNFYKCYKHLMSASILLTCVFSLLYAYKNYKDPEAIFFIAYLPATLT
VPMKFVMFPLNLAKIKRLLDILGPERAIIRTQKQKVILENSLQLSRQIYRIFTACFVSASAGIVATPLSMRRQILL
FLEWLPVDYSKDCVHYGVFVGLLYFIFHLILVNAAGDFFFYICAIQIEGRFDLINDTLLNLEEISAQNEDKYER
MHRIVIECVQQYNIIIECSKLLVDCFKEILINQLICSCLSLMFTLYQLNAAELFSLNFFRVVFIAMALGSEIFLFCF
FGNRLIVKGEILYYSSFESGWYKAPLKIQRDVLIFMQQLQKPVMINVGNIFPLNYETFKGIMQKSWSFFVALK
NTQELRTRNZ

>TcOr159

MRGKTIESTTNPYSSLKKVFIDFAYSKLVISYTKASLTFHVLSLLLEVYYLVTNFSVELICRYGCMMCLMTYMYS
KKLKLLEKPCLLDFWKVYNSSTATQRLISEKSSKTNRRLYCALTCCFFLAIILFPIWGDLNEFFIFSQVYEKYFTS
WAPAFCYFYVSTLLWCCFYCFHLPGIIMYLTLHLDLQFKLIKDKITEIDKNCSQKEIYQILRLCISHHVALKKW
MDKLADLLVTIMPFFFLFGALNSIATSFFVLYTLQNTTMILKIRLGTLTLCNFIIVSTFAEVGQIFSGQNNSLFEQ
LMDCSWYLWNIKNRKTLLMFMLNCMKPKTFSWGGITLNYSFVLFILKTSLSYASVLFKLRGETFZ

>TcOr160

MSGKTKRITTKTIHLSNPYSSFKKVFSDFAYSKIMIFYTIATLAFHMLSLFLQIYYVATNYSVELICRYGPMMCL
AIYVVTAKVVGVFYYKTFTMLENQCLFVLWKTCNSSPTTQRLILNKSLKMNQKLHLALMSYFLLAIVMLPTW
GDLNELFIFSQVYERYFKFWAPVLYYFYISTFLWCSYYSFHLPGCILYLTLLLDVQIKLINDKITEIDQNFSQNEIS
ETLRLCISHHIALKRWMSTLAKMVNSVMPVFVLLGALSTVAVSFFVLNTLQNTTMILKIRLAILTVCNFVIVST
FAELGQIFSDQNNSLFEHLIDCPWYLWNVKNRKILLMFMANCMKPKTFSWGGITLDYSFAISILKTSFSYALIL
FKLRGETIRNZ

>TcOr164

MSGKTKRTTTTRKINLANPYSSLKKVFIDFAYSKIMMFYTKATLAFHVLSLLLELYYVATNFSVDLICRYGCMI
CLMTYVVTAKVVGIMFSKPFKLLEKQCLFVFWKTYNSGPTTQRLILDDSLKMNRKLYLALMFYLLLAIVLLPV
WGDLNEIFIFNQVYETYFKFWAPVLYYFYISTFLWCCYYSFHLPGSIFYLTLHLDLQIRLINDKITEIDQNFCQN
EISETLRMCISHHIALKSWMSKLAKLVDAVMPVFVLLGALSTVAVSFFVLNTLENTSLILKIRLTTLTVCNVFIV
STFAELGQIFSNQNNTVFEHLMNCPWYLWNITNRKTLLMFMLNCMKPKTFSWGGITLDYRFALTILKTSFSY
ALVLYQLRGETNZ

>TcOr165

MSDNTKKATTKSLDLTNPYSSLKKVFINFAYSKIMIVYTSATLIFHILSLMLEIYYLATNFSVELICRYGCMMCLI
TYMVTAKFFGMLFSNQFKFLEEQCLLDFWKAFNSGPTTQRLILKESSKMNRKIHLALTFYVILAIIMLPIWEDV
NDFFMFSQVYENYFANWAPVLYYFYISTFVWCSYYSFHFAGVIMYLTLLLDLQFRLINDKITEIDQNSTQNEIC
GTLRLCISHHIALKRWMNKLANSVDTAMPVFILLGALSTIAVSFFVLNTLQSTSVILKIRLATITVCNLIVVATF

AELGQIFSDQNNSLLEHLMDSPWYLWDVENRKTLLMFMANCMKPKTFSWGGITLDYSFALSIFKTSFSYALV
LYQLRGNTFZ

>TcOr167

MAKTGDIFPVRDPVKRCLFIPKLLLESTNFWPEKRNFLTKFANWVMLIICVLIESGQIAFVVVNIKDITKIASA
MSTVSTTFQAITKLTVLYIYNDKLRLILKSVWYEFWPSYTAGREINTKLETYNKIVIVSFLTILISGICFAFGFLSSP
LISGERILPFETVYPFDWTKSPYYEIIYVTEWMTNIAFILIGICGHDFLFMGLCSNVVGQFTLLRELFGYLGTKNV
AQIIKKLGHDTNIEPNRQLLRICIIHHVRVTEICKEIAEIFSFSCFIQLLSSVTALCVGALIMTFADIDAALFTVSSA
YIVGHLLQLFLYATLGNEVIYYASRLPNAIFHSHWYNIDLEVKKDILFVLQRAQKEVKISAMGVSVLDYQTFIQ
VLRLSFSFYTMLSKVTDHZ

>TcOr168PSE

AATKLIVLYIYNDKLRLILKSVWYEFWPSYTAGREINTKLETYNKIVIVSFLTILISGICFAFGFLSSPLISGERILPF
ETVYPFDWTKSPYYEIIYVTEWMTNIAFILIGICGHDFLFMGLCSNVVGQFTLLRELFGYLGTKNVAQIIKKLG
HDTNIESNRQLLRICIIHHVRVTEICKKIAEIFSFSCFIQLLSSVTALCVGALIMTFADIDAALFTVSSAYIIGHLLQ
LFLYATLGNEVIYYASRLPNAIFXSHWYNIDLEVKKDILFVLQRAQKEVKISAMGVSVLDYQTFIZVLRLSFSFY
TMLSKVTDHZ

>TcOr169PSE

AVTKLAVLYIYNDKLRLILKSVCYKFZPSYTAGREINTKLETYNKIVIVSFLTILISGICFAFGFLSSPLISGERILPF
ETVYPFDWTKSPYYEIIYVTEWMTNIAFILIGICGHDFLFMGLCSNVVGQFTLLRELFGYLGTKNVAQIIKKLG
HDTKIEPNRQLLRICIIHHVRVTEICKEIAEIFSFSCFIQLLSSVTALCVGALIMTFADIDAALFTVSSAYIIGHLLQ
LFLYATLGNEVIYYASRLPNAIFHSHWYNIDLEVKKDILFVLQRAQKDVKISAMGVSVLDYQTFIQVLRLSFSF
YTMLSKVTDHZ

>TcOr170PSE

TVTKLAVLYIYNDKLRLILKSVWYEFWPSYTAGREINTKLETYNKIVIVSFLTILISGICFAFGFLSSPLISGERILPF
ETVYPFDWTKSPYYKIIYVTEZITNIAFILIGICGHDFLFMGFCSNAVDQFTLLREHFGYLGTKNVDQIIKKLGH
DTNIEPNRQLLRIFIIHHVRVTEICKEIAEIFSFSCFVQLLSSVTALSVDALIMTFXADIDAALFTVSSAYIFGHLLQ
LLLXHSHWYNIDLEVKKDIXFVLZRAQKDVEILAMGVSVLDYQTFIQVLRLSFSFYTMLSKVTDHZ

>TcOr171

MVKLFLLLKHLTMKAQDSDNPYIVLRRVFVDFAFTSHMIIYTKITFVFHFLTLLLETYYMITNFNVELFSRYGC
MMCLMTYSNVQIVLAKLLEILFARHIKFLEEERLSHFWKLEESSEETQKVVNAESSKIRKKTFFVLSWFVALGF
VLFPIFGDLNDLFMFGRVYRNYFGSWAIIPFCIYVSTFPSIAYNSICLPAVVSYFIFHLNLQISLINDKLGKISEKSR
QSEIYQKLCSCVAHHVRLRRWTNIFQNELESALPFYLFLGAINSIAVSFFILYNLQNMTLIFEIRLVVISVCNVLI
LWIFAEAGQEFSDNSDSIFDAVVACPWYSWNAQNRKIMLIFMLNCLKPMTFSWGGVKLDYQFTVTIVKMSY
SYALVLYNWRYEKZ

>TcOr172

MSFQALKHLLKMCAEKTPDLDNPYLTLRRVFIDFPYSKSMKIHTCITLLFHFLSLILEIHYLVTNFSFELSSRYGC
MMCLMTYVISVKIFVIMFAKPLKILEEQRELHFWKIGDSSHAMQQSVATEALQVKKQTYFALSCFVLLAVILY
PVWGHVNDLFMFSQVYEKYFGDWSVIPYYFYVFTFMSSSFNSFQLPGVILYFTLHLNLQISLINEKITKISGENY
CQDEVFKQLRDCISYHVALERWMARLIDLTKTAMPVFILLGALSSIAVSFFVLYSLENTRFILKIRLTVVAICNV
LIVATFAKAGQRFSDKTGLIFDAIATCPWYSWNVPNRKIVLIFMANCLKPKTFSWAGITLNYQFAIKIVRTSCS
YALVLYKLRNGNYZ

>TcOr173

MSNVTFDEPFMFFKKVFFDFGYCTSIRFYHLFCFTFHICCQIIENYFFLTEYLSADFVTRYGCPMIVIGYTIVCEFF
LMKWEEPIKELLDERETIFWEIDSNSKPQILKYSSKVNRIYKFFLFWVVILAIFLLPFWGDLDETFFIIRIQKIYFG
KWSTLFYTLYVSTLPFMVYSGIRFPIVTLYLIMQSHLQILILSQKIGQISQNNNHMDDVSKFHDVGYQKKIRTSL
HVCMCRHVTLKQWISKILQIVQKAIPVYFSLAIIVLVTVMFCILYNVESASTTTIFKIRLVLVGICGAVVLFTFSE
TGQLLSDDTSQVFDTLAASPWHEWDPKNRKTLLMFLLNSLKPVKIYWGGFALDYQLGGSVIKTTFSYALVLF
NLRKDZ

>TcOr174

MILFCSTLAKMSNVTFDEPFMFFKKVFFDFGYNKIIKFYNLFCFTLHLCCIITEHYFFLTKYLSADFVTLYGCATI
LIAYIIVCQYFVMRFEKPIKQLFEERETIFWKIDSDQKAKTQIIKFAAKITRIYKFFFVWVVVLVIVMLPFWGEID
KSFLIIRVQETLFGKWSIIFYCIYVSTFPFLIYSSVRLPMITFYLILQAHLQILILNQKIVQIPQNDNLDLDAGLQKK
IYTSLKLCMSRHVSLKRWMSEILQIVRRAIPIFFCLSIICSVTVIFFILNYLENSNTSNLLKIRLVLVGICVAVVLYT
YSEAGQLLSDDTSQVFDTLATCSWYEWDTKNRKMLLMFLLHSLKPIKFYWGGFALDYRFGGSVVRTTFSYAL
VLYNLRKSSZ

>TcOr175

MRNFQDSDDPFIFIRKVFVGFGCSTIIMYYSRLIFIFHTLSLLLESYHVITNFSLDIITQYGSAMSLMLYSITSQFLLI
CEQNLITEVVEECKSFFWTMDFLSFIKQTQILKDMTKIKRKMYLSWIWFVVFGIALLPVWGDYNEMFLFPFIY
QTYFGNWSPLFYYFHASSFPFLAYIAIRIPAFILYLTLALHFQTLLLNQKILQIPQNKSGNQEDIFRNLCSCISHH
VALKKFVTKTQQSIQKMIPVYFVLAILCLVAVMYSCLNSLAMSTSNHFKVRGFFGGVCGVVVLYTFAEAGQL
QADTTGEVFNTLMQCSWYNWNNRNQKILLLFMVNSLKPSYIDWGGVIVGYSFGSSVIKTCYSYALVLYKLKI
SKEQNVTFZ

>TcOr176PSE

MXDVIKTQTNDPFIHIRKIFITYGYSKVISYYSRFILIFHTLSLLLESYHVIKNFSLDLITQYGSGMALMLYFITLQF
LVIIMQKFILQIIEDIKSFFWSMDSLHFDKQTQILKEVTRMKLKIYLVWLWFVALGIVMLPIWGDYDEILLFHLL
PDIFRKLSPFLLFYASTFPFVATLQFEYVLVXIVFHLELRFQTLLLNQKILQTSQDETRDEEKVFRNLCSCICQHV
ALKNLVRKLLQGIQKIIPVFFVLAILCLVAVMYAFLNGLQMSTSNHGKARIFVSGICGAVILLTFSEAGQLQAD
TNGEIFNTLTQCRWYNWSTQNQKVFMLFMLNTLKPLQVDWGGFILDYNFGSSVIKTCCSYALVLYKLKNAN
EQNVTFZ

>TcOr177

MYRQVPPHDPFGSLKFFFIDFGYNKIMSYYSHFGLIFHTCSLFLESYYMIENFSLDFFVKYGCAMTLMFYFIISQF
VTIAIETLVHELEEESKLFFWHIDSLDSKLQRKILKKSAKTNRKYCLLWSPFAVLVIVLLPVWENLNESHLFPQ
VYESYFRIWSPVFYYVYVSTFPLLGYTAIRNPAIILYAVLNLDLQIVLLNQKILQISGDNDFECVRFQEKVTRNL
CLCISQHVALKKWTNKFLKPMQKTIPVYSCLGVFCLVTIMFFILNNVNTNVSNHLKIRLLVSGICCSLILYTFSE
TGQLLVDTTGQVYNALVECPWYCWNIKNRKLFQMFILDSLKPLKIYWGGLVLDYSFGGSIIKTCCSYALVLY
KLKNAKZ

>TcOr178

MAKARYLQDSDDPFIFIRKLFVDYGYSRIINSYNRFIFIFHTCSLLLEGYYIIKNFSIDFFTQYGGATNLILYFLVT
QFIVIIKQDFVHQIVEESKSLYWSMDFLDVNKQAQILQEMTKLKRKIYVLWMSTVIFAIMMLPVWGDYSEAH
LFPQIYQTYFGNWSPIFYYFYVSTFPFAAYTGLRIAAMALYFTLIIHFQIILLNQKILQIAQERDTSQEEIAKNLRS
CVCQHVTLKRFVAKVLKSIENAVPVYFCLAILCLITVLFVILNNLDTSTSNHLKARFLFAGIYGTVILYTFTEAG
QLLSEINDQVFNTLMQCPWYNWNTKNKKTLMIFMLGSLKPVQIVWGGVNVDYKFGGSVIQTCCSYALVLY
QMRNAKZ

>TcOr179FIX

MGKPRCFEESDDLFLCQKKIFIDFGYNKFINYYNWFVFIFHTCNLLLEGYYMFKNFSLDNLTKYGGVMTLMC
YFLITHFVTIAKQNFILQIVGEQRTVFWSSDCLDPNLRKELVKNAAQTNRKLCLVWIWFVALGVVMLPVWG
DLDESHLFPIVYETYFGLWSPLFYYFYASTFPIIAYSSIRLPGLALYAMLNLNMQTVILNQKILQISADCKSDLVK
FRDNGYQDQVYKQMCACISQHQVLKKWITNLIEFMRKTVPFYSIVGILCLVAVMFLTLNNLSTNTSSLLKIRF
LVTGVCGCAILYTFSVAGQLLTDTSNDIFDTLLKCSWYHWNTKNRKLFVTFMLYSLKPLTINWGGVTLGYRF
GGSLIRTCCSYALVLYNLRNAKZ

>TcOr183

MYKQHKKQHFDKSDDPFINLKHVFITYGYHKLVKYYSGFAFIFHTCSMVLEIHYMIKHFSMDLVTKYGGAIM
LMNYFLISQIVTVVIEKLYLPELVKIRDLVFWKIDSFGSSVKEQILNDSIKMKRKLYFVWILFAAFGIILLPICGDF
DESHLFPKVYETYFGTFGTIFYYFYVSSFPFVLYTSLRIPVFALYGVLQLHVQIILLNHKIRQLSHGLVDIDNVRY
QERVSQNLCLCFSQHVALKNWLSKCLKFMKNVMPVYFCLAIICIVIIMFFILNNLVLNTSTHMKLRFFICGICS
SVVLYIFSEAGQLLSDDTGGVFQVLMECPWYCWNTKNRKIYTMFLLSAVKPLTVDWGGLILNYNFGNHVFR
TCSSYALILYKLRKAKZ

>TcOr184

MSQLKARTFFGELDDPFLLIKKIFIDYGYSKPVNYYNRLGFILHTCTLILENYFVVKNFSLDYFTKYGCVVILMH
YFLISQFLTILNEKLFKELIGERGSLFWKSDSSSPRVKNQIVKHSVKFNRKFCFVLFWFVALGIIVSPVWGDLSET
HLFPQVYKTYFGIWSPVFYYFYVSTLPLVAYTAIRIPAIALYLILQLDLQKILLCEQISLIPSSDQVRIYKKMRLCIS
QDRELKKWIAKIRLSLKRNMSLYICIAFLCLITVMFFILNNLTTTTDNVTKMGFFVAGMCGSLILYTFSEAGQL
LFDYTEDVSNTLIQCPWYCWNTKNRKLYLMLMQNCQKPLKINWGSVTLNYCFGGSVIKTCCSDASIFFKLR
NDKZ

>TcOr185

MDDPFIFIKKIFIDYGYSKPLNYYNRLVFIFHTCSLTLESYFMLKNFSLDFFTKYGCALILSHYLLLSQFVTLLNK
ELIKELIDKRGRFFWTIDSSAPSVKNQILKKSLKFNRKYYFVLFWFVALEVTLLPIWGDLNETHLSPQVYKTYFE
TWSPFFYYFYVFSFPLLAYHGIGFPATVFYFILQLDLQKILLTDMVLRIPDSGQEAIYENMCLCISQDKKLKKWI
ARIQYLLKKLMALYICVAFLCLISVIFFILSNLTNSRSILAKSRFFCAGMAGGVVLYTFCESGQLLMDYTEDVFS
TLTQCPWYGWNTKNRKLYVMFMQNTQKPLIINWGSVTLNYSFGGSVIKNCCSYALVFYKLRNGQZ

>TcOr186

MAHNPNLFELIKMFKIRDPFAILKIILKAEPFILLKWCFIDLSYHKFVKICNVLALIIHSVYFVFDVLYTIENFSLD
FLLKYSSSMLIYIYVITNMVFAMVMEKYTLEMVNLSESEFWPMDFAGKRMEAEIGRNYLMTRIVYYFLGGAL
LASMLILLPFFGDLNDWLLSSQMSVDYFGEWSVLVDLILFSTGPMVALSEIRAPAVFLYGIYKIDLQIFLLNKLI
VQLSHEKARDDAKYQRRIFAKLRQFTKHHANLKKCMRGISDIMNLSMPVFVFIGAPGVISIMYYFLYSLDSAS
TITKIRSVYVAVFTSVIVATFAHAGQKISDNTSLIFDTLTTCPWNLWDKRNRKVLLIFMANSVKPMTFSVAGIT
VDYQFAIKMIKLSSSYALVLYQLKNQYNMNZ

>TcOr187

MSTKREVVKNFPYYYLFKICIDFGYSNVVKRLNICCITMIVMFHLTQIHYMQENFSKELILKYGSGIALGIYTILS
MSVQMLIEHEIKDLIAEALFSMWAVDSCGPQVEKLILRRAKVMNIIYCSIFAWFALMATVMLPMWGDHSEW
LLYDPILVEDVKTRLKIIYYLSTFIIFPMIAFSAIRLPGILLYGILQIHMQIMLINHKLVQVSEDLDDLNNVKKIDQ
DDYQERIYKELCLCVEHHIKIKLWLNKLMKIVQLLMPPYFLLGSINAIYLLFFVVYNDTSNILKVRLCILLIVGG
QILCMFAEAGQALGEETGRIFDTLVNCPWYLWNKKNKQALTIFLSNSFQPYTIAFAGFTLNYSLALALLRSSV
SYALVLYNMRNZ

>TcOr188

MFVKRQVLEGFPYYYLLQLCLDVGYSKMMKIANIFCIIINLLNVLAQIGYIKQNFGKELLLRYACGIQLTIYTIV
TMLFEFLVEQNVKKLMDEALSEMWPIDFCGLEIKKLILKRSTVMNSIFYFMFAWFAILAIVMLPMWGDQSEW

LLYDRICKEFFATWWKIPYYFYFTTFPVVAFSGIRLPGLLLYTILQTHMQIILINQKLVQISGGLDGINDVRMID
QKNYQKRIYKGLRLCVAHHVAIKRWLQKPVKIVQSLMPIYIIMGSTIFISLLFATVYSFRDSSNILKVRMSVVL
MICCLILCMGAEAGQALSNETSRVFDTLVNCPWHLWDQKNKKALTIFLPNTLQPVTITLAGITLNYSFAVGL
LKSSASYALVLYNMRNZ

>TcOr189

MEKMFPQIRTEDMKKFPYYYLLKICIVFGYSKIVKLLNVVCIIITSSTIVLQVYYLKQNFSKELILKYGCGISLTIYT
IASMLVEFLIEQKTKKLLNEAGTILWPVNFCGVKVEKLILKRVTVMNIIYYFMSAWFALMGIIMLPIWGDHSE
WLLCDVISNEYFETRWKILYFACSCFSFPVIAFSSIRLPVILLCTILQTHMQIILINQKLNQISEQMGNLNNIKLV
DDKCYQKRIFEDLRLCVSHHGKIKKWLNKVLKLVQSIMPLYIILGCLNFISLLFFASDGLQNASNILKARLCVV
LIVCCLVLSMFAEAGQALSDETSGVFDTLLTCPWYLWDKNNKKVLSIFLSNSFQPDSISVAGITLNYDFAVAL
LKTSSSYALVLYNMKNZ

>TcOr190

MSTKKQDLLKHFPYYYLWKVFINFGYSKLTKLVTISCIIIHSSSLFVEIYYIYCNYNKEIIFKYGCMMSLLGYITIS
MVVELLLEKDTNNLVCEARSLFWTIDSCGVQAQQIIHKRAVVMNATFGFILMWVATLGVIMFPIWGDQSEW
VLCVKIFENYFENWSQMANFVFFSTFPMVAYSTIRLPAMLLYGILQTHMQIFLINQKITEISRSKDQEKIYKELC
LCVSHHVEIKRWLQRFLKMVQLTMLMLIPLGVLSCVCVLFFVIYSFLDTSNILKMRLTVVVACTVLIVYIFAEA
GQDFSDEISCIFDTLVTCPWYFWDQKNKKALVLFLANSLKPYTLSIAKITLNYDFAVALVRTSVSYALVLYNM
KNZ

>TcOr191

MRLEIEALKNFPYYYLLKICIDFGYSKIVKCINVVCIIINSSTLFIQVYYVQQHFNKELIFKYGCGMALTIYTIASIS
VEFLIEKNAKNLVNDATAFVWPVDFCGEKVKKLILKRATVMNKICYFMSAWFALMGIIMLPVWGDHSEWL
LCDLLSKEYFETRWKILYFACSCFSFPVVAFSSIRIPGILLCTILQTHMQIILINQKLNQISEQMGNLNNIKLVDD
KCYQKRIFEDLRLCVSHHGKIKKWLNKFLKLVQSIMPLYIILGCLNFISLLFFASDGLQNASNILKARLCVVLIV
CCLVLSMFAEAGQALSDETSGVFDTLLTCPWYLWDKNNKKVLSIFLSNSFQPDSISVAGITLNYDFAVALLKT
SSSYALVLYNMKNZ

>TcOr192

MVSEQTLLKNFPYYYLLRIFIDFGYLKITKVLSVACIIIHSLSTLLEIFYICQNFSKELVFQYGCITSLATYVITSMTT
GFIIENDAKNLIRETVTAFWPIDFCGPQVEQLIFKRVARINTFNFFLLAWFAIFGIIMFPVWGDESEWMLCVIAF
KKYFPKWWRVPYYVFFATYPMVAYSAIRIPAMLLYGILQINMQFFLISQKIIQISQKPQNKTHQPGFYQKTVY
KKLCQCISQHAEIKRWLQRFLKMVKSVMPVFIFVGGLCFMSILFFVVYTFQSTSNILKVRLGVILMICNLILVTF
AQAGQTVIDESSGIFDTLMTCPWYLWDEKNKKTLVIFFSNSLKPITFSIASITLNYAFAVALLKTSASYAIFLYNI
KNZ

>TcOr193

MSELEKQLPYYFLMQFCINFFYSKTVKVVTSSCIIIQSLSLLLQVYFIITNFSKELILKYGCEMSLATYLLTSLLVD
VVVENTTKQLISEGHTSFWSIDSCGHDVKNHIIANSARLSVVIYFILAWFAVLGISVLPVWGDQSEWILFVQIF
NTWKKILCYVYLSTLAVMVFLSIRLPAMLLYGILQIHVQIILINQRIIQIGRENTNDIRMMNQMSYQNRIYKEL
GFCVSQHARIKRWLKKLLGIVQSAMPIFTVLGGLIFISVLLFVLYSFENASCFLKIRLGMVVISCSLVLCMFAVA
GQAFSDETSRVFDTLMTCPWYLWDQKNKTILLIFLSNSLQPINFSIANITLNYSFAVALLKTSTSYALILYNMK
NZ

>TcOr194FIX

MAMKQYPFLYKIFLDFAYAKIGKMVTYSCIIIQSLALQLQVYFIVTHFSKELIVKYGPGVLVVTYLVTSLVVELM
IENKTRKIIDFARLTFWPTDFCGLEAKNRLIKNSSKVSIVIYLILMWFAAQGIVMFPVWGDTSEWRLHVEIFDQ
WKLFYYIYVSTFTIIVFSAVRLPGILLYSIFQTHMQIVLINQKITQISQNDPNDIRMMNQTGYQKRIYKEMCLCV

SQHIAIKRFIKKLLEIVRPVQPIFMVLGLLGVISIFFFALYNLENTSNILKIRLVMVVISCILILCLFAEAGQAVSDE
TSRVFDTLLTCPWYLWDQRNKKALAIFLSNSLQPISFSMAGFTLNYGFGISMLRNSASYALILYKMKNZ

>TcOr195

MFRERVYDDRFIVLKTIFLEFAYCKEMKIYNMFCLVFHLFSFSLQVHFIVLNFSVELITRYGCMLTVFLYLIAAK
SFSIIIEKQVRMLEMEATSFFWPIDCCGPQVKKNIYDRAARQNIQNYFTLAWFALFGIIMLPVWGDQSEWFLCI
QVFQQYFGCWKLFYYFYFSTFPMIAFTAFRLPALMLYGILHEHLQLILVNQKIVQLSVRRSLKENIVDNANYQ
KTVLKKLKLCISHHVKLRDSLGKLIGVIQLAMPVFLFIGALGSIAVLYFVLYIFLSSSNILKIRLVVITICNGLIVYT
FSAAGQALADETGRVFDTLMTCPWNTWNIKNRKVLLIVMSNTIQPLTFTLAGITLDYKFGLTMLRISCSYALI
LYNLHZ

>TcOr196PSE

MFKEKTFDDXFYCFQKSFFEFAYCKIMKIYNTTCLVFHLFSFLLQVYFMVQNFSAELITRYGCILAVFLYIIAAM
SFAIFIEKQVKMLEVETTSFFWPIDCCGPEVKKLIYNRSARINILNYFTLAWFAVSGIIMLPVWGDQSEWFLCIQ
VYQEYFGVYWKLFYYFYFSTLPMVAFTAFRLPGLMLYGILHIHLQLVLINQKIAQLSASRDLDENIVDDANYQ
KTVFRKLKLCISHHVKLKTSLRKLIEVIQMAMPVFILVGAICSIAILFFVLYIFSSSSHILKIRLAVSAITNVLIVYTF
SAAGQAITDETSHVFDTLTTCPWNAWNIQNRNVLLIIMSNTIQPLTFTLAGITLDYKFGLTMIRISYTYALILYN
LNZ

>TcOr197

MFKKRKFDDRFIVFKKIFFEFAYSKEMKIYNMICLVFHSFSFVLQVYFIVQNFSVELITRYGCILAVFLYLIAAMS
FAIFIEKQVKMLEVETTSFFWPIDCCGPQVKKLIYDRSARINILNYFTLAWFTLFGIIMLPVWGDQSEWFLCIQV
FQQYFGSCWKLFYYFYFSTCPMIAFTAFRLPGLMLYGILHIDLQLVLIYQKIAQLSARRIFSENIVDNAHYQKT
VFRKLKLCISHHVKLKTCLRKLIELIQMAMPVFIFVGAVCSIAVLFFLLYVFSSSSHILKIRLAISVVSNVLIVYTFS
AAGQAIADETSHVFDTLMTCPWNAWNNKNRKVLLIIMSNTLRPLTFTLAGITLNYKFGLTMIRISYTYALILY
NLNZ

>TcOr198

MPNVTNKRQKRLFSKTRTKSEDPFVMIKDVFVDGGYHPVTKMLNYICLVIHSCSLLLELNYFVHNYHFDLM
MKYCCAMSLMGYIIATMLFAIFQEHSAIDLTKDILSLFWPIDYCGPRVKEEIVKKATKINRIHYIVLLFAGALGI
TMFPIWGDQKEWFLCVQVYQHYFGKWSKIPYYVYFFTYPMLAFSSVRLPFMTMYAIVQIRMQVYLLHQHISE
ISGEYVYDMKNLQILCDQNYQNEIYDKMRLIISHHIMLKRWMRKLVHTVQISMPVFVLLGTMTSISVLFYAIY
SFHNINFILKVRLISVSVCTVLVVYMFSEAGQALSTETTGVFDLLMTCPWYVWNIKNRRILLIFMANSLEPMTF
SLAGVTLDYRFALGMLRTSCSYSLILYKLKTGIZ

>TcOr199

MSMTRSKYFQDSDDPFSFIRKIFIDYGYSKKINYYNRVTFTFNTCSILLESYYMITNFSLDLFVRYGGALSLMLY
HVVTQFLVIAKQKSLEQLLEESKSYFWKADIFNSSVKNQILKSCNHMQRKFCLLWTPFVACGIVLLPVWGDF
TESHIFPQVYKAYFGHWSPIFYYFCISSYPFAVYTSIRLPAIALYLFLQAHFQIVLLNQKILQISKNNDLDETTIFE
NMEYQKTIYRNLRSCISQHVALQKYITRILVSIQKAIPVYFCLAVLCLIAVIFFVLNNLNMSASNHFKARIFVSG
VCGSLILYTFTEAGQLLADTTGDIFNTLMQCPWYYWNIKNRTVFMIFMLHSLNPLKIDWGGFTLGYSFGGAV
IRTCCSYAVGLYNLRESKYZ

>TcOr200FIX

MSIQNTKKTLNGKEKHPDFIKFCFTDSNDSFLILKRVYIDFCYHKITKTCNFLLVISQLFFYLIQIHFLLSRFSLEL
LARYSTIMMITTVALFGLILSFYLEEDIHELYKILTEIAWPLDKASKKDQQDLRQKSRRINSLNLYFLGFLAFMI
VIFLPIFGDEENLFLCIQVFDEYFGDRAFIFYNLYFIGFPFLIYFSVQLCFMFLYAILHLHVQINLINHHICEMGAS
FELLSDWKKLHSVVYQSAISQLLCQCIRQDIALKRFIVKLNETVQLGLPFFLPVSGLCGISVIFFLLNYMCTMSL

VLWLRVSAFFICLVFVALIFSVSGQLLIDETGKIFDTLVKCPWHIWNVRNRKIYLICLTHCVRPNCISYAGITLN
RIFLITVFYKTVSNAFILYQVRNSZ

>TcOr201PSE

MVEFKDPFIMLKTIFLLNVKEMTKFSQVFLPIFAFHSLVXYLYKNFDVNLLIRYAPMTTGTLFVSNTKLLSSSLN
IIPVFSVVSETKLLRIATFIDKTCWSLDTIRKEARIKLERKCRATNISISCILLLLSVAVFSNFPCFGKQDDFFLCIKI
FEEYFGQWSSIPNYIYFTLFPILCYSYFRIVFSFVYAILETQLQFSLIEEYLFEVYQMDDLNWKYLQDPRYQQEIG
KSLQLCIEHHTALKKLIHSIMNVTLTAIPFFLPFVILLFVSCFLFFINFGDTMTIILKIRLLFYVGAILSMTLLMCW
YGQQVINVTNSIFFTLVGAPFYFWNLNNMKILLMFIMNCTKNESIVLTGIYVDYTLAVSVLRISVSYTLSLLGLR
NRSFDZ

>TcOr202

MVEFKDPVIMLKTIFLVNVKEMTKFSQVFLAIFTFYSLVHCVQMYYLYKNFDVNLLIKYAPATTATLFVSNTK
LLSSSLNIMPIFSVVSETKLLRITTFIDKTFWPLDSIRKEARIKLERKCRAINISIYCILLLLSVAVFSNFPCFGRQDD
FFLCIKIFKEYFGQWSSIPNYIYFTLFPIFCYPYFRIAFSFVYAILETQLQFSLIEEYLFEVYQMVDLNWKYLQDPR
YQQEIGKSLQLCIEHHTALKKLIHSIVNITLTGMPIFLLFGIGLFVSCFVFIINFGDTMTMILKLKTPLLLYVATM
LSMTLLMCWNGQQVIDVTSRIFYTLVRAPFYFWNLYNMKVLLMFITNCTRNENIVLAGICLDYTLSVSILRISV
FYTLGLLELRNHSFDZ

>TcOr204FIX

MTNFFSNFCSPLKNHWAKTKHLFSKFSLSSDQPFIMIKLVCVDIGYHPVAKTINYICLAIHISSFLLEMNYLRLN
FSTDLLIKYGCGISAVVYDISTLIVAPMIERPTIGLSEGITTSFWPIDFCGPKVKQLILEDTKKTSKIYYRTLVTIFGF
AAVIMLPIWGDQKEWFLCVQVYEHYFGKWAQIPYHIYFLSFMWFAFTSVRLPLMMSYAIKNIRVQVFLVNQ
KIAKMSKEYEEAKIEDVNYQNRVYKNLRLCISHHVLLKWWLRKLQKIVRFCLPVFVVIGILTESSVVFYLIYNF
KKVNLLLKIRFLLLACTTGVIIYFFSEAGQSLYIETSQVFDSLISCPWYSWNVKNRKVLLIFLTNSLQPMFFSLVG
FTIDYRFALTMIRTSFSYAIILYNLSSGSQIASIZ

>TcOr205

MTNIFSNFSLYFKNTWTKTKQRFSKTLPSSNVPFMMIKLVFVDIGYHPVSKIINYICLAIYMSSFLLEMNFLRLR
FSTHLLIKYGCGSSLSVYFISSMTVAAMTELLAVDLSEGILSSFWPIDFCGPQVKQLILKQSRADKRMHYVVLL
VFSITGLAMLPIWGDQKEWFLCVQVYEYNFGEWSKIPYYIYFFTFPWVAFSSLRLPFMMNYAILNLRMQVFLI
NQKIAKMSNAYDQTTIEDVNSQKRIFKNLRLCISHHILIKWWLRKFVNHVKFCIPIFVIVGIATSISIVFYLIYSF
QQVNLVLKIRFLSIACCCWFVIYLFSEAGQSLYEYTEIFHSLISCRWYIWNVKNRRILLVFLANSLEPMTFSLAGI
TLNYRFALNMMKTSCSYALILYKLNCDSQIMDZ

>TcOr206FIX

MWNNNPFIVIRTIFLDINNYKIVKFCYVSLTVFYSLVHCLQFYYIIKNFNLNLIIRYGFITSLLSYVLAAGILSLVV
EKRIRKTQIFFDEIGWSLNIVGKDAEMKLEKKCKLINISIYAIMLLLIITLLVNLPFVGSQRDLFLSIQVFEEYFGK
WSEILDRLYFALAPFLSYHGARLSFTCIYAIMQVQVQFSLIGEYLFETYQVDDSKSWKYLQDTRYQHDIGESLR
LCVEHHVALKKSIKMMVDIALTCLPFLVLLGLCTLISCLAFIMNFWDTMDNILKLRIFMWAAWIVLITIMFCR
SGQQLIDATSDIFFTLGGAPWYYWNLDNIKILLTFMANSTKNDSISLAGICLDYPLFVSVANTTVSYALVLYNL
RESSLDSSNKKZ

>TcOr207PSE

MVEFNGPFIMLRTILFIDMNTYKILKLFNVLLNVIYSLIHCLLVYHMFKNLNINLIIRYGPAVLFLILVIVGAVFS
VYLEKDILEIVTLFRKTRWSLSMIKKDARIKLEKKCKIINIFILFLVLLIISTITINAPYFGDQRELFICIQVFEEYFGE
WFFIPYNFFFVAFPFLYYNFFKLWMTFVYGILEAQLQFFILEEYLCGTFETDFRKNWEYLQDTRYQQEIGTSLR
LCIAHHINLKKLIKMIQNVTLMVMPFFLVLGVLILISSFSFIINFADTMTTIAKIRMVISAISMVGITILLSWIGQQ

VIDVTSDIFVTLGGASWYYWNQKKYXKTLLMFLTNSIKNESIVLAGICADYGLCVALLRLSVSYALVLFNLRKSTLVZ

>TcOr208

MEKLDDPFITLRKMVFIEAKNCKIARFCDVLLIVLYSLAQCLHLYYMCQNFNLNLLIRYGPILISCLLVIVTAVISVGLDKEIFEVYTVCWKISWPLNFLRKDAQTKLRRKCQIINRGILCSALLFLTTVISTFPCFGSVRDFFICVEVYEKYFGEWSFIPYYFYFAAAPFLYYHFFRVCYVFAYAFLHAQLQYFLIEEYLLETYQTNDLKGWKYLQDTRYQQEIGKSLLLCITHHIALKKYVKISQNLVLIGMPFFLVLGVLLLINSFGFITNFGDTMSNILKIRILIFVACGVSITIVMCWIGQQLIDVTSEIFVTLGGAPWYFWNRDNNNILLMFLTNCTKNESFILAGICVNYQLFFSIVRLTVSYTLVLYNLRESGFIZ

>TcOr209

MMPEFSDPFIMLRKMIFIKNHKIAKFCDFLIIAIYSSAFCLQIYYLCKNFSISLLIQYSPTLLCYIFVIDAAVLFFYVEKNILEAITYYDEIGWSLSMIPKDAQTKLRKKCLIINICVSFILLLILSTLTINLPYFGSQRELFICIQIYEEYFGNWAFVPHHFYFGVFPFIYYNSVKMWISFVYTILEAQLQFILVEEYLLESNIINDFKGWKHLHDIRYQQEIGKSLRLCITQHIALKKLVKMIVNITMAAMPSFLVLGVLLLISSFAFILNFADTMTNILKIRVLMFVACIVCITTLLCWTGQQVIETTSDLFDSLVGAPWYLWNRENIQIFLMFLVNCTKNESLVLAGICLDYRLFVSMLRISVSYALVLFNLRKSSITZ

>TcOr211

MTNKLSYAPFTLLRKLIFIESKHCKLARFCDFLLIVLYSLAQCLNMYYMYQHFNLSLVIRYGPVLVFSLLVIVTSVISVAWEKEIFELHMVNRKIFWPLNCVGKNAQTKLTRKCQFINHWISCSLLLFLITVIINFPCFGSQRDFFICVEVFEKYFGEWSFIPYYLYFAASPFLYYHFFSSSFLFVYTILDAQVQYFLIGAYLFETFQTDDLKGWKYLQDAHYQQGIGKSLRLCIENHIALKKFMKMSLDFVLIGIPFFLVFGVLLLISSFAFITNFADTMSNILKIRMLIFATSSVCITMVLCWTGQQLINLTSEIFLSLGGAPWYFWNRDNRKILLMFLTNCTKNESVVLAGICINYAFFLSLVRLTVTYTLVLYKLHRSGIVZ

>TcOr212

MAGFKDPFIVMRTIFLDIINYKIVKVCYVFLFMIYLLVHCLQLYYMIKNFDLNLLIKYGFMTALFSYILVVAVLGLVVEEKIRKTLKILDGVGWSLNIVGKDAEMKLEKKCKMINISVYAIMLFLIITLLVNLPVFGSQRELFISIQVIEEYFGKWSEMLNRLYFTFAIFLSYHGVRLSFACIYGILEVQLQFNIIEEYLCEIYETDSSKSWQYLQDTGYQRKTGKSLRLCIEHHVALKKVIEMMLEISVICLPFLAVVGLANLISCLTFIMNFWDTMDNILKLRIFMWAGWIVLITVLFCRSGQQLIDVTSNIFFTLGGAPWYYWNLENIKILLTFMTNCTNNDSIALAGFCLNYPQFVSIANTTISYALVLYNIRKSSDSDYZ

>TcOr213

MAKFNDPFKFVRTIIFVDMNSYKVIKTCNVLLNIIYSLIHCLLIYYLCKNLEINLLIRYAPAILLFILVIFGAVFSIYMDEDILEVRSVFRENRWSLSVLKENSQTKLGRKCQFINIFILLVLLLIVSTLAINAPCFGNQRELLICIQVFEEYFGEWSFIPYYFFFLGFPLLYYNFFRLWMTFVYGLLEGQLQFFILEEYLCGIYETEDSKSWKYLQDSRYQQEIEKSLRLCISHHIGLKKFLKMVENQTLKVMPFYLVFGVLILICYFSFIINFADTVTTIGKIRMFMTAICMMGVAILLSWIGQQLIDVTSDIYFTLGGAPWYYWSQKNAKLLLMFLTNCTKNESVTLAGISLDFTLFVSIVHTTLSYALVLYNLRESSLVSSSQKZ

>TcOr214

MAEFIDPFLMLRALVSVKFNDYTSLKLCNILLITIYSLIHCLLIHYMFKNLDINLAVRYVPMIMFLTLVIVGAIFSVAIEKDILEAQVFLFKANWSLEMIRKDAQLKLERKCRIINICILCVLLLIFATITINAPLFGSQRELFICIQVFEEYFGKWSFIPYYFYFAAFPFLYYDFLKLWMSFVYAVLEVQLQLTLVEEYLFETYQINSSKEWKNLQDTHYQQQIKKSLRLCITHHIALKKFVKMTVDLTIKVMPFYLTIGVLILISFFSFIINFADSMSNILKIRIFMFSASIVSITVLLSWIGQ

QLVDVTSGIFWSLVGAPWYFWNLENVKTLLIFLMNCTKNESIVLAGICIDYSLGISILRLSVSYALGLFNLRKSS
LDZ

>TcOr215

MAEFIDPFRMLRALVSVKFNDYTSLKLCNILLITIYFLIHCLLIHYMFKNLDINLVVRYVPTIMFLTLVIVGAILS
VAIEEDILEAQVFLFKANWSLEMIRKDAQLKLERKCRIINICILCVLLLIFATITINAPFFGSQRELFICIQVFEEYF
GKWSFIPYYFYFAAFPFLYYDFLKLWMSFVYAVLEVQLQLTLVEEYLFETYQINSLKEWKNLQDTHYQQQIKK
SLRLCITHHIALKKFVKMTVDLTIKVMPFYLTIGVLILISFFSFIINFADSMSNILKIRIFMFSASIVCITVLLSWIGQ
QLVDVTSGIFWSLVGAPWYFWNLENVKTLLIFLMNCTKNESIVLAGICIDYSLGISILRLSVSYALGLFNLRKSS
LDZ

>TcOr216

MTKFNDAFPMIRAIVSLNFNANTSFKLCNIMLITIYSLIHCLLIHYMFKNFDINLVVRYTPTIMFITLVIVGAIFS
VAMEKDILEAYAILPKANWALEMIKEDAQLKLERKCRIMNICILCVLLLILSTITINAPFFGSQRELFICIQVFEE
YFSKWSFILYHFYFIAFPFLYYGLLRLWMGFVYAVLEVQLQLTLVEEYLFETYQINSLKEWKNLQDTHYQQQI
RKSLRLCITHHIALKKFVKMIVDLTIKVMPFYLTIGVLILISFFSFIINFADSMSNILKIRIFMFSASIVCITVLLSWI
GQQLVDVTSGIFWPLVGAPWYFWNLENVKTLLIFLMNCTKNESIVLAGICIDYSLGISVLRLSVSYALGLYNLR
KSLDZ

>TcOr217

MVEFKDPFIVLRKIFFIKFNNCKLTLFLNISIIVFFSLVLCLQICYLMKNFNLNLLFRYGPVTVLFTLVTVTAVLSL
TLEREIFMAITFFFKFCWSLNIIRNDAQITLKRKCRCVNIGLLCILLIILIAIVIGFPCFGSQKDFFICLEVFEEYFGE
WSFIPYYFYFAASPFLCYHFLRICFTFVYAILEAQLQYLIIAEYLFEIYQTNPSKRWKYLQDTRYQQQIGKSLRLSI
VHHVVLKKFLKRTLHLTKIGMPFFLVLGILLLTSSFAFIMNLGDTMSNILKIRIFLFTTSVLCITILLCWIGQQLID
VTSQIFVSLSGAPWYFWNLENIKILLMFLTNCTKNESIILAGICLDYKLFVSVARLTVSYAVVLFKLHKSSLVZ

>TcOr218

MMDKLNYAPFITLRKIIFIEAKNCKLARFCDVLLIVLYSLAQCLNMYYMYQHFNLSLVIRYGPVLVLSLLVIVT
SVISVALEKEIFELHMVFRKIFWPLNCVGKNAQTKLTRKCQITNCWISCSLLLFLVTVISSFPCFGSQREFFICIEV
FEKYFGEWSFIPYYFYFAASPFLYYYFFRICYLFVYAILDAQVQYFLIEEYLFETFQTDDLKGWKYLQDAHYQQ
GIGKSLRLCIENHTALKKFMKMTLKFVLIGMSFFLVLGVLLLVSSFAFITNFADTMSNILKMRMLIFATSTVCIT
MVLCWTGQQLINVSSEIFLSLGGAPWYFWNRDNSKILLMFLTNCMICLNYELFLALVQLTVSYTLVLYNLHK
SGIAZ

>TcOr219

MNKFHDPFIMLRKLIFIEARNCKLARFCEILLIVLYSLAHCLQIYYMYQHFNLNLLITCGPIMGTVLLTIVTAV
MSVGLEKKIFEALTVLSNISWPLNFLKKDSHTKLTRKCQVIKWCISCSVLLSVITLISTFPCFGSQRDFFLYVEVF
EEYFGEWSFIPYYFYFAASPFLCYHFLRVTFVFVYAFLHVQLQYLLIEEFLFETYRTDDLKGWRYLQDIRYQQKI
GRSLRLCITHHVALKMFVKKTVDLVMMAMPFFLVLGVLLLISVFTFIINFAVTMSIISKIRILLFAATGVCITMT
FCWSGQQLINVTDEIFWVLARAPFYFWNRENSVILLTLLTNCTNNDSVVLAGICLDYKLFLSVVKLAVSYSLV
LFKLRKSSLVZ

>TcOr220

MADSKDPFIMLRRIFIDVNSYKITKLCDVSVVTFHSLVLCLQLYYMIANFDVNVLIINGPPTIVFLLMTVSAVLS
EAMAKDIFKGITFFQQIRWSLDVIEKNARIKLERKCQTINICITCILLFLSTTMVINMPFLGNPRQFFISIQIFEEYF
GKWSVLLNVLYFTGLPYLGYHAVKLCFAFVYAILEIELQFSLIEEYLFQMYEVDYLKSCKYLQDARYQQEIGNS
LRRCIIHHIALKKMVKMLVEVVLKCMPFYLVLGVLLLITCFAFIINFADTTTSTIKIQIFMFVASTLCITVLFCWN
GQQLKDVTSNIFFTLGGAPWYFWNLENIKILLMFITNCTKNDSIVLAGICLDYKMFVSVLRTSVSYALVLFNLR
KRSLVZ

>TcOr221

MAEFKDPFIMLRTIVFINMNSYKVLKVCNILLIVLYSLIHCLLIYYMFKNVNINLVIRYSPAMLLSILAIIGAVFS
VFKEKDVLEIDKVFHKTRWSLNMITEDAQNMLKRKCLIVNACILFLLLVIVAMFIINAPCFGNQREIFICIQVF
EEYFGKWSSIPYYFYFFGYPFLYYNFFKGWMAFVYAILETQLQFLMLEAYLCEIYHTDNVKNWKYLHDTHYQ
EEIGKSLRLCIAHHNVLKKMFKMVVNITDTAMPFFLLLGSLILISAFAFIINFVDTMTTILKIRIFISAIVMVSITM
LLSWIGQQLINVTSDIFFTLGGAPWYYWNLKNVKILLIFLTNCTKNESIILAGICLDYQMCISLFQLAVSYALVL
LNLRKSSLVZ

>TcOr222

MDKRDDPFIILRKMIFIEAKNCKIAKFCDAFLILFYSLVQLLDIYYMSKNFSISLLIRYSPITIMYLLIIIAAVISVGL
DKEIIEAYTVCCKIRWPMNVVKKQTQIKLKKKCQIINAGLSCTVPLFLVTIISTFPYFGSERDLFICVEVFEAYFG
EWSFIPYYFCFAASPFFYYHFFRITFVLVYAFLHAQLQYLLIEEYLFETYETDEAKGWKYLQDTRYQQEIGKSLQ
LCISQHIALKQFVKKTVDLVLIGMPFFLVFGVLLLTSLLAFITNFEDITSNILKIRILLAAGCSLCITIVFCWIGQQ
LINVTSDIFFSLGGASWYFWNRDNMKTLLMFLINCTENESVVFAGICLNYELFLSVVRLTVSYTLVLYNLQKQ
Z

>TcOr223

MTDFKDPFIMLRRIFIDVNSYKITKLCDFTVITFHSLVLCLQLYYMIRHFDVNLSIKYGPITAFFLFMTVSAVLSG
ALSQDIFRAVAFFEKISWSLDVIRKEARIKLERKCQVINTCISCILLFSSTTMVINLPFCENQRYFFISNQVFEEYF
GKWSVLLNVFYYSGVPYLGYHSVKPCFVFVYAILEIQLQFSLIEEYLLQTYETDYLESGEHLEDTQYQREIGEAL
RRCITHHVQLKKLIDMMVDIVLMYMPFFLVLGVFLLITCFAFIINFADTTTNTVKVQAFMFVVTALCNTVLFC
WNGQQLIDVTNSIFLTLGGAPWYHWNVENIKILLMFITNCTKNDSIVLAGICLDYKMFVSVFRISVSYALVLF
NLRKRSLVZ

>TcOr224FIX

MTEAGDPFIMLRWILLMDVSNNKITKYCNVFLTTIYSVVLCLQIYYIFKNYDTNLLIKYGPITISLLFMITVAVIS
LIMQKEIFKTVTFIRETCWPLNIIQKSGQIKAERKCRTINFYITSTFLLFLSAIIIHYPCFGSQRDFIICIEMFEEYFGE
WSSVLYYLYLIGVHFLYYRLFQTCYMFVYGMLEAHLQFFFLGEYLLETYQTDCLKRCKYLQDTRYQQEIGQSL
RFCIKHHIALKKLVKMALNLAFIGMPFFLVFGVLLLISCFTFIINFADTMSTILKIRIFMFASNTVCIAILLCWIGQ
QLIDVTSDIFVTLCGAPWYNWNLDNIKLLLMFIMNCTKNESIVLAGIRADYQLFVSLLRISASYALVLLKLRKC
SLVZ

>TcOr225

MKTSGDPFIILRWILLMDVSNNKITKYCNIFLTIIYSLVLCLQIYYMFKNDDINLLIKYGPITILLLFMITVAVISLI
MQKEFKTVTFIRETCWPLNMIKNNGQIKAERKFRTISFYTLCTVLLYLSVIIINYPCFGSQRDFIVCIEMFEEYFG
EWSSVLYYLYLIGAHFLYYRLFQTCYMFVYGMLEAHLQFFLIGEYLLGTYQTDCLKRCKYLQDIRYQQEIGKS
LRFCIKHHIALKKLVKMVVDLAVIGMPFFLVLGVLLLISCFTFIINFADTMSNILKIRIFMFVSSSVCNSILLCWI
GQQLIDVTSDIFFTLGGAPWYNWNLDNIKLLLIFIMNCTKNESIVLAGIRADFQLFVSLLRVSASYALVLLKLR
KCSFVZ

>TcOr226

MAKTGDPFIMLRWILLMDVSNNKITKYCNIFLTTVYSLVLCLQIYYILKNYDINLLIKYGPITTLLLFMITVAVIS
LLLQKEIFETDTFIRETCWPLNIIQKSGQIKAERKCRTINFYIGSTFLLFLSVLILNYPCFGSQRDFFICIEMFEEYF
GEWSSVFYYLYFIGSHFLYYRLFQTSYLFVYGMLEANLQFFLIEEYLLQTYQTDCLKRCKYLQDTRYQQEIGKS
LRFCIKHHIALKKLVKMVVNLGVLAMPFFLVFGVLLLISCFTFITNFADTMSNILKIRIFMFVSSTVAIALLLCW
IGQQLIDVTSDIFFTLGGAPWYNWNLDNIKLLLMFITNCTKNESMVLAGIRADYDLFVSLLRISASYALVLLKL
RKCTFVZ

>TcOr227

MPESIKLFSEESSTFRTMAEFHDPFIMMRKILYNKSKNYNLAKLCHVLLIIIYSSVQCLQIYYVFKNFSINLLLRY
APTILFFIFITGAVLSLIFMENDILEIVTFLDKICWSFNMVRKDAQMKLKRKCQVINMCILFVLLLLLSTLTINAP
CFGSQREVFIFIQIFEEYFGEWSFIPYYFYFTAFPFLYYNFLKLWMSFVYMVLEAQLQFILVEEFLFETNQVNRLK
GWKYLHDTGYQKKIEKSLRLCIIHHNALKKYVRMTLNVTLKAMPFFLILGILLLISMFAFLINFADTITTMSNIL
KMRIIMTVTVTMCIAALLCWLGQQLIDTTSDIFASLVGAPWYFWNLGNIRILLMFLTNCTKNESVVLAGICVD
YKLLVSMLRTSVSYALVLFNLRKSSVVZ

>TcOr228

MTDSRDPFIMLRRIFIDINSYKITKLCDFFVVTFHSLVLCLQLYYTIRNFDVNFLSRYGQTTVIFLFMTVSAFLSG
VLEKDIYRILTFSKTFLWSLDVIGKDARMKLERKCQMINMCITCLLLFLSTALVINMPFLGNPRQFFISIHVFEE
YFGEWSSLLNVLYFASMPYLAYHATKPCFLFVYATLHMQLQFSLIEEYLFQVYEIDYLKSWKYLQDVRYQREI
GNALRRCIIHHIALKKLIKIIVDIVLVYMPFILILGVLLLITCFAFIINYANTTTNTIKIQMFMFMASALLIAILFC
WNGQQLIDVTSNIFFTLGGAPWYYWNRENIRILLMSIMNCTKNESIVLAGICLDYKMFVSIFRTSVSYALVLFN
LRKRSLVZ

>TcOr229

MSARPLHLRNFPYYFLKVLVFDFEQYSAGKVLSYFCAIVHSISIFLQMHYLVKNFTKETMFQYGCVLTVLTYC
VVALFFAIASGNFVEKLESEISSFVWPLDICGEDVKAAILKRAFYTSLVAYITIIAFPIFSVIMFPVLGDQSDMFLC
VRVFNEYFTKWSQIPISLYFYSFPVIAFSGIRLPGMLLYAILITHIQMFLLNRRIEQISELSNQRRVFETLCSCIELQ
AKLKRLIRNVFQLVYIAMPIFILLGAVSSVFVLFFVVNSLETASYFLVLRMGCFFGANVLVVFIFSQSGQSFSDET
GRIFDTLVMCSWYNWDKRNKKVLLMFLANSLEPMSITIAGITLDYKFALAMLRTSCSYALVLYQMKNZ

>TcOr230

MREKPLHLSHFPYYLLKIMLCDTEQYRLGRFLSYSCAVIHSISLLLQMYYLIDNFNKETVSRYGCVVIVTTYCV
VALIYEILYAQPSVSMMSQQISTLWPMDACGEKVKQMILKRAFFTSVVTYSILFSFPIFGIIMFPLWGDQSDMFL
CVRVFNEYFTKWSKVPIYLYFCSFPVLTFSGIRLPGMLLYAILITNIQIILLNQKIAHISDLGDQRLVFGTLCSCVS
LQIKLRQMLNKVLQFVYLVMPVFLLLGALTAISVLFFLFYSLENPSDYLMIRLACFLGGNILVVFTFCESGQAL
SNDTGRIFDILLTCPWYKWDKKNKNILLMFLVNSLKPMSITIAGITLDYKLAVTLIRTCCSYALVLYQMKNZ

>TcOr231

MEVTGKKVTSGDPFITLKKLYIDLGYHKVTKLVNVFFIVFYGFVYLLQIYYLIVHFNFEIIAKYSTILLLSTYLFNV
MIFSIIYEKCILDAYKTFSQIAWPCDNASKPLQIIILQRSKTIKYLNYFFLGFIFFMACINWPWLGDQNDFLLCIQ
VFKKYFGSWSPLFLLFYYLGFPIIGYSAARIFFIILYGVLHLELQIRLITELFCKISRNATLEDVRNAKYQRDVYWT
LREGIRHDTALKKVLFDLNKEVKHGIPVFLIVTLLCSVSIFFFAITYLESMGFGIQIRTIAFVGAVIFVLFSYSLLG
QHLINQTSLLFDQLYECPWYTWNVKNRAIYLNFMLNTVRPIKITYAGICIDSRFFLSITRIILSNAFMLYQLRNS
Z

>TcOr232

MLDLNDPFAVLKKFYIDFGYHKIVKSCNTFFITLYSLAYALQIYYLIYHFTPDIIQKYSLALLMSIYLLSTLIFSAV
YEKTILQTYDMFIRVAWPHTNVSQKLETIIQKRVTIIKILNNFFVSAICVMCVFNMPLFGDQSDFLLSIQVFGEY
FGDWSFIPSFFYYLGFSILGYNAARMLFILLYGVLHLEVQILLINELVSKFSEINTLDDFGNEEYQSCVYTILCDCI
THDVVLKKIMSDLSRKVQNGIPIFLIVLLFCLISLFFFTLTYMGTLSFIMQFRMVAFLLTIIVVIFSYSMVGQHLL
NQASLLFDELLKCPWYVWSTNNHKIYQIFLLNSIRPIKIIYAGVCLDGGFFLSMTRITFCNAYMLKQLRDSZ

>TcOr233PSE

ZTVQDIVKFGFPILGYNAARMLFILLYGVLHLEVQILLINELVFKILEIANLDDFENEEYQSCVYMILRDCITHD
VVLKKXIMSDLSRKVQNGIPIFLIVLLFCLISLFFFMLTYMGTLSFIMQFRMVAFLLTIIVVIFSYSMVGQHLLNQ
ASLLFAELLKCPWYVWSTNNRKIYQIFLLNSIRPIKIIYAGVCLDGGFFLSMTRITFCNAYMLKQLRDSZ

>TcOr234

MQQAALRNFPWHYIKRIFIDFGYHRTMKIFTIVYFILYSGSLLLDLYYLFNNFSIAAMVRYGCMIMLISYVIAG
MLFCFIFEKQLLNLLSEAETIFWPPEMITSELPKFIHRTNVLNYFIIAWFGLLGVILFPVWGDQSEWFLNVWAY
KAYFGSWWYIPYNLFYYSQPMAAWTCVRLPFIMMYFSLQIKLQIFLLNQQILEIPKGHNTNSETAPDDLSYQE
AVSQKMCLCISHNVKIKRWTKSFLRKVIQAMPVFVLLGILGSIFVTFSVLYSFESTSTILKIRLVVVVGCTILSVY
MFVEGSQRLCDESSQMFEMLAYSPWYLYNKNNRRILLTFMTNTLEPITITWGGIILNYNFGLTMLRMSFSYAL
FLYNIHZ

>TcOr236

MMSEFNDPIIVWRMIFMINFKKHKITKFCEIVLIEIFSLIHCLLLYYIFTNFSVNLLIRYGPTTLFGIFIIAVTIFSVA
LKKKLSGAIDILDEICWPLNMIGKEAQLKLERKCRMRNMCTAFVVLINLTVIIVNYPCFGDQRDFFICIKVFEE
YFGEWWSIPYYFYFITIPFFCYNYHKLCFTFVYAVLETELQFFLIEEYLLETFKMGYLKRWKYLENTQYQQELG
KSLRFTIAHHIALKKMVKVIVNVTVNGMPLFLLLGFLLYISCFTFVINLADSMTNILKIRIFVCGASCVSVTVLL
CWNGQQIIDVTNSIFSTLIGAPWYFWNLDNIKILLMFITNCTKNDCIVLAGICLDYKLFVSIVQISISNALVLFKI
RNASASZ

>TcOr237PSE

MTFEFNDPFIAWRMIFMINVKTHKITKYCELVLIVNYSLVHCLLLYYMFTNFSVNLVIRYGPMIIFYIFTIAVTIF
SIAFEEEICEGITLLDEISWPLNIIEKDAQIKLGRKCRIINLWMTFVLLNILSAIIVNYPYFGDQRDFYICVKVFDE
YFDEWSSIPYYFYFIASPFFHYNMFKVCFTYIYAVLESELQFSLIKEYLLETYKVDNLKHWKCLNDTRYQQKLG
KSFRLCVAHHIALKRMVRVIVKVTVNGMPIFLLLGFLLYISHFAFIISLADTMTNILKIRISLVVASCVGMSVLL
CWSGQQIIDVTNSIFSTLVGAPXYYWNLDNIKIFLMFITNCTKNDSIVLAGICLDYKLFVSIVRVSFSYALVLFN
LRKTSVSZ

>TcOr238

MMSEFNDPFIVWRMIFMINFKKHKITKFCEIVLIVIFSLIHCLLLYYMFTNFSVNLLIRYGPTTLFGIFIIAVTIFSV
ALEKELSGGIDILDEICWPFNMIGKEAQLKLERKCRMRNMCTAFVVLIILTTIIVSYPCFGDQRDFFICIKVFEEY
FGEWWSIPYYFYFITIPFFCYNYHKLCFTFVYAVLETELQFFLIEEYLLETFKMGYLKRWKYLENTQYQQELGKS
LRFTIAHHNALKKMVKAIVNVTVNGMPLFLLLGFLLYISCFTFVINLADSMTNILKIRIFVCGASCVSVTVLLC
WNGQQIIDVTNSIFSTLTGAPWYFWDVDNVKILLIFITNCTKNDSITMAGICLDYKLFASLLRISFSYALVLFNL
RKSSLSZ

>TcOr239

MVEFNDPFIVWRLIFMINFKKYKIPKFCEIALIVIYSLIHCLLLYYIFTNFNVNLVIRYGPIMIFYIFMIAATIFSVA
LEEELSGGIAYLDEISWPLSTIGKEAQLKLKRKCRIINMCIAFVLLIILSEIIVNYPSFGDQKDFFICVKVFDDYFG
EWSSIPYYFYFTASPFFYYYYFKLCFTYVYAVLETQLQFFLIEGYLLQTYKIDNLKRWEYLKDTRYQQELGKSLQ
FAIAHHVALKKMVNVIVSLSVNGMPLFLLLGFLLYISCFTFVINLADTMTNILKIRIFLLGASCVCVTVLLCWN
GQQIIDVTNSIFSTLTGAPWYFWDLDNVKILLIFITNCTKNDSITMAGICLDYKLFASLLRISFSYALVLFNLRKS
SLSZ

>TcOr240PSE

MSEINDPFIVWRMIFMINFKKHKITKFCEIVLIIIYSLIHCLLLYYMFTNFSVNLLIRYGAMTIFYIFMIAATIFYVA
FEEELSEGLTFLDEISWPLSTIRKEAZLKLERKCRIINMCIAFVLLMILSEIIVNYPCFGDQKDFFICVKVFEEYFG
KWSFIPYYFYFIASPFFYYYYFRLCFTFVYAVLEAELQFFLIEGYLLQTYKMDYLKRWKCLKDTRYQQELGKSL
RLCIAHHVALKKLVKMIVKITLNGMPLFCCLGFFFISXYFTFVINLADSMTNILKIRIFLLGTNCLCVTVLLCW
NGQQIIDVTNSIFSTLIGAPWYFWDLDNIKILLMFITNCTKNDCIVLAGICLDYKLFASLXLLRISFSYALVLFNL
RKQNNSZ

>TcOr241FIX

MMAKFNDPFIVWRKLFLINVKKYKITKSCEIALIITYALVHCLLLYYMFTNFSVNLLIRYGPVTIFYIFMLAATIF
SVALEEELSEGLTFLDENCWPLNTIGKDAQIKLERKCRMINTCTALVVLIILSAIIVNYPCFGDQRDFFICVKVF
EEYFGQWSFIPYYFYFITSPFFYYNSFKLCFTFVYAVLEAELQFFLIEGYLLQMYKMDYLKHWKCLKDTRYQQE
LGKSLRVCIAHHIALKKSVKMIVKITVNGMPIFLLLGCLLYISCFTFVINLADSMTNILKIRIFLLGASSVCVTVL
LCWNGQQIIDVTNSIFSALTGAPWYFWDLDNVKILLIFITNCTKNDSITMAGICLDYKLFASLLRISFSYALVLF
NLRKSSLSZ

>TcOr242

MGNFDDPFLVLRMLLFIDIINYKITKFCSVLLITFYTIEHCLEIYYIANNFNINLLIRYGPVTTFSLLLIVAAVFSG
ALGNEIFKIAAFHCKISWPLDMIRNGAQTKLKKKCQTINGCLLGIVLILVSGLSINLPYFGNQRELLICIQVFEE
FFGEWSFIPYYFYFVGYPFLYYYFFRMCFGFFYIFQEAQLQFLLIEEYLLKTYQTDDLKHWQYLQDTQYQEEIG
KSLRLCIAHHSNLKKLVKMGVNITVTAMPLFLLLGILLLISSFAFIINFAGTMTNILKIRVFLFFASTISITILFCWT
GQQLINVTSNIYFTLGGAPWYYWSLENIKILLMFLINCTKNESIVLAGIRLDYQMFVTMCRISFSNALVLFNLR
KRSLVZ

>TcOr243

MGEHDDPFSVLRMLLYINIDSKITNVLTVLLVIIYTLEHCLEINYMITNFNLNLLIRYGPVTTFSLLMTVGALIPV
ALGKEIFEVVAFHRNTCWPLNMIREDAQTKLKRQCYVVNGCLFVIVLLLLSTLIIYLPFFGNQRELLLCIQVFE
KYFGKWSFIPYYFYFVGFAFLYYQFFRISFGFVYIFLETQLQYFLIEEYLFETYHIDNLKHWKYLQDTQYQEEIGK
SLRLCIAHHNDLKKLVKTSVKLTITAMPVFLLLGVFLYISSFAHIINFADTMTNILKLRMFLFLASTMSITIMFC
WTGQQLINVTSNIFFTLSGAPWYYWNLENIKILLTFLTNCTKNESIVLAGICLDYKMFVTMCRISFSYAVVLFN
FRKQSLIZ

>TcOr244

MMCEFDDPFIVLRTLLFINVSYKVTIFWSIFIIIFYTIEICLEIYYMLTNFNINLLIRYGPITTFFLLMIVTAVLPVILG
KEIFEVLAFQCNTRWPLNMIRKDAQTRLKRKCQTINGCLLCILVILLSALGVNIPYFGTQRELLICVRVFEEYFG
EWAFIPYYLYLAGFPFLYYHFLRISFGFAYVFLEAQLQFFLIEEYLFETYQTDHEKHWKYLQDTRYQQQIGKSL
RDCIAHHNDLTKLVKMSVNVTVTAMPIFLLIGIILYISSFAFIINFADTMTNILKIRVFLFLASTMSITILFCWTGQ
QLINVTSNIFFTLSGAPWYYWNLENVKILLTFLTNCTKNESIVLAGIRLDYRMFVSMCRISFSYALVLFKLRKRS
LVYZ

>TcOr245

MQSLIFFRYLSRHKDNYILVESFQHSQMAEFNDPFILLRTILFTNMNSYKILKQCNVLLNIIYLLIHCLLIYYMF
NNLNLNLLIRYGPGMLFFIVVTAAAVLFLHLEKKILEIVTFCDKICWSLSLIRKSAQTKLKRKCQIVNICVSFVL
LLLVSTVTINAPYFGNQRELFICIQVFEEYFGEWSFVPYYFYFVGFPFLFYNFMRLWISFVYAILEAQLQFFLIEE
VLYETYQIRYLKGWKYLQDTRYQQDIGKSLRLCIAHHNALKKLLKMVVNVTMTTMPFFLVFGVLLLISSFAFI
INFADTMTNILKIRMFMFALSIMCVAVVLCWIGQQVIDVTSKIFFALVGAPWYFWNAANIKILLMFITNCTQ
NDSIVLAGICLDYNLFVTMVRISVSYALVLFNLRKNSVAZ

>TcOr246PSE

MASCQQSKMAEFNDPCMFVRTIIFVDINSYKIIKAWNVLLIVIYSLIHCLLVYYMFKNLDLNLLIRMVILVIGG
AAFSVYIDKYILZVRTIFRKFVGLFNMVRKEAQTKLEKNVKSZISLYYSLFCFLZVHSLSTLRVLKIRGSFSFVLQ
VFEKYFSEWSFFPYYFYFLVFPLSYYNFLRFWMIFVYGLLEGQLQIVILEEYLCATYETDNLKSCKYLQDTRYQZ
EIGKSLRLCISHHIELKYLLKHYFZESCSIYYVPKFLLYIKVYSTXSYHSVTIILKIQIFISAFSMVSITIFLSWTGQQL
INVTSDIXFTLGGGLWYYWNLKNTKTFXNXLTNCTQIESVTLAGICLGFTLFISVXTTVSYAVVIYNLREISLVZ

>TcOr247

MYEFEDPFIWLRNSFHINESNNTMTKKIYVLLILIYSFAQCFQIYYMFKNFNMNLLIRYGPAIILFILVILVATLF
LYIEQDMFKALTFFDKISWSFSMIREDAQIRLKRKCLIINICILFILLLVVTTITIGAPYFGNQRELFICIQIFEEYFG

KWSPIPYYFYFVGFPFLYYNFFKAWTVFVYGILEVQLQFNLVEEYLIESNKINDFKGWKHLQDTRYQQEIGKSL
QLCITHHIALKKLVNKIINFTMTGMPFFLVLGVSLLICSLAFIINFAHTMSNILKIRILIFVATNVCLTILLCWIGQ
QLIDATSNIFSSLVGAPWYFWNLKNTKTLLMFLINCTKNDSIVLAGICLDYRLFVSISRITVSYALVLQKYZ

>TcOr248PSE

ZQLQSTKIVQCFTYCTILTHPLLMIFYMFKNLYINLVIRFSPGLLLFILVSEVIVGGSVFMGKDMQEIDKVFRKTR
WSLNMMTEDAQIMLKRKCSVINXFEYYLFCWLSCYVICYAAMLTINAPYFGNPSEIFICVQVFEKYFDEWSFIP
YYFYFLEVPLLYYNFFRLZMAFLYGLLERQLQCIIFEEYLCAIYETDNLKSWKYLQNTHYQEEIRKSZPLSIAHY
NNLKXVLDRPATAIXVSIMMRLSWIGQQLINVTSDXFFTLGGAPWYYWNLKSVKILLIFLTNCVKNESIVLFRL
SNVYCGAYILTCDFNVYFYILTDVTTRCFIZ

>TcOr249PSE

MMPEFSDPFILLRKMIFIKKSQNXTKFCDFLFIAIYTFAHCLQVYYLCKNFSLGLLIQYSPALICYTFVIVAAVLIF
YSEKNILEAVIFYDRICWSLSMIRKDAQTKLRRKCLIINICIVYVLLLILSTLIINLPYFGSQRELFICIQIFEEYFGK
WSFAPYHFYFAVFPLIYYNSIKMZISVVYTILEAQLQFILVEEYLFETNQTNNQKGWKYLQDTRYQQEIGKSLR
LCITQHIALKKLVKMIVNITMMGMPPFLVLGVLLLISSFAFILNFADAMTNILKKRILKFLACIVFNLRKSSIAZ

>TcOr251PSE

MSFRYFVSEKGRIVVVESFDQSKMAEFKDPCMIVRTILFVDMNSYKIIKTCNVLLNIIYSLIHCLLVYYMFKNLN
INLLIRYGPAILLFILVIIGAVFSVYMDEDILEIRTVSRETRWSLSVLRENAQKKLERKCZIINAFIFFILLLIVGTLTI
NAPYFGNQRELLISIQVFEEYFGEWSFIPYYFYFLGFLLLCYNFFSLWMTFVYGLLEGQLQFFILEEYLWGTYET
NDSKSWKYLQDTCYQQXNRKSLRFCISHHNGLKKXFLKMIENLSLKVMPFFLVLGVLILICSFSFIINFADTMT
TIAKIRIVISAISMVGITILLSWIGQQLIDVTSDIYFTLGGAPWYYWNQKNAKILLMFLTNCTKNESVTLASISLY
FTLFVSVVVQTSVSYALVLHNLRESSLLSSSQKZ

>TcOr253PSE

MDKFDDPFIMFRKMIFIEAKNCKLTRFCDVLLIVLFSLAHCLQIYYMWQNFTFSFLMYSYFSFXVIITAVLSVGL
EKKLZKRVQFVVKFVGLZISQRNKLKLNLXKNCQIINGGILCRVLLFLSAVISSFTCFGSQZEFSICVEVFEEYFG
EWSFFIQHFYFTVAPFLYYHFIRVCYLFVYALLQAPIQYFLIEEYLFEIYQTDDLNGWRYLQNTRYQRKIGKALL
LCITHHVALKKYVTMSLNLLLIGMSFFLVLGVLLLISSFAFIINXFADTMSTILKIRILFFAASSVCLIMAFCWIGQ
HLZMRFTNEIFWSYFWNRENSKILLMFLTNCTINDSVVLAGIRLDYQLFLSVRTLZLVVRLTVSYALVLFNLH
KSSLVZ

>TcOr254FIX

MDKLDDPFIVLQRLIFIEAKNcklaRFFDVLLIVLDSLAHCLQIYYMCQNFAFSLLIRYGPVFIFFLLVIVTAVISVG
LEKEIIETYAVYCKTCWPLNIVKKQTQIKLKKKCQIINRGILCSLVLFLAAVISLFTCFGSQREFSICVEVFDEYFG
EWSFFIQHFYFTVAPLLYYHFFRVCYLFVYALLQAHLQYFLIEEYLFETYQTNDLKGWRHLKDTRYQQKIGKS
LLLCITHHIALKKFVKMTLDLVLIGMPFFLVLGVLLLISSFAFIINFADTMSTILKIRILFFAASSVCLTMTFCWIG
QQLINATSEIFWSLGGAPFYFWNRENSKILLMFLMNCTNNDSVVLAGICLNYRLFLSVVRLTVSYALVLFNLH
KSGLVZ

>TcOr256PSE

MTDKLSYXPFNLAEKTFIEGKHCKLARFCDVLLIVLYSLAQCLNIYYMFQHFNLGLLIRYGPVLVFSLLVIATSL
ISVALEKEIFELHMVYRZMFWPLNFVGKNAQAKLTRKCQFINRCISCSLLLXFFGSQRDLFICVEVFEEYFGZW
SLFPYYLYFAATPFLNYHFFRXFVYAILDAQIQYFLIEAYLFETFQTNDLKGWKYLQDACYQQGIGQSLRLCIE
NHIALKKFMKMSLEFVLIGIPFFLVFGVLLLISSFAFITNFADTMSNILKIRMLIFATSSVCITMVLCWTGQQLIN
LTSEIFLSLGGAPZYFWNRDNMKILLMFLTNCTINESVVLAGICINYEFFLSLVQLIVSYTLVLYNFHKSGIVZ

>TcOr258PSE

MNNERKDPFIMLRKMIFIEARNCKLARFCEVMLIVLYSLAQCLQIYYMCRNFNLNLLIKYSPAILCFLLEIGTS
VISVGLEKKIFDAYTVCCNIGWPLDFLKKDAQSKLTRKCQIIKRCISCSLLLVLITEISIFPCFGSQRDFFICVEVFK
EYFGEWSFIPYYFYFAASPFLCYHLSRITFVFVYAFQHVQLZYLLIEEYLFETYQTDDMKGWRHLKDTRYQQKI
GKSLLLCITHHIALKKFVKMTVDLVLIAMPFFLVIGVLLLISSFAFIINFAGTMSTILKIRILLFAATCVCITMTFC
WTGQQLINVTKEIFWSLAGAPFYFWNRENSVILLMFLTNCTNNDSVVLDGICLDYKLFLSVVKFAVSYSLVLF
NLRKSSLVZ

>TcOr259

MMPEFNDPFLVWRMIFTINFKKFKITKFCEIVLIVIYALVHCLLLYYTFTNFSANLLIRYGPVMIFYIFMIAATTF
SIALEAELSEVITFLDEICWPLNMIAEDAQVKLQRKCRIINMCIAFLVLIILSAIIVNYPFFGDQRDFFICVRVFEE
YFGEWSFIPYYFYFAASPFFYYNYFKLCFTFVYAVLEAGLQFFLIEGYLLQTYKVDYLKRWKCLKDNRYQQEL
GKSLRLCIVHHIALKKLVKMIVNLTVNGMPIFLLLGSLLYISCFTFTINLVNSLTNILKTRIILMGASCVGVTVLL
CWNGQQIIDVTSSIFTTLVGAPWYFWNLDNIKILLMFITNCTKNDKIVLAGICLDYKLFASILRISFSYALVLFN
LRKASVSZ

>TcOr260PSE

MMSEFNDPFIVWRMIFTINFNXLYYTFTNFSANLLIRYGPIMIFYIFMIAATTFSIALEAELSEVITFLDEICWPLN
MIAEDAQVKLQRKCRIINMCIAFLVLIILSAIIVNYPFFGDQRDFFICVRVFEEYFGEWSFIPYYFYFAASPFFYYN
YFKLCFTFVYAVLEAGLQFFLIEGYLLQTYKVDYLKRWKCLKDSRYQQELGKSLRLCIVHHIALKKLVKMIVN
LTVNGMPIFLLLGSLLYISCFTFTINLVNSLTNILKIRIILMGASCVGVTVLLCWNGQQIIDVTSSIFTTLVGAPW
YFWNLDNIKILLMFITNCTNNDKIVLAGICLDYKLFASILRISFSYALVLFNLRKASVSWNNQNZ

>TcOr261PSE

MEVTGKKVTSGDPFITLKKLYIDLGYHKVTKLVNVFFIVFYGFVYLLQIYYLIVHFNFEIIAKYSTILLLSTYLFNV
MIFSIIYEKYILDAYKTFSQIAWPCDNASKPLQIIILQRSKTIKYLNYFFLGFILFMACINWPWLGDQNDFLLCIQ
VFKKYFGSWSPLLLLFYYLGFPIIGYSAARIFFIILYGVLHLELQIRLITELFRKISRTIFLNRKHHLCLYTYQZ

>TcOr262PSE

MEVTGKXVTSGDPFITLKKLYIDLGYHKVTKLVNVFFIVFYGFVYLLQIYYLIVHFNFEIIAKYSTILLLSTYLFNV
MIFSIIYEKYILDAYKTFSLIAWPCDNASKPLQIIILQRSKTIKYWNYFFLGFIFFMACINWPWLGDQNDFLLCIQ
VFKKYFGSWSPLFLLFYYLGFPIIGYSAARIFFIILYGVLHLELQELFCKISR

>TcOr263

MWNNNPFIVIRTIFLDINNYKIVKFCYVSLTVFYSLVHCLQFYYIIKNFNLNLIIRYGFITSLLSYVLAAGILSLVV
EKRIRKTQIFFDEIGWSLNIVGKDAEMKLEKKCKLINISIYAIMLFLVITLLVNLPFVGSQRDLFLSIQVFEEYFGK
WSEILDRLYFTLAPFLSYHGARLSFTCIYAILQVQVQFSLIGEYLFETYQVDDSKSWKYLQDTRYQHDIGESLRL
CVEHHVALKKSIKMMVDVALTCLPFLVLLGLSTLISCLAFIMNFWDTMDNILKLRIFMWAAWIVLITIMFCRS
GQQLIDATSDIFFTLGGAPWYYWNLDNIKILLTFMANSTKNDSISLAGICLDYPLFVSVANTTVSYALVLYNL
RESSLDSSNKKZ

>TcOr264

MVYLKDPFITLRVMFLNFNKYKIVKCCDFSFIIFYSLVFCLQIYYLISYFSANPLIRYATTILLVLWGIVGAILSVTL
EKQILEATAFLDEMCWPLNMVRKEAQTKLERSCRIINIYITCSLLLILITVVFNMLCFSSQRDFFINIQIFEEYFGE
LSHVFNGLYFTGFPYLCYHGARLCYVFVYAILQIQLQFSLIEEYLLQVYEIDCLKSWRYLRDTRYQQEMGKSLR
LCITHHNALKKFVKMINDMSLICMPFCLVLGVLILISCLAFVINFGDTLTIFVKLRILIFVVSCLCVLSVFCWSG
QQLTDVSSYIFLTLARAPWYYWRLENIKILLTFSTNCTKNDSIVLAGIRLEYMLFVSMLRISCSYALVLFNLRKZ

>TcOr265PSE

MADFRDPFIMLRRIFINVNSYKITKLCDFSVVTFHLLVLCLQILYMIRNFNVNLLIRYGPTTALFLLMIVSAVLS
GALENDIGTTLGFSQKICWSLDVIGKNARMKLEXEMINVTISAILLLLSSTLVINLPFFGYHKDVFMCIQVFETY
FGTWSYLLNVLYFTVLPILSYHGVKPCFIFVYAILQIQLQFSLIEEYLFQTYEVDHLKSWKYLQDARYQQEIGNS
LRRCIQHHIALKQFVKMMVDVVLIYMPFIFVLGLFLLITCFAFIINFAETTTNIIKIQVFMFVASTLGITILFCWN
GQQLKNVRSNIFFTLGRAPWYFWNLENIKILLMFITNCTKNDSIILAGICLDYKMFVSVFRTSISYALVLFNFRK
RSFGZ

>TcOr268PSE

MMDDFNNLFIMLRNTTFINARNNKIVKFCDIFFIISTHSFSFLTYTNYISKNFNISLZFPVINTAVISVGLEKEIFE
AYKVLZNLLXSLNIIKKHPQIKLLKKCHIISGSIVSCSLLLFZTTIISNFPCFVNLRDFLFVFLNNILVNXSLISSHF
YFAASLLFYYHFFRVVFVFVYEZLZAQLQYFLNEQYLVETCQADNSEGWKYLQDKHQIKLKKTL

>TcOr269

MAQQSNLEPSDSLKTLYLMTFGVMRLRLVKAILIIIFIFHSTIGVIQIIELLVTFDGNQLLQYGPFFLVSCSCLVTIS
SVFTAEKILTKLFIKLAVVRFWTLTRFPPEISKKIAFESKIVTGASIIALTVMFLFSTVNVPFFGDKNVIYGIMLIQK
SFGRTAKLFCFLYYGTLPILAYVAFISGGVLIYTLMHLRFYIFVINHYLERIVLQYDLVISDSHKLRDEEYQKSIY
DQLKICIGRHQFFKKIEKKLNAKLLPYLLLLLYLGTAAVLSMLFNILVDASKFGYFVAIGGMVVSVIVPLLLVH
VGQCLKDEYEKMFANTLKFPWIVWNSRNRKILFIFILNLQKSLCIGQPGLVLCEYSFIPSVLSFVWTFSTFIVQV
ASKSRZ

>TcOr270

MNYRKQEVMKDDPLKLVRIMGQDFLYIKFINILLKICLSYNIILLGFTVYYCLSTFDITEVWTYSWLMLQFPYFV
LILFTHIIKHGVVDDAMSVESPWSIDSAGEELSLKIKRESKLLNIFTVVNTVFVIVGVILFSYPQANDEQIIFFLYK
FREQFPYQNWIFSILNKLNICMIGLVVPAHSYQVIYVSQHWRFQMCMLNKYIQELDNYSNRGSIALLHNESY
QKEITARLKFCIKRHQEIIHVTAESVKIAGPFIVPFLITGLFLGTGQVLSILEVLDGVPIQLWRIACLSIATLICASC
FLLSGQSIESESERIFSTLIQLDWYNWNNKNRRLLLTVLANSLKPIQFKFATTFVINYVAGLNLCKMIYSAVSVF
TSVFZ

>TcOr271PSE

MDYRNPSPMKNDVLRIVRFLGQNVFYVNIVKSVLKISVCYSMIIWAYIIYHLFFVFDVNQTLNFVPTLLQMLY
FTLTQLTLIFKHGLADQILSVLPLWEIDSAGQETALKIRKEAKIYTFCTLVTTATAVVAVLLLTSPQDNADQVII
IFCKFRELFPYQNWVFARLSKLIYLLLILAAPAHTYQTIYVTQHLKFQLYLLNEYLKGLGSYKKMDRNALFYD
TEYQKLVRFRLTVCIKRHLEIINARNNAVGLMDNYIVPFIITGALLVAGLLLSAFHLVGEVHLPWWHCAALGI
PVFLCACYFFVSGQALESESERVMTTLMELDWYDWYSHNKKMFLFFFKXNTLKPLQISFVNVALNYVTGVK
VLKLVYTAVSIFGSFKZ

>TcOr272

MTRTPTDDTTFDLDNFMKNDSMKLVRVIAYDTLKFKITKLILFITFLVHFSTTLIQVYFVCVDFNVYFFVKYAP
AMFGSLFVMVSIIALFVTAETDMVVRVFRKAQLRKLTVEDGPSFHVFQKECKIFTVFFVLNLIIALFSGYLHAL
PDDDDREIFYAFAFFEDYCSEWKDFCSFLYRITFLPVAYVMYVPINVFVYAAIHLKSQIYYLKEHLIQINEGYDI
SNNNDLFYDENYQRIIREKMIYLYKIHVKLFLAALDIRKLIRGFIALFAIVGCLLGISILYFVMLFQGNLFDKFGR
LSTLTVVAFNSFAAVIISGQMIESSSSDDVDAIIYNCNWYDWNEENKRFFLLIRMATMHPFKLQFSQNYAVNY
QLGVAILKAMYSAFSLLKAIKNDFZ

>TcOr275FIX

MVHFPSQKRPLKDDPFQYLRRCLEPWGQKPSLLPLCLLILLIKIFFFTARTVFILKWMKEIDRFGNFSHMACTT
FGKRNRKFWDLGVGGPGLEKELEKRFKLINRFLFGHVSLGVFYVGLYAFFADVPIPKGRTRWLPVLASMPFD
QDQSPQYEILYVLMYWNLVVSILGHGVFDMVFIYSSQHLVGQFILLKALLRKLDYGFEGLEIVAKARSRQFQK
EIRKRIAICVQHHNLLLAYGNELKKIASMIFGVHVLSTSLTLILVGYILSKNLEKILQYSMLLSGVVSEALQFIIFA

VQSGEIYHKSVSVAQAAYQSNWYVFNAKAKRDLTLLILNSQKGISMYGAGLVTINNEILVSMIQKIFSSITLLR
SLGEQKZ

>TcOr276

MTMQFIVKRATRGIFHDLRVLKFISSDIFDIKIMKLCLFITFLIHLTACAITIHAFMFNNFSRREFISCAPVLFGCF
YGLLGLGTILFKPSMTRTLMLELKAWDITAADDAVSSRIKFEINVITVFCLVNYLLALVASFFYYMSFYGDEEIF
YLIRFLEDHCPNHKRVLIKLYKISFVLLGYVMVVHACQVLYATQHVRFQLILCAHFMANVTKQAKNIKDEH
LPDDNNYQNMIRERLKFCIIRHQEIRRFYFDKLEEMGNLIGGFALLGCFLGISFAMHMLTSEFLRYHFARTVSS
IIAGVTTFATVIAAGQSVETEVDISTRVVKEVKWYTFNESNKRSYMLMLLNSMQTYKIKFSENYSINYELGLSIV
RGVFSIVSVVVQLDYZ

>TcOr277

MDQVLEKFPENDWLRGVKFISSDIFQRKLVKAVLFMVLLVHLTASVITIRAILIKDITAKEFTFYGPVFFGCFYG
MLAIYIILFEKNFIANLSGELKMWSFRSAGAEITRQIRFESRVVTIYAIINFVMVVIASCLHITPLESDYETFYMIRF
FEDKIPDYANVCKTSYRSTFLVMGYVMMVHVYQIIYATQHGKFQIMLYLEYVKRVTQFNEKIGEKCLFYNESF
QKMVARKLKNCVIRHNEFLKYHRKNTREMSHWIVAFSLCGCLLGISVFFYILSGVIYREQYFRVAVLLTTAAS
TFVAFIVAGQSLESRVDNGYSVVSRIEWYNFSETNKKTYFLLLVMLMQPWKIKFSDKYSINYELGLSIVRGIYSII
SVMVNIRFDSZ

>TcOr278

MNQPQESFLKNDYLKVLKLISSDVFEPRLVRAILFVVFAVQLTASIITVRALLIKELTAKEFVLYGPVFFGCFYG
MLAIYIIIFQSSFITNMSQELEMWSYSSGGEEINRRVKFQSRVITIYALVNFLLAIVASYLYFSPLDSDNETFYMVR
FIEEKIPDYAKICKIAYRTTFLAMGYVMIVHSYQVIYASQHVRFQIIFFTEYVKKVVEFDEKISEECLFYNERFQTI
VGKRLQNCVIRHIQFLKFDRIKIKEMSNLIAAFSLCGCLLGISISFYVLSGIFYREHFLRVALISVTAVSTFFALILA
GQSMESKANSAHIIMNNIKWYNFNQSNKKAYLLLLMMSMKQYKIKFSENYSINYELGLTIVRGIYSIISVMAN
MHFDNZ

>TcOr280PSE

MDYSEKSLMQXRDCLKLLKVISSDIFQPKLVKLILLIVFGVHLIVDLLTLRALLVNELDFKEFVFYGPVFFGSFY
GMMALLTLVLKDDFISNLKQEFRLWPLDCAGDEIYSQIKFENKIIKIFVVFNCIVTFIGSYLYFLPLDSDNETFY
AVRFIEENYPDHRNLLHGLYRSTFLIFGYAMTVHVYQVIYNSQHLRYQIIIFTEYVASIGNPDKRKENELFYDK
GFQKVVYERLKFCIMRHQEFLVISNKKVGDMRVFIVGYSLCGCLLGISLTFYIFSGKFYREHFPRVAVACVGAV
ATFWAVITAGQAIESEYDSALSTLLGKIEWYYFNDSNKKNYLVMLINLMQPWKIKFSEEYAVNYELGLGIVR
AIYSIVSVVASMHFEVZ

>TcOr281

MDYSEKSLIQGDCLKLLKVISSDIFQPKLVKLILLIVFGVHLVVDLLTLRALLVNELDFKEFIFYGPVFFGSFYGM
MALLTLVLKDDFISNLKQEFRLWPLDCAGDEIYSQIKFENKIIKIFVVFNCIVTFIGSYLYFLPLDSDNETFYAVR
FIEENYPDHRNLLHGLYRSTFLIFGYAMTVHVYQVIYNSQHLRYQIIIFTEYVASIGNPDKRKENELFYDKGFQ
KVVYERLKFCIMRHQEFLVISNKKVGDMRVFIVGYSLCGCLLGISLTFYIFSGKFYREHFPRVSVACVGAVTTF
WAVITAGQAIESEYDSLLSTLLGKIEWYYFNDSNKKNYLIMLINLMQPWKIKFSEEYAVNYELGLAIVRAIYSI
VSVIASMHFEAZ

>TcOr282

MHDYCFEPLTKNDYLKTVRFLCCDVFEAKIVKLGLWITFGTHLIVSVVTVRALLYDLTINEFVHYAPVFVGSF
YGLLALWTILFRIEMVRDVRKQFKFWTIDCAGQEAHSRIKSEIRITTVLSVLNFIITLYASYWYVYPIEGDKEIYY
ALKFFEEYCPRHKMVLSVVYRATFPLLSYAMIVQAYQVIYTTQHIRFQAILFIEFVLNIGHQTKNLSEEKLFYDT
DYQKIVGERFKFCIMRHHEFIAFRRLKLNEMSNLIVGFSILGCLLLFSFGLFVLTGKLHREHFWRFGLGSLAAV

CTFGSVIWAGQSIEIESENVVNSLNSVKWYTFDENNKRNYIIMLVNTMQPYKLKFSENFSINYSLGVSIVRAFFS
ILSVAAKLYFNHVZ

>TcOr283

MLKFEPKPTTKDELLWVVRTIYVDLFRNKLIQFALKMLFYGSIIMAIYQGVLFLYEFEIHYFVKYSSMYCFTCFI
LLAAYSVPIIAEVATTAFTTIKCWKIDSGGALVENKIKQEAHFTNIITAINCIFGLMVLVLFIVPFEDDNDFYFLF
IAFEKYFPQWQQLLKWGFKAFFPCITILLQAPFYIVIYACLRIKFELYMWMEFLKNLNIVYEKSDICELVHDSEY
QTEISKRLRFCIERQEHIYRSLLYGKKYVQQLDIYIFAYAILGSLGGISIIFVCISFEGNFFQGTYLRLSALTFVTLTT
FMHVIWAGQSVETTSSDSYDILKQCDWFLWNLKNRKTYLMCLNYTQRPLKAQFTQNVSINYVLGFSVVRTV
YSTLTALNSLRKASKZ

>TcOr284

MISFHKKAALNLDQEDKLWFLRKIYIDIFTSKISKILLSLLLINGIFLMLVQAVLFLEKFETTYLLKYVSSYSAAFF
VLACLYALPLVAKTIKLKFIQQFQLWPIDSASDEIKNQIKKTAFFVNFYTVVSLAVGVMCATVFAIPTDDDTDF
IFPIALMEEFIPEWKNFLLWCYRLAVFCFIVILTGPFFIVVYVIHNIRFQVLMFLHYLNNLNSGYTQRTINDKKY
QNVIKKRLQFCIKRHVHITSIINNNRKELYDFIVIFAVTGVLVLISVLIFVFSFQGSFESQNIRVAVFTLACMLTFL
HVIAVGQWIENVTSEVFEILKTIDWTCWNLANQKTYLIFMQNTKNHVKVQFSENISLNYELGVAMLKSVAS
MISVLHQLKNIDYSKSNZ

>TcOr285

MISFNEKLLLDSNQKDELWFLRKVYIDIYTTKIAKILLRLLFINCIFLMSVQAVLFLQKFEASYLLKYVAGYSGSF
FVLACFYAVPIMVKTMKVKFIAGIKKWRVDSASEKIKNEIKTTAFIINIYTGVTVVLGVFCSLLFSIPTEDDTDFI
FSIALMDRFVPEWKSVLMWPYRFSLFPLAVILTTPCFVVIYVVHHARFQMLLLLHYLKNVNSGHMTQSIGNA
RFQKEIRKRLKFCIKRHNHIITSLNQCRIDLYNLIIVFAVTGGLMLISILIFLFSFQGSFKSQYVRVTAFTIEYILTFS
HVIVVGQMSENVTFEFFQILKTVDWNRWNLSNQKTYLVFLENTQNHFKIQFSENIALNYELGVSILKTVASM
LSVLNQIKSIDYSKEKMZ

>TcOr286

MSVQKVSPDNLWLSAKICLHIFQYKAIKIILKILSLSIIILTCIQTFLYLKRFDSAYFMKYLPVYAGSLFILASIFCIE
HISHVILSTVEEFEFWDYADSKPEIRNWIKWEALYINTFMVVDAFVAYLSGIFHAIPLDEDYEIFYPLPIFQEFFP
DWTNVLGWLYRSSFLIVPVVMTAPSLMIIYFTSRLRFQMFLFMDILENISEGYDISEANDLIENSTYQKEIKERL
KTCIKRHNEFLSAGGQVMKNGQLFILIMSAAGVILGVSIIFFLFSFEGSFEKRYPRLVTLVISTGLTFTHVIIAGQL
VENIATRLYEILHFMDWHSWNQENRKILLIFMHNAQQELQIKFLDEVAVNYQLGISIGKAVYSMISVLSSFKN
LEESYNZ

>TcOr287

MISFEEKHLTNVRESDVLWLGRIMSLEIVQYKPMRFILNIIAVSIIALTLIQTYLFLQKFDGLYLIKYASVYTASLF
ILFSIIAAPFLTKFSTEALNNLEYWPIESAGAQIEKQIQREAIYINTFFVVNMVVSLISGVAHMIPLDDDKELFYP
LAIFEEFAPKWKNWLEWGYRLSFLVVPVVMLNSSYVGIYTLSNFRFQISLFNHLLKNINFPLNDNEQTIELMD
DQKYQNEINKRLKFCIKRQTHLYKVAHYVTGKVKHLSFFVAILTILLLIAVIVFLFSFQGTFENRYFRIITLVLTA
GNTFIHVIIMGNRIEEETEKIFENLKSLNWSSWNLQNRQVYLIFLHNNEEHFKVPISENASVNYELGISMAKTI
CSMVSVMSQLKNIDYSKNZ

>TcOr288

MFSFEVKLDENLFKNDVLWLSRKLCLDYYNTKLVKIILFVLSIGVAILTIIQTFLFLQRFDGRYFIKYAPVYAGSF
LIFLSVEHIPFALMLINSFKTITFWRIDSCGPEIEQKIKKHAMWTNICLISCTVVGLVSAIFHAMPLEDDDELFYP
LAMFEEFMPQWKNLLSWIYRSSFLIVPFSMPIPVYIAIYVITKSYFQILLFLSCLENLNTGFDTTSNHLLIYNNRY
QNTVKKRLVFCIKRHAYFSRAMNVHIKKMYVTIATFSIMGVILSVSVIAFLFSFQGNFENRYIRITTLVFTMVTV

STHILYVGQLIEDAAFQVYTTLKTVDWNNWNLENRKLYLIYLQNAQIIFSIKFTQDVSINYRLGFSMAKAIYS
MISVMSKLRNVDYSKIZ

>TcOr289FIX

MLNFEEKLDENIFKNDVLWLSRKLCYDYYNTKIVKIILFVLSIRVAILTLLQTLLFLQRFDGRYIIKYAPVYAGSF
LIFLSVKHIPLSLMLINSFKTITLWRIDSCGPEIERKIKKHAMWTNICLISCTVVGLVSGIFHAMPLEDDEELFYP
LAMFEEFVPQWKNLLSWMYRLSFLTVPFSMPIPVYIAIYVTTKSYFQILLFGHFLENLNTGFDTTLNHLLIYNN
KYQNTIKKRLVFCIKRHAYFSRAMNELIKKTYVTIATFSIMGVILSISVIAFLFSFQGNFENRYIRVTTLVFTIVTV
STHILYAGQLIEDAAFQVYTTLKTVDWNSWNLENRKLYLVCLQNAQIIFSIKFTQDVSINYRLGFSMAKDIYS
MISVMSKLRNVDYSKIZ

>TcOr290

MTAMLNFEEKPDENIFKNDVLWLSRKLCFDYYNNKIVKILLRVLSIGVAILTILQTCLFLYRFDGRYFIKYAPLY
AGSFFICVSVEYIPLSLTITRYFQSITFWEIDSGGPDIAKKIQKHAMCTNVCLVSCTIVGLVSAIFHIIPLEDDDELF
YPLAMFEEFVPQWKNLWSWMYRLSFLTVPFSMPIPVYIAIYVITKSYFQVLLYLPFLENLNTGFDTTSNHLLIH
DHNYQNTIKQRLVFCIKRHSYFSREMNSLNHRMYVTIATFSVMIIVIGVSVFAFLFSFQGTFENRYIRITTLVFTI
ATISIHVLYIGQLIEDACFRVYQTLVMVDWYYWNIENRKLYLVFLQNAQIIFKIKFSQNISISYQLGVSILKTIYS
MISVMSNLRNVDYYKNZ

>TcOr291PSE

MFNYEEKPDENIFKNDVLWLSRKLYFDYYNTKIVKILLRVLSVGIAILTVIQTCLFLYRFNGRYFIKYAPIYAGSF
FIFLSVEHIPLSLELANSAKNVTFWKIDCCGPEIERRIKKHAMWTNICMISCTVVGLVSGIFHALPLEDDDELFY
PLAMFQELVPQWKNVFSWMYRLSFLIVPFSMPIPLHVAIYIITKSYFQILLFMPFLENLNSGFDTTSNCLLIYNQ
KYQNFIKKRLVFCIKRHSYFCKAMNELIKKMYVTIATFSIMGIIIGVSVLAFLFSFQGTFENRYIRITTLVFTIATIS
IHVFYVGQLIEDACLQGYKTLVMVDWYZWNVENRKLYIVILQNTQKIFKIKFSQNVSISYQLGLSIAKAIYSMI
SVMSKLRNVDYSKNVZ

>TcOr292

MQPNLQKNDILWLIRKLTFDLFQLKITKMFLIITSVSIILLTIIQTFLFLKKFNGYYFIMYSAVYTGSLFILVSSLSV
LPISKLIKTAWVKFSFWEINSATPKIERKIRKEIFYINCVVFFNTIVAIISGIFHAIPLQDDEELFYPLAIFETYTPEW
KDWFSGIYRASFLPMPIIMVAPAYTVVYLCAHMRFQFCLLLHFLENINPDNENISDKKYQAQIKERLHFCIKR
HIHLFSKSRPVLEDLKKFVFVLTLCGTIFCISIIIFHFSFQGTYEGRYPRIITIIIAASITFFLSILPGQLIENTSSEIFEVL
RNTNWVSWNEQNKKLFIILLLNTRQIYKIKITENVSLNYELGVTMAKAMYSMISVMKQLZ

>TcOr293

MISFEENIDHDIYKDDVLWIMRKISIDYFHYKVVKILLTLLSIGITILTVIQTFLFLERFEGRYFVKYAPAYIATFL
MVVAMQYISFSIRLAALIKRITFWTINSARVETERKIKKHAMYTNIFFLGTVIMGVISALFHIMPLDDDNELFFP
LILFEEFVPNWKNFFSWMYRLNFLAVPFTLPIPIYITTYHLIKSYYQILLYLDFLKNINTGFDTTSSNNIESAEYQ
QVTRDRLVFCIKRHSYFYTQMREVNRKMSKFIAIFALISVLLGGSVLTFLFSFQGTFENRYPRIVTLILTAGCIFA
HVIYAGQLIEEAATQVCENLKVLDWYHWNCHNRKLYLIFLQNTQKPYKTQFSQNVSINYELGLSIIKTVYSLI
SVLRNLQDINZ

>TcOr294

MIVYEEKIDQDVNGNDILWVMRKVCIDCFQYKIVKILLLLLAIFIAILTLVQGFRFLERFDGPYFIKYAPAFIRTF
VILVAIAFISFGMETAIYVKDTTFWLIDSAGLESEKRIKKHAMYTNIFFVSYIVVGVISAIFHIIPLDDDNDVLYPL
ALFEEYVPDWKNLFSSIYRFTFLTVPFTLGIPLYTAIYIITTVYYQILLFVVYVKNINTDLDVENVKYQETIEKRLIF
CIQRHSSFLKRMKESNRKMSATVLVFSIVGILLGASVLMFLFSFHMSFKIWYYRIITMVLPTGAIFIHIIFIGQSLE
NAISQLEANLKMVEWYHWNIPNRKLYLIFLINTQEQKGVKFSQNVSVNYKLGVSIAKAVYSLISLMSNLRSID
Z

>TcOr295

MKFAEPQIQEEDYLKLIKICFIDVFETNLAKLALIVAFVIATGLTILQSYYSINNFTANQFLKYAPVYFGRFYVLV
CLVFIFYYTKVIFKIFDTVPKWKYETVSKKYNQHVRRDALYMNYFCLFSVLAGLCTAVLYVIPSEEDHEIFFIVS
WFEDNVLEWADILSLCLRLSFLFVSYLMQAPCFQMCYLLRHIQYQMGILKFYMRNIHHGFENLDKLIFDDSF
HKEIKRRLEFCIKRHVTIVLIGTTMVREGRIFILLFAISGAMISVSLMMFLFLVERSLPFTYLRLSALIFTATLTGIQ
YIAQGQSIQNTSEQWFRTLVRIKWYYWNRENQKSYFIILNAYKPFSIKFSQKLAVNYKLGITIAKAVYSIMSFM
RYITDHVGNZ

>TcOr296

MNFDFSEPEKQEEDYLELLKLCFIDLFRTYFAKFFLTIAFINITALTILQTYYSLTSFNTVQFLKYAPVYFGRFHA
FICLCFILHYIHVTDKLFENVPRWSYTTIGKKYDNEVKRNALYTNILVACIAIAGFGSGVLYAMPTEKDNEIFFI
MTWFEDNLLEWADILSLCHRMSFIFVSVIMQAPCIQICYTIKHSQYQLGILKSILENIHQGFDNLDELVYDDNF
HKEIKKRLVFCIKRHINLILVGRKMVRDTGIFVPFLSISGGMLGISLMMFFYVIEDTSTFVIVRLCGLIFVATVTG
MLYIIQGQNIQDISEECFQTVTQMDWYYWNKENKKIYQIMFLNSFEPFTIRFSENLAVNYELGIELAKAVYSM
MSFMNYVSDYMKTZ

>TcOr297

MKFEEPEEENDDCLEIIRICFIDFFQTHFAKLVLVIAFFNVTGLTIMQTYYSINGFTAIQFVKYAPVYFGRFYVLV
CLSFILYFITTMYNSFQKYPLWKVDTVGTAYKKIVERDALYTKIYIVVIIVIGFCTGILYVIPTDEDHEMFFIMTW
FEDNLLEWADILSLCHRCSFLFVSLLMQAPCFQICYLLKHAEYQLGIIQKLLENIHYGFEDLDEMTCGDTKVQ
GEIRTRLLFCVKRHVSFIVIIKKHIREAGVFVFLFSVTGGMVAVSMLLFFFLNEENLTFNHFRFWALIVVAFLTG
VQYVLQGQSLEDITVEFYETLTRIEWYYWNKENKIIYQILLINASDPVTVKFSENVSVNYKLGIDVGKAVYSM
MSFMTYIRDSVLTRKGZ

>TcOr298PSE

ZLKQVPKLNFDFRGKKQKEDYLXLVYFGRFHALVCLLFILHYINVMDKTFQNIPRWSYTIIGKKYDKEVERNA
LYTYILVACIIIAGFGSSVLYAMPTEKDNEIFFIMTWFKDNLLEWADILSLCHRIGFLFVSVIMQVPCIQICYMIK
HTQYLVGILKSILENVHQGFDNLDELVYDDKFHKEIKRRLVFSTKRHINLVLLGRKIVRDTGLFVFFFSISGAM
LGISLMMLFYVVEDTPTFVNVRLCGLIFAATITGMLYVIQGQNIEDISVELFETVTQMDWHYWNEENKRIYQI
MFLNTFZPFSIRFSENMAVNYKLGIELAKAVYSMMSFMTYVSLKVNZ

>TcOr299

MNFDFRGEPKKQEEDYLELLKLCFIDSFRTYFAKFFLTIAFVNVIALTILQTYYSLTSFSTVQFLKYAPIYFGRFH
VLVCLLFILHYINVTDKNFQNIPRWSYTTIGRTYDNEVKKNALYTYILVACIVMAGFGSSVLYAIPTEKDNEIFF
IMTWFEDNLLEWADILSLCHRMSFLFVSVIMQAPCLQICYMIKHTQYQVGILKSILENIHQGFDNLDELVYDD
NFHKEIKKRLVFCIKRHINITLVGRKMVRDTGIFVLFLSISGAMLGVSLMMFFYVIEDTPTFVNVRLCALIFAAT
ITGMLYVIQGQNTEDISEECFQTLTEMGWYYWNEENKRIYQLLFINSCEPFSIRFSENMAVNYKLGIELAKAVY
SMMSFMTYVSNYIKNZ

>TcOr300

MIGLTNGDYSPRPSMEGDCLKILKFFAVDIFNPKIVRFFLWIMLLYHVVFTLVTAYFMLYVLSNSEIIGYTPAFL
GNFYPMLCVWSVLFISRLIYVKEDMPLWAIDTAGAKVQASIKRKIFLYTAFGIFNLVLSLSAGSFYLKNVSEDV
NVFLALRIFRDYFPNYYQVLDLIYRLIYFCFSYLMVAPSYLLIYYILHVRIQAIIFAAYVAHIDGHSDYGTDIDLF
DNEEFQSEVERRFKFCIKRQIEFLLMESKKLSQISNLIAAFSLAGCLFGISIIFHLFTGQLIQEYYFRIGLTSLAAIA
TFSAFIYTGQSTEVQIELVDNAIDNLCWYNFNRSNKLLYLIAKADLARVRKIKFSGQWAVNYDLGFAIVKGIY
SIISVVVSMWZ

>TcOr301

MSFENEHLGIKLTHFLCVDLIKKRPVKFLKNLIFFQYFVVQIIQTYYFLKIFEIRFFVKYAPVYFGTYFLLFVIIVS
WFSENIDNYTTAQFDKWELENMTQTFDEKLYKKIKTPSMIVSIVIITNFALALVSGYFHILPDDDDKEIFFIFIFV
EEHFPKWQSVISWAIRSTYIFTAYFMIYPINTLSYFMWRLKFHMYFYLENIKKINEGRPQNEKNITTCIPFQKEV
RKRLIRCIKRHTEIAHLYTLTNGIFGTMILVCAVLGGLLMVSTAFFVLAFEGSFFKKIWRVGTLILCAAITSAGS
THSGESLETSGNNIFLYLKEQDWYIWDRENQKIYLIFLTNVKPLRIEFSADVGINYKLAVSTLRTVYSIVSVLSQ
LIKZ

>TcOr302

MDFSEPAPFHADALWIVRLLAVDILNKPFVRFLMFTVLLFHVTVLLIQIYFICFVQTFGEFVKYSAIFCAMFYV
NLSMITLLYEKNMVKHILEKCKLWALNSIDDKIYQDIRREALFGTVFVITNLILVFVTTITFIIPAPLDRDVFFVV
YFFESYLDQTWGKILTLVYRATFLLLGFIVVTCAHQLFYTIQQFKFQIYLLKEHVENITNIEFFDEDDSLLLKNPS
YQREVKRRVRFCIKRHIQILNGADVGMKLVKKWIPLYSIAGILFFVSIVFSCISFDGSLEDVYLRVGSLGVVSVTT
FCSIIFVGESIATQSDVLAQVNTFIRWCSLNRENLQLFAIMGIMSKEPYYVKFSQNLAINYALGIKVVKLVYSFL
CFLIQCKGVLYZ

>TcOr303

MDFPAKRPFKDDPLWIVRLLTVDTVTKKISKFLMTLVLIILAAAQVTQIYYHCFVLPFNDFIKYGPVFFSMFYII
LSICALLFKGEMVITLLAQSNRWAVDSINNQILPEIKREAFFCNTFVIINLILTLIPAILFVIPVEHDEDVFFAFFFF
KREFYHFTNYFTNYFTILSVIYRTTFPILAYVMVTCAHELIYGVEQMKFQIYLLFKHVENITNFCDLDDSVLLRN
SLYQKEVKRRFIFCIKRHIELLTVAKNGLKVVEQLIPVFAINGIMFLVSTIFFCFTFEGTLEGRYPRICSLAVVSFT
TFSSLIYIGQSVETKSDVLTLANTYVKWCSLNKENLQYAAIMSIIAKKPVSVRFSDNLVINYELGFKVLKMVYT
FLCFLVQCKGILYZ

>TcOr304

MRCYNHDVLWLLRFWCCDIFQYKLLKFVIFVMLVANTVLMVLQGYHFLINFNSLYFISYSPYWFGSFFIILSLT
TCLTISNIGPEGIKSATLWQIKSTDPKLINRIKFQVKLITAYIIVNTVIALIAGLAHTFPSKNAEEICYVYKIIEIYVP
KWKTELCWMYKASYIVMALALPATCNQVVYGATHIRFQFYLVLDWIRNNIIESGCDDLKLPNDKDFQHKIT
KNIIVIVKRYTEFHRTFQAVNQRIAIYILLYAVIGGLLGISILMFYFKFSDTLVISDYLHAGTLTVAAITTFMATVT
SGQKLEDIFEELLYTWCSVPWYLFNKWNKQIYLMIMVNIKPINFRFTENSSVNYNLGAAIAKTIYSMLSLLTQ
MSDKDVSTLZ

>TcOr305

MFREQSLMNIPNDDCLWPLRFIFQNLYKIKVTNFIIKKMLTNLTILLIIQTYLFLRENSGSYFLKFLPAFAGSFFF
LASYYSTPIIGKCVFDAIENAQLWQLNTSSPKLTKQVRREAFFTNLVGIVASILAILSATAIGLPSGDKKDFMFF
LAIYESFTPIWKRLVLWNHTFDIAFVALVAIAPCYNVIYLLTHVRLQCLMLLHHVRNLNCDYKFSNLFDLFH
DDKYQRTIEERLKFCIIRHSILLSILNKVRNELQHFIVIFATTGAVLFISIAVFCYSFEGNLRNEYKSLLLLVLIGAL
TCGHVVVSGQLIENVTFDHYEILRTVEWDCWNTRNKNLFVIYLQNSREIFKIRFTENLSVNFLLGISIVRALFT
AFSLMRQLQLSRKEZ

>TcOr306FIX

MQFVEENIIEDDPLRILRFCAIDIFQNVFVDFVLKVIFVCICGTIMVQGYYTLHYFDGATFLKFAPFYFIQFYIIVC
IIFVLYFVNIIYKLQETIPQWKPNVDPTSYHHNIRKISFRITIFQLSLILVCCCGTIVNLISTERDGELYFMIGWVEE
NILIMQQMFIYCLRITFVVCALILPFPCAQICYILQHHRFQIYFLLHHLNHINSNYEDFNDDYLYCNKNYQITIR
QRLVFCIKRHTDLLSLGQRMTPNSGIFIFLFAITGGFVMISILLFVTSVQDMPLIVYIRLYYVTFSSVFILYAYYKE
GQAIENAYEEIIAALRNTNWYCWNNANQKYYFNIYTMASKASKIKITENVSFNYQLGASVVKGIYSGFCLLY
HMNMSNIZ

>TcOr307

MIFKEEKFLQLSNNDCLWPLGKLLHNLMKNKVSMFLFRENLIILLFLLTVQIYLFLKESSMKYFIRFSASYSGSF
FFLVAPYSTPTFSQCVFDTFQKLKLWQYDVAGPELDKRIRMEVLSVNFSFIVNVILAQICAIAFAIPTEDDEEFL
YFFEIYDIFIPRWKNVMIWLCKLNGFFVGVASLIPYYTFVYVSTHSRFQYYLLSHFVKNLNFGFNQDDRLDQH
YQNEIEQRLKFCIKRQTYSSEACRSMNRPLKNYVILFGLSGVILLLSIALMYYSYEGSFKNQFFRIVSFILGTGLT
AIHILRAGQLHEDVTSDLLTILKTLNWENWNTKNRKLFVIMLQNTQESIKIQCTENLAINYELGVSVAKRVYS
IVSVMRSFKGHZ

>TcOr308PSE

MVHLNYYFSCWRAIVTMTRKVFENRGDFSMSVVYFVATGFFQIKCNRFLAYWALTANALSFLIMLYQFILDA
KLVYIIQYGPVISGLTYQVLVSLLGVIFMTDTEEFQQEFDFWDELDASHETQVQIKQHINSVTVYVILNTVLAF
VTGITLILPNEDEARFHYFLKLLGELEAAVPPKIIQIFYYLYKIDFVLMFPVMTINSVRMLYFSRKFKFQVKLLVE
RIGAMTKNYNVDDLSLFYSARYQNDMKQKLKNFVHRHSYIAQYVAKINESIGPFVVLFAISVTLSGISVLLIAA
TGTFYYNKYQITPSIMLYSSTLFCAIDATETVEREXSVGIYNALLAQPWYTWNNENKKNFIIFLMNCEKPIQIT
KFTNTFYFNYEWGFSALKKIYSMGSVFFNLRHYIDKZ

>TcOr309

MPFEWTIRKNKIKPILQNDVLLNLMLVPNTIISNKFLVILNYFYFGFIILQSVFVAVIIITKDEWKLLNGQYAGY
TSGCAIVWSSYITMYTYVDKFLNLYKEIFPHLWSLDVVGQDHFNKFSKMAKVLKLGKNILLVVGFLSATVGL
PWYRDEYEIIITVRVYKDYVDKWTTLLYFVLFSSLYHIALTVIFCVLCLVYMVLHLHNQCVMLNKRLEALDDE
QLFLDNDNYQDFVTKELKFCIQQHQFLLKFAKRLNDILYYPTFYYVLSGVVTGVSLLLFPKNDIKNLLRCVLII
VLGGGFAISFCFLGQILENASEELLFSAYSARWYLWNIKNRKLLSVFLLKTQDNIVLSSSGIITINFRLLISLYQSI
YSCLTFLLNIKZ

>TcOr310

MKQKAVGAKKQLKTDNRDVLVTVKLLANDVFHAKAAKIVLKLLVLIHGSISLIQIYTILFKPNIEEFTLKAPIF
FGLIYPISGALTLLLKPELIDNFPNYTKISLTDNIDPKTYQSIKKSAKIAFYYTVATISLAIISGINYIQFLKHEDEIFF
AYKFFNDYFPKYSLILKIIYKATLPILGYIAVAIPIQTVYGIEHMKYQIMLVKSWVKKIDSDADNKKDQKMVRS
QLVLCIQRHSSLITFIHKIKKEIDSLFLMSLLNAVLCFMSIFLFIFSATFYREYYLRIGLTCITSGFIGLIVVFGQQFE
NEVENLATVTCNLNWYFFDLKNKSIYLIFVGQIIRPLRMQYLDFGLNYRLPLSFLKLVYTVLSLMLKVRPTFHE
QNZ

>TcOr311

MHHKNIQPMTDDYLKFIKFVSSDIFQLLPVKIFLAVVFLTHAVLDLLTIYFVLFVIEPHDFITYISVFLGEFYAPL
FAIVMLLFRGKITDSLKHKLAMWTITSTDEKTQSDIKRQIVFFNGFVVLNSVIISIASWFYAARLSDDVNAFFAL
RLIHEYFPKSIFEVIYRVTNFVLGQMMCVHVHQTLYYTQHINIQVQMFKKIIRDLENESKIEQQLKFCIERHAE
FIKIITLTTKELRGAFVGFAFGGLLLGVAVAFYIFSGLLTPEYYLRVGAIGLASVVNFAVTIWFGQSTESHLDEL
MLAVGEVQWYNFSQRNKKVYLILLMNVMKGRKWRVSEEYSVNYRLGLAIVRGVYSIISVTSSYKKSZ

>TcOr313

MEQLPKNDPLLVLRVLPELLLLHKIVRHFVVFIVCYLTATTIFCLYVLATVRGLWDLFWSQYSLLTFGSVIGFS
CYFVAFWKGFKFLELRRRVFADYWALTSLGEESFQKIKKLSKSANIFTVGTILASIATSSTCMPWVGDEYDIMF
PVRVYTDYFGERAVPLLVPFYLAMYCTGFVMIATGFIFVHFALHLKFQFFLLNRRLDGLQTEPLVNDFSYQNR
VKEELTCCIEYHQKLLKVAKEMNEIVYYPIFIVVSSGIICSVCLIFYMKTFENSIVRGTAMAISGGLITFGFGFTG
QLMENESGRLFDTSVMLPWHLWCLSNRKLYHIFLTKSQYHVSFSSSGIINLNHTLFISLYRKVTSIFSFLMNVSN
KNSTZ

>TcOr314

MEQLPKNDPLLVLRALPEILMQHKIIKYVVLFIICYMTVTMILCSYVLATVRGLWDLFWSQYSLLAFGSSIGFS
CYFVAFWKGSEFIKLRRRVFANYWPLTSLGEESFQKIKKLSIFANVFMVATILASLATSTAGLPWVGDEYDIMF

PVRVYTDYFGERAVPLLVPFYLAMYCTGFVMISTGFIFVHFALHLKFQFFLLNKRLDGLRTEPLVNDFLYQNH
VKEELTCCIEYHQKLLKVAKEMNDIVYYPIFIVVSCGIMFSVCLVFYMKNFKNSFVRGTTMAMTGTLTTFGFG
FTGQLMENESGRLFDTSVMLPWHLWCLSNRKLYHIFLTKCQYHVSFSSSGIINLNHTLFISLYTKITSILSFLLN
VSKKNHTKZ

>TcOr315

MTLVRKLQAAATNAFEIRIKDDILAELFNWPFLVLDSKWSTKFAVFLTVYCVFETLACALVYSTLDVNMMGT
YAIVIARFATTFCSFFSFFTKRKQYFEIINENFPHFWPLQSLGKSTFNRIKMRASSVKFYSFLNVVVMLIGAVILIS
FTQDESEVYLSVKIYKDYVNKWTTGFVMFFYVSFIYIGLVVAAISFVLTYTAFHLIFQCFLLNQKLKQINDSIVE
NEQKQAKFDEKYQSFIYKELISCVKLHQRLILFGKRINHLVYAPLLVYIFGGIVVGVALIYYLKSSVQHIFTSLIL
LLIALINSTTFVINGQMLENEAENIYISLTNLPWYSLNVQNRRVVYVMLMQSQKIIHMSASGLVSLNYQLTIVF
FRCIYTGMTFLVNVGLZ

>TcOr316

MTLMRKLQTAIRNLFEIQIKDDILAELLDWPTLVLFSKWPKNFAIFSTIYCVFDTLVCTLVYSTLDVEMLGKYA
IFIAKSTIALCSFFSFFAKRKQYHKIINENFPHFWQLQSMGESTFDQMKKIATTVKFYSCLSVVAMLIGAVILILF
TEDESEIYLSVKIYKDYVNKWTTGYIMFFYASFLYIGIVTAAVVFGLTYIVFHLIFQCFLLNQKLKLINSYIVKNG
QKLVKLEERNQNFIYKELISCVKLHQRLIYFSNQINDLLYAPIFMYTFSGIVVGVALIYFLKTSIQYILTSLVLSIVS
LIITTTFVINGQLLEDETENIIISLTNLPWYSLNVQNRRVVYVMLMQSQKIIHMSASGIVSLNYQLTIVLFRCIYT
AMTFLVNMGLZ

>TcOr317PAR

MQRLITNLIIIRSKILKIFKDPDDRDVLRVVKLLSSDIFHTKYAKILLIILILVHGSVTFCQIYFIVMEPNVQDFILK
APIFVGILFPLTSVITLLLRLNLIDDIPYSSMENVDAKIHTWAQLTRICTVSTFILAFLCGLSYDERLATVTYNLE
WYNFDLKIAKSTVSFIKCHETVSNEHMGLAINHQLGLSVMRTLYSAVSFTLKIMDSKLKDKAZ

>TcOr318

MTQTDVLHLIKFLTNDIFRSKIAKIFLLSSTIFSASVTLIHSYFMLFRPNLREFSLKAPMFFGFCYPFLAGVVLLFE
NKLIENIPKQVKSGPIDQKLRKKIKTIKLYVIFVMISAVLAGLSYVQNVTNEAEIAFALQFFLDFVPNYYTFLAV
CYKVSLFLMAVIAIIHPMQGIYAIEHMKIQVILLQKHVKMIEKKAKSGKDVETTLKFCIKRHINFLNFAKKLSA
RLSFLIAILVACGCVILMAISVFMLSGSFSPDYTFRITATSLETLATFVGVLKSGQELEDEIDKLGQILCTLDWYSF
NCKNRKLYLIFLMNAMKPFKLKSLESYAINYQIGLSAVFTVISFTSQMHSKLYKNGHZ

>TcOr319

MLINCYIFTILISFSEMLNPQHDRDALKIVKFLASDIFHNKSAKLFLLIVALIHGSVTLIQIYCTLANPDIKDFILK
APIFFGMFFPLTGVITLLLRPELIDDVPQYMKNWSMENVDAKIHTWVQITRIYGVGTFILAFICGLSYSQFLINE
NQFFFFYQFLDDFVPKYEFRIFCKIIYKATFPVMFYAAVLHAIQTAYGTQHMKFQLIMLREKIEKIDCNEKHQ
KKIRQKLIYCIRRHDSIITFGRNKMEQMSELIGIFLTIATLCFISILIFMFAGPFYKEYYVRLCLTCATAVAAFGSL
LVFGQKLEDEIEQLATVTYNLKWYNFDVENRKIYLLFLLNVMKTFQVKYLDFAINYRLGLSIVRTVYSVLSVM
LKMDSKLYDKQNZ

>TcOr320

MLIYNRFSKNYFSMKKLNDPLRFIYKMTLDAIDYKIMQFFLTLTFVLHSFVSVAFILELTLTFDADLLVTYGPIIF
TFGSGIGGFLVVYKIRRTLKLLLQKCKLPAPDSKSQMLERIKRESKIILNAVYFDNILMIVTVLFHWPIIGENNNI
YYATVLFEKVVPSFSSKLCVLYYLSLFFIVYLVAMNPYLLLYTSTYIKFQLCYINELLTNIDAKWSKFGDYQLMR
NNVYQEMISEELKKCIDRHRLFQSLVKELNNLIYIPLLILMSLAILAIVSILFNIIVKRQETFYCRVCSTLLFALLT
LSFAIISGQLIENEWKKLIERAFCCRWISWNFENRRTLLIFFMNAQRPSRIGNAFIRCEYSFLLTAGRFVCSLCAF
FFQLARIREEEMLQIZ

>TcOr321

MGKMNDPLQFICKMTLGAIDYKFVQLCLLLTFLLHSFVSLAFSWELILTFDANLLVAYGPIIFTFGSGIGGFLVI
YKIRGTLKLVLKKCSLPALDSNPIIFERIQSDSKIILKAIYFNNILMVVTVYLHWPVSGQNNNIYYAIVLFERIEA
KFSSTFCFLYYMTFFFVVYLVAINPYLLLYMSTYARYHLCYINELLTNVDAAWSKYGDYQLIHNNVFQNIVSK
QMKKCIERHCLFQSLVKEINDVIYTPLLILMTLAILGIVSILFSIIVKRQETSYCSLSSTLLFAFLTVSFVIISGQLMQ
NECEKVIERAMCCRWTSWNYENRQTLLIFLLNAQRPSKIENVFIRCEHPFILTAGRFVCSLCGFFFQLARIREQI
VQNZ

>TcOr322

MTFHWITTPLEPILKDDPLFVLMALPNKLIGSKLQALVNYFFFVYMVILPVSCFLVIVATNQWQIFYSPYSGYA
SGVFIVWSCYVSFFIFGSKYRRVYRDVFPHLWSLDVAGEEHHNRLKKIGKQLRTFKLVLITLAFIGATSGLPWF
GDDYDFYIPIKLIVDYCDQWKLFFSIFFYLSFYHIGVTVLSCFFSLMFLVLHLQNQFYLLKTRLQTFATDSGTSD
VFLSMKVKDEEYNRSVTQEIVFCIRHHQSVLMYCDRLNDLLYLPIFYFTLSFIVTGVSVILFPKYDLQALIRSLFV
IVLGMCMTLLFCSLGQLIENESENVLYSLIEAPWYLWNTTNRRLYYLFLLKAQDTVNLSSSGLITINFQLILTLY
RGIYSALTFFLNFSZ

>TcOr323

MNFNFFDKSDDFSMPVVYFVAEGIFHIKFIRFLAYLALISNALGLLLMLYQFVLDAESLYIIKYGPVLSGAIFAL
VSLHAILFMRDLETFKQEFDCWSEHDASQETQNRIKQHINSVTIFVIFNSVLAFVAGVSLVLPSKDEVHYHYFI
KILLELEVVPRGVTRTVYYLYKIDYVVMYPILTINSVRMLYFSRKFKFQVKLLVDRIVAMTKDNVDDLSLFYST
PYQNDMARKLKTFVRRHSYIAQYVAKINKSIGPFVVTFSLSATLMGISVLLIAAAGTFYYNKYQIILCGAMYLC
TLCSAIDATETVEMESIEIYNALLAQPWYIWNNENKKVLIIFLMNCEKPIQITKFSDVFYYNYDWGISVLRKAY
SLGSVFFNLRQYIGKZ

>TcOr324

MNIKFLDKSGGFSMAVVYFVAEGVFHIKFIRLYTYSALISNSLGFLFMLYQFIVGAESLYIIKYGPVLTGCTYVL
VSLWGILFLRKTEEFKQEFHFWSEHNASKEIQNRIKQHINSVTVYVILNIVLAFTAGTSLILPNKDEIHYHYFIK
KLSELDTIPRGINETCYYFYKINFVLMFPIMTVNSNRLLYFSRKFNFQVKLLVERIETMAKDYNVNDPNLFYNV
RYQNDVKQKLKIFIRRQAYIAQYVAKMNKFLAPFIIMFAISATLLGISVLLLLVTATIYYNKYQLILCGAIYISTL
FCAIEATETVEMESVEIYNALLAQPWYSWNNANRKTFIIFLKNCEKPIQITKFSDIFYFNYDWGISVFKKVYSLG
SVFFNLRQYIDKZ

>TcOr325

MCNLHDRYSQFGCFIVITFRQDSKVFFLTNMYSKLFGERGEFSMSVVYFVATGLFQIKLIRLMVYLLLIANTLG
FLAILYQFILDAELVYIIQYGPIISGSTYALGSLYGIIFLRDAEEFQHGFQFWNEHEGSKETQNRIKQHINSLTVSV
ILNTALAFVTGTSLILPNKDEIHYHYFIKILMDLETVPRRLSQALYYLYKLNFVMMFPIMTINSYRVLYFSRKFK
FQVMLLLEHIETLTKNYNVDDINLFYNTRYQDYIKQKLIFFIRRHSYIAQYVAKINNSIGPFVVLYAISATLLGV
SVLLIVATGTIYYNTYQIILCGAIYLSTIFCAVDGTETVEMESVEIYNKLLGQPWYTWNNENKRIFVIFLMNCK
KPLQITKFSDTFYVNYDWGIAVLKKVYSLGSVFFNLRRYIIDKZ

>TcOr326PSE

MFPKLLFEIIVFSHEKENKNDVLWLLRFWCCDIFQYKLLKFVIFVMLVANTVLMVLQGYHFLINFNSLYFISYS
PYWFGSFFIILSLTTCLTISNIGPEGIESATLWQIKSTDPKLINRIKFQVKLITAYIIVNTVIALIAGLAHTFPSKNAE
EICYVYKIIEIYVPKWKTELCWMYKASYIVMALALPATCNQVVYGATHIRFQFYLVLDWIRNNIIETGCDDLK
LPNDKDFQHKITKNIIVIVKRYTEFHRTFQAVNQRIAIYILLYAVIGGLLGISILMFYFKXFSDTLVISDYLHAGT
LTVAAITTFMATVTSGQKLEDIFEELLYTWCSVPWYLFNKWNKQIYLMIMVNIKPINFRFTENSSVNYNLGAA
IAKTIYSMLSLLTQMSDKDVSTLZ

>TcOr327

MYGFKLSYRAFHDVLRLLRFWCCDIFQYKLMKFVIFVMLVANTVLMVLQGYHFLINFNSLYFISYSPYWFGSF
FIILSLTSCLTLPTIGPEALQSMDLWKIQPMKRIKFQTELIRAYTTINTVISLIAGLAHTFPSKNAEKICYVYRIIGI
YVPKWKTELCWMYKASYIVMALALPATCNQLVYGASHIRFQIYMLLDSIKSNLVKEFGDDLKLSNDKDFQE
EITKKIIFSDNRFSMIHKSIQTVNRRTSVYILLYAIMGGVLGISILMFYFKFSGTLLISDYLHAGTLAIAAVSTFMA
TITSGQTLENLTEELLVTWSSIPWYLLNKRNKQIYLMVMVHFVKSLSFQFTENMAINYNLGAAIGKMIYSMLS
LLTQMSHKDTDTLYRQZ

>TcOr328

MSYNIKLTKDDRLKLLKIMASDVFQSKTVKIILIVVFLVHAIANSLTIYFALHVSDTKQFISYASVFFSEFYPMLA
ILTIIFKGEVVQHLTDDINIWTIDGASKKLQSEIKLKIKILTAFVIINSFSVVIGGFCFVQQLSDDVNLFFAIRLIRD
YFPNHSTILEFFYRMTYPICAYLMAVHAYQCLYYTQHINFQLQMFTEIITELTDLKTISLPENRLFYNKKYQTVI
EQRLKFCIKRSQEFIKVCVTKNKEIGSLIPGFAICGLFLGIGITFFLSTGKFTTEYYLRMGVTSICGLTTFSALIWSA
QTTETMINDLVMVINKVSWYNFNQSNKKLYLTFLLNTMKERKIKFTEKYSVNYQLGLAIVRGIYSVISVVASK
RHHZ

>TcOr329

MNCENQFAKDDYLKTLKIMASEVFQSKAVKVILIFVFLVHAIANLLTIYFVLYVSDTKLFVNYASVFFSEFYPM
LAILTVIFKGQIVQHLTDEFKIWAIDSASKKLQSEIKLKIKIITAFVITNSLIAVWGGFLYVQPLSEDENLYFALSFI
HQYFPNQSSTLEFFYRMTYPILGYLMTVHAYQCLYYTQHINFQLRMFTEVVAEFAPVKRFLLFEHHLFYNKK
YQTEIEQRLKFCIKRSQEFVQICVIKNSEIGSFIPEFAICGLLFGIGVTFFLSTGKFTSEYYLRMGVTSFGGVMTFSA
LIWSGQTTETMTSELVKALNEVRWYNFNQSNKKLYLTLVMNIMKERKIKFTENYSMNYRLGLAIVRNIYSVIS
VVVSKRRHZ

>TcOr330

MNYKKQFAKDDRLKTLKLMASDVFQSKTVKIILTVVFLVHFIANSLTIYFVLYVFETKLFINYASVFFSEFYPML
AILTIIFKGDVVQNLTDEITFWTIDSASKNLQHEIKLKIKFLTAFVIINSFTVVMGSFSYVQQLSDDVNLFLAIRLI
RDYFPNYSTILEFFYRMTYPICGYLMAVHAYQCLYYTQHINFQLQMFTEVITELNNSKTSSLLENHLFYNRTY
QTNTEQRLKFCIKRSQEFIKICVTKNKEIGSLIPGFAICGLFLGIGITFFLSTGTFTTEYYLRMGVTSICGATTFSAL
IWSAQTTETMTSDLVMVINEVNWYNFNQTNKKLYLTFLMNTMKERKIKFTENYSVNYQLGLAIVRGIYSVIS
VVASKRQHZ

>TcOr331

MNFFQKKLAKGDFFKTLKFIASDVFQSKAVKMVLILLFLIHAIIYLLTIYFLLYVLEPKQFVNYATVFFAEFYPM
LAILTVILKGKIIENLTDEIKIWAIENASKNLQSEINLKIKIITTFVIVNTLIAVSGGFLYMHPLPEDVNLFFALRLI
RDYFPNHYTSLEFFYRMSFPIFAYLMTTHANQFLYYTQHINFQIKMFREVCLEVKAWKTVSPFENHLFYNKK
YQTEIEQRLKFCIKRSQEFVKISVYKNKEIASFIPGFAICGLLLGVGLVFFLSNGKITWEYYLRMGFTSLGGVTTF
LALVWTGQTTENITSDIERAINEIRWYNFNQSNKKMYLILVMNTMRERKIKFTEKYSVNYRLGLAIVRGIYSVI
SVVLSKYQHZ

>TcOr332

MEFGNYKLMTDDYLKTIKFMSSDIFQPIPVKILLGFIFALHSAVNLVTAYYMLTTFDAKLFINYSSVFFGDFYPL
LATFALISKNNTVRNLKDELEIWTIDSAGEKLRSEIKLKIKFLNIFVVCNSLLVLVTGLTFIQPLPKDSDIFFAYRL
IHEHFPKHGQALEFLYRTTYVLISYIVAVQPFQIFYYCQHINFQLQISIETLKKISDWKTLSEDGENLIDNVKYQ
TEIKRRLKFCIQRSQNFICLHTEKIKEVSTFIAGFAVCACLLGIGVIFYLISGNFTPEYYVRMGFTSVVGIIIFAATI
WAGQSTESAIDEMVTSLNEVEWYNFDQSNKKLYLIFLINSMRERTIKFTENYSFNYQLGLAIVRGIYSVISIVLZ

>TcOr333

MEFEVKTFMTRDYLKVVKFLASDIFLAKPMKILLLLIFIVQASVQAMTGYFMATAFNAKFFNNYAPIFFGTFFP
LLAISILLLKNKIFHNLKNELKIWSLDNAGEKIHSGITTEIKVVTYFVIVNSVFVLLANSTLAYPLSQDVNVFFG

CYLIHKYILTYGRTFEFFYKATYLVIGHTNTGHVYQLLYYTQHINYQLQLYIEFIKFLDEGKTISKNEDDLFNNP
TYQTLINQRLTFLIKRGQEIVKFHIKKTNEIRTLIPAFSVCTCTMGIGVVFFIISDNFIREYYFRMGMVSLVTVSTF
AAGIWSGQSMETNLNEITTALNEVKWYNFNKSNRKLYLIFLTTSMRERKIKITENYSLNYQLGLTIVRGIYSVIS
VIINMKZ

>TcOr334

MDHPDIKPMTDDSLKLIRFIASDILQPLPVKIFLGVIFLFFTVGSNLLMIYFVLYVYDIREFMDYAPVLFASFYSG
VAILSAIFKGKIIHTLPDDISLWALDSGGEKIHSEIRFKARMVTIFVICNTLLIIGGIILNLIPLSDDLHVYFALRFIH
EYFPNHKTCLIILLKASIFPVIPHMLVVHAYQILYYTQHSNFQIQLFNKVIAEVDFWETPLRETELFYSKPYQKG
IEKKLKFCIQRLQVLINAYIVKTKEIGTLIALFAICGVLMGIGFSLYLFSGKFTPEYYLRLTFMTLVAVTTFSSIIW
GGQSTETIITEMITALCQVRWYNFSQTNKKLYLILLTNMMKDRKIKFTENYSINYQLGLAIVRGIYSIMSVVVK
MRSZ

>TcOr335

MDHPDIKPMTDDPLKLIKFMASDILQPLPVKIILLVTLLALPVGSNVLMIYFVLYVIDIREFIDYAPVLFGGFYPS
LAILIAVFKGKLIHNLQDEIKLWAIDSAGEKIHSKIKLKVRMVTIFAICNTLLLIVATVHNLIPLPRDLHIYFVLR
LIYDYFPNHKTYLLILMKLMSPVTTYMLLVHAYQILYYTQHINIQIQLYNKFVADVDFWETPLCEPELFYNEL
YQKRVEKRLKFCIQRSQHFVYVHVAKIKEIGILIALFAVCGVLMGIGISFYLFSGNLTPEYYIRIFIIALVGATTFS
SIIWGGQSTETIVTEMIATISQVRWYNFSQTNKKLYLILLTNMMKERKIKFTENYSINYQLGLAIVRGIYSVMSV
LVKMYSINTZ

>TcOr339

MMLFRNTEIRRFQTKAKDPLHFLYASTFGLFRVKLVRLVSIFTLVFHSVISCAFFCEMLYSFDPNLLIEYGPMIFI
FGSGVMAIAVFFYIEQNGRLLLRQSVGRLWNFDSSSNLYKKMKLESKYILQVVHFNNFLGVCMVFFHLPAGG
QSDKVFYGINLFRRILPQHLNFLKQIYYSTFPILTYMVTVNPYLLLYASSHMKFQVCYVNELLVKMTKDYKDI
DYNLLRNEKYQKTVTLGLKNCIHRHAILKFMDKKLNKLIHWPLLILMALSVVAIISLLFIAIVTRQDVHVPSFG
ATMIFGIVTNSLAVYSGQEVINESMKSLSCAGTSRWTSWNFFNRKMLVIFMTNAQKPFILQSPFFVCEFAFVIK
ALKFVSSVCGLFFEVARRRDLGELZ

>TcOr340

MPYVQKGRLNFKPTNPYFNFRRPFLGPEKTVHRHRVSHRNEKTKFRHLLIQFDTSIVIFQALAQTVYEKTVLE
LLCEFYSLYWPLDNVNPGKFLKQFRFLYVISFVTGGMFITSVLLVSPVFKNEKDIFLIREMFHNWGQILEVVFW
AGLFFQTVWAILIACVLAYAIFGIKFQLSLLLYQIKGMKGLRHQSMVKEKLHSVIRRHVCLTGFVRKVVKTYW
GLLQVEVCMFLVVNISMLFFFINSFSRSDWQHNLRPLCITVTSFMLTTCLIVLCLQIPDMTGRIFDTTLELPWHL
WNSKNRRTLLIFMTNSVQPIYINILGLGRLNSSSVSEYVKMIYSTTTVLCSLREGKKZ

>AglaOR1/Orco

MMKFKVSGLVADLMPNIRLIQASGHFMFNYHADNSGALHALRLGYSCAHLLFCLFQYGCIFGNLVVEKDD
VNYLAANTITVLFFTHCITKFVYFALRSKLFYRTLGIWNQSNSHPLFVESNNRYHALALKKMRTLLICVTATT
VLSAAAWTGITFVEESVHNIKDPDNENETITEEIPRLLIKSWYPWDAMSGMAYYGSLIFQIYYVLFSLAHANL
MDSLFCSWLIFACEQLQHLKEIMKPLMELSASLDTYVPKSADLFRAPSAKSQDNYIENDYNAKNEELNLKGI
YNTRQELGGNFRSGALQTFGQGGVGPNGLTKKQELMVRSAIKYWVERHKHVVRLVTAIGDAYGVALLLH
MLTSTVMLTLLAYQATKINGVNTYAATTIGYLVYSLAQVFHFCIFGNRLIEESSSVMEAAYSCHWYDGSEEAK
TFVQIVCQQCQKAMQISGAKFFTISLDLFASVLGAVVTYFMVLVQLK

>AglaOR2

MKQNYDFYGYFTSNIMIYKIVGFWRPDEDMKFKKLYNCYTAFCTLAWMAFLLSEIIYITNNRKNVQEITAAL
YVTGTFTIDFIQMIFTYKNMNHLKILMKEMNRTLFQVKCREHYRIAENTKRTYNVLFKSCLYLALLTAVFVMI
VPLVGKERATSIKGWFPYDWTKPLYFALTYIFQNLVFIWNALICSNFATFTSALLMQVGLQCDLLCCTLDSLD
DFYTEDDVLYEISLENKKKLRKDEERFSEAMTKNLVVCIEHHRQIMRVTKNIEQICGTSIFILFIGGTLILCSSLF
QLSVVKVGSVESVMLLLYLICMIVDQLCYSWFGNEVICKSSLILQSAYKTPWVDCNIKFRKILLQFMTQTCQPI
SILTGGLFTMSVQVFVSIMRTAYSYFTLLKNIQ

>AglaOR3

MQQNYDFSSYFRPSIIMLKLFGFWRPDRNMKFKGIYNCYTCLCSSIWVAFLLSQIIYIINNRNDVQEITAALSVT
VTFTVDLIVMMFTYKNMNCLKILIKEMNRPLFQVKCQKHYHIAKNTERMYKLMFKSCLYLATLTDVFVMVV
PLMGKEKMSSVKGWFPYDYTKPLYFILSYIFQKLVFIWNTFISFNIGMIILALLIQVGLQCDLLCCTLDSLDDFY
TEGNVLYEISLEDKLKLTKDREIFSKEMIKNLVICIKHHRQILRVVKDVERISGTGLFILFVGGGLILCSSLFPLSIV
EIGSIEFIMLLFYLICMLVEQFCYCWFGNEIIFKSSLILQSAFNTPWIGCNVKFQKILLVFMMNTKPMSILTGGLF
TMSVPVFVSILRTAYSYFTLLQNIQ

>AglaOR4

MQQNYDFSSYFRPSIIMLKIFGFWRPERNMKFKGIYNCYTALCSLIWMAFLLSQIIYIINNWNDVQEVTAALSV
TVTFTIILIQMMFFYKNTNYLKILIKEMNRPLFQVKCQKHYHIAKNTERMYKLMFKSCLYLSVLTDVLVTVVP
LMGKEKKSSIKGWFPYDYTKPLYFILTYIFQNLVFIWNTFVNLSIFMIILALLIQVGLQCDLLCCTLDSLDDFCT
KDNVLYEIGLEDKLKLRKDRERFSEEMTKNLVICVELHRQVIRVIKDVERINGTGIFILFVGGGLILCSSLFSVSV
VKIGSLEFIMLLFYLICMLIEQFFYCWFGNEIIFKSSLILQSAFNTPWVSCNVKFQKILLVFMLKTSKPISILTGGLF
TMSVPVFVSILRTTYSYFTLLKNIQ

>AglaOR5INT

MQQNYDFSSYFRPSIIILKILGFWRPERNMKFKGIYNCYTALCSLIWVQFLLSQIIYIINNRNDVQEVTAVLSVT
VTFTINLIQMMFFYKNTNYLKILIKEMNRPLFQVKCQKHYHIAKNTERMYKLMFKSCLYLAILTDALVTVVPL
MGKEKKSSIKGWFPYDYTKPLYFILTYIFQKLVFIWNTFICLNTAMTIIGLLTQFGLQCDLLCCTLDSLDDFYTE
GNVLYEISLEDKLKLTKDRERFSIEMTKNLVICVEHHRQIIRVVKDVERISGTGLFILFVGGGLILCSSLFPLSVSS
LILQSAFNTPWIGCNVKFQKIMLLYMMKTSKPMSILTGGLFTMSVPVFVSTLRTAYSYFTLLQNIQ

>AglaOR6

MKKNKTFDYSQLMAHIFKIYKILGFWRPDPDMKHKNLYHCYTAFWLSLSITFMSSQVIYMYNNRKSLKEVLA
ALYITLTFVSILARQLTTYKAMNELKEIIKQLNRPLFQVKCQKHYEIAEETNRNQRLLYNICLFLGVSTDIFAAL
FPLFSKEKVILAKAWFPYDWTKPFNYFMTYIFQNAVLIWHTFVCYSIDIFTFILLVQIGIQCDILCYTLNHLDDF
YFKDGILHEISLRDKLELRKDMEKFSKAMVKNLVVCVQHHREIIKLAKDIQRIKEIGIFVLFASGALVLCTCLF
QLSMVQFGSVESMMLLFFSICMLTEQFLYCWFGSDVIYKGSLILQAAYNTPWTDCNSKFRKILLQLTTQACCP
LNILAGGLFIMSVPVFISVLQTSYSYLTLLHSIQ

>AglaOR7

MKKNKTFDYTQLMAHTIKIYKFLGFWRPDPDMKHKNLYHCYTAFWLSLSLIFISSQVIYMYNSRKSLKEVLA
ALYITLTFVSILARQLTTYQSMNELKEIIKDLNRPLFQVKCQKHYEIADKTTRNQRLLYNICLFLGLSTDIFAAIF
PLLANEKVILAKAWFPYDWTKPFNYFMTYIFQNAVLIWHTLVCYSGDMSTFTLLLHIGIQCDILCYTLNHLD
DFYFKDGMLHEITPRDKLEFRKDMEKFSQAMAKNLVVCVRHHREIIKFAKDIQRIKEVDIFILFASRALILCTC
LFQLSMVQFGSVESMMLLFFSMCMLTEQFVFCWFGSDIIYKGSLILQAAYNTPWTDCNNRFRKILLQFMTQA
CCPLNIPVGGLFVMSLPIFISVLQTAYSFLTLLHSIQ

>AglaOR8

MKETLNFKEIITLNVEVLNGFGYIAPEFQHKIEAVWFGIRSVVFIGFIYLGMLTSEIVNMILVSGDMEKMIEATF
LTLTHLVEIRKVYAVIRYRDRLKKLLNSINRKEFLPKTTTQAKALQNYVQDSKVISKVFLGACVATCSFWGIYP

FVDDGDLRLPLGGWFPFDTRYSPWFELAYVYQVIGSTVNGLVNVSLDTFMSGLIMVVCAQLNILNDSLKNM
REQAETELKGVGIEVGQYMTNTLQEKMNEKLLNCVNHHRCIIEFANELTFLFTTSILGQFIVSVVIICITLFEITL
LPALSIKFFSLILYQFCMLLEIFLLCYYGNEVIRESAELTKFAFCSDWMDCSPEFKRNLVFFMTRSQMALKLYA
GGFFTLSLETFVKILKSSWSYFAVLNSVHTDD

>AglaOR9

MNFNFQLYFQIDFTRLKFFGVWINDGKKKPRLYPQYFCVINIFFIFLFNILQFIHLSYTSDSLRSIASSGYIIATCF
MSNVKSFFMFRNRHRFEKLSSALNENVFQPKNNFQLLLTKKVLSLYSHVKTSLLVFCSVAVFSSMVNPLFHK
NNDQDLVMPAWYPFDISTFLVHNVVYIHQCIAVFYTSYINIYVDIMLAGFATFIALQCDLLCDSLRKIPKEKY
VLNKYVEHHWKILSFAKDTETLFSQIYFGQFINSTLAYCMTLFLLTLVDRTSFEFFYLVFYQTSMFCLLLVPCW
FSSEITTKSKNIPIAAYECFWTDKPNYFKKDLLFFIHMSQEPIKLYAIGFFHISVEIFVKLLQSLIYSFSYL

>AglaOR10

MKRFQLKEYLHYNLVVMKYLGLWPKKDFTSDKSYLIYTIIVNGFFNFTMSVGLTGYILTSSNLLEDVIGAGYII
LGIMATAKTFFIMKYSKMFKLLVDAEIHRSVNVELNEEQTKILCDYVGFWKKVHLIYSYLSVLVFFNYVTLPIF
SKTPYTLPLNCWYPFDYKRPVVYEIVYFHQSVSALIDIFVNLNGATLAAGFLAYLSAECDVLVDLLKNLNNGS
AMNEINLDTEMLKICVQYHKKIVEYSKLVESYFSKYLLIQFTSSCVSTALIMTSMSMNHDVNADFWFLSVLQL
GVWADLYIYCWYGNEVTEKSKKIPYAAFESNWVPASKGYKKDLLFFICRTQKPIKLYAVNFFELSLSTFIGILR
MAYSYYMLLSQLSKDE

>AglaOR11

MKKFQLKEYLHYNLVVMKYLGLWPKKDFTSDKSYLIYTIIVNGFFNFTTSVGLTGYILTSSNLLEDVIGAGYIIL
AIMATAKTFFIMKYSKMFKLLVDAEIHRSVNVELNEEQTKILCDYVGFWKKVHLIYSYLSVLVFFSYVALPIFS
KTPYTLPLNCWYPFDYERPVVYEIVYFHQSVSALIDIFVNLNGATLAAGFLAYLSAECDVLVNLLKNLTNGSA
MNEINLDMEMLKICVEYHKKIVEYSKLVESYFSKYLLIQFTSSCMSTALIMTSMSMNHDDNADIWFLSVSQLG
VWADLYIYCWYGNEVTEKSKKIPYAAFESNWVTASKGYKKNLLIFICRTQTPIKLYAVDFFELSLNIFISILRTA
YSYYMLLRQLSADE

>AglaOR12CTE

MPPRDLVQSSFKYHLLILKIFGLYPYDSFPKLYKPYAFFFYIAFTVITPILALVAIIVSEDHDIAAISQKGFMIVEL
NAMIVKLLPCKINPEGTRRTVFALNKKIFNSQLPEQDHILSEAVRNIKYVLLIFSTTCTCAVMTWASLPLMYN
DRRFPFEVWLPFDPFQNTAVYLFLYLFVFLCTMNGGVDNAVLDTMVASLIYHAACQIRVLKDTLLHLDRRIE
DQISKEGKSLSTEEREQLKNKVIYKKICDCIDHYDAIYEFVQDLERTYTVVVFSQLLSSVIIXXXXXXXXXXAVPL
TIPFFSTSSFTVAILMEQFLYCYSGALLNEE

>AglaOR13

MSSEDLVQRTFKYNIAIMKIFGLYPFDSWPEIYKLYSFIFYLVFTFITPALIVVLLVVRAEESIQIIRQDGFMMVELI
TLTMKMLPCKVNPEGTRRTMYALKQRIFNSQLPEQDWILAETVDNCRFVFLTYCTSCVFTVLCWACVPLVYE
VRRFPITVWLPFDPFENTPIYFSLYIFLVLVVVNAGVDNICVDTLLAFQVYHAASQIKILKDTLAHLGERAEEQI
LKEGKSLSLEDKDNLKNNIIYKKICHCVDHYEAIYKFVEDLETTYSFIVFSQMIATIVVICICCLRFTVDIPFTMPF
FGTATFTAAALIEIFLYCYSGTLLYEESNSVINAIYMSEWYTYDEKSKKALLTLMERAKRPIKVTAGKLLDLSLA
TFTAIIRRSYSLLAVLKNY

>AglaOR14

MFSRDLTESTFKYNIAIMKIFGLYPFDNWPKVSILYGFISYVVLTFITAVLVVVLLIVRIKDSVQILSEDGFIMVELI
VLSVKILPCKLNIKGIKRTMHALKQEIFNSQLPEQDRILAETIDNCIFIFLTFCTCSVITVSLWACVPLAYEARRL
PIAIWLPFDPFEDTAIYISLYIFLIYVVVNGGVENVCIDTLLAFQVYHAASQIKILKDTLAHLGERAEEQILKEDK
SLSLEDKDNLKNNIIYKKICHCVDHYEAIYRFVEDLETTYSFIVFSQMIATIILICVCCLRFTVDIPFTMPFFGTAT

FTAAALIEIFLYCYSGMLLYEESNSIINDIYMSEWYTYDEKSKKALLTLMERAKRPIKVTAGKLLDLSLATFATII
RRSYSLLAVLKNY

>AglaOR15CTE

MPSNDLVQRSFKYHLIIMKIFGLYPFDSWPQYFTPYALFLYVIFTIATPILAVIHLIVGEKPIVDVITENGFMIVEL
IALIAKFLPFKMYPERTKKAFSALNKEIFNNHLPEQEAVLDETVENCRFIFRIFCMSCAFAVLSWASLPLMYED
RRFPIDVWLPFEPFENTAVYLSVYLFVCLSGVHAGFDNATVDSIVALLIYNASSQVIILKDTLMYLSKRTEDEIS
KENRSLSTEEKENLKSNIIYKKICHCVDHYNAIYQFVEDLEDIFSMVVFSQLIASIIIICICCLQLSVAVPFTIPFFG
AVSFLTAALLELFLYCYSGTLLFEESGTIVTAIYMSNWYNYDKKSKKALLTMMERAKRPMMVTAGKLMNFSL
ETFST

>AglaOR16

MVLYEIVNAIEVPKNMLIVSGVWPHDGPTFWYRLRKLMSYGGTMLLLALMFLEVVVSISNILNLSYVIPLSFE
YFSYAVRLMVFRLEKVNVRRLSERLKDPLLNSYGEEHNEFVKSAIARTNRIATVFKVNCLLTTSFLALTPFIAD
AMFPIPFSFFQEGSMWYVVYLVQVNGLAVGIWNNAGLDTLNIGYMGVALAELDILIYNIEHSAEASASGVTK
SKSDVDEYLFTCYKHHNKIMEFIKNIEAVCTKSILVHYVSSIVVICNSGFHLIMTPLFSGQFIILIVFFSAIMIELA
MYCWIGNEIILKSLQIGDACYMSKWYEFSPRTNKILFLIMERSKRPLTISAYKFSVLSMSAYLKIIQCSYSYFTVL
RRVYMKD

>AglaOR17

MLKNTSNAIKIQKFILTLTGLWPEEHPTLYGKISGRISVVTAIIFTATLIAEAIKQIGNYVVLIEHLSLIISPTSFLIK
LIMFLRKTGQFVRLYRNLDMDIFNKHPDQFNTIKRKSETTSAVIGLSYMFSCFVITFFFCARPLYTSANMPVRFS
FEMGQYKPIVAVFQIFCMFNAALSNSCLDVIAMTLMGIASVQIDILNRNITNFKKECDESATGTDGYIRYLNH
CVKHHNEIIRYIGDIEEVFSLVFLAQYLTSGALICNIGFLLVHIRGLNLQFFNTVFYFAAMMCQLGMYCWFGN
EIIVKSSDTKTACYESDWIDCEVKVRKILIIIMERSKRPLFLTAGKFSVLSLNSFTTVVNSSYTYFALMQKLYSKT
NN

>AglaOR18NTE

HADLLGFLNTEMVALKIAGFYPLRGNKYKHLHTISATYFLSITILYIILACIHSFLNLTNITELSETVTFLMTILAY
LGKILNLFFYRKNLIKLEDMLQNPILTKLETEEEENILKRNFRYSRLFTNIFKAKSATAASVHAVYPLLGDYGSK
NFLFLLWFPFDPKDYYIPVYFFEMTLLLSSTWFDITMDTLNILMMDLCATQFEILKKRLIRIGTSFTGDEAIDDK
LRLRKLRKYIIHHNYIYSCSELVRDTYSIGEFCQVGCSVMVVCFGLFKLLIIPLKSAQFLMLVTYSTTMIYQISLY
CCYGQKLLNASDTVTEACYMSRWNDCSTQVQKYLAMIMNRANTPFLMKAGGIFSLTLETLMTIYTSAYSFF
AILWKVYHSEDQAA

>AglaOR19

MKRHIDLLKFIKEERTCMVFGGFYSIEKYKVLHNLSAILIMTVVSLYNLLGLVHGFQHISNVAVFSQSIAYLLT
GISFSCKMINLIMHKNNLLLLDEILQNPIFTELETTEEEVVLKNTLKFGQTLKKTYKLYTSATVTVQVLYPMIN
NPGHKNFPLLFWFPFNPEDHYYKVYFAEILMIFCICTFNVTVDLLNVLFMDLCAAQFELLKYRLKHFGREFH
GGEAVNDRTLYEKLNKIIVHQNLVYRFSKLTEETFSAGVFCHLACTVIVLCCAIFKAVITPINSMQFLMMATY
SFCMTLEVSLYCCYGQKVLDSSSTITEACFMANWYNCNVKVQEDLVIIMNRANKFVTMKAGGMFPLTLETL
MRIWSSAYSFLTLLMQIYNENY

>AglaOR20NTE

HIDLLDYIKAEKKTLAFIGYYPIKEKMYKTLHTLSAIFFGFFPMMYSVLGIMYGFQHMSNLEEVSELIGYLLTG
MSIVVKMMNLIWYKKNILELDDMLQNPILTKLESEEEEILLKNKLKLGQILKKAFKIFIAVTAILHTHILYLLVI
NIDPNAITLLLWFPFNIDDYLYEVYISEFFLVPSIAVLDAILDILNIIFIDLCYVQFELLKHRLKHFGRTSHEEEAV
DDTVVYDKLGKIIIHQNYAYRFSEIVEETFSVSIFCQLAVTVAVLCCAICKVVITPISMQFLIRTFYTFTIVLEIAA

YCCYGQKVLDASGTIAEACFMSNWYNCSVKVQKNMVMVLNRANRPVTMKAGGMFPLTLETLMSIWRSAY
SFLTFLMQVYKEDN

>AglaOR21

MQKFDLLASFDTEKYHLTLGGFYPSTHIKYKYLYVLSCIFNLFISWSQFLSMATFSYFNSSNLEKLSEILLFCMT
QFAFLNKLTNFVAQKTSLMKLEVMLQSYLLTNVTQKEIDVLENHIKEGRLLAKVYRVLCFLVVLFYALFPFLD
DRSGESHKFPLPCWFPFDEIKYYYQVFFMEILSIAVGAWINSNIDILTVMMCVLATAEFEVLRNRLATILQPLST
SASIAEGDALVKTKLRDCVNQYDELLCLVNQIEITFSKGIFVQFFCSVIVICLTGFQMIVISFNSMQFFLLIVYFSC
MMCQVAMYCWYGHTVMESSDKIRDACYLADWNESDLIVQKSLLMIMERAKRPAILRAGNFFELNIPTLMKI
LRSSYSYFAVLQRLYGKK

>AglaOR22PSE

KFDLLASFDTEKYHLTLGGFYLGTHIKYKHVYVLSCIFNLFISWSQFLSMATFSYFNSSNLEKLSEILLFCMTQF
AFLNKLTNFVAQKTNLMQLEVMLLSYLLTNVTQKEIDVLENHIKEGRLLVKIYRALCFLVVLFYALFPFLDDR
SGESHKFPLPCWFPFDETKYYYQVFFLEILSIAVGTWINSNIDLLTVMMCVLATAEFEVLRNRLATILQPLSTSA
SVDGDAVVKIKLRDCVSYFGKPICWILVNQIEVTFSKGIFVQFFCSVIVICLTGFQMIISFSSMQFLLLIVYFSCM
MCQVAMYCWYGHTVMESKSLVMIMERAKRPAILRAGTFFELNIPTLMKILR*TYSYFAVLQRLYPLNI

>AglaOR24CTE

MENFNLLDFFATEKLYLTVAGFYPAELSLSRYLYILSALVNLSTSWLQFLSLITFSYFSLNDLRKLSEILLFCMTQ
FAFLNKLTNLIWHKSSLKELEAMLQKTVFTSVRIEEKHILTNHLQGGKLLAKMYRVLCFLVVLFYALFPFLDE
RSDKGHTFPLPCWFPFNEGDYYYPVFFFEVWSIAISATVNSSIDVLTIMTMILATAEFKILNRKLTNIASYSGVD
GDYDDDVEVRSRLGECIIHYDEALNLVRHIELTFSKGIFVQFFCSVMVICLTGFQMLV

>AglaOR25INT

MMETFNTMDFIRAEKLYLTLTGFYPAEPGLRRYLFILSALVNLSISLLQFLSLITFAYFNLNDLRKVTDILVFCVT
QFAFLNKLTNLIWHQSSLKELEALLQKPVFTSVRSEEKHIKTSHLQGGKLLGKMYRGMCFLGVVLYTLFPLLD
KRSDKEPKFPLPGWFPFNEVDYYYPVFFFEILSIAILSVINSSIDLLTIMTMILATAEFKILNRKLTNISTSSGADG
DYDDDDEVRSRMGECIIHYDEALNLVKHIELTFSKGIFVQFFCSVMAICFTGFQIIVCYKTRWDKCSVEVRKSL
MIVMERAKKLATIRAGNFFTLNIPTLMTILRSSYSYFALLQRVYGNN

>AglaOR27CTE

TFNTLDFFAAEKLYLTLAGFYPAEPGLRRHLFILSALVNLSISWLQFLSLVTFSYFNLDDLRKLTDILVFCVTQF
AFLNKLTNLIWHKSSLKELEALLQKPVFTSVRSEEKHILTSHLQGGKLLAKMYRVMCFLGVVLYALFPFLDER
SDKEPKFPLPCWFPFNEGDYYYPVFFFEIWSIAISAVLNSSIDLLTIMTMILATAEFKILNRKLTNISMSSGVGGD
YDDDDEVRSRLGECIIHYDEALNLVRHIELTFSKGIFVQFFCSVMAICLTGFQMLVISFSSMRFALLVVYLLVM
MCQVAMYCWYGHNVMDSSDEVTSACYMSRWDKCSVEVRKSLMIVMERAKKPATIRAGNFFTLNIPTLMT

>AglaOR28

MSTIEDYDLRNAFKIERKLLLLCGIYPNEGRINKKLYNLSAFCHISFSLLITLSMVIFLAMNMKNILSVVEALLFL
ATQMAFLCKLFNVLNKKHKLLEIEDILANPAFYGYPKEKRHLIEDSVRFTKIFGMCYRSICTVVSITYAIFPLM
DDDEWALPLSGWNPIQIDTKFKYWTIFTFQWVSYYMSVYINSGIDILIYILITVVTSQFEILKDNLTNIRYETDTA
KRDFAKNVVLHYGILKLVRVIEDTFSYATFFQFFSSVVVICFTGFEMMIVPPNSIQFISMCTYFNAMIFQVAMY
CWFGHSIIASSDKINDAIYMSNWYEADLSLKKSIMIFMEKCKKPVVLTAGKIFPLSLVTFTSIMRSSYSYLAVLQ
SMYGQE

>AglaOR29

MELKTPYFKKHLKWLLVLGVDIIPVKNVWYKYFYKLWSLFIIGFVVLYTLLEVIDILNTSDFNSMTFGLCYSAT
HLLGLAKIIILIVKKKKVREMLNELESGDFLPKIERGGEEEIRLINIAVTRCARHAEIFNLIVYSIVSIRCLYALFDT

GYNDELFDEQLNTTTPIHTRILPYRIWLPIETTKSPIFEIVFFFQAFTLTLYGYYIGMMDSMVYGMMIHMNTQY
LILKRVLERYVSIATNMVSKNLLDKGVKDIRNGVISLPVGYERIDFLSEPVQEKVREIVHNCAKHHVHILEFCE
KVEKEFSYLMLSQFLFSLYTLCFQLYQLSLMANVLSFDFISMCCYLTLLMYQLFCYCFYGNEIMVQSEKFSEAL
YNSDWLVLDNSTKKSLLLMMMRAQRPIRFTAGKFALLSLQTFMAIVRGSASYFMVLRQMNR

>AglaOR30

MEEQYAEDFFVANRWILQCAGLWSPDGQHKVLRMLYKLYSIGIFLFVNIFFTATEFISLYYTYGNEYDLIKNIS
FALTHLMGAIKVVFFYFQKHKLLAIMDTLENKEFRYDSCTEKSFFPGLISRRYKNIGVKYTILFFILAHATLFSSY
LPPTISTLMNSSRRGAEGQGLYVLPYYSWMPFKYNTDDSFLLALGYQAIPMFSYAYSIVGMDTLFMNILNSIGF
NLEMIQGAFLTIRNRIIKQTGKSIYKSSILLDSEELKLKLCNEMKKICHHLQIIYKVCEDLENVHKYLTLAQMT
ATLFILCSCLYLVSSTPIASKQFYAEIVYMVAMGFQLTLYCWFGNEVTLKAAELPLYIWQCDWLTADNNFKA
SMILTMARARKPLYLTAGNFAPLTLSTFVSIIKGSYSFFTVLKSTNE

>AglaOR31CTE

MDSYAEDNFFHVRWILRCAGLWPPATKNKAVQALYRAYAVFIFLFVNVYFTATELVSLLYTYGNEDDLIKN
LSFALTHAMGTVKAVFFYVKGDKLIELMKVLESQELRYEPCEERNFFPGLISRKTKAAMKKYTLICFALPHLT
LFASYIPPAATAILVMFEGNGTRQLPERLPYYSWVPFGFDTGPSYLIALGYQAGPMFSYGYSISGMDALCYGL
MLCIAGNLAIIQGAFLTIRERSLKRIKGPEWAADGLYNSACLNAAMNGEMRSICRNLQTVLNSCKDLEHLQK
YVILAQVSTIQMIICSCLYLVSTVPINSKQFYAEIVYLVAMGIQPFFLCLFGNEVTFQAKYMPDYLWQCDWLA
ADKKFKKSMIITMARLSKPVSLTAGNFASLTLTTFVS

>AglaOR32

MLSEDISYPSNFFHVNEIILLISGLRLPNKHDSLLKKCFYYVYAIFLYGASITFFVLEILKLEDTVKDPTKFFSHIGL
LLTHLVGILKACLLLFKYDEIQKVMDLLQDKGFRYETSDGFQPGLMVREAKRKSFKVSVLVFSLYTLAGVLAF
ASAWTSIIVKVKGQQFEVNITCYDFLPFYFHIPFPVETKSQCENVFTFMGIALCAFAWFIASEDAIFCALLNCLK
TQLDVVGEALLTIRLRTLRKIDFPPGYAGLRDDDCPELERSLYSELNGCIKHLTSLLQARDELEGIFTYIILAQTI
CSILIISSCLFVAAMVPISSPTFFSQGEYFLCILLQLWLICNFGNEITFSSSEIIRSLYESDWFSCSLRYKKSMIMIMC
RMQRPFYLSIGKFTPLTLITFMAVCRGSFSYFAVFRSM

>AglaOR33

MSGMNYPKDFFYVNKILQRVTGFWLPGKEIHLILRVLFYLYITLAYSVSIFFFICEIIIVSETVKDLNKFVRQFGM
LFTHVVGILKFYILVFRRKSLQKIMDMLQDKDYVYEPQGTFQPGLMLSKAKRLTSFVSVMVFILYNFVGMSAQ
ISSTITLNEVVKDDHLPGNLTCYDFLPYYFYIPFNAVTKFQCHVAFTFMNVSLDVFAWIIGTHDGFFVTLMNC
LKTQLEIVQCAFRTIRERCEKRLDLPENYKIFVDESNPTLEKTLYDELTHCTKHLNILLQVRDDIENVFTYVTLA
QTLASLIIFASCLYVASTVSMTSPEFFAQVEYFLCVLVQLSVICFFGNEITTASAQTGVSLYECDWFSSSLRFKRS
MILTMSRIQRPVYYVSIGKFSPLTLATLVAVCRGSFSYFTLFKSVQ

>AglaOR35

MKQHSSYPKNFFETNEILSYLTGLWLCKSNNISKWKKYLQIIWILIEYTVGTIFLILQFLILSETSKDINKFFSHIG
LLFSNALGTVKIAFIILRRNKFKKFMDILHDENYQYESLDEFKPSLIFLKEKRFSKVISISVLCTFTLVGVSAHASA
VRVIRKEINGTIFEGDYNCQDFVPYYFLIPFEVDTTTKCEYVLFFMDISVCAFAWLVGCYDGFIAALLNCVKTH
LVILKGAIRTIRKRVMRRLDLDENLEIFHDEHLPLFEEELYKEFKHLVMHLNIILNVTEEIEKMFNLMILAQTLS
SLFNIASCLFTAAKEPVGSVMFFATLTYFLSMLIEFALVCWFGSEITTASEELMFALYDIDWFSASHRFKKSLLL
TMSRMQRPIYLSIGKFFPLTLKATVSVCKASFSYYTVFRRVED

>AglaOR36

MMEHSGYLNNFFKTNEILCKIMGLWLYDSNGESRWKRYLQICFASFEYTTAGIFLTLQFLILNETSKDINKFFS
HIGLLLSNVLGAAKVAVMVLQYSRFKNIMDILHDKNYQYEYTKKFNPSLIFRKEKRFSQLCSISMYCIYAFVG
LSAHVSVAATIRNKVNGTKFEGEYSCQGFVPYYFLIPFEVDTTTKCDYVLFFMDISVLFCGTLVGCYDGFLAA

LLNCVKTQLLILQDAIRSIRERVMQRLALDERLDIFHDETLPKLEEELYKEFKHLVKHLTIILRVADELESMYNL
MILAQTLTSLFNVASCLFSASREPAGSAVFFATLVYFTSILIELGVVCWFGGEITTASEDIMFALYEVDWFSSSQ
RFKHSLVLTMCRMQRPIYLSIGKFFPLTLSAMVSVCKASFSYYTVFRRTDE

>AglaOR37

MEYDSNFLKLVKKVLIVIGIWPMNLSIYANLAYRIYHGMVLTYFTIFVVTLAIATKDILHVNPEKQAYVIQYGF
SYALFLWKVILCQKESIRKLMKIISTKERLITNSNDNDIKNIYRICLQHNTTVFYIALLVIVAVVVLFAVTIYFEV
KSWPAPEGISNCSVSARELPLPQAVPFGIDKYFKLVYIFQVSSRVFAAFIFLGCDTLLNYLIYFPAAYIKVLGHKF
EHIGDQNGNRLYSSEAFLKKLILEHKEMIEYVIHLNDILQWLLSMDFFILSYHISLTLMGIMSLLESPVFNMDF
MITLFFSVSYLMGVFFQMCLLYYNCNELIVESLEIPNSIFRSNWYELPQAVQRSLLIVIMRTQKPLEMKIGKLYA
MSNDLIVGFVKAGFTYVLLSHLDFQQA

>AglaOR38

MEDNIHMKFAKILMIILGIWPVKLTGWKLILYNAYFYASYVYYIMYDISQGAIIFVARGTFLQTVGNLGVTIVY
IINIYKVFICRSTSVKKILQEIEAKERLILENDDDTIKKIYYDHVQSSMLAMKYYVSLGSVGISLYFIAPIVRNLVE
EDDHKYLIFTSWFPFDSDKHYAVAYLIQFFGGFYGYAYIVYCGSFFFCMLKYCVGQIKILQHIFRNLRQYTVKY
SRNNDLDEKCSEEIFVKLCIREHQYIISLVKRLDDCIKTLMLLEFQISSFQLSLVVYQILQSSGWTIERTAVLSYLV
TLCSQLFIFYWNAHEIIIESTELAQAVFENEWYTFDRNIQNLLIFVILRAQKPVGISIGPIYKVKMDALIGIFKAIY
SYVAVIQK

>AglaOR40

MGFLPQVLHFKVAMYFSVCIGLWPLITTKNKTVRKLYEIMSKLHYIYFNLFCFTQLVQLVVLLMEDQVNYTE
VVNNLCILFIYFNSALRCKSIKGKAIRNVIKDVGKSEKKLWESMDENLKKIYNENVKRNNLFCKIFAGNTLLT
NVGYAIWPLFMEKQTVEINNTTMEVKYLPISAWMPFDVQKHYVSAYLLLLFNCNYLCTMFFISTEALAFGLM
TYPLGQIHMLNHILRNFRKYKDSLQRSMNLKEEAASETQLRECIVKHQDIIKYVDIFNAEMKHTYLLDFLQCS
LQLTCVLYRFMTQRNLVLILINLQFILVMLIRLFITYWTANEVTLQSLAVSDELYSMEWLDETQKVKKMILFMI
MRGQRPLILFIGIFKAMSLEVFVTIIKVTYSYISLFYRHN

>AglaOR42

MIFPKNEHLKITIYISAVLGVWPFIFEDNPVLRKIYDVYSKCIFCYYLLYVLTAIIQLFIIVTDEVLDVDEIVANLCI
TLLNFVAILRVNAIKTERVKNIIQNVFKLEEKMMNSGNDEIIEIYNRHARQNQTCNKIFLVNVYLVAILYFIHP
LYVEDTIKYYPNRNETVVIKALPLSTWYPFDQQKHYVLTYIWEQIDAFMATTFIASSDIFAFSLIIFGVGQIKILK
LILSNFQEFAMNIKDQLHCSQEEASYITLRECILKHQEIIEYINEYNLIMKNIMVLDFLLSSVELASGVLTLLVTE
MTLPNTIYSSQLAFSLFLRVLVYYWYANEIMVHGSEIGMALCNSNWYEESERVQKMMVIMLMRCNRELYLEI
GPFAAMTLRTFLGVLKATY

>AglaOR43CTE

MIFPKNEHLKITMYISAVLGVWPFIFEDKPVLRKIYDVYSKCIFCYYLLYVLAAIIQLFIIVTDEVLDVDEIVANL
CITLLNFVAILRVKAIKTERVKNIIQNVFKLEEKMMNSGNDEIIEIYNRHARQNQTCNKIFLVNLYLVDILYFIH
PLYVEDTIKYYPNRNETVVIKALPLSTWYPFDQQKHYVLTYLWEEMDVFMATTFIGCSDIFAFSLIIFGVGQIKI
LKLILSNFQEFAMNIKDQLHCSQEEASYITLRECILKHQEIIEYINEYNLIMKNIMVLDFLLSSVELASGVLTLLV
TEMTLPNTIYSSQLAFSLFLRVLVYYWYANEIMVHGSEIGMALCNSNWYEESERVQKMMVIMLMRCNRELY
LEIGPFAAMTLRTFL

>AglaOR44

MIFPKNEHLKVTMYANALLGVWPYIFQDNPCLRKLYNVYSRFTFYYFILFIISAIMELFILVADDENRTEEIVA
NLCITLIYIITAVRVYVMRSTTIRNLIKQILATEDAILKNDDEEIMEIYKFHARQSQITNLIFIVNITVETIFFFTHPL
YVDEKIKFNKATNETKVIKALPLSSWFPYDPQDHYLASYMWHIFDGTVGASYVMYTDAYNFSLIIFPLGQIRIL
THVLSNFPRYVLKVKDQLQCSRDEASFITLRECILKHKEIMRYLQEYNDSMKNIMLLDFLQSSLQLASVVIQLF

VTKATVFNIIFHGEFTICMLIRLLVYYWYANEIMLESLNVSTAVYECGWYDEPQNVKQMMLLVIQRANKALE
LDIGPFTTMTLRSFLGIIKATYSYLMVMYR

>AglaOR45

MIFPINDHLKITMYANSVLGVWPYIFQGNPKLRKLYNIYSKFMLSYFLFFLLTALIQLYILITDDELKVEEIGAN
LSITLLYPITVRRVLVIRSTKMKKLIKKILAAEEAILGGKDEEVLKIYKFHAQQSQFTNLLFIVGIAVQAVFYFTH
PLSLDDVVKFNNVTNETTIIKALPMSSWLPYDPQKHYLISYLWHMFNGLIGASYVTVADAFSFSMIIFPLGQIR
ILLHVLTNFSEYVGKVMEQNQCDRDQASFLTLRECIIKHKEIISYLQDYNHSMKNIMVFDFLQSSLQLASVVIQ
LIVTTPTFFNVVFYGEFAICMVIRLLVYYWYANEIMLESINISTTVYECGWYDEPRKVKQMMVMVILRANKAL
GLDIGPFTTMTLNTFLGIIKTTYSYMMIMYR

>AglaOR46NTE

FPKSEHLKISIYTSSMAGIWPFVFQENSTERRLYDIYFRYLFYYFILFIFSAIVQFFIIIQDYETGVQQVMNNLCVTL
PHSICAMRLWSIKGDTIQNLIKGILATEKGILESNDEGTIKIYKFHARKSYITILLYTSNLTMAAILIFIHSLHDEN
KIKFDKATNETKIIKSLPLSSWLPYDPQDHYLISYLWLTFDGMVGAFYMMYTDAYNFNLIIFPLGQIRILTHVLS
NFPRYVLKVKDQLQCSRDEASFVTLRECILKHKGIIRYLEEYNDTMKNIMLLDFLQSSLQLASIMIQFVLTKAT
VFNIIFHAEFVFSMLLRLLVYYWYANEIMLESINVSTAIYECGWYDEPKKVKQMMLLMIQRANKVLKLDIGPF
TTMTLGTFLSILKASYSYLTVMYR

>AglaOR47

MIFPKNEHLKISIYTSSIVGIWPFVFQENSSRRRLYDTYSKFLFYYFIIFILSAIIQLFILIQDDETEVQQIMANLCITL
LHVITAMRVWSVKGETIKNLIKDILATEEDILNSNDEEVIEIYKFHVRQSQITNLLFIGNIMLETVCYLINSMYA
EAVVKFDAATNETILVKALPLSSWFPYDTQEHYLMSFMFHVFDGSVGACYVMFTDAFFFSMIIFPLGQIRILQ
HVLSNFSEYVAKVKNQMQCTRDKASFLTLRECILKHKKIIGYLKDYNNAVKNIMLLDFLQSSLQLASLAVQL
LLSKPTIFNIIIYGQFIICMLLRLTVYYWYANEIMVESIEISSAVYECEWYNEPHQVKQLMSLVILRANRPLGLDI
GPFSTMTLNTFLGIIKTTYSYMTMMIVYR

>AglaOR48

MGVTFLHYLKHTLLITGIVPYDMLTCNKTRYKLFRNTSHVFYLFLLTFMYLEFPYIWNDSEKFIESLNLITTYTV
LAIKMFICRNTNFGGLMCDMVAVEQQTVASRDDKVLNIYKRYVKRAKYIQLFNIFVYVASAVVYSAPYILKY
LILYKIKEQVDGEERKLPFYMWMPFDKKNHYLLAFAINLYFLFITCIYYSTVPVVIIVLLLYTSFRLKVLGCLLK
NIKIYMSKFKENDENITVEAVIRYCVLEHLKIMQITNTLNNCLRNVTLLEFIVLSAQIALIAFEGFTSRSANTVV
VCIVHVLLLLVHMLLFYWHADEIRHESMAISEALYESDWYEYSRTTSSTIHIMMMRSQRPLSLSVGPFGEMSL
TMALKILKGVYTYMTFLQHSYGQTSSLGTNKYPFR

>AglaOR49NTE

TGNISLLCYVKPIMIFLGALPYQLVDINKRFYTSYKYVLQSTFFLVLIFFYLETPNFHGPMEKLFQNVSILSTYTIL
YVKGLVCMSKNMSKLLLDMVYYESNIKASKSEEIFNIYLSAVKRSKIVQVFYIILTYLTGISFFQKALNYAMSSD
EIKFNSTLEAHESLPYAMWLPLDEKEHYLAALCIQTLCALLAINYYCFVQCILVILPLSVTLRLKVLGNHLENI
KYSRNNSLQTSEADDLDCIKDMVVLCIKEHTNIIEICSILNNTVKYLMLLEFLMSSAQISLALVLLTTTHETNEQ
LFALIYLFQLLTQLLILYWHGDEIREQSVAISNVLYGMQWYEYNKSINTSIHIMMIRSQKPLSITVGPFGEISLEM
AVKIMKAAYTYVMFMKQVYE

>AglaOR50

MDSPFLHYVQYLMVVGGIFPVGLINSHKRIYILYRYVTHALLFLFLFCLFDGNKDFHGSKEMLFKYINDRILVI
VIYINLVTSMSENFDELIFEMIAYEAELADVGTEKHLKINEQIVTKTRRVQVFYMSNILACYVALVVPAFVEYA
VVQASEKSTNITINTYTATPYEFWVPFEESKHYLALLLIQAYYFALMGVSIYCPFQIILINLFLTVILRLKMLRAN
IESMNDIDTLDVNKFIRIYAKEHIDIMRNCRFLNDTMKYAMLLEFLITSGELSMFIFIVIMTNSPNSKMFSGACI

VNLLVKILILCWHADQIREESIGISDAVYELPWFEYDKSDVTSLHIIMMRSQKPLTLTIGPFGTMTLELAVKIFK
ATYTYVTFMQNVYGKLD

>AglaOR51

MDSPFLLYIKHLMIIGGTLPVDLINSRKRIYIFYRYVTHTFVSLAGIYWLGGNMNIDGFDRTLFNYIYDRIFVMIS
YVNLAITTSGNFQKLIYEMIAYEAEVLDVGTEKHLKIHEQVVKKCRTLQVYYISILSSCSIAFVVPAFVEYALVE
SSEESTNSTINTHNYDLWIPFEESKQYLVWLLVQSCYTLIVTCIYCSYQTILINLLLVVILRLKILRARIENMKDIE
TLDAKRFIRIYAKEHIDLIRNCKHVDDTVKYVMLMEFLFASLRLALSIFLLVTTNTPNSKIYFGSVTVNVLINILI
LCWNADQIREESIGISDSIYQLPWFEYDKSDVISLHIMMIRSQTPLTLTTGPFGTVTLDLAGKILKATYTYATFM
HQMYEN

>AglaOR52

MSQTIHLKLQKYIMLLIGTWNFKSYNNAFLVMYKAYSYFIFWHYVVMTQLVMVSIPIQWDCKSRVIELICFYI
QYTNNIIMMVLSKYSNKMQKVFDHILDYEDIKLRSTTDVEQKIYFKYAKLNNRVSLFVTIVLLGTSVYWYTTS
VRYSLFGQPTDVCPLTKGTIYQIWLPSIIRKYHWLMILNDIVFFMSVINITLYREIIMFAICIFMLGQIKILQQNVR
DLEKNSEVVRKSRNVSYDDGLLISVVKCAQEHQQVTKLMEIVQSATSIFILVLYFSNTFEMAAYLFQLISEKSIY
NILRPLYVFTLMISQLYIFYKYTNEILVESTALCDVIYNETNWIDYNQAVKKNLLLMMRRSQKQLSFKAAGLG
DMSLQTFTNLIKLCYSIVACLKTAYDV

>AglaOR53NTE

VKHDAIYLNIHKYIMIIVGKWYVGFQNKALDKFYRLYSMFFETFVILITQQIFVSIIVYRSCPAERVIELICYYIQY
TNMYVSSLLCRRASMRRVYEYIFDYEKTKLHKENDVSVNIYFKYTTLNRRMLVFFALLTNITGAIWYILVVRH
TLTEHDTTLCALKRGLNFQIWYPFDMYNKCYFLTVLTDLMFYCSVVSIHIYNKLTPVTFMLFELGQIKILQNM
IRCIDKNASELASFEDIDSDEAVLITMRGCVKKHQEIIRLLHMMDNACKKIVLIIFFSNSVELAAFIVKLLTETN
TLDIFRTFGILIMLITQIFMFFWYANEIQVQSTAISDILYNNTDWINYNKPARMRMLLMMIRSQKPLTFNAAAI
GDMSIDTFKRIMKLCYSITAFVKTVFNSRTEM

>AglaOR54

MGDDHTYLPFTIKILNMINMWPNEAKKCDFIKKYYILFGILLSITAIMADFILQFYDDDFSFTSLVDSLIGLSALL
SVIYISVCFIVKDQEIKRLTENLKIFEEYIPKVKIEETEDKAKFHTKIFIIYGIMGNIIYDCLPLLTYEDCHKNRSKH
MIKYGIPCGGIVHYVLPYKQDTFPLAEITIVNQMLISTVGTLIVMTVTMLVCGILIHVSANLKYLRSMISNLTYT
PNNRIMQHVNSCVKYHTMICDTMMLVHITWTSFIISVLGFEIIMDTNYSNSIRFSLHLGGWLGMLFLICFYGQI
LMDDSFAVSEALYEAEWYEKPPIVRKSLVLILLRSQRPLVLKAAGVNVMSLATFLGVLYNAYSYFTLLLKIKP

>AglaOR55

MDILRVNIKTLKLFLLWPSDNMSWVTNIVLSVIHQTFIFLSSTPTFSAVGYQIAVGIDDINIIIESIIGCGDYLGFLF
VYICFRVNVPKIKKTIKRSSVFLEYCGNSIVEEAEKKVRFYTLGLMIYFFIGLTINTILPLLDYNNCNERRLSDYY
RLHDPCGMPTRVWVPFEINTPTLFYLLIFLHNDACVHISYTVLCITMILIGLLIHITAQIKNLRKHLIDIFNTEGS
DSESDELIENTEEKLKFCIKYHNTIIGYTNEVFEAFNITLIVHVCLTSMVVAVIGYQIISADNLMDKLRYFLHLM
GWIGMLFITCFYGQMILDESITIADAAYQSEWYNGPEHFKKMICLIIVRSQRPLLLRVASIGVVSLETFVSVIKT
AYSYFALLLTIAK

>AglaOR56

MYKAEREEPFYTSLNYLSRMRQIPVSMKKSYMRKFITLFLMSKVVGLFCTAIGPILHLIISIMRNSGTNVTEDISII
FGASGGGIVSLLFTLKYKNWSNFFKDLVDTRQYTRPRNLQARATRLNHLGKICFFCAWWVTITYAIAAWFSS
KGCQDTNDPGKFGEICPTFIPIWLPFGTNDVAAGTVVFVLQTFFVGCLIAQSFLAECFVMYESTEIFICHISELK
SQLKETLDAPFSVETRDKLRLCVDYHVYILNLGYRLSSLIKYTAGHMSLLSALVCGCVANQMLNTKPMGSLF
FLAAWLFALFCFCHSGQRIKDKTLSIADAIWNTKWYKADVKTLKDVQFILLRCQKPICFEALPMGVTDYAFY
FMMIKTGYSFFALFRQSL

>AglaOR57CTE

MYKIEKNGAFSNSLNYLKMTYQIPVSMDKSYLKKFVAIYLLSKVVSFFFTTIGPLLFLIVIIVTHSETDISEAASIII
AGTAAFLVNVLFTLKYKHWSNIFKDLEDLKKFGKPASYQKTVNFLNRLGHISIFGSSMVIFIYGLVAWYNTKF
NKEFKEDFNQISPVVVPIWLPFGANNLTAGTIIFTMQFIFSGILTCQTYLIQSFIIYESTEILICYISHLNDNFKETF
GASSRVEMRNRLRFCIGYHVHILKLGYRLRSLVKYTVGHVSLLSALVCGCMANQMLTTKPIGSFILLIAWLIA
VFSYCHAGQRIKDKSMSIGNALYESKWYNADNETMKDVQFILMRCQKPICYEAIPLGIIDYPFYFM

>AglaOR58

MYKIEKNGAFSYSLNYLKMTYQIPLSMDKSYLKKFVAIYLLSKVVSFFFTIIGPLLFLIVIIGTHSETDISEATSIAL
GGTAAFLVNVLFTLKYKQWSDFFKDLEDLKKFGKPASYQKTVNFLNRFGYISIFGSFMVIIIYGLTAWYNTKLI
KEINEDFDQISPVLVPIWLPFGANNLTAGTIIFTMQIIFTGLLTGQPLLIQSFIIYESTEILICYINHLNDNFKKTFE
ASSRVEMRNRLRFCIGYHVHLLKLGYRLSSLVKYTVGHMSLLSALVCGCMANQMLTTKPIGSFTFLIAWLIAV
FSYCHAGQRIKDKTISIGDALYESKWYNADNETMKDVQFILMRCRKPICYEAIPLGITDYPFYFMMIKTGYSYF
TLLNQST

>AglaOR59

MYQVQRNKPFYWTIKALHIVMAIPVSFEKSYLRRFYIKNVVVRFGAFACLAILPSCHLISSIKEHNGADISEDIS
MILGGVGTVVTNTLLAFKYDLWSTFFMDLENCGHLFGEPSTLKKRKKELNIYSLTYTIYTYFGQFVYGLEAAL
KWSDCKKLNEEKGLKEICGAFFPLKLPLDVTSPLGSAAIFALQFSFTIFSIPSGGVICFMVLEVTEVVICYYKELK
SNFERVFDVPDEEKRKERLRFCIRYHKHMLGLTRRLQKMVKYTAGHLSLISAIVFGTLGNQAFKSKPLGAMIF
LVGYIIALFSLCRAGQRLMDETQAVSDAAYNSKWYDADPKIMKNIVLIIARAQEPEMLDALPLGSFCYPLFLL
IVKTSYTYLTLLQQTT

>AglaOR60

MYEISKEKSFYSSLTILRYTYAIPSATEKSGLTWFRFIFIVIRLASLLNFVILPCLHLISTVRAKTGVDISEDLSTMFG
AFGFITNAVTFLILYEKWSQFFIDVEDCNRFEIPEGIEERKKKLNLYSLLYALYAICGVLIYGVIASSETSYCKRM
NEEHGLSETCGMFTPIVLPFDGSNVYIRSIIFGIQMLLGMFTLPSTALISFLLYEGVETLILHITQLKKSFIEVFDV
ASNAERKKRLRFCVYYHINILSLCQRLSTLGKYTSGFLCMACALVFGCIGNLILKSKPIAGISYLLEYVFALFLL
CHGGQRLLDETLSVADVIYDTKWYDGDVQIMRDIRFILARSQIPVMIGALPLGCMNYALFLMIMKTSYSYLTL
LTQNT

>AglaOR61NTE

NTGVDVSEDFSNMFGTFGANVVTISFLLLYDKWSQFFIDLENCNRFGIPTDIEALRKNLNLYSVLYALYTVVG
VAIYGVIAIAETSHCKKFNEEHGSNEICGTFTPLVLPFDGNPIFVRSIIFVIQAFIAMTTFSAPAVVCFMLYEAVE
TLVAYITLLKQSFINVFETTSEAERKDKLRFCVYFHDNMLSLCQRLSTLGKHSAGFLCMTCAVVFGCIGNFILK
SKPVAGICYFLGYVAALFLLCHGGQRILDETLSVADALYDSKWYDGDVQIMRDICFILARAQIPATLGALPLG
VMNYPLFLMIMKTSYSYLTLLSQNT

>AglaOR62

MEAEDIVIMENSLRYLSRNLVFPRKKDVNNPGVSFHLKFVALNLSTVMFLTGNILHLMINIKRKTYINLDLDL
ALAISLLGSYYFNFSYIRQVKNTINIYKQLSDLRSYGIPKDFYATNKKLNNYSKYHYIYIVSAVLGLSVAPLLEY
KKCRKENVVKNINEICGLIGSIWLPIDLDSTPYKQMYYIFQVYSSFVIYQTSSLISFSIMETVEHLILRLNHVKNV
FLEALMEREHGVRREKFRRAVKYHVNIMRISKLVNTSFNMCMFVHVLLTGAILGCVGYRLLKSYSLGAICLF
VGWIVSMIMVCISGQRLRDQSLSIGDAICRSNWLDLNKELQKDLVLVLLRCQKPIFLNAGPFGYMTYAMILT
VLKTSYSYLTLLSSTS

>AglaOR63CTE

MDADDIVIMENSLRYLSRNLIFPRKKDVNKPGVIFHLKFIALNLSTVMFLTGNILHLTINIKRKTYINLDLDLAL
AISLLGSYYFNFSYIRQVKNTINIYKQLSDLRSYGIPKDFYATNKKLNNYSKCHYIYIVCAVLGLSVAPLLEYKK
CQKENIVKNINEICGLIGSIWLPIDLDSIPYKQMYYIFQVYSSFVIYQTSSLISFSIMETAEHLILRLNHVKDGFLD
ALMEREHGVRREKFSRAVKYHVNIIRISKLLNTSVSTIMFGHVLLTGAILGCIEYRLLKSYSLGAICLFVGWLIS
MSMVCIGGQRLRDQSVSIGDAICRSNWFDVNKELQRDLVLVILRCQKPIFIDAGPFGYMTYAMILT

>AglaOR64

MEEENTQVLENTLKYLRFFLIWPQEGTNFNLGKAFYLKFVLLTISTSFILYGSVAHMIVTLYEKNGAQIDLDIA
VTISVIGSYYFNFSYLSNIKEIADIFKDLANFKTFEMPSGYKHFLKKWNELSTFHFIYYVILCSFCLVVALLESRRC
LAKNALKGTHEICGLALNLWLPFDIDYFPLKQIICCYQIYSVFFSYVLSSTVSFSIMESMEHVIFRLDYVKQLFVE
ALRQEEQVIRKHIFKHSVQYHNFVIRLAMRFNNCYSPCIFIHVLLTSVILGCVGFHLTQSPHLLTIGIFVGWYM
ALIMVASGGQRLIDEATSIGDAIYSTRWYDAENEIQKNVVLVLVRCQKPIVLRAGYFSFMSFQMIVTILKTSYSI
ITVMKGIDLN

>AglaOR65

MVTSIVYFATTLKIFRIYQVFPAEGHELKPGKMFYIKFGFTCLFASAIITGSFMHFVYSLKSRNNIYIYKDICYVLS
MLTGYSFFVLFSFNIKSAAKLFILLSDFNKFGKPPKFDERNEQLNRFSRYHYIYINLASFAFLSVTNIFKSHQCIK
ENQEYNFNEVCGLITNAWLPFDIDYFPLKQIYATLQVFSIFYVYVTAGTVTWLVVEVVEHISVRIDHIKHLFTS
ALKEKDPEEMKRNFRFAVQYHSWFLELEDELNKSFSIPMFSMMLLGAPILGCAAFGYMETGANSLLVICLSW
FNALALVSFGGQRLINENLTIAEEIYNSKWVDVAPILGKDIIIILTRCQKPMKLRAAAFGVMNHAMIVSVSRA
AYSYLNILRASSK

>AglaOR66

MSKPQIVCLQNTLKIFRFYAMFPEKDKELNPGKYHYVKFAILACFSSVVLIGSFLHLIKSVNANNYLSLDTDLI
YTLSLGITFAFSIGFLYKTEALVEFFLFLSDFEEFGKPLDFDRDNTFYDRLSIYHYIYLETLVSGIVIISTYLNMNKC
QKLNLEYGLDEICGLFSYTWMPFEIDYFPMRQIFLVVQLFGAHHFYMIAGMCAWFVFETVQHFRIRIRHVNF
LFQEAIKEEDPQRCREKFNHAARYHASLLGLEDKMNGAFGTFMFTHMVITAPIIGVGVFAIVSGGSVSSFLVC
LGWFGGLAMACFGGQWLQDECFAVGTDLYEADWLHCPEDIKKDIMIVIQRSWTPMYLRASSFGIMNYRMF
LGVLKASYSYITLLTQT

>AglaOR67PSE

MAKLQIVCLQNTLKMLRFYTMFPEKGKELNLGKYQYVKFAVLAFFFVSVILIGSFLHLIKCVSVAFSLSFLHKT
EALVELFLFLSDFEEFEKPVDFDRDNTFYDRLSVYHYIYLEILVSGIVIISTYLNMNKYQKLNLEYSLEEICLFSYT
WMPFEIDYFLMRQIFLVVQLFGAHFYMIAGMCAWFLFQEAINEENPQSCMEKLKYTIKTRIRKKLEDIINGAF
GTVMFSHIATTALVIGIGVFAIVSSREAILVHFWYVWAGLVDCFVGQWLQDECFAVGTDLYEADWLHCPEDI
KKDIMVVIQRSWTPIYLRGSSFGIMDYRMFLGVLKASYSYVNLLTQA

>AglaOR68

MAKPQIVCLQNTLKIFRFYAMFPEKDKELNHGKNHYVKFAVLACFSSVVLIGSFLHLIKSMSDNNYLNLDTD
LMYTLSLGITFAFSIGFLYKTEALVEIFLFLSDFEEFGKPVDFDRDNTFYDRLSVYHYIYLETLVSGIVIISTYLNMT
KCQKLNLEYGLDEICGLFSYTWMPFEIDYFPMRQILLVVQMFGAHQFYMIAGMCAWFVFETFQHFRIRIRHV
NFLFQEAIKEEDPQRCREKFHHAARYHALLLGLGDRMNGAFGIFMFTHMAITAPIIGIGVFAIVSGGSVSSFLL
CLGWFDGLAMVCFAGQWLQDECIAVGIDLYDADWLHCPEDIKKDMMVVIQRSWTPICLRASSFGIMDYRM
FLGVLKASYSYITLLKQT

>AglaOR69

MWQNLSKFAPQNFYTWMDFFFKILGLQPPKDKKGKLLYLLVAVPHISMSAFLVTGLEWSKLFRDFDKDFKV
SMLTLSVCTLHSVFIFRIIVWFFTREKLAGVARVVQRDSFEFNCFSIYKIKNVIERFRREKMVHTRAICCLIYLSIN
FISLFTINVSYINVLSTETYEGFNERYNTTSTYRVYPYHMYFPFDTSLSHGYYWVGYFYQLYAYCGIIITFLPVDTI

VINSIIHVISQTTLVGEAFNQLGSNIDYSQSLDEVLMLHEIRIVKCITELQNIYRVLYLLEDLYNVQLLIQYGASV
FILCSLCYIIPLVDNVGELICSFIYLSATLGEIFVFTYCSQTLSLKLQNVKNAVYNLDWPCYPPRLRRNLVFLISKL
QKPCLLTAGKIFTLDLLFFIQVTQKSYSFYTLISNTGNNK

>AglaOR70

MNFKALLFPENYFRWLLGFFKLFGLYPPKQHKYKIVYYIFSLIHVTLTMISIILELLNVLLSQDTLQRRMLNLGV
TFMHITAGFRVLKWIWTKNRFEEVISIIQRPSFEFTVYIYNGGSKTTKPNRTPIETFKDARQRRTNFICCGFFWIA
FFFLFANIFVTYIYNLFNATYEKFNPHAGKISVYRDFPYPMWFPFNVSLSDGYYLVAFLYQPYAYFSLMASLWF
VDWSVLSSIIQLQNQLTVVSFALKEIDRNVPRVGAKKISLIKKIRLTKCVEELDAILRATRLMEELCNIDLLVQN
GLAAFFSCTLLHVLSTLTVPIEIFSISTATAVILLESFVQCWICQNLTVEFSKILEKIYELDWADYPPDLRKFLVII
MVRLQNPTVITLGKWKAIDVSFYIHLLKMTYSFYTLISRQANKNK

>AglaOR71

MFKGISKKVLNTSDSIKICVWFPKLLLNSVCMWPGQINDILQGFAFWSIMAVCTFVEYGLYNFIANNFENFNE
SFTAISFFSTTLQVIVKGSVFYYYLNTAKRISDVILYKFWPSNLLGKEVESSLKRYYQILLIGMMSIYFNGSFFSIVF
ILTPLFKETRQTPYKTVYPFEYSDGPKFEIMYLVQSFTNFYVVLGVVIGVDTLFMAVCCNITAQYRLLKNAFLK
LGTDEVKELNSKLSTLCAESDTSERNTTEEKKFLIRCIKHHQTIEDVETVYSVIGFFQLGFSIVSICLSSFVLTTKN
LEFSQLVNITIFLSGNIVQLFCYCSVATEVNFEMDNLSQYIFSSYWYQADFVNFKRDILFVIKKSQEVQKITALK
LFPLNYNTFIQVLRVSFSFYTLLSNITVK

>AglaOR72

MFEGIPKRILNTSDCAKICLWFPKLLLNLVWLWPDQMNDILQGFAFWSVMGICTFTEYGLYSFISNNFKNFN
ESFTAITSFSTLMQVIVKESIFYYYLKTAKRILDVILYKFWPSNLVGREVESSLNRYYHILLIGMISIYFHGSIFSIIYI
LIPLFKETRETPYKTVYPFEYSSSPKFEIMYLVQSFTNFYVVLGVVIGVDTLFMAACYNIIAQFRLLKGVVLKLG
TDEVKEINSKLIILSSESDNIGRNVTEEKEFLIRCIKHHQTTEDIETVYSVIGLFQLGFSIIAICMSSFVVTTEEIEYV
QLVNISIFISGHIVQLFCYCSVANEIGLEMDNLSQHIFSSYWYQTDFVNIKQDILVIMKKSQEVKRITALKLVPL
NYDTFIQVLRISFSFHTLLSNITVK

>AglaOR73

MYFTLLNFSIIILNAMGVNPRKGYTIPQVLLYGTTLSFQMSIIYLGMLLLVYNKSITIIDITYALETVLIMCHGITK
CTLLFVKRNKVRDLLDRIGHFWKIEDIQDEEEKKEHMKYLKFMKTVFLLYTILCIWTTITFTCRPLYINGEITFN
TYRPEWIPFYVLQVYESIAFIVGIYLPLPGLDKFILTLLLLTKLQFKLLNQEIEKVFNEMASSEGKWKHFDKKIK
KIVEYHDFLLDFVNRINDVFSEGLLVYVIVIVLSMCVQMYIVSTQHSLMELIKPLFYLTNALVQYIFCYCLPAQ
ALSDEADRTTEYAYFSRWYENPSSAKVAQLMILGRSQKKTHILAGGIVKIDLETCLKTIRTMVSYAMFIRTMGI
GQDQTR

>AglaOR74

MYFTLFNICIILFNRMGVNPMKGYTIPQVLMYVTTLSLQMSIIYLGMLLLVYKESVTITDVTYVLDSFMLISHGL
TKCTLVFVKRNNIRDLLDRIDKFWKIEDVQDEAERKEHEKYLKFIKTMSFLYNFLCICTTLTFSCKPLSGELSFN
TYRPERIPFHLLQVYESIAFVVGIYLPLTGLDIFVLTLLLLTKMQFKLLNQEMERLFNEMASSDGKSKHFDKKIK
KLVEYHDFLLDFVDRINKGFSEGLLVYVIVLVVGMSVQMYDVSTEPTLIGLIEPIARLITGLTQFTFCYCLPAQA
VTDEADRTTEYAYFNKWYDNPSSAKAAQLMILGRSQRKTHILAGGIVNIDLDNCLKTIKTMVSYAMFIRTMG
IGQD

>AglaOR76

MYLSLFNICLAMLEYVGTNPKKGYSIKQISLFIITFVSQWSIICLVIMYLIYDEKQITILDVTNALESLLLTFHAIV
KLTFAFVKRRELHDLLERTKFFWAVENTEDKQQKNEHLKYLKFLKMAVLMYNALCLTTTLAFTCKPFFVKG
GYLTFTTYKPDWIPFYVMVFYEGLVFIFGLYFAIAGFDIFIVVVLMLLKMQFKLLNQEIRRAFQEIGKPGKNLK
NIDAQIKKLVDYHSFLLDYVNRINSAFSEGMMAYIITIVLSMCVEMYIVSTQNSLTTSFKAVVYMINGLSQYIL

CYCLPAQAMCDEAERTIDYVYFGKWYKHPVSSVKVAQIMMIRRAQKKTLILAGGFMKVDLETSLITVKTMV
SYAMFIRTMGIREN

>AglaOR77

MYLSLLYIPLTMCRFMGVYPEEKYRTGQIFIFVIIFFWQWSIIYLAILHLIYKDHITISDLTTVLETLFLIFQAMMK
LTMFFVKERQFYDLLERINYFWKVDDVQDEVEKKKHLNYLKLIKNRSAVYNIWASATTVAFLLKPLFVKGD
NLIFTTATPTWIPFGVMPFYEEVLFVFGVHGPIVGMDLFTLALFLLVKLQFDMLNQEIQRMFQNMKESEINQR
IKKIVDHHNFILDYVNRINNTLSEGMLLYVVNVLLAMCVEMYIASVQKSIMAAIKAIMYASTGVLQYCICYCL
PAQAVTDEAELTSNYVYFNNWDNHPVSSVKVAQTMMLARAQQQTLILAGGFIKFDLETYLKTLKTMVSYA
MFIRTMGIGQD

>AglaOR78

MYLSRLHIPLTMCRYMGVYPEEKYRTGQIIIFLIIFISQWIIIYLAILHLIYKDHITISDLTNALETLFLIFHAMIKLS
MFFVKESEFHDLLERINYFWKVNDVEDEVERKKHQKYLKLIKIRSSMYNFWASATSVAFMLKPFLLKGDNSIF
TTASPTWIPSGVMTFYEEVFFAFGVYGPIAGMDLFTLALLLLTRMQFDMLNQEIQRVFQNMTNSEENVEETN
ERIKKIVDYHNFLLDYVNRINNALSEGMFLYIVTILLSMCVEMYIASVQKSVMVAIQAIMYASNGLLQYCICY
CLPAQSVTDEAELTAKYVYFNNWNEHPLSSVKVAQIMIIARAQQRTLILAGGFIKLDLETYLKTLKTMISYAM
FLRTMGIGQD

>AglaOR79

MSFYNINFCLKLFNLMGTHPDKRGSVLQVMIYILGAVLNLSIVILSILLLFIKERVTITDVTDSIETIGLLLHGFLK
LTNLFIKRSKISNLLRTMEAHFWKIEDVTDTDARTSHQKFLNSLKNVLKFFTSLCAFLAVLFIIKGFVGQTVFEI
YLPNWFPLYLSVFYQSFTCVLTISFPVISTDLFIFTIFMLTSLQFKLLNEEARNIFGKTGECGIDDEVIKARIKKCV
DHHIFLKNFVKMLNDTFSQVLFIYNGDIVLSLCVEMYIVSTQNSFQTAAKAAVYVFTGMFQYTVCYCIAAQA
ITDEASKISKSVYFGNWYEHPEKYIRNATTLMIAVGQRPVTIIALSFIKVNLETCLKTTQTILSYCMFLRTMGIN

>AglaOR80CTE

MNSYYNVVLYCTGVHPSYSVGNFRVIANLSVTVCGTILVLMNIYKETKLEGVESLSLLLIVLIKYLTLVFGRRLL
KKITYATENFWEVNITDEGIEFFKQHLDRVDKFHKFLSFTGMVAYAAKPLMQNNKSSILGCYVPPAIPYPVFFI
FELYFLGFVSSTFISFNILICSLIISVVIQFRLLNSKIRTINFLGIETKHDMDICFRKLKHRIKYHQFLIRYVQDLNKL
LSTPVAILIVFSVSLVCLNMYNLSVNKSSIVNSYKVMITVSSVIAEFFSVYGFPAQLMMDESAATADTIYYECN
WYLPKVRPLRKDFLMIIMRSQKNVCIKAGNYHVVSSQTILL

>AglaOR81CTE

MISNFCNISLYCTGVHPRHPFGIIRIILNVLVVSCGTVLVLLKIFKENDMQGVESLSIFFLVITKYLTLIFGRRLVQ
QILKATENFWPIDITDDQTIFFKKHLDRVQNFHLFVMYTGMLTFAVKPFVLTDASSMFGCYVPPQIPYPLFYIF
EFYLLSFLSLGFVSFNVMICTLIMSVVTQFRFLNYKIKAINFSDIENDHDLETCFSKLKDRVRYHQYLIGYVQEL
NKLLSTSLALMMIFSVSLMCMDMYILSVHKGTIVDHCKVILTSSAVISEFFSVYGLPAQLMMDESAATADTIYY
ECNWYLPKVRPLQKDFLMVIMRSQKSVSIRAGNYYVVSNQTILM

>AglaOR82CTE

MKDSYFYKHFMFLGIHPDKQFNKCRFIFDVITITCIFILALIKLFQDKNLINVELVSSVFLAAIKYLTLVNWRKM
TRQFEYTIEEFWKIVVADNQITFLLRHLKAVETFYRYVFIFGPFVFMAKPLVTQDSSIFNCYVPPVIPFPLFYILEC
YLVFLGSSAFIALNLFACSLIVLVAVQFRLLNLKIKYLNLEDIENSQDFNRFVRELKCIVNYQQFLMRYVKDLN
KLFCVPVAFLMIISVIILCSNMYILSTNQGTVVDRSRIMVTSSCVIVEYFLVYGLPAQLLMDE

>AglaOR83PSE

MISSYCYKYFLFLGVHPQQPFSKCRVFFNILITTFFFLMACLKCYQDKNLDYVESLNSFVLASIKYLGMVLSRII
QQILQRMEDFWKVNVADDKIMLLMQYLKRIETSFNCLLIFGIFIFALKPFIVKGSSIFNCYTAYDSISFVLYFEFY

YLLLAAISFISFSNFTCALITLVVVQFRLLNLKIGSINLNAENTDGLDEFIRDLKTIVEYQQFLIRYVKDLNKLLSV
PTALLMVSSVTEMCINMYILSLNGDIIDKTRIILVSLCILMEYSLVYGLPAQLLMDELAATADMIYHECEWYLPI
LRPLRNDFLIIIARSQKSVRLRAGNYHVINNQTILLMIKTAYSFYTFLHNIVQRANN

>AglaOR84

MISSYCYKYFLFIGVHPQQPFSKCRFIFNITIIIFFFLMACVKCYQDKNLYFAEILNSFFFVTIKYLGMVLGRNKIQ
QILQSMEHFWKVNVADDKIKYLMQHLKRIERSLSCLHIFGVLIYAGKPFIAKGSSIFNCYVPSIIPFPLFYVFEFY
YLLLASTSYICFSSFTCALIILVVVQFRLLNLRIRSPDLNDVENTDGLEKFIRDLRSIVEYQQFLMRYVEVLNKLL
STPIALLMVSSVTELCINMYILSLNGEIINKSRAIIVSFCTLLDYTMVYGLPAQLLMSESAATADMIYHECKWYL
PILRPLRNDFLIIIARSQKGVRLRAANYHVINNQTILLMVKTAYSFYTFLQNIGQRPRN

>AglaOR85

MENISYFKHFSFLGIHPSQPMDKLRIIFNCSLVSCICLLALLNSYFEKDLGVVDVCNGLIIVTVKYLGLIFGRAPI
KRILEAMRHFWNTDVEDDQVKYYVQYFKTIEKVYKHAIFFGIILHVAKPFFVRGSSICNCYIPPRIPFPIFYAFEI
YVIIVGAASAFCFNVFVCSMIVSVAAQFRLVNLKIEDLNARKIENDHDLRVYKVNLKSIIKYQQFLIRFVDDLN
KLLTVPIALLMVSSVTMICSSLYVRTTHQGSVFEEGRFFIIIFIVTAEFFLIYGLPAQLLMNESEATADTLYSECK
WYSPQLRSLRKNFLIMMIRSQKSICIRAGHYHVINNRTILLMMKTAYTFHTFLQEVT

>AglaOR86

MENNFCFKSFILLGIHPHLSACKWRVTFNNFLTACVIVLAFLKCYLDRDLQIVETISALVVIMVKYVPLALGRK
KIKQILEANELFWKVNVADDQITYLLQHFNGVQKIYKNILILAILIYVIGPFVSNASSIFNCYIPPFIPFPLFYTVE
FYVIINAAFAFVSFDIFICSLIVVVAVQFRLVNLRIVDVNTMEIENDHDVEIYIRKLKSIIKYQQFLMRLVDYLNE
LLSVPIALLMVTSVALKCISMYNMTINQGSISDKGRTIVTILSVIAEFFLAYGLPAQILMDESTATADTIYSECKW
YLPKLRCLRCHFLIMMTRNQRGVCIRAGNYHVINNGTVLLMVKTAYSFYAFLQNVT

>AglaOR87

MENSFCFKILFLLGVHPYYTMNKLRVTFSNLLTACIMSLAIFKCYLDRDLEIVEAISALNIVIVKYLTLAFGRKTI
KRILEAMELFWKVNAVDEQVTYLIRHFKGVEKIYKYIFIFAIVIYAVGPFISKASSIFNCYIPPHVPFPLFYLVEFY
VLLICSFVFTCFNILVCFLIVFVTVQFRLTNLKIKDLNFGEVESDQDVDVYYVRNLRSMIQHQQFLMRCVDDLN
KLLGVPIALLMVTNVSLMCISMYNITMNQGSISDKVRMFVIIFTMVAEFFMVYGLPAQLLMDESAAIADTVYS
ECKWYSSKLQHIWKDFFIVIIRSQRSVCIRAGNYHIINNATVLLMVKSAYTFYAFLQKVT

>AglaOR89

MENNFCLKAFVFLGVDPNRPIRKWRVTFNSALAVWIISLSLCKCYLDRNLQNIEPISALNIIYVKYLALAIGRK
TVKQILESKEQFWEVDIVDDEITYLIQYLKAIDRLYKSSIFLAVSAYTVPPLLSIKSSVFNCFIPEYIPFALFYIVEIY
VIIVGSFVFIYFNIFVCSLIVLAAIQFRLVNFKIRDLSLKEIENDQDVEMYVRNMKKIIKHQQFLMRYVDELNTIL
SVPVALLMVTNIPMICITMYNATLTGSVLLDQFRTLATILCVLTESFLVYGFPAQVMMDQSEATAETIYSECK
WHLPKLRHLRNDFLIMMIRSQEGVSIRAANYHIINNRTVLIIMKTAYTFYTFMQKVA

>AglaOR90

MENNFCLKAFIFLGVDPYRPIRKWRVTFNNLLSVWIISLSLCKCYLDRNLQNIEPISALNIICVKYLALALGTKT
VKQILELKEQFWEVDIVDDEITYLIQYLKAIDRLYKSAIFLAVSAYTVPPLLSINSSVFNCFIPFHIPFPLFYIMEIY
VIIFSAFVFIYFNIFVCSLIVFAAIQFRLVNFKIRDINLNDIENDQDVEVYVRNVKKIIKHQQLLMRYVDDLNTIL
SVPVALLMVTNISMICITMYNATVTEWVLLDQIRMFATVLCVVTESFLVYGFPAQVMMDQSEIAAEATYSEC
KWYLPKLRHLRYDFLIMMIRGQEGVSIRAAKYHIINNRTVLLIMKTAYTFYTFMQKVT

>AglaOR91CTE

MENNFCLKAFIFLGVDPNRPIRKWRVIFNNLLSVWIISLSLCKCYLDRNLQNIEPISALTMICVKYLALALGTK
TVKQILELKEQFWEVDIVDDEITYLIQYLKAIDRLYKSAILLAVSAYTVPPLLSMNSSVYNCFIPFHIPFPLFYIIEI

YVTIINAFVFIYFNIFVCSLIVFAAIQFRLVNFKIRDINLKDIENDQDVEVYVRNVKKIIKHQQLLMRYVDDLNT
ILSVPVALLMVTNISMICITMYNATVSEWVLLDQIRMFATVLCVVTESFLVYGFPAQVMMDQSEVAAEATYS
ECKWYLPILRHLRYDFLIMMIRGQEGVSIRAAKYHIINNRTVLL

>AglaOR92NTE

IMFVKYLALALGTKTVKQILEYKQQFWEVDIVDDEITYLIQYLKAVEKFYKIFAIPAASAYIVPPLLSINSSVFNC
FIPHYIPFPLFYIAEVYVIITVGAFLFISFNIFVCPLIVLAVIQFKLVNFKIRDLNLMDIENDQDVEVYVRNMKKII
KHQQLLMSYVDDLNTLLSVPIALLMVTNISMICITMYNSTVGESGLLDQIRIFATILCVLTESFLVYGFPAQVM
MDQSEAAAETIYSECKWYLPQLRHLRNDFLIMMMRSQEGVHIRAANYHIINNRTVLLIMKTAYSFYTFMQK
VA

>AglaOR93

MENNFCLKAFLLLGVDPNRPIRKWRVTFNNSLAAWIMSLSIFKCYLDRNLLSIEYISALNIIFVKYLALAFGTK
TVKQILEFKEQFWEVDIVDKEITYLIRYLKAIERLYKSFVVLAVSAYIVPPLFSTNPSVFNCFIPYYMPFPLLYIAE
VYVTICSAFLFISFNIFVSSVILLAAIQFRLVNFKVRDLNLKDIENDQDVEVYVRNMKKIVKHQQLLMRYVDEL
NTILSVPVALLMVTNISMICITMYNATVTESVLLDQLRTFATILCVLTESFLVYGFPAQVMMNQSEATAETIYS
ECKWCLPKLQHLRNDFLIMMMRSQEGVSIRAANYHIINNRTVLLIMKTAYTFYTFMQKVA

>AglaOR94

MESTFCLKAFPFLGVDPNRPIRIWLVTFNNLLAVWIMSLSLYKCYLDRNLQNIEPINALIIIFIKYLALALGTKTI
KQILESKEQFWEVDIVDDKITYLIKYMKAIERWYKSFVILAVSAYIVPPLLSINSSVFNCFIPFHIPFPLFYIAEVYV
CIYSGLLFISLTIFVCSLIILATVQFRLVNLKIRDLNLKDIENSQDVEVYVRNMKKIIKHQQLLMRYVDDLNAILS
VPFALLMVTNISMICIIMYNTTITESVLSDQIRMFATILCVLTESFLVYGFPAQLMMDQSEATAEAIYSECKWCL
PELGHLRNDFLIMMIRSQEGVSIRAANYHIINNRTVLLIMKTAYTFYTFIQKVA

>AglaOR95CTE

MENNFYLKGFLLLGVDPNRPIRKWLVTFNNLLTAWIMSLSLFKCYLDRNLENIEPISALNIIFVKYFPLALGTK
TVKQILEYKQQFWEVDIVDDEITYLIQYLKAIERLYKSAIFFGVSAYTVPPLLSINSSVFNCFIPNYIPFPLFYIAEI
YVATIGGLLFLSFNIFVCSLIVSATIQFRLVNLKIRDLNLKDIENDQDVEVYVRNMKKIIKHQQLLMRYVDDLN
TILSVPVALLMVTYISIMCISMYNATVSEGNLFVQVRLTVTIVCILTESFFVYGFPAQVMMDQSEATAETVYSEC
KWYLPKLRHLRNDFLIMIMRCQEGVSIRAANYHIINNRTVLL

>AglaOR97

MGNNFYLKAFLLLGVDPNRPIKKWLVTFNNLLAAWIMSLSLFKCYLDRNLENIEPISAINIIFVKYLALAFGTK
TVKQILESKEQFWQIDIVDDEITYLIQYLKAIERLYKSSIFFSVSAYTVPPLLSINSSVFNCFIPHYIPFPLFYIAEVYI
VIFSSFVFVYFNIFVCSLIVFAAIQFRLVNFKIRDLNLKDIENDQDVEVYVRNMKRIIKHQQLLMRFVDDLNTIL
CVPVALLIVTNISIMCIAMYNATVNEGNLFVQVRLIVTIVCILTESFFVYGFPAQVMTDQSEATAETIYSECKW
YLPKLQHLRNDFLIMMMRSQEGVSIRAANYHIINNRTVLLIMKTAYTFYTFMQKVA

>AglaOR98NC

KTAKQILESKEQFWEVDIVDDEITYLIQYMKAIEKWYESSVIVGACIYTVLPLLSIHSSVFNCIIPHYIPFPLFYIVE
IYIIIVSSFVFVYFNIFVCSLIVSATIQFRLVNLKIRDHNLKDIENNRDVEVYVRNMKKIIKHQQLLMRYVDDLN
TVFSAPIALLMMTSITILCINMYTTTVTEWKLSNQVRLFLTILCILTESFFVYGFPAQVMMEQ

>AglaOR99

MENNFCHKHFLFLGAHPFHSLRKWLCIFNVTLIIYFLFMALYKFYRDNDPEALEIVMCMILVLAKYLTLIFRP
KLIRQIVEATENFWELNVMEDPVTFFLEHLKRVENICKYTFVLALLLLGVKPLVVKGSPVFNCYVLPGIWYPF
HYVAEMYTLTVAASAFLSWNVFVCFLIILVVVQFRLLNLKIEALNCEDVENEHDFDRNVRGLKSMIQYQQYL
MRYVKDLNELLYVPLALMMITSVFLMCLNMYTLTTSEGTMVDSGRVMLTSFALVVEFLIVYGLSAQLLMDES

EATADMIYFECEWYLPNLRPLRKYFLIMMTRSQKDVCIRAGNYHMINNRTILVIMRTAYSFYTFLQKVAYVK
E

>AglaOR100

MKNKFCHKNFLFLGAHPFHSLRKWLCIFNIALLIYFLFMALYQFYRDKDLEALETLSCIILVMAKYLTLIFRPK
LIKQIVEATENFWELNIIDERVTVFLKHLKRVENIYKYTFVLVLLIFAAKPLVVRGSSVFNCYVPPEIWYPSYYV
AEMYTLTVATSAFLSWNVFVCFLIILVAVQFRLLNLKIKALNCEDVQNEHYFDRYVRDLKSVIQYQQNLMRY
IKVLNELLHVPVALLMITSVFLMCLNMYALTTSKGSMVDNGRIMISCSTLIVEFFMVYGLPAQLLMDESEATA
DMVYFECKWYLPNLRPLRKHFLNMMTRSQKGVCIRAGNYHIINNRTILIMMKTAYSFYTFLQKVA

>AglaOR101

MYEEKAEIIFPEENEHLLKEELFWKINLLLKFRRFDDGRKYIWFSAIVLKAIIAVSVTIFAYKVKNEPDKLAACV
ATCPAILMAVFKLFYMYFQSEGIKYLFDAINQEFWDLGIAGSLVRKRIMRRFLLINLTLTSQVCLSFVCIYILNV
YPLMEMPKGVKRLPNPVWTPFDTNVSPLYEILYFLLVCSQVLTVFGNGYYDFAYGSATQHLCAQLLLLKEQL
KNITVGIMPHASDLEKFHSGYFQKRVMERLKICVRHHCRLLKYGKNLDKYSSSILLLQLLMSYLAMVINGYIL
TSNKADTLQTVEICSYTMTIFTEFVVFSVQASDLKDQSITVVDAVALSEWYLFKAPIKKALALLTMNAEKSIVI
TVGGMLEADNSLIIEVVLKAFSAITLLKALTIEEG

>AglaOR102

MVIKFPKPGEFILRDDHMASMRKFLKLHDFDGKTKYFWIGVISLEILLLVSRVTVIFQCLDRPRRVVDLSATIPI
TITALLKAIYLYFDKNRVKYLYNTIESEFWDFRICGPEVQKQAITRYKYSSLVLWSVVTNVIICYLLLQIFPMTP
MQSESDRLLPKVVWSPIDLNPSPLHEIAFVIILLNGLLTALTNIIYDYFYVYCAQHLVVQLMILQDLLRNITEDV
MVDLSDVEKFNSEDFQDTVMDRMKICAENHSKLLKYGENLGRFCSLVLVPQLALTYAGLVVSGFILATDGSS
FYQDVMYFSLFLNTLVELSIFAIPSSHMNTQSLLMLDAIYDSKWYLFNSRMKRACTFMMMNGQEGISVKAGG
ITKIDNSLLVDMMQKVFSAITLMTALIDANQTK

>AglaOR103

MVQFPEAGEFHLRDDPMIMLKKILKMHGFDGKKKYFWTVATIIKIVLLFDRIAFVILCLDRPRRVAELTASLP
VAMTGVLKMIYLYVDKNRVNYLYDTITSEFWDYRIYGPKLEKEAMTIFKYTNRILWTYCANSVFCLTLFLIFPV
AEMPSENDRILPNVVWSPVDLNPSPLYEIVFTVLLVNGIMTYLGNVAYDFFYSYCVQHLIVQFVILKELLRNIT
DGIMDDSSDVEKFNSKYFQDTVMERLKICAEHHSRLLKYGKNIESFCSLVLVPQLVLTYAALVVNGFMLSTD
RSDISKTIALFNLSFTTFIQLVQFAIPSSQLNAQSISVLDALYGSKWYLFNARLKKACTFMMMNGQEGIVVKA
GGTTRIDNPLLVDMLQKVFSAVTLLRALIGVDGNTAK

>AglaOR104PSE

DCLKFVRILFIDFYGHKYTKICTFIYLVFHVPIWIEQYYQYFKTFDLLMVAQYGPGSFILLFGNDVCCRHLYQC
YYPQYVQRTCDCSFGIRKNFSGLSTQ*ARNLEELCSMRYITYIATINLTLGLLTYVAVIMKGTRDNNWEDIYQI
TFIKKHFPRYSGILIVTFYASISYLILSMLMPIYAMMYVTAHIFFQSAILCEYIKRIIENYPEDNELFYKESYQKRVF
QKFRFCIMRGMTIKRITNQQIAALQPLMVLFIVLGMLLVVCFVGSWQLNINKYYPAVFLLIITSGLACLIIVEVG
QWIEEKSLEEYYILCALPWYTFDLTNQKILLCVLLNAGIPITLSPFREFPINRALIVKFYKVACSVYAVAVHMEK
KIPLTDGIMK*

>AglaOR105NTE

SLKLLLAFIKLSTNRYVKILSYFLLVFHTSSFIWQIYLLSKIWENSLISQYSPCLLISFTGIFNISMSLSFEKELRKFM
DLVENNFYSLDMAGEETRQRIARESKQIEYFLCINFLLVLTTCITEVHNEGFNQEHFLFTTYFKETFPTQAHVL
NYSFYFLYLILYMYLLNGAYIVVYLSKQSIFQLYILQYCIFYMLGRTSNVCEKCIQFNENYQKKVFKRLKFCIKY
QELLMRNGRVIIKISNKFLGVSVILLYILWVSALANIIWSPVTNYPYFICLAITLAVTGGMAAEIGDNVSEETTN
LHYAFTNLPWYTWNQKNKKMFLFILIHVGKGIHVGPLKMFIVNRTLMIRWVKFLWSISSLIGKIRDT

>AglaOR106

MEKKEVVNDMFENIRFMKDDPFTHLLYLLDFGYKERIFQINIVKCLIVLYIQIFNVNSIVSIIKDTTGTYFKQYM
SNVLYLQIGIINYMFHIVYGSYIVQFPDLTQKNFLSLDAAGRKVHEKLLRECSLIKRGIRLLLISIFISFIIILPIVGD
EENVMFEVKFIKQNFGKWQKLFLGIYFLYVYSFSYSCAVTYLSFFYVMTHLRYQFILLTELLKKISHGYSPDVD
NEFYQAKIKKRLKVCVQIHNRIIKVSSIFLELGRYAIIILYISVSLDCASAVDYIVERISPPSDFRAAGILVMVGTSL
VVFSRYGQILQDTSIEFYDAVCNSDWPYWNIQNRRTLMIIMMNSVKVTKVSSFDIVDLGHLTVLQVAKVVYSI
VLLFVNVKH

>AglaOR107CTE

MDGAHKTIETSRLLYTGNYMKDDPLIYLIGIIHFNGKHYTNYFNTTRILIIIYLQTFNILGIVYIYKDESGAMLRQ
YVSVILSQQFGIVCHILLIYYSRSILRLPNLAKLYFLPLNATSKKLHQRILRQCATIRTLHPYVFIVSIVTYLIFLPFV
SDQEDIEFTAMFINKYTGTCSNFLLVLYYLCIISLGHSLASMCSICLYITIHLKFQFELLTEYLIQISCDFKPSNEN
VFYQDVIRRRLKSFVQSHNNIKAVSPMFLEIAQGAVLMMAVAGALAYTALVYFIASGISPPSNFRFIVSLLGAV
VTTYLLVVSGQDLQDKSIDVYTAACRCQWVYWNARNRKTLLMIIMNSSKVTKLTSFGILDLGNEIVLR

>AglaOR108INT

MSKNKNLKICGLLGETFMKGICGYILSIFYVKRVLRLSDFIKMSFLPLDATGVEVQRKILHQCVLIKKLFPLILISI
MIMVCITSILSDMEDINFAVMFIRKYFVFPNFFLGLYYLCFLSLGYGIISLYIATFYLTVHLKFQYCLLSEYLKQIS
HGFKSNTEDHLYQDVIQKILRSFIQIHNKIKIFSFELQEMGNWTVLTVSVSAVLLYTAFAYPIASGASPKTNFVC
ISNLILSAIVALIMAGTGQSLRDESLNIYSAACQCDWVYWNAQNRKTLQMIIINTSNEVKLSGFGLMDLGHEII
ITSLKVSYSIVLLFINLNN

>AglaOR109INT

MSKTENEKICGLLGETSMKDDPLIFLLNFLRIYMKNQIPYVNIVKSFIVLYLQTFNVIGIVYICRDETITSLRQYIT
FILAPQIGIFGYILTICYLKRVLYLSDYIKMSFLPLDATGAEVQRKLLRQCALIKKLFPLIFIVIMVMMFKTSVLG
DVEEFHFAVVFIRKYFVFPNFFLGLYCLCFLSLGYGITSSCTATFYLLAHLKFQFYLLSEYLKQISHGFKPHIEDH
LYQDVIQKRLRSFIQVHNKIKIFSFGTLDMGKWAVLAVAVSTILFYTAFAYFIASSVNMYSAACQCEWVYWN
ARNRKTLQMIIINTANEVKVSGFGILDLGHEIIIVILKVSYSIVLLFVNVNN

>AglaOR110

MCKLDKSANKHKLFAKIFMKGDPLGFLAYPMHFNMRNHITLINMVKALIVLYLQTFNIIGMVYILKDESGVY
LQRYTSVILAQQIGIISLVIHIYYTKDIMLLRDVIEASFLPLNAVGKELHQNLLRQCSLIRKLYLLVFILAIPILCN
VLPLTAGREDVQFTVMFIKKHFGVWSYLFQGLYYFCLMALGYGLASVCTVCLYLMTHLKFQFRLSSEYLKQIS
SDCKLDYEDHYYQEVIKQKLRSFVQAHNKIKATSVFLEIAKWPLLVLTMASILSYTAFGYFAVSEDIPSKFVRF
GLGTVIAAIFTVILITSGQGLQDEAADLYNAACHCDWNHWNIQNRKTLLMIIINTSIEVKMTSFGILDLGHQI
LLLLLKVSYSMVLLFVNLKK

>AglaOR111PSE

MSAKEENARERNLLGDTFMKDDPLFFLIYPVLFNMRNHIFLINMIKGFIVLYLEVFNVIGIVYIWKDEIETYMQ
RYISVLLAQQIDILVPRVFIKEHFSVWPNFFQGIYYFCLLALGYGIASTSNSCLYLMTHLKFQLHLLSEYLKRISS
GFTPDCEDAIYQEVIKERLRSIVQAHNNINRTAAVFLTVGKWSLIISAMSTTMSYTAFGYIAVSEDIPSKYYRCG
LGVMTAGIISFIIIRSGQQLQDASANLSSAGYQCEWVHWNVQNRKTLLMIITNTSKKVKMTSFGL

>AglaOR112INT

MDDTCKISKTHKCICKMFMKEDPLHFLIKPLYFGMKKHIFVINVIKSLIILYLQIFNAICVVYICSDVTYLRRYIS
GILIQQVGISSYALMIYYARDILHLSDAVGTSFLPFNATGTKIHQELLRQCALTRNFFPLTFILLTAVHCGLLPV
VSDFAHYQFAVTFLKEHLGVWSYFFLGLYYLCVLSAGYSIGCICCVSLYLGLHLKFQFQLLVSYLKQISLDTDA

NIEDGFYQDLICKRLTSFIVAHVKIKRLSFMFLEIGKWSVLVTTVSCSFLYTSLVHFIAMSSNLYSAACECDWA
DWNVQNRRILLMIIINTSKTVKMTSFGLLDLGNVIMLRMLKLTYSLVLLFINVNK

>AglaOR113

MDAKEVRDTFRLLDERPMKDDPLFFLLYPLYFNMRDRILLINTAKCLNILYLQTFNVIGLFYIFKDETGVYWK
KYLSVIFIQQIGISSYLWQIYYARHFLYFSELTGKYFLPFDATGTKVYQRILGQCALTKKLLLAMFTGLIGTISILL
PIIGNEEDIEFTVMFIKTHFNVWSKFFLVLYYLCVVSLGHSIACSTSMILYLAMHLKFQFYLLVEYLTQISCIEPNI
DDVFHQDALHKRLRSFVRAHGKIKTTAAIFLELSKLSVLVSVILLSLLLSGLAYLVMMDVSPSIYFKCILALSISA
TVGYMITKGCQDLRDESTNLYNVACQCKWFLWNVQNRKTLLMIIANSSKEVKVADYANLDLGYEIIIAIWKI
TYSIVLLCLNLNK

>AglaOR115CTE

MDAKEVRKTFRLLDERPMKDDPLFFMLYPLYFNMRDRILLINTAKCLIILYLQTFNVIGLFYIFKDKTGVYWK
KYLSVIFIQQIGISNYLWQIYYARHFLYFSELTGKFFLPFDATGTKVYQRILGQCALTKKLLLAIFTGLIGTISILLP
IIGNEEDIEFTVMFIKTHFNVWSKFFLVLYYLCIASLGYSIACSSSMIIYLTMHLKFQFYLLVEYLTQISCLAPNN
DDVLQQDALHKRLRSFVHAHGKIKTTAAIFLELSKLSVLASVILLALLLSGLAYLVMMDVTPSTYFRSILGLGT
SLSVGSIITKGGQDLRDE

>AglaOR116

MFTFKKIVQLLFGECITNEEDILHPIINLSLLLFRTRIHKMIGFIILILAGLENILYVKYVIRRPPMEEVIKFLPMQI
VTVAGMVAYTYTIIYFNLCKGLIETMMHFLKPISEASEKTIQRIKKETKYIKMFMIIPLVATVVSPLPWFRTYEE
VHFIIMYINQRYAVRYTVLLFQMIYLFCLSYSCFTATSIHFYWLYYVYHIKFQLYILRENVQELMTERDMFYDD
VYQNEIYDKLIKCVQHHQAIKRCHAVFNHTMYWGLLVGQGSAIVIMTISVIFIIARTEPLPDVPVLVLFLITNM
LLWEYCYFGQQYEYEWETLHLTLNNLPWYNWSIKNRKFLQIFIINTQHALSINSFGRIKANHEIITKTYQAVY
TLITFFCTVSTRN

>AglaOR117

MSTLKKIVQLFFGECITFEGDILHPLINLSSLLFRTRIHKTIGFIILILAGLDNILYVKYVISRPPMQEVIKFLPMQIV
IVAAMLAYTYAIIYFGLNNGLIETMKHSLKPISEASEKTIQRIKKETKYIKMFMIIPLVAVVVASSPGFGTYEKIH
FIIMYINQRYERVLLFQITYLFCLSYSCITVTSIHYYLLYYVFHIKFQLYMLRENVQELMTERDMFYDDAYQNEI
YDKLIKCVQHHQAIKRCHAVFNHTMYWGLLLSQGAAIGVITISVIFIIRRTEPLPDVQVLVVFLITNVLLWEYC
YFGQQYENEWEMLHLTLNNLPWYNWSIKNRKFLQIFIINTQHALTINSFGSIKTNHEIITKSYQAIYTLITFFCT
VTKRN

>AglaOR118

MSTLEKIVQLFFGECITFDGDILHPVGNLFSLLFRTAIHKTIGFIILILTGLENILFVKYVISRPPMEEVIKFLPVQIV
TVNAMLAYTYSIIYFGDELIETMKHSLKPIGEASEKTIPRIKKEIKYIKMFMIIPLVAVVASLTGFGTYKKIHFVIM
YINQRYERVLLLQMIYLFCLSYSCVTVTSIHYYWLYYIFHIKFQLYILRENVRELMTERDMFYDDAYQNEIYDK
LIKCVQHHQDMKRCHAVFNHTVYWGLLLGQGAAIGVITISVIFIIRRIEPLTDVQVLVLFLITNVLLWEYCYFG
QQYENEWEMLHLTLNNLPWYNWSIKNRKLLQIVMINTQHALTIKTFGSIKVNNEIITKSYQAVYTLIAFFCTV
TRN

>AglaOR120

MRAFQKVFNWFFGECETFDGDVLHPLFNLVTLLHRNSANKIINLIIMVLSGLANILYVKYANSGPPVQDIIIFL
PIQFLIFGGMVGYMFVFALFNLYQEVIKTMRHSLKPINDGSEKTIQRIKRVSKNLRKIVIIPALTALFTLSGLGGS
DEIHFILVYINHRYGRKLMFRLIYYFCLSQSSFVVMSMHLLWLYHIFHIKFQLYILTDNIQVLMTGTDMFYDAA
YQNDIHVKLKEYIQHHQAIKRCHAGFNQCVYWGLLIGQSTAVAVITISILFIIWTIQFDANVIIALFVANLLLW
EYCYFAQQYENEFDLIYLTLNDLPWYNWSIRNRKILHMIMMNTQHALTMRTIGSHRVNNRIITMTYREVYTL
VTFFWTVTRWT

>AglaOR121NTE

DVLHPLFNLVSLLHRNSANKIINLIIIVLAGVDNILYVKYVNSGPPGQDIIIFLPIQIYIFAGMLGNMYVFAFFNL
YQEVIKTMRHSLKPISNGSEETIRRIKRIAKNLRKTLIIPALAMLIILSGLGGSDEIHFILMHINQRYGRKLMIRLI
YYFCLSRTTFSVMSMHLLWLYHIFHIKFQLYILTDNIQVLMTGIDMFYDAAYQNDIHVKLKEYIQHHQAIKRC
HTGFNKCLYWGLLTGQLAAVAVITISIIFIIWTIQFDVNVMALLLVSNLLLWEYCYFAQQYENECDLLYLTLN
DLPWYNWDIRNQKILHMILMNTQHALAIKSIGSHSANNQIITMTYREVYAFVTFFCTVTRRT

>AglaOR122PSE

MSTLEKIAQLFFGECITFEGDVLHPLFNLVTLLHRNGANKIINLIIMVLAGLDTILYVKYVNSGPPGHDIIIFLPI
QMYIFGGMLGNMYVFAFFNLYQEVIKTMRHSLKPISNANEKTIRRIKRIAKNLRKTLIIPALAMASESSTIQFD
VNVMALLLVSNLLLWEYCYFAQQYENECDLLYLTLDDLPWYNWSFRNQKILHMILMNTQHALAIKSIGSQ
QPDYNNGELIP

>AglaOR123

MINFYRSSYLLNDVYIKLFGPLVTFEGDTISLLMHLSSLLCRNKLQIRLSMISSSIMVFNIVYYIYYSLFSSNARDE
ALKFLTGQITSFHFFVSYCLMYIFATSYLKVRNLYLKNYKPIFTGSDEVTNSIQKESRLAIKFAWVLVAAVVVN
VTLFLPWTGDEEDFIFILNRFKLHFGNDSLGILLQLGIFFTIYHAAYVAITPMFIVIYYMLQIKFQLALLIEKVERL
NLIESKNLSKFVTNDVNHQRKIKRRLVSCIRHHQFIKRFQRDLGERIYWPTFILASSVLLFETSLLIFYLSNVCPS
SNYRILYSFAVTILQHVVFGFVGETVVEESERLYVAIKCCNWYNWDKKNCRLLEIFLNHTQEPMAFTSSGIIGV
SKELIAKSFKVLYSAITFYHSMSNNRSMKQ

>AglaOR124

MWFGPLTTFEGDTIAVLMHFSTLLIRNRLQRFICTLSSMVIGFSLLYFLCYVVFSRPSRMEVMGFMATQLNTLH
FYISYCIILRYKGTYLEIIDAFLKISKPIATGSDELVAIIKKDSKFVVKLVFVFLSAVAVNTSLFLPWTGDEDTVLF
VLNRIHLYFGESSFRSLLVIVEYIFFYHVSFTATIPMIATIYYMLHIKFQLALLIENVERLNLLAHENVPELKIYDV
NFQRKIKRRLVSCIRHHHGIKTFKEKLNDEIIYWPTFILSASAVVVETSLIIFYLSKINPSSNYRMIYTWFLMTLG
HTVFAMAGQTIVDESERLYIALKCSNWYNFDKNNCRLLTIFLIQTQEPMVITSSGIIGVSKELILKSYKVLYSLIT
FYNSMSSKDPKK

>AglaOR125

MTRLFAFSRTLVIKLIDNLLAKLIVRCERLRYIYNEWFGPLATFEGDTIAALMHISTLMGRNKLQRRICMLFSV
FAGVCVLDFLCYIIYARPSRREIIGFLTLQLAALHYCVAYFLVQKYTGMYSEVMESFLKNCKLISTGSEKIAGIIK
KESKVTIRLVFVFLAVLAVNLTLFLPWTGDEDKINFAVYRIEKYLGDSFFGILLQILLYFCCYHGAYVASIPMFTI
SYYMLHIKFQFVLLTEKVEKLNLLGSKNSSDLIIYDVNFQKKVKRRLVSCIRHHQGIKRFKQKLDKIVYWPTF
MLASSVVLFETSLIIFYLSNIYPSSNYRMIFTWSVTTLGHTVYAMVGQTIVDESEKLYTAVKCSNWYKWDKKN
CCLLTMFLMNTQEPMQITSSGIIVVSKELILKSYKVLYSLITFYKSMSTKND

>AglaOR126

MMKFVLSNFPYKKLNITDDPLYFIPSLLDAILRRKLIKIFIATSVLYFQLLNSFSFFFIMTGQIAFQEYIALSLLCQV
EIFGVISLLDFMRKIPTVLKTTVEYSWPVNIAGHKVENKIVYRLNLLNTIFLWLTSVSMVTHILIGLSYIGDESH
NPYVRIAVEYLPYWEGLIVLIYFIYLPCFCFGCTFPVMLVFYLLTQLNNQMELLNNRLLIISRKFKRNEALLHN
ENYQNFIFDSLKSCCQHHCDILRMKKKYLSICRLWVLFLPVTSLYPAVVAYMLLNGTDVASSFEMISVMIVTM
VLLAVASLLGQIITNKSEEIFKIAYECPWIYWNKRNRQTLLFLLTNSLRPMEFKSYTNIKLNLSFLFTIYRCIYALI
TYLY

>AglaOR127

MTRISLINRSHNSMRDDSDPLSFIEKLLNMNLWRSSIFRIITVVVAVTIQSSNIACLVTDKNNIHRYISLVLTTQP
GIIATITTMFFSSKLDQSLKPSTRNFWPIRAAGKDVENKILQTLKIINRCIVLIILVFIGGYFITFLPLVGDELKINA

LEMISVNYFGRWSPLFCFTIYIYCPVFAFGLLYPFLLVSYLTALATIQLKLLEEKIKTMANDCNDEEGNHLLYN
QNYQNDVYQRLIMCIEHHIRLKKFRNTWVKHSRFAMLYIPVASLAPVGAGYFLIHQISPSSNFRMVLVLLIFA
TSVTGLCFIGQHFQDSFAQIFTVACNCPWIYWNEKNKKALLLLLINSVKPVEIASYTTIAVNLRLLTTFQKWIY
SLITLMH

>AglaOR128NTE

MLKTILGKKLLQHRLTRLFIISSVIIHMGYIVIGLSLALEKGNFHRYVPYFLLYLPCICAMVSTIFMSSSLKKIDVT
INMKFKSPIIQKKVSQECRFLYRASIVLYVVACMAFLIGQVPLFGDEYMFIPIQLILQNYSFPKEVGIVSTILSSISS
LAVAYPSVSGLYLTTLIKIQVYLVSDKLISISDGFGNLKGDELLDDKLYHDIVYKRLRICVKDHVKLIKFYRKAV
EYNQIGITVAPVSSLTPVAILYFILNDISARNNFRMVPIMILFLYQLVIYCKLGQDVEDVGNYLYTVVTKTPWV
FWTKKNRQALLIMLMNAKPMEVKAYNLISLNHRLLLLVYKVVYSLITVLI

>AglaOR129NTE

IICALPITMIMSRNINKLKVTTNMEFNDPNIEKIVALNCRIFYRTSVICYVLVFITIVITGVPLFGDEHTFIPVQLIL
QEYSFPRVVGIATTIHSAFSFMGIIHAFICGLYVIVLGRIQVYLIIDKLKSISRGFSNLKEDELMDDKLYQNMVYK
RLIVCIKDHIEFIKFCKKAMEYNAIGIVFIPFATLYPVAVLYFILNDISAKNNFRMVLLLFVFIYVLVSICKIGQEA
ENETNYLRRVITETPWVWWDRRNRQALLIMLMNAEPVEVKAYNLITLNLRLLLTIFKWVYSIITVLV

>AglaOR130NC

DLFQFALTMLGYKMRRHIYIIKTFIISAVIYIQIFTGLYILEIIFFDGLDNALQFLPLIIIHQTFCCINVILCLNYNRM
MRAQEDIIFKYGWCVKFANAEVMNKVEREVKNLKLTIVFLEIVIMSTYFSFVPLIGDEDQFSFCVMYIETHFGP
WTPIFSVLYYLCISILAYGSTGYVIFMLYLSFYGLVQYHQLNVYLKEFAEDLTFRKHADNSQDMIHRLLSNFVD
HQNDLRCATKKIHDFQKDVVFFVYFFSAVLILTTAMYFILW

>AglaOR131NTE

FLEIVTMSTYFSFVPLIGDEDQFNFYVMYIETHFGSGASIFSVVYYLCMSILGYGSTLYLALTMYLTFHLLIQYHQ
LNVYLKEFPDDLTLRKHADNCQDMIHRGLSNFVNHQNALRCAIKKMYEFNKDLIMFIYFFNGVLVLIAALY
FILWNVSPPSNFRMVLVIVTMLMQSIAFTTIGEKLSDETEQIYYTIINCPWVDWNLKNKKSLLILLTNNMVPYE
INALGILTLNYKIILSEFKMVYTVVVALYSSQK

>AglaOR132NTE

DPFQFVLTMLGFKLRRHIYIIKTFIISAIIYVQIFTGLYILEIIFFDGLDNALQFLPMITIHQTTCCFNMILYVNYNRI
MRAQEEIIFKYCWCVKFANTEVMNKVEREVKNLKLIIVFLEIVTMSTYFSFVPLIGDEDRFNFCVMYIETHFGP
GAPIFSVVYYLCMSILGYGNTLYFALIMYLTFHGLIQYHQLNVYLKEFAEDLTLRKHAENSQDMIHRGLSNFV
DHQNDLRCGTKNIYEFHKDLIMLVYFFNGVLLLTAALCFILWNVSPPSNFRMGLVIVTVLMQSIVITAVGERL
SDETEQIYYTIINCPWVDWNLKNKKSLLILLTNNMVPYELNAFGILSLNYKILLSEFKMVYTVVVALYSSQK

>AmOr1

MENTTNYRNIHYKSDAEYTVHVAKTLLTLIGIWPRRNTFIDNVKFYVQIGIVFFLMCFLLLPHVIYTYFDCENL
TKYMKVIAAQIFSLLAIIKFWTIIINREEIRFWLMEMEIQYRDVECEEDRLVMMNTAKIGRFFTIVYLSLSYTGAL
PYHIILPLISERIVKEDNTTQIPLPYLSDYVFFVIEDSPIYEMTFVLQIFISSIILSTNCGTYSLIASITMHCCGLFEVT
NRKIKTLCKWNNRDLHDRVIDIVQSHLKAIEYSARVGESLSIVFLSEMLGCTIIICFLEFGVIMELEDHKTLSTVT
YFVLMTSIFVNVFIISFIGDRLKQESERIRETSYFIPWYDFPTEVAKNIKTIILRASRPSSLSGAKILELSLQAFCDVC
KTSAAYFNFLRAMTV

>AmOr2

MMKFKQQGLIADLMPNINLMKATGHFMFNYYTDSSTKHIHKIYCIVHLVLILMQFGFCGINLMMESEDVDD
LTANTITMLFFTHSVVKLVYFAVRSKLFYRTLGIWNNPNSHPLFAESNARYHQIAVKKMRILLLAVIGTTVLS
AISWTTITFIGDSVKKVIDPVTNETTYVEIPRLMVRSWYPYDPSHGMAHILTLIFQFYWLIFCMADANLLDVLF
CSWLLFACEQIQHLKNIMKPLMEFSATLDTVVPNSGELFKAGSAEQPKEQEPLPPVTPPQGENMLDMDLRGI
YSNRTDFTTTFRPTAGMTFNGGVGPNGLTKKQEMLVRSAIKYWVERHKHIVRLVTAIGDAYGVALLLHMLT
TTITLTLLAYQATKIHAVDTYAASVVGYLLYSLGQVFMLCIFGNRLIEESSSVMEAAYSCHWYDGSEEAKTFV
QIVCQQCQKAMSISGAKFFTVSLDLFASVLGAMVTYFMVLVQLK

>AmOr3

MSVKTARNIRDYHNIHYRSDAEYTVRVAKILLTMVGIWPRRNTFSNNVKFYVQTTIVFFLMCFLLLPHVIYTY
FDCENLTKYMKVIAAQVFSLLAIIKIWTILINRNEIRFCLMEMEVQYRDVECEEDRLVMMNTAKIGRIFTIVYL
FLGYGGALPYHVILPLISERIVKADNSTQIPLPYLSDYVFFVIEDSPTYEITFVVQMFTSFLIMSLNYGIYSLIASIT
MHCCGLFEVTNRRIETILKNRDLRGRIADIIQSHLKAIEYSALVGKSLSIVFLSEMLGCTIIICFLEFGVIVEWEDH
KTFSMVTYFVLVTSMFVNVFILSFIGDRLKQESERIGQTSYFLPWYEFPTEIAKNIRIIILRASRPSSLSGAKMLDL
SLRVFCDVFKTSAAYLNFLRTMTV

>AmOr4

METKHTEKDLKQAFYVQTFLKIIGAWPIAIESSLGSKIQKWFIISFYLFLQICIVAPCILDVFLKEKNGSRRINLF
MLLISTLNQVFKYVITLNRANELRIAIHEIKKDWLTATPEDRFIFVMNSRIGQRIMLIMAFIMYISGLGYRMVLP
LLKGKIVLPNNVTIRLLPCPTYFTFFNELVSPYYEMIFMLQLLARFFIYTVLNSTVGISLMLSLHMCSLLKILTRK
MADLTDGSIISEKIMQQRIVDIIEYQTRIKRFLSNTELITQYFCFYDIGCSTCLICFIGYSIIVEWENHNIASTVIYFS
GLVTCTLMIYIICYIGQLLLDESNNLAQTCITLNWYRFPKKKARYLILMIIMSNYPIKLTAAKVVDVSLTTFTDV
MKAAVGYLNMLREVI

>AmOr5

METKHTEKDLKQAFYAQSFLKIVGVWPIPIGSPLSSKIRNWFITFFSLFLQICIVGPCILVMFLKEKNGKRKINLF
KLLTNTLNQLFKYIITLNRANELAIAMNEIKNDWLTATSEDRWIFTANSKMGQKVMLIVAVTVYSSGLGYRM
LLPILKGKIVLPNNVTIRLLPCPTYFTFFNELVSPYYEMIFMLQLLAGFFSYTVLNGTVGISLMLSLHMCSLLKIL
TRKMANLTDRSITSENIIQEKIVEIVEYQTKIKRFLGNAELITEYFCFYDIGCNMCLMCFIGYSAILEWENHNIA
AIVVHFMLLGTCIFIIYIVCYIGQLLLDESNNLAQQCITLSWYHFPTRKARCLILMIIMSNYPVKLTAAKVVDVS
LTTFTDVMKAAMGYLNMLREVI

>AmOr6

METKEKDLKQAFYAQPFLKIIGAWPILIESSLSSKIQKWFIISFSISLQMCIVVPCILVMFLKEKNGRRKINLFMLL
TNILNQVFKYVITLNRANELRIAIHEIKKDWLTATPEDRFIFVTNSRIGQRIMLIIAVITYSSGLGYRMVLPLLKG
KIVLANNVTIRLLPCPTYFTFFNELVSPYYEMIFMLQILAGVFVYTVLSGTIGISLMLSLHMCSLLKILRRKMIDL
ADGSITSENTMQKRIVDIVEYQTKIKRFLGNTELITQYFCFYEISCNTCLICFIGYCIILEWENSNVVAIVVHFML
LGTCILVTYIVCYIGQLLIDESNNLARTCITLNWYHFPTRKARCLILIIIMSNYPVKLTAAKVVDVSLTTFTDVM
KAAMGYLNMLREVI

>AmOr7

METKEKDLKQAFYAQPFLKIIGAWPIVIESSLSSKIRKWFIISFSISLQMCIVVPCILVMFLKEKNGRRKINLFMLL
TNILNQVFKYVITLNRANELRIAIHEIKKDWLTATPEDRFIFVTNSRIGQRIMLIIAVIMYSSGLGYRMVLPLLK
GKIVLPNNVTIRLLPCPTYFTFFNELVSPYYEIIFMLQILAGFFIYTVLSGTIGISLMLSLHMCSLLKILRRKMIDLA
DGSITSENTMQKRIVDIVEYQTKIKRFLGNTELITQYFCFYEISCNTCLICFIGYCIILEWENRNVIAIVVHFMLLG
TCIFVTYIVCYIGQLLLDESNNLARTCITLNWYHFPTRKARCLILIIIMSNYPVKLTAAKVVDVSLTTFTDVMKA
AMGYLNMLREVT

>AmOr8

MVQIRNAREGINHTFWFAYPLSRMLGYWPLNVPSSAFSKILNSFTIFFSYLLPLIVLIPGLLYVFLKERNGRRKV
KMLMPHINSIAQMTKYTIILRRTKELGKLLDEIKKDWSTATQENRRIFSERASIEHKLTMIVAITIYGGGFLYRAI
LPLSKGRIVLPNNVTIRLLPCPGYFGSLDEQVTPNYEIIFTLQVLGGFVTHTAVCGIKSACLMVCMHMCGLLRI
LTNKLTDLTNDNDERVVQEKIVHIVEYQTRIKEFLNHVDQFVPYVYLIEIFVGVLITCILGYCIIVEWEDSDAM
AIIAYVALQTTCVFGTFSICYVGQLLVDESESVRQACKTLKWYRLPTKKARSLILLIIMSNYPIKVTAGRLVDVS
LVTFTSIIKSAVGYMNILQQVT

>AmOr9

MARIRNAREGINHTLWFAYPLSKMVGCWPLNIPSSTFSKIFNAFIIFISYLLSLIVLVPGLLYLFLKEKNGRRKIK
MLMPLMSTIAQMTKYTILLRRMKEFNKLLDEIKKDWSTATQENRQIFSAKASIEHKLTTVIAITIYGGGIFYRMI
LPLSKGRIVLPNNVTIRLLPCPGYFGSLNVQITPNYEIIFTLQILGGFVIYTALCGVKSSCLMLCMHMCGLLRILT
NKVMELTSDKDEKVVQEKIVYIVQYQTRIKEFYNYVDQFVPYVYFIEMIVGVLITCVLGYCIIVEWEDSDAMAI
IAYVVLQVTCVFGTFSICYAGQLLVDESENVRQACNTLKWYRLPTKKARSLILLIIMSNYPLKVTAGRIVDVSL
VTFTSIIKSAVGYMNILQQIT

>AmOr10

MVQIRNAKEGLRHTFWFAYPFSRMLGHWPLSVSSSAFSKILNSFIIFISYLLQMIVVIPSLLYVILKEKNPKKKIK
LLMPHLNSIVQMIKYTILLRQMKLIDKLLDEIKKDWSIATEENRRIFSRTASVEHKLTSIIAITIYSGGFFYRMILP
FSKNKIVSNNMTIRLLPCPGYFGSLDEQVSPNYEIIFILQVFGGFVIYTAVCSTKSICLMLCMHMCGLLRILTNK
VMELTNDNDERVVQEKIVHIVEYQMKIKEFLKQIDQFVPTIYLFEVFIQVLIMCIIGYCIIMEWKESNGMGLITY
VIVQMTCLIGSFSVCYVGQLLIDESENIRQAFIALKWYQLPVKKSRSLILLIIISNYPIKVTAGKIIDLSLVTFITIIKT
AVSYMNMLQQIT

>AmOr11

MVQIRNAKEGLKHTFWFAYPFSRTLGYWPLVSPSAFTKFFNSFTIFTLYFLELIVLIPGLLYVLQVKNPRTKIKL
LMPHLNSIAQMAKYTIILQRAKEFSKLLDEIKKDWLLATEENRQIFSERASIEHKLTTVIVVTMYGGGFFYRTIL
PLSKGKILLPNNMTVRLLPCPSYFGSLNEQATPNYEIIFTLQVLGGFIIYTVLCGTKSACLMLCLHMCGLLKILT
NKVMDLTNDSDEQVVQEKIVHIVEYQTRIKEFLNQLDQFVPAIYLIEVVIQVLIICIIGYCIIMEWEDSNAMAM
VIYVVFQVTCVIGTFSVCYVGQLLLDESENIRQAYNTLNWYRLPVKKARSLILLILMSHYPIKVTAGRIMDLSL
VTFTSIIKSAVGYMNMLRTVT

>AmOr12

MARIRNAKDGIRHTFWFAYPFSRMLGYWPLSVSSSAFAKISNYFIIFLSYLLTLIFMVPGLLYIFLKVKNGRSRIK
LLMSHINGIVQMAKYTILLRKTKEIAKLLDEIKKDWMTASEENRQIFSTRASIEHKLTMVVVVTMYGGGFFYR
AILPLSKGKIVLSNNVTIRLLPCPGYFGFLDEQVSPNYEIIFTLQVLGGFVIYTAVCGTKSICLMLCLHMCGLLKI
LTNKVMELTNDKDEKVVQEKIAHIVDYQTRIIEFLNDLNQFVPSVYFFEIILEVLIICIIGYCLITEWEDNNTMA
TVIFVIFQITCFIGTFAVCYAGQLLVDESENVRQACSTLNWYRLPVKKARSLILLILMSNYPIKVTAGRIVDVSL
VTFTSIIKNSVGYMNILQQVT

>AmOr13

MGQPYSLKLVYPLLKILGAWPKSSPSSVLSTILKCCLISICYLIQLMVLIPGILYIFLKEANLGGKIKMFVPHMNG
ITQVSKYTILLRQIKEFNIILKEVKRDYSLATDKNMWIFTTRAYIGHKMMIAIAIAMYSSGVGYRMILPFLKGRI
LLPDNTTVRLLPCPGYYMFLNEQVTPNYEIIFTIQVLGGFLNYTTLCGTTGITTMLCLHMCSLLEILINKMNDL
TCQSDECEIIVRKKLADIVEYQMKIIDFLNHVEQLTSYLYFCEILEYVCGACVIGYCLITEWENSNAAALIVYFIL
EFLCIFCTLTICYIGQLLIDESDKVRQISVTLDWYRLPVNEARGLILVIIMSNYPIKVTAGKIVDISLITFTDIVKTS
VGYLNILRTVA

>AmOr14

MSRVGKAENGMRHTVWFAYPLLRILGAWPNRVSSSTLSKIFNWYLIFTCYTLQLIVLVPGFLHVFLKEKNGR
KKMKMMIPQVNGYLQLCKYSLVLRWTNKLRVLLNEMKEDWLNTTEEDQLIFRAKASFGHRVMSMIAIVTY
SAGLGYRTILPLSKGRILLPNNTTKRLLPCPGYFVFFNEQVSPIYEIIFIIQVLGGLLTYTIMCGTIGMCVMFCLHS
SSLLRILLNKIYQLTKQLDVNEVVVHEKIVDIVKYQTKVKGFLKNVEQLTTYLFLLEIMVETSIGCVIGYNVVTE
WEDSNAAAMIIHLMMQVSTISCTFIMCYVGQTLIDEGNNVRRMSITLDWYRFPVKEARNLILVIIMSSYPVKL
TAGKVVDISLATFTDIIKTTVGYLNMLQKVT

>AmOr15

MSRIGNAEDGMRHTIWFAYMLLGKLGAWPNRATSSTSFSRTRNCILIFMCYSVQLIILIPGLLHFFLKEKDSRK
KVKILIPLINGYLQLCRYSLVLRSANKLCHLLNEMKKDWMNISEEDRLIFRRKASIGHRLMSVVAIIMYSAGLG
YRTFIPLSKGRILLPDNTTIRLLPCPGYYIFFNEQITPNYEIVFTLQVIGGLLSYTIMCGTTSMCAMLCLHATSLLR
ILVKKINELTKQPDINESAVHMKITDIVRYQTKIKQFLNDVEHITTYLFLLEIIDETGIGCVIGYCAITEWEDSDA
TAAIIYLLLEASVFGVTFTMCYVGQILIDEGNNVRRMSITIDWYRFPAKEARNLILVIIMSSYPVKLTAGKVVDI
SLSTYTDIIKATVGYLNMLRKVT

>AmOr16

MENISGIAKAEEDLKYATRFVKPILATIGAWPISSSTSFFLKALQRLGHIFTYFLFFLIMIPTLAYVFLKEKNSKVR
LKLMGPIINCSMQFFKYTIIIWRRKEIQEGLHAIRHDWIQATEEERLIFRSKMKIGRRVVLIAAFTMYGGGLCYR
TILPLLKGTVITADNITIRPLPCPSYFIIINEQQSPIYEILFVLQVMAGMAIYAVISGTCGISALLVLHACSMLRILV
NKIKKLVNKSDMSEVTLQRKIMDIVEYQMKIKRFLKNIETVTEYICLIEMIGGTCLMCLVGYCILMELENTNT
MAVVVYITLQISIIFCVFILCYIGQMLVDENYIVSQASSTINWYRLSIKNMRCLILIIAMSNYPMKLKAAKMME
MSLTTFTDVMKMSMGYLNILREVI

>AmOr17

MENISGIAKAEEDLKYATRFVKPIMGMIGAWPISPSTSFLLKVLQRLRHIFTYFLFFLIMIPTLMYVFLKEKNNK
VRLKLMPPIINCSIQCFKYTIILWRRKEIQEGLYAIKHDWIKATEEERLIFRSKAKIGRRVVLVVAFTMYGGGLC
YRMILPLLKGTIVTANNTMIRALPCPSYFFILNEQQSPIYEILFVLQIIAGIAIYAVICGFCGIFALLVLHAWSMLR
ILVNKIKKLVDKSDMSEVVLQRKIMDIVEYQMKIKRFLKNIETITEYICLIEMIGSTCMICLVGYCILMEWENTN
TMAIVIYITIQISIIFCIFILCYIGQLLVDENYIVSQASSTINWYRLSIKNMRCLILIIAMSNYPMKLKAAKMMEMS
LITFTDIMKVSMGYLNILREII

>AmOr18

MNAEKLMIEGKPPNANYKNDLSFNVRLNVWTLRTIGTWPRSPDHSWLETLEHVCLNLFCYELLAFILIPCSIY
IILEIKDFYNQLKLGSALSFFLMAVMKYCVFIIREDDIRKCVELIENDWKNVRYQEDRKIMLENASFSRRLIVIC
GTFMYGGVIFYYIALPLTRAKIVEEGGNLTYRRLVYPFPKVLLDARHSPINEICYTIQLLSGFVAHNITVAACGL
AALLAIHACGQLQILMSWLEKLVDGRKNDNENLDQRLANIVKQHVRIINFIALTEDLLHEISLIEVVGCTLNI
CFLGYYSMMEWDSKQPVSGVTYIILLISVTFNIFIFCYIGQLLAEQTVKVGEKSYMIDWHRMPWKKSLAIPLMI
SMSHSTTKITAGNIIELSISSFGDVIKTSVAYLNMLRTFTT

>AmOr19

MNMEHFIVEKKSYNASYKNDLFFNVQLNVWTLRTIGTWPKSLDRSWLETIEHVCLCFLNYVLLAFILIPGVM
YFLLEMKDFYDQMKLGSALSFFLMAVMKMCVFIIRENDIRKCIECIEDDWKNVKYQEDRKIMLENASFSRRLI
VICGAFMYGGVVFYYIALPFTRAKVVEEGGNLTYRRLVYPFPKALLDARRTPANELLYTIQLLSGFVAHNITV
AACGLAALLAMHACGQLQILMSWLEKLVDGRENDDENLDQRLVNIVEQHVRIINFITLTEDLLREISLVEVV
GCTINICFLGYYSMMEWDTEHLIRGMTYIILLTSVTFNIFIFCYIGELLAEQTVKVGEKFYMIDWYRMPWKKSL
AISLIISISRSTTKITAGNIIELSISSFGAIIKTSFAYLNILRTLTS

>AmOr20

MEKSKDKANQKFYLTDYEYQKNVNLSIQYNRWLLKPMGLWPNSYTSKDYPYWLINIVCYCLISFLFIPCTLYL
FLEIEDFYGKLKQFGPLIFCMMAFVKYYYLIFHKTDIRECVERIKWDWRNITYAKDREIMIMYANFGRKLVMV
CTFFMYSGFAFYYIAIPISVGRVKTDNLTFVPLVFPFSRFIVDTRYSPTNEIVFSIQLMAGALMHGITSAACSLVA
TFAVHACGQMQVLMNWLQHLIDGRLDMDERLDGRIADVIRQHVRVLKFLALTEKTLQQISFTEFLGCTLDI
CLVGYYVIMESKSNDVTSVITYIILLISLTFNIFIFCYIGEIVAEECRKIGEISYMIEWYRLMGNKKLFCILIIAMSNS
SIKLTAGNIVNLSISTFTDVVKTAVTYLNVLQKTT

>AmOr21

MSSVKIDQDYKSNVNLSIKYSRRISKMIGLWPIFDKISTIHKFLRMLYNTICYCLLMFMIVLGWMYIAFEVKNIY
DGLKFVSLMSFCMLSITKYHLINIHKDDVRECVKRIEWDWKNISYSEDREIMLMNANFGKRLIIVTTTVTYSGF
VFFYIAIPMKIGKIPAPDANISFIPTMFPFPKYIADVRYSPINEIVFFFQFMCGFLVHGVTSSACSLAAIFTVHACG
QIQVMMIWLEHLIEGRLDMCYSVDQRIAKIVSQHVRILKFLSLIEKILQQVSYMEFLECTVNVCLLGYCAIIEW
ESNHLTEVVTYVIILITIIFNIFVFCYIGELLADQSRKIGEVTYMIEWYRLSGKKKLCCVLIIAMSNSSMKLTAGN
LIELSMSTFSDVVKTSFAFLNVLRTLT

>AmOr22

MEKSKINSISCIQTNHDYKRNVNLSIQWSRWILKPIGLWPNSSTISTTGKYLYRLINVICYSLISFLSIPCSLYVILE
VEDIYNRIKLFGPLSFCVMAFLKYHLLILHKDNISECIKRIEWDWKNITYSKDIEIMITNANFGRRLVIICTFFMY
SGFAFYYIAVPISVGKILAEDDNITFIPLVFPFSRFIIDTRYSFINEIVFSIQLIAGALMHTITTAACSLAAIFAVHAC
GQMQVLSNWLKHLINGRSDMYNNVDSRIASIVSQHVRILKFLALTEKALQQVSFVEFLGCMLNICLLGYYVI
TEWSSSHLTSAITFFILLISLTFNIFIFCYIGELVAEQCKKIGEISYMVDWYRLEGNKKLCFVLIIAMSNSSIKLTAG
NMVELCLTTFSDIVKTAVAFLNVLRTLTI

>AmOr23

MSSVKINQDIKNNINFSIKYSRLILKMIGLWPIFDKSSTIHKYLQWLYNVICYSLIMFIIISGWIYISLEVENIYDRL
KFVSLMSFCMLSITKYHLINIHKDDVRECVKRIEWDWKNISYSEDREIMLMNANFGKRLIIVTTTVTYSGFVFF
YIAVPMKIGKIPAPDANISFIPTMFPFPKYIADVRYSPINEIVFLAQFICGFLLHGITSSVCSLAAILTVHACGQIQ
VMMVWLKHLIDGRLDMCNSIDQRIATIVNQHVRILKFLSLIEKILQQVSYMEFLECTMNVCLLGYCAIMEWE
SNHLTEVITYLILLITIIFNIFIFCYIGELLANQSRNIGEVTYMIEWYQLFGKKKLCCVLIIAMSNSSTKLTAGNLIE
LSMSTFSDVIKTSFAFLNVLRTLT

>AmOr24

MSYTKTDHDYKRNVNLSIQWSRWILKPIGLWPNSSTISTTGKYLYRLINVICYSLISFLSIPCSLYVILEVEDIYNR
IKLFGPLSFCVMAFLKYHLLILHKDNISECIKRIEWDWKNITYSKDREIMITNANFGRRLVVICTFFMYSGFAFY
YIAVPISVGKIPAEDDNITFIPLVFPFSRFIIDTRYSFTNEIVFCIQLVAGVLLHTITTAACSLAAIFAVHACGQMQ
VLSSWLKHLINGRSDMYNNVDSRIASIVNQHVRILKFLALTEKALQQVSFVEFLGCMLDICLLGYYVIMEWSS
SHLTSAITFFILLISLTFNIFIFCYIGELVAEQCKKVGEISYMVDWYRLEGNKKLCFVLIIAMSNSSIKLTAGNMV
ELCLTTFSDIVKTAVAFLNVLRTLT

>AmOr25

MEKQQYVIAQDDGKKANLSIQWNRWLLTPIGAWPNLRKSRIGKCYSLLISIICYGLIGFMLVSCSMFLMVEIK
KVYNRIKMIGPLSFFLMTFMKYYLLLLHENDIREGIECIEWDWKNMKHQEDRNIMIEYANYGRKLVLICTFF
MYSAFAFYYLVLPFSVGKIEDGNLTFIQLPFPSSSLIADIRYSPYNEIVLSVQILTGVVMHAITSAACSIAAVFAV
HACGQMQVLMNWLDHLVDGRSDMSKAIDDRIANIVIQHDRILKFLALTEKALQQISFVEFLGCTANMCLLG
YYLIVEWNPKEIILSVTYVALIISITFNIFIFCYIGDGVAEQCQKVGEMAYMIEWYRLTGKKKLCCILIIAMSNSS
VKFTAGNMVELSIYTFSDVVKTSVAFLNMFRALT

>AmOr26

MMNQLNEQSVLMPVSYARDYEYSIQVNRWLLKPIGAWPNLTKATRTEKLLVKLLNFICHSLIIFTVMPCIMYI
FYEDESLKTRMKAIGPTSHWLMGELNYCCLLMRAKEIVYCIEHIKYDWKTVRRARDRELMIKNAKLGRFIAC
IAALCMHSGIMSYTVITGFKKITFQIGNDSYSMYRLPCPFYTNLLDVRFSPMNEIVFALQLLSGFISTSVTVGAC
GLAAVLAMHACGQFNVVMIRSDKLVKDNNEKKQDEQTLHKKLGFIVEHHLRTLSLVWYMEKVMNMICLV
ELVGCTMNMCILKYYFLTEKSKTILGIYAIVYASMVFNIFIFCYIAEIVTEQGKKVGEKFYMTEWYQLPHKTAL
GLVLIISRSSMVIKITAGKLIQISIATFAAVFKASFAYLNMIRTIAM

>AmOr27

MMNQTAITEEIKTNSDYSLQLNRWFLKPIGAWPLFSTTTKFEKTVSLILNIICYAIVILCATPSLMQIILAEESFYL
KLKTLGPVSHWFVSTVNYTALLMKSKDIRYCFEHMEADWQTIKRMEDQQTMLKNAKFGRYVAASCAIFMQ
GGILCFCFVTILTTETIQVGNETRVLHVLPCAVYKKLVNVEENSINIFMLCFQFVAAAIANSSTVGIFSLAAVLA
AHAYGQLSVVMVWITEFVNQSRNQKKTDDFKEIGIIVERHLRVLNFITYLENIMNRIYFLELFRCTMIICIVGYY
ILTEWAEKNVQNLTTYFMMLLSICFNIFIICYIGEILTEQCMKIGEVVYMTDWYYLPDKTILNLILIILRSTVVVQI
TAGKLFNMSIYTFGDVLKTAFAYLNLLRQMT

>AmOr28PSE

MSNRSVAIKTDPDTNSDYCLQLNRWFLKPIGAWPSFPSTTKHERIISFLLNVSCYSSLLFTLIPCLLHMLLEDESF
YLKMKVLGSLAHWFVGTMNYTTLLLRGKEIRLCVEHIRTDWQTVTREEDQQVMLKNAKFGRYVAAVSAAI
LQSGVNCXCCMTISRTELIQIGNETRIVHVLPCAVYRKLIDVTHSPNSELIIASQFLSGFIVNSSTAGIFSLAAILG
AHACGQLSVVMTWITEFVNKSKKREKMIFREIGLIVEHHLRTLNFISCIEETINRIIFLEVFRCCLHICCLGYYIL
MEWSDYDKRSMIIYFMLFVSVCFNIFIICYIGEILAEESMKVGEVVYMTDWYYLPDKTILDLTLIIARSSVVVQIT
AGKLIHMSIQTFTDVIKTGFAYLNLLRQVT

>AmOr29

MKNQQVVITQDDYKRKTNLSIQWNRWLLTPIGAWPNLRKSRIGKCYSLLISIICYSLIGFMLVSCSIFLMVEIN
NIYNKLKMVGPLSFFVMTIMKYYFLLFHENDIREGIERIEWDWKNVKHQEDRNIMITYANYGRKLAFICFFF
MLCAFIFYFLIQPFGGGKIVDGNLTFIQLPFPISILIADVRDSPYNEIMLSIQILTGIVMNAIRSAICSVAAVFAIHA
CGQMQVLMNWLNHLVEGRSDMSKKIDDRIANIVIQHDRILKFLALTERALQQISFVEFLGCTANMCLLGYY
LIVEWNPKELIVSFTYIAIIASITFNIFIFCYIGELVAEQTEKVGEVAYMIEWYRIRGKKKLCCVLIIAMSNSSIKFT
AGNMVELSIYTFSDVVKTSVAFLNMLRALT

>AmOr30

MEKNRSIIGHDDYERNVNLSIRWNRFLLKSLGTWPNLRESRIGKCYSVLIGIVCYGLISFMLTSSNMFLVVEVK
DTYNRIKMIGPLSFFAMTLIKYYFLTFHEENIRKGIEHIEWDWKNVKHEEDKRIMIEYANYGKKLALISIFFVYS
AFVFYYFVVPISVGKIRDENLTFIPLPFPSSKLIADMRQSPANEILFSVQVLSGVIIHAITATAVSIAAVFAVHAC
GQMQMLMNWLECLVDGRSDMNKIVDKRIAKIVVQHDRILKFLALTERALQQISFVEFLGCTMNMCLLGYY
LIVEWNPKEISLSLTYISLLISFTFNIFIFCYIGDLVAEQCQKVGEMTYMIEWYRLTGKKKLCCVLIIAMSNSSIKF
TAGNMVELSIYTFSDVVKTSVAFLNMLRALT

>AmOr31

MTSKSVISEESFDSLCDYSLQLNRWLLKPIGAWPSSSSSSKLERIVSFFLIVLCYGFILFTVIPSLFHIVLEDENLHM
KLKVFGPLSHWFIGGINYTTLLLQNKEIQYCVEHMQTDWKIVNRAKDQQVMMKYAKIGRYIAALCAIFMQT
GVLTYCVVTAFSTRIIEIGNETRIVHMLPCPVYKELISIDTSPTNEIVLISQFVSGFIVNSIAVGAISIGAVFTAHAC
GQLTIIKRWIREYINRSKDNNKNVVINEIGEIVEYHLRILNFIEGIEDVLNRFCFMELFKSTLDISMLGYYILTEW
ADHDIRNLTTYFMILTSMSFNIFIICYIGDILMEQCRKVGEVLYMTNWYYLPYKDILDLILIISRSNAVIKITAGK
LTNMSIYTFGNVMKTTFTYFNLLRHVT

>AmOr32

MIDKFASIQQTNNNLSNYSIQLNRWFLKPIGAWPPSPSTTKLEKIISIVLIICCYSSICFTVIPCLLHVMLEDESFR
DKLKVLGPLSHWFIGAINYTTLLLRSKEIRYCIEHMQRDWRIVTRTEDQQIMMKHAKIGRYIAVFSAAFMQG
GVLSNCAVTAFSTQTIEIGNVTKTIHMIPCTAYKKLIAVDTSPTNEIVIASQFLSGFIVNSSAVGAVSIAAVFAA
HACGQLSLLMVWIREFVDHSKKIHDKNIGLNKIGKIVRHHLRTLSFVTGIENVMSGICFMELFKCTVNICMLG
YYILTAWSVHDIQNMVVFLVILLSMIFNIFIICYIGDILTEQCKMIGEAVYMTNWYYLPGKDILNLVQIILRSSM
VIKITAGKLVHMSIYTFGNVMKTAFAYLNLLRQMT

>AmOr33

MMTSKSVPIEQDNHSLSNYSVQLNRWFLKSIGTWPLSPSTTKLEKTISFLLIICCYCFICFTVIPCLLHIILGDDSF
REKLKVLGPLSHWFIGGINYTTLLLRKKEIRYCIKHVQRDWRIVTRMEDQQVMIKHAKIGRYISMMCAAFMQ
GGVLSYCAVTAFSTQTIEIGNETRIVHMIPCIVYKKLIATDTSPTNEIVIASQFVSGFIVNSSAVGAVSIAAVFAA
HACGQINLLMAWIRQLVNHSNVNNKNVGLDKISNIVRHHLRILSFITGIENVMSGICFMELFKCTMNICMLG
YYVLTAWIDNDMRNLIVCSVILFSMIFNIFIICYIGDILTEQCKMIGEAVYMTNWYYLPGKDILDLIQIILRSSMV
IKITAGKLVHMSIYTFGNVMKTAFTYLNLLRQLT

>AmOr34

MMIDKFVPIEQDNHSLSNHSVQLNRWLLKSIGAWPSFSSTTKLEKIISFVLIICCYCFICFTVIPCLLHVILEDDSF
HEKLKVLGPLSHWLVGGINYTTLLLRNKEIRYCIEHMQRDWEIVTKTEDQQVMIKHAKIGRYITMFCAAFM
QGGVLSYCAVTAFSTQTIEIGNETRIVHMIPCVVYKKLIASDTSPTNEIVIASQFVSGFIVNSSAVGAVSIAAVFT
AHACGQVSLLMAWIRQFVDHSNIQDKNIVLNDIGEIIRHHLKILSFITGIENVMSGICFMELFKCTVNICMLGY
YILTAWTGHDIQSLIVFSVILFSMIFNIFIICYIGDVLTEQCKMIGEAVYMTNWYYLPGKDILNLIQIILRSSMVIKI
TAGKLVHMSIYTFGNVMKTAFTYLNLLRQMT

>AmOr35

MLVLKDSSSVSYSKDWIYSVQINRWLLKAIGIWPLSLCVTTTEKIHSVILTLISIFLIGFLLVPCTLCTLLDKTGDL
DTKIKMIGPFSFCIMAAIKYYVLLSRGSHIGKCIEDIRVDWFRVSSHNCLEDRKIMMENARIGRSLAIFCAGFM
YSGGFFYTTVMPLCTKRTEIIDNEIVRSQAFPIYRGLLDPRTSPSFEIVQLMQCLAGFVIYSVTVGSCSLAAVFVM
HACGQFQILVTKLRRLIDGLKEDKDMENIVHEQRLGNIVEHHLHILGFISQIEELLNEICFVEFIGCTLNICFLG
YFLLKEWEQSETIGILTYCILLISFIFNIFILCYIGEILSEECKSIGLSAYMIDWHRLPGKKALSLILISAASNSSTKLT
AGKLVELSLSSFCSVLKSSLAYLSLLRTLTT

>AmOr36

MTDDISAIQKKFGSLNEYSIQVNRWLSKTIGVWPLPSSTSKFEKITTRILILFCWTIAVLDTTSGLLHFVLVKEDII
IKLKSLAPISYILGGGLNYAVLLLRKNDIRYCIDRIEADWKVITRMADRQVMLKNAKIGRIISCCIVGFMQLGT
FCFCTILGVFKRTIKIGNDSMEIYVLPSPTYKIPVDTNPGHDIVLGFQYVAAYITSATVISAFSFATVFACHASGQ
LTIMIIWIEEFINRSQKENKNRIDEISVIIEHHMRILSFLERAEHLLSPICFMEMFKNILSICLFSYCILAEWSEHNI
RILSTYILAVINITLNTFLICYIGEVLTERCKEIGNMVYMTNWYRLPKKDILNLIMIITRSSVEYKMTAGKIIDMS
VITFGNIIKTVFGYLNILRQVTML

>AmOr37

MMADDIATVQKEFENLNEYSIQFNKWFSKTIGVWPLPSSTSKFEKIMTRILILFCWIIALFDAISGLLHFVLVKE
DIIIKLKSLAPISYIFGGGLNYAVLLLRKDDILYCIEHMETDWKTITRMTDRQIMLKNAKIGRIISCCILAFMQVS
AVCFCTVLGVFKRTIKIGNESMEIYVLPSPIYKIPVDTNPGHDIVLGFQYLAAYITSATVVSAFSFATVFACHAS
GQLTIMIIWIKEFINRPQKENKNRIDEISVIIEHHMRILSFLERAEHLLSPICFMEMFKNILSICLFSYCILAEWSEH
NIRILGTYIFAVINITLNTFLICYIGEVLTERCKKIGNMVYMTNWYRLPKKDILNLIMIITRSSVEYKITAGKIIDM
SVITFGNIIKTVFGYLNILRQTTML

>AmOr38

MMADDIATVQKEFNNLNEYSIQFNKWFSKTIGVWPLPSSTSKFEKIVTRILIIVCSIITLFVIIPSMLHFILVKEDII
SKLKSLGPISYCFGGGLNYAVLLLRKNDIRYCIDHIETDWKVITRMTDRQVMLKNAKIGRIISCCIVGFLQIGTF
CFCTILGVFKRTIKIGNNSMEIYVLPSPAYKIPVDTNPGHDIVLCFQYLAAYITSATVVSAFSFAIVFACHASGQ
LTIMIIWIEEFINRPQEENKNVHIDKISVIIKHHMRILSFLERAEHLLSPICFMEMFKNILSICLFSYCILAEWSEH
NIRILGTYIITVINITLNTFLICYIGEVLTERCKEIGDMVYMTNWYRLPKKDILNLIMIITRSSVEYKMTAGKIIDM
SVITFGNIIKTVFGYLNILRQTTML

>AmOr39

MMADDIATVQKEFDNLNEYSIQFNKWFAKTIGVWPLPSSTSKLEKIMTRILILFCWITTLFVTISSLLHFTLVKE
DIIIKLKSLAPISYCFGGGLNYAVLLYRKSDILYCIEHMEVDWKAITKTADRQIMFKNAKIGRIISCCIAAFVQIS
AVCFCTVLGVFKRTIKIGNESMEIHVLPSPTYKIPVDTNPGYGIILGLQFLTGYIMSATVVIAFSFATVFACHTIG
QLTIMVTWIEEFINRPQEENKNVHIDKISVIIKHHMRILSFLERAEHLLSPICFMEMFKNILSICMFSYCILAEWS
EHDIRILTTYTFAVMNLIFSTFLICYIGEILTERCKEIGNMVYMTNWYQLHDKDILNLIMIIVRSSVEYKMTAGKI
MDMSVITFGNIIKTVFGYLNILRQTTML

>AmOr40

MADDITAIQKKFGSLNEYSIQLNRWLSKTIGVWPLPSSTTKFEKIMTKILIFLCWIIALFVITSSLLHFTLVKEDIIS
KLKTLGPISYCFGGGLNYAVLLLRKDDIRYCIDHIETDWKAITRTGDRQVMFKNAKIGRIISGCIASFMQVSTIC
FGIVFGVFKQKIKIGNESMEIHVLPFPTYKIPVDTNLEHSIVLGFQFLTGCIMSATVVIAFSLATVFACHAAGQL
TIMVTWIKEFVNRPQEENKNMRVNEISVIIEHHLRILSFLGRTEHLLSPICFMEMFKNVLSICMLSYCILVEWSG
RDIRALSAYTFSVMNIALSTFLICYIGEVLTEKCKEIGNMVYMTNWYRLSDKDILNLIMIITRSSVEYKMTAGKII
DMSVITFGNIIKTIFAYLNILRQMTIL

>AmOr41

MADDIVAIQKKFGSLNEYSIQVNRWLSKTIGVWPFTSTTSKFEKIMTKILIIVCSIIALFVTVPSMLHFILVKEDIIT
KLKMTGPIIYCIGGGLNYAILLFLRDDIRYCIEHIEADWKTITRTGDRQVMFKNAKIGRIISGCIGSFLQFSTISYC
TVFGVFKQTIKIGNESMEIHVLPFPTYKIPVDTNLEHGIVLGFQYLTACIMTATIIIAFSLATVFACHAVGQLTI
MVTWIEEFVNRPQEEKKNMRINEISVIIEHHLRILSFLERTEHLLNPIYFMEMFKNILTTCMLSYCILVEWSGHD
IKVLSAYSFTITNIILSLFLICYISEVLNEKCKEIGNIVYMTNWYRLSDKDILNLIMIIIRSSVEYKMTAGKIIDMSVI
TFSNIIKTIFAYLNILRQVTIL

>AmOr42

MADDIVAIQKKFGSLNEYSIQVNRWLSKTIGVWPFTSTTSKFEKIMTKILIIVCSIIALFVTIPSMLHFILVKEDIIT
KLKMTGPIIYCIGGGLNYAILLFLRDDIRYCIEHIEADWKTITRTGDRQVMFKNAKIGRIISGCIGSFLQFSTISYC
TVFGVFKQTIKIGNESMEIHVLPFPTYKIPVDTNLEHGIVLGFQYLTACIMTATIIIAFSLATVFACHAVGQLTI
MVTWIEEFVNRPQEEKKNMRINEISVIIEHHLRILSFLERTEHLLNPIYFMEMFKNILTTCMLSYCILVEWSGHD
IKVLSAYSFTITNIILSLFLICYISEVLNEKCKEIGNIVYMTNWYRLSDKDILNLIMIIIRSSVEYKMTAGKIIDMSVI
TFSNIIKTIFAYLNILRQVTIL

>AmOr43

MMADDIAAIQKKFGSLNEYSIQLNRWFSKTIGVWPLPSSTSKLEKIMTKILIFLCWIIALFVIISSLLYFALVKEDII
SKLKTLGPISYCFGGGLNYAVLLLRKNDIRYCIDHIETDWKAITRTGDRQVMFKNAKIGRIISGCVAGFLQLSTI
SFCTVFGVFKRRIKIGNESMEIYVLPFPTYKIPVDTNPGHNIVLGFQFLAAYIMSATVVIAFSLATVFACHAIGQ
LTIMITWIEEFVNRPQEENKNMRVNEISVIIEHHLRILSFLGRTEHLLSPICFMEMFKNILSICMLSYCILAEWYG
RDVRVLGAYAFSVTCITLNTFLICYIGEVLSEKCKKISNMIYMTNWYRLSEKDILNLIMIMIRSGMEYKMTAGK
IINMSVVTFGNIIKTILAYLNILRQMTIL

>AmOr44

MADDIVAIQKKFGSLNEYSIQVNRWLSKTIGLWPLTSTTSKFEKIMTKILILLCWIIALFVTTLSLLHFILVKEDII
TKLKMIGPISYCVGGGLNYAVLLFLRDDIRYCIDHIETDWNAITRTQDRQVMLKNAKIGRIISGCIAGFMQLD
SICFCTVLGVFKQTIKVGNESIRVYILPYPTYKVPVDTNPGHSILLLLQFLTTCIMSTTVVIAFSLATVFAYHAVG
QLTIMVTWIEEFVNRPQEEKKNMRIDEISVIIEHHLRILSFLGRIEHLLSPICFMEMFKNILSICMISYCILAEWSG
RDVRALSTYAFCVTCIILNTFLICYIGEILSEKCKKISDMIYMTNWYQLSDKDILNLIMIMIRSGVEYKMTAGKIV
NMSVITFGNIIKTIFTYLNILFQMTML

>AmOr45

MADDIAAIQKKFGSLNEYSIQLNRWLSKTIGVWPLSSSSSKFEKIMTKILIFLCCIIALFVIIPSLLHFTLVKEDIISK
LKTLGPIGYCFGGGLNYAILLLRKNDIRYCIEHMKADWKAITRTDDQQIMLKNAKIGRIISCCFAAFMQFSTVI
FCAVFGVFKRTIKISNESMEIYVLPFPTYKIPVDVNPGHNIVLGFQFLAGYITTGTVIIAFSFATVFACHAVGQLT
IMITWIEEFVNRPQEENKNVRVEEISVIIEHHLRILSFLERTEHLLSPICFMEMFKNILTICMLSYCILAEWSGHDI
RALSAYASAVMNISLGTFLICYVGEILTEKCKEIGNMVYMTNWYRLPKKDILNLIMIITRCSMEYKMSAGKMI
DMSVITFGNIVKTIFAYLNILRQMTIL

>AmOr46

MSDDLVEVEKKFGSLNEYSIQFNRWILKPIGAWPISLCTTRNEKIISKILIIVCWSLSLFTLIPGLLHFILEKEDTYL
KLKTIGPLSHWVIGGFNYAVLLLRKNDILHCIEHIRVDWNIITKKQDQQVMLKYAKIGRYIAAFCTAFLQGG
VLCTCIALGAFKTTIKNGNETIEIYSLLCPAYKLPVQTNPTHDIILGTQLLSAFITSSSAAGAFSLAAVFASHALG
QLNIMVAWINEFVNRPIDLNNVYVNKISIIVEHHLRILSFITHIEHLMNPICFMEMFKCMVGMCMPSYYILAE
WSEHNVQNLAVYVMIIISMTCNIFLICYIGEILTEQCKKIGEIIYMTNWYELSNKDIFNLMMIISRSSISVNMSAG
KLIDMSVLTFGNTVKSVFVYLNMLRQMTMI

>AmOr47

MADDIVAIQKKFGSLNEYSIQLNRWFSKTIGVWPLPSSSSKLEKIITKILIFLYWIIVLFIIITSLLHFILVKEDIVSKL
KSLGPISYCFGGGLNYAVLLLRKNDIRYCIDHIEADWKVITRMGDRQVMLKNAKIGRIISCCIVCFMQIGTLCF
CTILGVFKRTIKIGNDSMEIYVLPFPTYKIPVDTNPGHAIILGLQYLTSFIMSATVVIAFSLATVFACHAIGQLTI
MISWIQEFVNQPQKQNKNIRIDEISVIIEHHLRILSFLERTEHLLSPICFMEMFKNILSICMFSYCILAEWSEHDV
RVLGIYAFAVICITLNTFLICYIGEVLTERCKEIRNMVYMTNWYRLPDKDILNLIMIITRSGVEYKMTAGKIIDIS
VITFGNIIKTVFAYLNILRQMTIL

>AmOr48

MADDIVAVQKKFGSLNEYSIQLNRWLSKMIGVWPLPSFTSKFEKIMTKILIFFYWIILLFIILASSLHFLFVKEDIV
SKLKTLGPISYCFGGGFNYAVLLLRKNDIRYCIEHIETDWKIIKRMEDQQVMLKSAKIGRIISGCIAGFMHIGTF
CFCIVLGVLKRTIKIGNDSMEMYVLPFPTYKIPVDTNPGHGIILSLQYLTSYTSSATVVIAFSLATVFAYHAIGQL
TIMISWIQEFVNQPQKQNKNIRIDEISIIIEHHLRILSFLERTEHLLSPICFMEMFKNILSICMFSYCILAEWSERDI
RVLGIYTFAVMNVILSTFLICYIGEVLTERCKEIGNMVYMTNWYHLPDKDIFNLIMIIVRSGVEYKMTAGKIIDI
SVITFGNIVKTVFVYLNILRQMTIL

>AmOr49

MADDLAKIEKKFGDLSEYSIQFNRWILKPIGAWPASFYKSRIEKIVSKILIVICWISSLFTLIPGVLHFFLEKEDIYV
KLKILGPLTHWLVGGFNYAVLLLRKDDIYYCIKQICADWNIITKKQDQQVMLKNAKIGRYVAVFCTVFLQG
GVFCTCLALGAFKKTIKVDNETVNIYNLPCPAYNMPVDTNPTHDIILGTQLLSAFICSSSTAGAFSFVAICASH
ALGQLNLMVIWINEYVNRPKKLNNNAYINKIGIIVEHHLRILSFIARVEHVMSPICFMEMIKCMVGICMPIYYI
LMEWSEHNIQNLTVYVMIIISMTYNIFLVCYIGEIITEECKKIGDIVYMTNWYELSDKNILNLMMIITRSSMNIN
MTAGKMTNMSVLTFGKIVKSIFAYLNVLRQITMI

>AmOr50

MTNDINVAKQRSDNLSEYSIKLSRWYLKPLGAWPASSSTTKMERIISQILIVICWCIILFTVIPGILYILFVKQDIY
VKLKIFGPLSHWCIDGFNYAILLLRKNDILHCIEHLRADWKLITRTQDQQVMLRNAKMGRYIAAFCAIFMQV
IIFFTCFILGIFKRSIHIDNKTVELYNLPCPAYKIPFDTDPTIHDIMLGTQFLSAFVVSSSASASFTLATIFTCHVLG
QLNIMMIWINEFVDRLQRKENKDNHINKIGVIVEHHLRILSLIARIERITCPIYFMELFKCMMGMCMPSYYFL
AEWSERNIQNLTIYVMVALSMSFNILLVCCIGEILREQCKKVGDMVYMTNWYQLPDKDILNLIMIISRSSVEV
KITAGKIITMSIYTFGNIVKTVFAYLNMLRQITMM

>AmOr51

MRSTNNIDNLPLNDRYESDIQYTFQFCHWILKPLGIYYFIYNQANKFEKILSIILILICFFIIQFVIVPFGYYILFYEK
DMNTKIKFLGPLTFCLSALFKYSYLGIKSSELGHCIKHVEKDWKMLQNEDHRVIMSRYVIMGRNLITLCAAF
MYTGGLSYHTIMPLLSKRKVENFTIRPLTYPGYEAFLNIQKSPTYEIIYCMHCIYVIVVGNITMAAYSLTTIFITH
ACGQIKIQMLRLENLKNEKKVLETGIESHLAVVVKNHVEILRFAKNVETTLRELFLVEVIVSTLLMCLLEYYC
MVEWETSDSAAILTYVILLFSFTFNILIFCYVGELLLGQGSEIATALYEIEWYNLPGRKARDIILLLVISKYPPKLT
AGKIFILSMNTFSVVLKSSVVYLNMLRTITEL

>AmOr52

MFDRSYNNSQLKNIHYENDIHYTLQMCQWLLKPIGVWPFVYDRTSRFEQLISIILMATCFSSLLFIILPSGHHIF
FVEKDMHLKVKLLGPVGFCLSSTIKYCYLGVKGVFFEQCIKHVKNDWKMVQDPSYRIIMLKYATISRKLIIMC
AVFLYTGGMSYHTVMQFLSKEKDNNNTFKPLTYLGYDPFFDTQSSPIYEIVFCMHCFAAMIMYSVTTVAYSL
AAIFVTHICGQIQIQATRLQNLVENKDKKNNCDPFALIVHDHVEILRFSKNVEEALREICLAEIIESTIIMCLLE
YYCMTEWQNNDAIAILTYFTLLISFTFNIFIFCYIGEILSEQCSQIGTISYEINWYKLPAKKAHDLILLISISQYPPK
LTAGKIIDLSFNTFSSVVKTSVIYLNLLRTVTD

>AmOr53

MHDRSHDNINGQLKNSHYKSDIHYTLQMCQWLLKPIGVWPLIYNQTSRFEQLISIILMGTCFSSLLFIILPSGH
HILFVEKNLHMKVKAFGPAGFCLSSTIKYCYLGLKGSSFERCIEHMRKDWMMVQDPNHRTIMLKYATISRKL
ITMCAVFLYTGGMSYHTIMQFLSKGKNKDNYTIRPLPYIGYDPFFDTQSSPTYEIVYCIHCFTAMIMYSISTVAY
SLTTIFVTHICGQIQVQIARLQDLVESKEKRKYKDCDPFALIVHDHVEVLRFSNNIEEALREICFTEIIECTIDMC
MLEYYCIMEWSVGDTITLLTFFTLLISFTFNIFIFCYIGEILTEQCSQIGTVSYEIDWYKLSPKEAYDLILLISISQHP
PKLTAGKIIELSLNTFSTVAKTSVVYLNLLRTVTDW

>AmOr54

MHDRSYHDIESQLKNSYYKSDIHYTLQMCQWLLKPIGVWPLISNQTNKFEQLVSIILMITCFSSLLFIILPSGHH
YFFVEKNLNMKVKALGPVSFCVSSTIKYCYLALKGSSFERCIEHMKRDWMMVQDPNHRTIMLKYATISRRLIT
ICAVFLYSGGMSYHTVMQFLSKGKNNYTIRPLPYIGYDPFFDTQSSPTYEIVYCIHCFTAMIMYSISTVAYSLAAI
FVTHICGQIQIQIARLQKLVECKERKKYESCNLFALIVHDHVEILRFSNNIEEALKEICFTEIIECTLNMCMLEYY
CLIEWSAGDTITFLTFFTLLTSFTFNIFIFCYIGEILTEQCSQIGTVSYEIDWYKLSPKEAYDLILLISISQYPPKLTAG
KIIELSFNTFSSVAKTSVVYLNLLRTVTDW

>AmOr55

MHFSVRNLINKPRNPNYEKDITYVMKHNKWVLISIGIWPTVLKNIGKFLPKIVIGINNLMCFFILIQSALHIILE
QKDTLLRLKFFGLIFFSFMSLMKYWALTIRKPEIEHCIQQVQSDWKQVKMENDRELMLKYGIIGRNLTIYSILF
MYISGIMYISFMQYAMRLQINNDNQTNKVLIFPAYSNSIQKSPIYEITYGIQCICGYVLDSVTSGACGLAALFVT
HACGQIDVVISRLDDLVAGQFYKKNSNPNIQVIKIIKHHIKILKFSAVVEKVLQEVFFLEFISSTFVICLLEYYCIT
DWEQNNIISLTSYALLLISLTFNMFLLCYIGDLLIHKSGNIGVAVFMIDWYHLPAKTIQNLILIMAMSNSPAKL
SVGRIVDLSLSTFGNVLKTTFVYLNFLQTAVMQ

>AmOr56

MYLSIQNPINEPRNPNYEKDIAYVTKYNKWVLTCIGIWPIILKNINKILPKIVIGINNLLCSFILIQSALHIIYEEK
DVLLRLKILGLIFFSFISLMKYWALTIHKPEIKYCIEQVQSDWKQVEMENDRELMLKYGILGRNLTIYSILFMYM
GSITYMSITQYAMGLQFNEHNQTIRVLIYPTYGYNIQKSPIYEIIYGVQFMCGYVVDTITSGACGLAALFVTHA
CGQIDIITSRLDDIVAGQFYSKNLNPDIRLMGIIKHHIRILKFSAVVETILQEVFFLEFIGSTFVICLLEYYCIADWE
QKNIISLTSYVLLLISLTFNMFLLCYIGDLLIQKSSNIGVAVFMIDWFHLPTKTIQNLILIMAMSNTPAKLTVGRI
VDLSLSTFGNVLKTTFVYLNFLQTAVMQ

>AmOr57

MHVSVRDPINELRNPNYEKDIAYVTKHNKWVLASIGIWPTVLKNIGKILPKIVIGFNNLLCFFTLTQSALHIILE
QKDTLLRLKFLGLIFFSFMSMMKYWALMIRKPEIEHCIEQVQLDWKQVEIENDRELMLKYGIIGRNLTIYSILF
MYLSGIIYVSIMQYAMGSQINEHNQTIKMLIYPAYGGYNIQKSPTYEIIYGVQCICEYVFDTIASGACGLAALFV
THACGQIDVIMSRLDDIVAGQYKKNSNANIRLMEIIKHHTRILKFSAVVETVLQEVFFLEFVSSTFVICLLEYYC
ITDWEQKNIISLTSYILLLISMTFNMFLLCYIGDLLIEKSGNVGVAVFMIDWYHLPTKTIQNLILIMAMSNTPAK
LSVGRILDLSLSTFGNVLKTTFVYLNFLQTAVM

>AmOr58

MHLFVRDQTNQPRNLNYEKDIVYVTKHNKWILNSIGIWPTVLKGIDEYLPKIAIALSNLVLSFTVIQCVLHILL
EQKDPILRLKILGLTFFSFISLMKYWVLTMRKPKIKLCIEQIQHDWKQVEFERDRKLMLKYGIIGRNLSMYSIVF
MYSGGIIYHTVMHYKLGSYVDEYNRTIKLLIYPTYSRLYDVQKSPVYELVYILQCICGYMFDAVTVGACGLAA
LFATHICGQIDIVMAKLEDLVDGKFSKENSNPNIRLIEIIEHHIKILRFSAMVETVLQEVCFLEFIGSTFVICLLEY
YCITDWQQNNTIGLTTYSLLLISLVFNIFLLCYIGNLLIEKSSNIGIVCCMIDWYQLPIKTIQGLILMIAMSNSPAK
ISAAGIADLSLSTFGSVLKTSFAYLNFIRTTIM

>AmOr59

MHPITLNESDCKARNLKYKEDIAYVTKHNKWILKSIGIWPSIFKDVSKFLPKIMFGLCNFVLFFAIIPCILYIVIEE
NDTMIRFKLFGLLSFCLVALIKYWTLLYRKSRIKNCVEQIWIDWEQVELYEDREMMLKYGQMGRNLMIICAM
FTYTGGTIFHTILQYKVGTFIDEYNRTIKPVIYPTYNGLFNVQRSPIYEFVYILHCMCGYVMHSVTAGACGLTA
LFATHACGQIDIVIARLNDLIHGKYSKEKINLNARFTKIIEHHLRILRFSATVQEVLQELCFLECIGSTFLICLLEY
YCITDWELNNTISLTTYIILLISLIFNIFILCYIGELLMEKSSNIGLSCFMIDWYYLPSKTIRGLILMIAISSNPTKISA
GGIVDLSLSTFGNVLKTSFAYLNFFRTTIM

>AmOr60

MHLTILNESDYRARNLKYKEDIAYVTKHSKWILKSIGIWPIILKDVAKFLPKIVIGISNFVLLFAIIPCILYIILEEK
NNLIKLKLFGLLMFCSIALMKHWALAYRKPKIKNCIEQIQNDWEQVKLYEDREMMLKYGQVGRNLTIICAV
FMYTGGIIYHTILQYEIGTFIDEYNHTIKPVIYPTYSGLFNVQKSPIYELIYVLHCTCGYVMYSITAGACGLAALF
VTHACGQIDIVIARLNDLVHAKYGKGKFNLNARLIKIVEHHLQILRFSATVQVILQEVCFLEFIGSIFLICLLEY
YCITDWELKNTISLTTYIILLISLTFNIFILCYIGELLMEKSSSIGLSCFMIDWYHLPVKTIQGLILIIAISNSPTKISAG
GIVDLSLFTFANILKTSFVYLNFIRAAIM

>AmOr61

MHLTTLNKNNCKVRNLKYKEDIAYITKHNKWILKSIGIWPSVLKSVSRFLPKIMFGFNNFVLLFSVIPCILYIVY
EEKNIMIKFKLVGLLSFSLIALIKYWTLTYRKPRIKDCIEQIWIDWEQVELHEDRKVMLKYGQIGRNLTIICAVFI
YTGGSIFHTILQYKIGTFIDEHNRTIKPVVYPTYNALFDVQKSPIYELVYLLHSICGYIMYSVTAGSCGLTALFAT
HACGQIDIVIARLNDLIHGKYTKNTFNLNTRLVKIVKHHLRILRFSESIEMALQELCFLECIGSTFLICLLEYYCI
TDWELSNTISLTTYTMLLISLTFNIFILCYIGERLMEKSSSIGLSCFMIDWFQLPTKTIHDLILIIAMSNNPIKISAGS
IVDLSLYTFGGVLKTSLVYLSFLRTTIM

>AmOr62

MGKRKESIDERIRNFMVQKMVLKIIGIWPTNGERSFFGRWIFAVTTQIGIIYILSLEIYRHCLDIDDTMDAFVMD
LSAVISLAKLFILRLNSKHAWVLINSVVEDWSAVHDSRHEYIMTEYLKKGRIVSLMILYLGYASGFSFIVKALPF
GDILPFQMFQNSRNSSMNPDIPLKLNYFLASYCVFGSLPLLHHVCVLLLQGIFIFVNAVAHCGNDGLFFSLTM
HLCGQFEILKTRIAKIEFVDRRKIGPLVKRHCQLAVLVNDLEQTFNMIIFVQLLMSALLICVEGFVFLVCLSTK
DNIGALKSMVLMVTLLIQLYLYAYAGDALESRTEEIAQAAFHSFWYQSRGRTARDLILIICRGNSSYHVTAGK
FVFMNIFTFKEILKSSASYLSVLKVMMDT

>AmOr63

MLKKMKTTSNKDFAYAMTPLKFLAWPVGTWPLQVFNTFSIIRATFSTFLLLLMLTILQVELYLDSSNPEYNLD
ALILINAGILAVTKVICFHVRSLGLVSNFTSAVKDYKELNSEENRVIVRRHAYMGRAACISLIFCSYVGCTLFMI
VPIVAGDKEEVINVTEESAMKYPVPFENTLILINMPENMYFLIFIVEYLMLLLTTTGNLGSDSLFFSIVFHLCGQ
VEILRLEYNKLSNENERTTKHITLLIKRHIYLLKLGDMLNKTISSILIVQLSSSCMLICTTGFEFILALSIGNIVMIV
KTFAVICVLLIQLFAYSYVGEYLKTQTEGLGNSIYFCTWYDMPKNVSHNITFIIMRAQHPVLLTAGKFFVINME
TYMSILRISMSYLSVLRVMVNS

>AmOr64

MKTTSNKDFAYAMTPLKFLSWPLGTWPLQVFNTFSIIRAMFSTFLVLLMLAILQVELYLDRSNAENNLDALV
LINGGILAVAKVMCFHIRPLGLISNFTSAVKDYNELNSEENRVIVRRHAYMGRVACASLIFCSYVGSTLFMTV
PMLAGDEEEVINVTEESAIKYPMPSENTLTLINMPEKMYFVIFIVEYLMLLLTSTGNLGSDSLFFGIAFHLCGQV
EILRLEYNKLSNENERATKDIILLTKRHIYLLKLSDMLNETISSILIVQLFSSCVLICTTGFEFILALNIGNIVMTIKT
FIVMCVLLIQLFAYSYVGEYLKTQTEDLSNSVYFCTWYDMPKNVTQNIIFIIMRAQHPVFLTAGKFFVVNMET
YMSILKTSMSYLSVLRVMVNS

>AmOr65

MKTTSNKDFTYAMTLLKFLSWPVGTWPFQVYDTFSLTRTIFSISLLLLMIIIVQVELYLDRTNAENNLDALLLIN
CGILAVGKVMSFRVRSTGLVFNFTSAVKDYNESNDEENRMIMRRHAYMGRVACTSLISCSYVCSTLFITVPML
AGDEIQVINATEENAIKYPIPSKNALEIINMPDNLYFVVFIVEYMMLLFTSIGNLGSDSVFFGIVFHLCGQVEVL
KREYSKLFNKNEKITEHFILLIKRHIYLLNLSKMLNETISSILIIQLFSSCVLICTTGFQFILALSIGNIVLTIKILIIMC
VLLIQLFAYSYVGEYLKTQTESVGNSVYFCTWYDMPKNVSKDIIFIIMKAQRPVLLRAGKIFVVNMETYISILKT
SMSYLSVLRVMVNS

>AmOr66

MKTTLNKEFAYAMTPLKFLSWPVGTWPFQVYDIFSLTRTIFSISLLLLMIAIVQVELYLDRTDAENNLDALLLI
NCGILAVAKVMCFRIRPVGLVSNFSSAIKDYNELNSEENRVIVRRHAYMGRVACASLIFCSYAGSTLFMTVPM
LAGDEEEVINVTVESAMKYPIPSKNILAIINMPENMYFVVFIIEYIMLLLTSTGNLGSDSLFFGIAFHLCGQVEIL
RLKYNKLSNENERTMKHISLLTRRHIYLLKLSDMLNETISSILVIQLFSSCVLICTTGFEFILALSIGNIVMMIRICI
AMCVLLIQLFAYSYVGEYLKTQTESLGNSVYFCTWYEMPKNVSQNITFIIMRAQHPFLLTAGKFFVVNMETY
MSILKTSMSYLSVLRVMVN

>AmOr67

MKTTSNKDFTYAMIPLKFLSWPVGTWPFQVHEIFSISRTIFSISLLLLMVVILQVELYLDRSNAENNLDALLLIN
CGILAVAKVMCFRIRPIGLVSNFSSAIKDYNELNSEENRVIMRRHAYMSRVACASLISCSFIASTLFMTVPMLT
GDKKDIINVTEKSIIKYPIPSKNALAIINMPENLSFMVFIVEYMMLLFTSTGNLGSDSLFFGIVFHLCGQVEILKL
KYNKLSNTNERTMEHIILLTKRHIYLLNLSKMLNETVSSILVIQLFSSCVLICTTGFQLILTLTFGNVVLTIKILAEI
SILLIQLFAYSYVGEYLKTQTEGIGNSVYFCTWYDMPKNVSKDIIFIIMKSQRPVLLTAGKFFVINMETYMSILKT
SMSYLSVLRVMVNS

>AmOr68

MTILQPIFNILTICGCRMPSSCRTSYKRMLYILYATFVLLLLYSFCISQFLNVIINVRTADELCNSFYMFIASLLSC
CKIVALLMNHKAIKIFRRKLEEEPCKPTNTKEVTIQKSFDKNIGSITIYYTVMVEFTVFCMIVSSLVTDFRNQRL
AYEAWLPFNCSAPNYYYYYIAYVHQIIALIGTSLLNVACDVTICGLFVHMYSQQEILKHRLKESVNVENRLNI
GKIVYFHNYLYGYAFMVQEKFKKIIGIQLLSSTLVVCFILYKLANTSLISTKFLEFVLYLACMMTQIFVYCWYG
NQLKLKSVEVVDTIFELDWISLDNRSKKDLINIMRRAMNPIELTCAYIFTIDLRTFVTILKMSYSTYNFLQRTKV
N

>AmOr69

MQLLRTIYHLLTSCACWRPPFLSPLKNFAYTVYYCYVILLIYGATFCQFVDLLLIVETEDEFCDNFYLTLAIFISC
HKMYSMLVNRENIILVNRMLESEPFQPETEEEMDMRDKCDKQARLNAIYYAILVELSVMSLSFGGLLKAESH
KLPYRMWLPYNYTSLSAHIFIYTQQVVSLIVSAMIHVACDSFIWALLMHICSQIEIFNCRLRKIKHEKNEVTKL
CIHYHNLIYRLATTINEQFKMVIFVQFTVSTLTICVNLYILMGTQITFERIMQLAIYSSCMLTQIYIFCWYGNEVK
LKSLDISNMIFELDWPDLDNTTKRDLLMIMMRASYPIEMTSVHVITMNLDSFVILLKTSYSAYNLLQSNRE

>AmOr70

MQALQWTRFLLSVCGCMPPTSWKSSFKKSLYNIYTCVIWLLILSLVSTQILDIIINVKNKNEFIENFYITLVVFVT
SCKMTIILRYRKNILSLMDDLQHEPFSPMTHEENEIRTKFNKMNERTSICYTILVLVSATWIFVRSFFTDFKKRK
LTFRAWLPYDYSELLPFALSYAHQATTSMFCSCQNISCDTLFAGFLVQIYCQFEILEERLKNVQQDESNYSAK
QCVKHYHQIYKFSRTLNEKFKVILFLQFCAIAFILCFNLYRMTTITMIPKLLEASLYLIRVLVQILYYCWFSNEVK
LKSLEVPGMIFKSDWTSWDDKTKKIFLIIMTRATQPFEFTSGYLVTLNLEFFVALIKASYSVFNLLQRTK

>AmOr71

MRILRWTFLLFALCGCFPPSSWTTRLKRYLYKIYAVFSFVALNSFLLSQILDMVYNVKGTDDFSDNFSVTVVVF
VTCFKLITILTRRENILLLCNTLKQEPLSPINTEEFEIFLKFEKLTDWNTLGYFILLMSSSLCILMGSLLANFKIRKL
AFRTWLPYDYSTASAFLLAFAYQVVVATVCTFACVASDTLYSGLLIHISCQFEILEHRLKNIGSDKNYTMKQC
VRHHNHIYKYGEMVNDAFQSIMFFQFCTSLSMICFNFYRIMQIEMDSRYVGTILYMVCSLMQIFYYCWFSNEV
KLKSLELSDMIFRSNWTSLNNNVQRAILLVMRRSMKPIEFTSIYIVSVNLDSFMTLLKSSYSAFSVLQQSRES

>AmOr72

MHLLRWTFKLFVATGYFLSPKIKSPRKRFLYNVYTVVVTLFLLSFLLTLIMQIVFNVRTADELSENFGITITVFTT
ICKFINLLFRRGIIISLLDLLQKEPFLPMDIEEIKIHTKYNKLIEKVSIFYTLQNVSCLVALIGATLITDFKKKKLTFE
AWIPFNYTASWFLFSLTFIHQCGCAVVTSFGISIFDTLFAGLLLQVCCQLDTLVYRLQNIKEDAIQSLKYCARQ
HELIYRFTELMNKLFSSILCLQFLISAVAICFSVYRVIYTKTDSQFAGAIIFVFSALIQIFYFCWHGDIAKYKSLEIP
DMIFNSNWPNLSNEAKKILLIIMARSLTPVEVVSAHIIPLNLESFKRLIKATYSAYNMLQQTK

>AmOr73

MHKLSLSFALLTYGGYWRPTKWPASSYKYHLYNIYSAFMIFLLYFITFCTCVDSLISKNLKTMSEKFSLCISVFG
VSLKVANLFLQRGKIINIMNSLTKENSIPRDEQEEIIQRRNDNYARKVTIYCEILNESAVFFATVGQYKRFINTR
TLPVSDWIPYDLSSTELYIISLLYQTVGLLICANASVGNETLIAGLMIQAGVQFEIFCHRAQNLPSLVLTVTRNS
NVFAETVNTVFQYMIFLQFTISSVVLCLSIYKFSTVDPLSMNFVWSGFYLCCMLMQVYLYCWFGNEVTLKSN
KVSDAIYEMDWTILPSNVMKDLLLVIARSKKPVKITSGQIFILSTESFMKIMKISYSSFNILKNSTMK

>AmOr74FIX

MQGEGYTDVSLKVSQFLLKSAGIWVIGNDAEERQRKFAVFYTLAALIYGIYVNAVDIYHNLDNLAHCVFLTC
NMMCILLGLFKCFVISFFRIEFSRIVSYAQKHFWRLDYDYDEKILFGECQKFCRLWIIVVSMISQSSLAFYIITPIY
ENIGKNKSERILPFKMWVDLPLSVTPYYEIMFVIQLLAVEQIGIAYVCSDFFLCILNLHALYQFRMMQQELSKI
WSAIEQQTTSVAAYTRGCHVALKKCIRRHQSLIEFCNKLEQVFTFPILSHVVVFSLLMCFDTYEILLADIPTLKR
LIFLCHMVASFIHIIFFTYICHGLMEESGNVGLATYSGWWTTLPMNETGRMLRKDIRIIMMKSMRPCYLSRSGF
FPMSLETSTALVSSTMSYFTLMRESSMKTN

>AmOr75

MRRRGSKDVSIIWTSFLMKIVGLWLATDRNEQRQRDFALIYTVGTLFISICIAFRDIYYSWGNFSNSVFICCNIL
YVAIVLLKISVLYAHREEFFNLIAFTQKNFWRLYDDPQELLIITGCKKLCNFSIVLIIFCAQGTCAGYMVTPLIE
NIGKNESDRALPFNLWIDFPVGLSPYFELLFILQILCVYHVATCYICFDNLLCIVNLHVAGQFRILQHRLKNLG
NAIRDETGLPRYEKCCYERLKDCVVQHQTLIEYCKRLEDIFTVMVLGQVMFLAVVICLVGFQLFLADTSASKK
ASLVLNLGGTFFQLLIFTYSCDNLIRQSVNVGNAVFSGPWVNLPMSKAGILVRKNLIIVIMRSQKICCLTAGKF
FPVSLETSTAVLSTAISYFTLLKQSSLENM

>AmOr76

MKSKEVRDLSITVTAFYMKIAGFWTSTNYVEERRRNVTMSYTLFAILFAATTEARDLYFSWGNFSDSIYVACNI
ITVSLVLIKLLTSFIYNEELLGIIRYAKTNFWHSNYDTCEKSIMNKCQRTCNYLVFVFTFFAQGTVLGFILRPILV
NRGKNESDRILPFNMWLELPLSITPYFEVMFFVQVVFVYHVCVCYHCFDSLLCILNLHTASQFRILQHRFANT
CNEKRGKRDEDEESALSFYEYSKLKAYIRQHQALIEYCKKLEQVFNSIVFGQVLLFSLLMCLDGYLILMEETPF
GRRVTFTFHITGCMCQLLMFTYSCDCLIRDSMDIADAAYNCSWSFLPMDKYGKMIRRDLMFVITRSRTPCCL
TACGFFAVSLETYTKVLSTAISIFTILKRYEKEFKSDSS

>AmOr77CTE

MNGLLNGGDASMTMTAAFMKLVGLWTAKNRREQRARKFALIYTVAAMLFALWIEFTDFYYSFGDFSTCLF
NTCNIIYITMPLLKIFVIVLNKKDFFHLIFYTEKHFYKDNYDEHEQRIFTNCRRQCIIFVCFLTFSTKGTLVCYIVS
PLVENIGKNQSERALPFNMWVNLPLSTSPYYEIIFTIQVLSLYHIGVGYFCFDNLLCVLNLQLAGQFQILQYKM
ANIVDLLKEKNEKRIINTSYFAKKCYEAFKKCIREHQALIAYCEKLEKVFSLIILCQVLTFSLIICLDGYQIIL

>AmOr78

MQSENQLDVSITLSTFFLRNIGLWMSDDPGEQRRMRILLVYTVWILLLGMIINGRDLYFTFLYNGDILYALTN
NVTMVMGLIKIYIILLYKGKFLNLIVHMQQNFWNVNYDYHEKEILDDCRKTCIFFVSSLTTIGICAMLSYLMTP
FAIRSGNNESERMLPFNMWLDMPLSKTPYYEITFLIQAMCVYYIGISNFCFDTVFCIMAVHLAGQFRILQYRFT
KLCDTDNQICKKNLILEEQMQKFHEKFKKYVRRHQALIDYHQKLENVYTTIMLSQVLLFSVLICLFGYQVLL
ATASLARRSIFIFLLMGAMFLLFMFTFSCNGVMEQSDNVAVGTYSALWTVMPMEKFGRMLRKDLIMVIMRS
RRVCCLTANRFFPISLETYTKILSTAVSYFTLLSKHVDNS

>AmOr79

MQAEYRLDISINLSTFLLKNVGVWMSHDPGEQRRMRMLLVCTVWMLLLGIVINTRDLYFTMLYNGDILYVV
TNNITLIISLVKICNIIIYKGKFLNLIVDMQENFWNVDYDYHEKEILDDCKKICIFFISSVTTIGICAIISYLMTPFV
AQSGSNESERMLPFNVWITFPVTRTPYYEIIFFIQAICLYYIGISSFCFDNIFCIMAVHLAGQFRILRYRLTKLCEQ
EIYEKDSTLTKQMHKFYEQFKECVRHHQALIDYHQNLENVYTIITLGQVLVFSVLICLFGYQVFVATASFARRS
IFVFMLNGSMFLLFMVTYSCNGVTEHSDNVAIGAYSALWTIVPMDKFGRMLRKDLIMVIKRSRRVCCLTAN
GFFPVSLETYTKILSTAVSYFTLLNNRIENANGL

>AmOr80

MQTESQLDISINLSTFFLKNVGIWMSDNPNEQRRIKMLFLYTIWNLLFGTVVNSRDLYFTLLYDGDILYVTTN
NITMIMGMVKICIILIYKKKFLNLIVYMQQNFWNVNYDHREKQILDDCRKTCIFFVSCVTIMAICAMICYIMIP
FIAQSGSNESERMLPFNMWINLPISRTPYYQITFLIQATCVYYVGISYFCFDNIFCIMAVHLAGQFRILRYRFTKL
CDMEYGIKENSQSILSKQMHKFYEKFRKCVQHHQALIDFYQNLENVYTMITFGQVLVFSVLICLFGYQVLVA
TISFARRFIFVFMLNGSMFLLFMVTYSCNGVIEHSDNVAVGAYSALWTIMPMDKFGKILRKDLIIVIRRSRRVC
CLTANGFFPVSLETYTKILSTALSYFTLLSNRIENSS

>AmOr81

MQTESQVDISMNLSTFFLKNVGVWISDNPSEQRWRNMLLGYTTWILLSGIIINGRDLYFTLLYNGDILYATTN
NITMIMGLVKICIILMYKKKFLNLIVYMQQNFWNLNYDHCEKRILDDCRKTCTFFVSSVTSMAICAMICYLMI
PFIVQSGKNESERMLPFNMWINLPVSRTPYYEIIFFIQAMCVYYVGISTFCFDNIFCIMAVHLAGQFRILRYRFT
KLCDVEYENSQSILSKQMQKFYEKFKKCVQHHQTLIDFYQNLENVYTTITLEQVLVFSVLICLFGYQVLVATA
SFARRFIFVFLLNGSIFLLFMVTYSCNGVIEHSDNVAIGAYSALWTIMPMDKFGKIFRKDLMIVIRRSRRVCCLT
ANGFFPVSLETYTKILSTALSYFTLLSNRVENA

>AmOr82

MQTESQVDISMNLSTFFLKNVGVWMSDNPNEQRQIKMLLINTTWILLSGIVINGRDLYFTLLYHGDILYSITN
NITMIMALIKISIIIIYKGKFLNLIACMQQNFWKVNYDYREKEILNDCRKTCIFFVSCLTTMVICAMISYLIIPFIA
KGNNESERMLPFNMWINLPLSKTPYYEIMFLIQAMCVYYIGVASFCFDNIFCIMAVHLAGQFRILQYRLTKLY
DVECIEMHKKDSILANRVPKFYEKFRKCVQHHQALIDFYQNLENVYTRIAFGEMLVYSILICLFGYQVLVATA
SFARRSIFVFLLNGSTFLLFMVTYSCNGVIEHSDNVAIGAYSALWTIVPMDKFGRMLRKDLIMVITRSRRVCCL
TANGFFPVSLESYTKILSTALSYFTLLSNRVETANDT

>AmOr83FIX

MQTDNQLDISISLSTFFLKNVGVWMPDNSDEQRRMKMLFLYTIWMLFCGTIISTRDLYFTLLYNGDILYAMT
NTITTIMALIKICIILTYKGKFLNLIVYMQQNFWNVDYDCQEKEILDDCRKTCIFFISSVTTIGMCTVMSYLTTPV
ITQSGSNESERMFPFNIWINLPITQRTPYYQIIFFVQGVSVYYIGISYFCFDNIFCIMAVHLAGQFRILRYRLMTLC
DTEPETREKDSRSTFAKQVYKFYEQFKKCVRYHQALIDYYQNLENVYTIITLGQVLVFSVLICLFGYQVFVAAA
STARRFIFVFLLSGSMFLLFMFTYSCNDVMEHSDNVAIGAYSALWTILPMDKFGRMLRNDLIMVIKRSRRVCY
LTANGFFPVSLETYTKILSTAVSYFTLLNNRVENA

>AmOr84

MRSTRDISIIWTSFLMKIVGLWLAADRDEQRRRDFALIYTVGALFIIVCIGFRDIYFTWGNFSDSVYISCNNLYL
MIVVLKVGVLYAHKMEFFDLVTFTRNNFWRSYPDPEEELILAECKRICTIFVVVISFCAQGTCTGYMITPIIANV
GRNESDRELPFNLWVDLPVGLSPYFEILFTVQILCVYHVGVCYICFDNLLCIVNLHVAGQFRILQHRLRNLNV
AVTGDRESYRANVCHAKLRSCVIRHQTLTKYCKQLENIFTIIVLGQVLFLALVICLVGFQLFLMDTPASRKVSL
TLNFAGTLCQLLMFTYSCDDLIRESVNVGNAVFSGPWAELPMDKVGRVVRKNLIIVVARSHRVCCLTAGKFF
PVSLETSTAVLSTAMSYFTLLR

>AmOr85

MSSNKVGGDLSITVMTFYMKIVGFWIASNYVEERRRNLTISYTFFAIFFAMATEARDLYFSWGNFGDSILIICNL
VTVILVLFKISISLMYRNKLHKIIQYAKTNFWNLKYDLHDEQIIINTCKRYSTFFVCIFTFFSQGTVFSFVIRSLKE
NIGKNETERIHPFNLWLDESWYMTPYFEMVFIIEILSLYHVGVCYLYFDNFMCIINLHIAGQFRILQHRFSNVC
NEMCEKCCYQLSRKSPYLSICKYAKLKIYIRQHQTLIEYCRKLEMVSNFIIFGQVLLFSLLICLDGYLILMEDTSN
MSRLIFTFHLISCMCQLLMFTYSCDCLIRDSTNIANATYNSLWSFMPMDKYGKMLRKDLILVIMRSKSPCYLT
ALGFFPVSLETYISILSTAISYFTLLRNRAEQTIMDA

>AmOr86

MHATPYSDVSIVVSQFLLKLTGVWMTVNDGEKRRRRIAMAYTFVIQVYGLYLNIGDIYHSWDDLSHCIFLTC
NTLCIVLTMFKFSILFIRRTEFKNLILFARKNFWHLDYDRHETILFTKCRKFCTLWTLTVFSFTQASLTFYIITPIC
ANIGKNKSERILPFKMWVDFPLSETPYYEIMFVIQLLTVQQIGIAYTCNDNFLCVLNMHVVCQFRILQHRLTK
LWSIIDERADKFNYASKCYEALKECIRQHQSLIEFCDKLEHVYTLPIFGHVVVFSLLMCFDTYEIFLANVPVSM
RLIFFFHMVGSFIHIIFFTYICGGLIEESSNIGLATYSGWWTVLPMDEAGRMLREDVKVMIMKSMRPCHLSAGG
FFPVSLETSTALMSSTLSYFTLMRESSKDK

>AmOr87

MGAKAVAKVVAHSKLVARHSNKDFALSMTAFLMKIVGLWLAKNEQEQRKRRLTLMYTVIAILFGVWVQF
RDFYYSWPNFGNCAYTACNILCLIMVLLKLFVLFVHRKEFIDLLVYTHENFWHTNYTNNELLLLQNCKRISM
LCITLINVCAQGTIVSYVLTPIVENIGRNHSDRVLPFNMWVDLPTLFLSPYYEILFVLQVLSLYHVGVCYICFDN
LLCLMNLHAATQFRILQHRLSDLGSGWDTRRSFNKIDRETSWSSCMENCYATFKLCVKQHQDRITYCHRLN
DIFTIIVLGHILVFSLLMCLVGFQVLMANSPPTRRLIFVFHITGSLCQLLLFTYSCDSLIQESTNVGSAVYSGPWIC
LPMNRIGRTLRRDLRMVIIRSRKPCCLTASRFFPVSLETCTTVLSTAMSYFTLMRQSFAN

>AmOr88

MNGRNVRNLSITVTAFYMKVAGFWVANNYAEKRRRNVAMFVTIFFAFMGISIEGRDLYFAWGDFEDSIFAG
CNVITIVLVLLKIFVLYINNEELLNVVNYAKTNFWRESNYEPHEKKIIDDYRRLCSFLVCSFTFFAQGTVVCFVI
TPVFVNNGKNESDRIHPFNMWFDRSLSLSPYYEIIYTIQVLSAYEIGICYHCFDNLLFVINLYTAGQFRILRYRFE
NICGKNDDKNYYKVSKSSKYCINEYKSFKTCVQQHQALIEYCKKLEDVFSIIVLAQVLLFSLLICLDGYLVLME
DTSRAKRVIFTFHLMGCMCQLLMFTYSCDCLMHDSMSVANAAYNSLWPCLPMDKYGKSLRKDLTFVIMRS
RSPCCLTACGFFPVSLETYTGILSVAVSWFTSLKKYEKKLLQYACIANQSLLKEEESKIIFYHQNI

>AmOr89

MQREVELDVSVNLAAFFLKNVGLWASNDPGHERRRKVILVYTMWCVTLSSVVIIRDVYFTWFYNGDILYVV
TNALSMMMITVKVCVIVVHKEEFINLIVYMQENFWNDNYHDLREREILENCKRTCAFFVSLVTAIGICAILSY
LATPLIVQTASNNSERMLPFNMWLKLPLSESPYYELMFVYQIMTFYFIGISYFCFDNIFCIMTVHLAGQFQILRH
RFDRLCNAEDRIAEKGAHAREFYDRFKARVPYHQALIDYCEKIENVSPTIILEPVMVFSVIICLFGYRILWANA
PSTRRSIFIFLLIGAMSLLFMFTFSCNCVTEHSENIAIGAYSALWTAMPMDKFGKMLRNDLIMVIKRSRRVCCL
TANGFFPVSLETYTTILSTAVSYFTLLRNNVEKANEA

>AmOr90

MEKELDISVNLSSFFLRSIGLWIGDGSTNERRRKGMLAYTIWCTFFSTIISSRDLYFTWIYNGDILYALTNYMSV
MMILLKICVIVVHKSEFINLILYMQRYFWNVNYDSREKEILNGCKKTCAFFVSTVTFIGICAILSYLTTPFTARIG
NNESERILPFNMWVNLPLSQTPYYELLFLIQIITLYYIGICYFCFDNVFCIMAIHLTGQFRILGYRFAKLCNIEHE
MREKDTVLSKHVHTCYEKFKEYVRYHQALINFYTKLENVYTMIILGQVIVFSALICLFCYQVLLANAPSARRSI
FIFLLIGAMSLLFMFTYSCDGVIEQSDNVAVGAYSALWTIMPMDKFGRMLRNDLIMVIERSRRVCCLTANGFF
PVSLETYTTILSTAVSYFTLLRNNMENDKDD

>AmOr91

MSFLESDVSVSLTSIFMKLVGLWMAADQYEQRLRNISVTYNLVAILFALYLQTTDIYYSWGNFSACLFSVSNTL
SLILPLLKIFILLSNKEDFFRLIVYMQRNFLQGNYDDHERKIVFGCKRKCTFFICFFTFFTMATIVSYIAGPIIGNIG
KNESDRVLPFNMWINLPLSMTPYFEITFTLQVLSLYQIGVSYFCFDNFLCIMNLHLAGQFKVLQYRISTIADRVI
EKEEKKEKLIIDSLYFSNKCYTTFKKYIRQHQALIAYCRKLEVVFNWIVLEQVLMFSLLICLDGYQILMANGDI
KTRLTFSFHILACLCQLLMFSYSCDCIIRESVSVATAAYGGPWTLLPMTISGRMMRKDLIIVIMRASIPCCLSGK
GYFIVSLETYTSVLSTAASYFTLLRNNIESDN

>AmOr92PSE

MNFLENDVAVSLTSIFMKFVGIWMXQYQQRMRNIMVAYNVIAIFFALWIQTMDMYHSWGNIRACLFSTSNT
LSLILPLLKIFILLCHKQDFFRLVLYMKRNFLXNYDDHERKIVIGCNQKCTFFICFFTFLTIATTASYMVIPLIVNI
GKNESDRVLPFNMWVNLPLSMTPYFEISFVLZVLSLYQIAVSYFCFDNFLCIMNFHVAGQFKVLQHRISTIAD
LTIKTEEKKEKLIIDSLHFSNKCYTTFKKYIRQHQTLIAYCRKIEVVFNWIVLEQVLMFSLLICLDGYQILMADE
DIKTRSIFSFHILSCLCQLLMFSYSCDCILRESVSVATAAYEGPWTLPPMTISGRMMRKDLIVVIMRASIPCCLSG
KGYFIVSLETYTSVLSTAASYFTLLRNNIESEIMKHD

>AmOr93PSE

MNFLESDVSXIFMKLVGIWMAGNGZEXITLFTAIIFSGYNLZMYFILGDFSACLFFISIILSSIMLLLKIIILFSHRE
DFFHLILYMKRNFLXNYDDHERKMIGCNZKCTSSSVSSRFSRWRPLLLTSSVRLLVKNIGKNESDRILPFNMW
VNLLLNITSYFEITYTLQXFSLSLYHIGVSYFCFDNFLCIMNLHVAGQFQVLQYRISNIIYXRFNEKKEKLIVDSX
ATKCYAIFKKXQHQALIYLIYCRKLEEVFNLIVLEQVLMFSLLICLDGYQILMADGDVKTRLIFSFHILGFLCQL
LMFSYSCDCIIRWTLXPLLSMTSSRRMIRKDLILVIMRSNVPCYLTGRGFFIVSLEMYXVLSTAAXFTLLKQRTE
ATS

>AmOr94

MTLKYRGDVSFSLATFFLRVVGFWLASSRLEEWFGNATVMYSIITIIFSMWVQMRGLYFSWGDFGVCTFIVCN
SLGLVMDLLKILVVFVHKKKFLGLIAYMQKNFWHLDYDQRENSIIADARQLCVYFVCVFSFFSQSTVFSYMF
MPMISNIGKNESDRILIFNMWLDLPLSMSPYFEIIYVIQALCLYQVGICYLCVDNMFCIMCLHLASQFRILQYRL
ANVSNVEDEEGVEENMNSSNRCYAILKNCIRYHQALIQFSITLEEIFTIITLGQVLIFSTLICFVGYQVLLVNMTL
SWRISFLCFLITNMCQLWMFTYSCDCMTRESVNVASAVYCIPWTRIPMDKFGKMIRKDLQFIVVRSRRACCLT
GCGFFDISLETYTKIMSTAMSYFTILKQRIVEVENT

>AmOr95

MKNDDFSINLSSIFIKLMGIWMANDQSEKYVRNVTILYSIIALLFGLWLQITDMYYSWGDFSECIFSMCNMLSI
AAPLLKLITLVVHREDFFYLILYLQRKFLHGDYNDYERNIVLNCKRKCTFFTCSLTFTTLATVVSYVINPLVANI
GRNESDRVLPFNIWIDLPLTITPYYEITFVLEVISLYHIGVSYFCFDNFLCIMNLHVAGQFQVLQYRISNIIDSIDK
EKKEKLIMDSCYFASKYYAIFKKCIRQHQALIAYCRKLEEVFNLIVLEQVLMFSLLICLDGYLVLMADTSTTTR
LIFGLHITVCLCQLLMFTYSCDCIIRESLSVATAANRGPWPMIPMTTSGRMMKKDLILVIMRSGTPCCLTGRGF
FVVSLETYTNVLSTAASYFTLLKQHSEAHS

>AmOr96

MNLKYRKDLAFTVASFYLRVVGFWLTTSRLEEWFRIGVVGYTILAITFSAWVQIRGLYFNWGDFSACTYIACD
GLGLVMDFFKIFSLFIYEKKFLGLMVYMQKNFWHYNYDEKEDLIVKDTKRITAYFVCILTFSSLSSIFTYMFRPL
LTNIGRNETDRILIFNMYLDLPLSISPYYEIAYTIQIAALNQAGSCYFCFDNIFCILCLNVACQFRILQYRIANVPI
LKMKGNPDANKNSSDECYKAFKNYVQQHQALLDFCETLEEIFTIIVIGQILMFSILFCFLGYQVILADLTPSYRI
SFISYLFAGMCQLWMFTYSCDCITQESAKIASAAYASPWINLPMDKFGKMLRQDLQIVVMRSRRACCLTACG
FFPISLETYTKIMSTTMSYFTLLKQRTVDT

>AmOr97PSE

MKXFQKNGKYKSTVFDISYYKTFKKYLKFLGQYPNQSRWNKEFNTNVMICSLISFLIPGLSRVYISIVEKNLNA
LMEIIPIVFATISCAIKLLNHRINKKNFDKLFDLMSKEWEMKNDRNQTCILDEFTKQGNKFAEIYKNVLLSALL
LFLLLPLFPSFLDIVFPLNETRQQFQIFKMKYFVNEDEYFYPIYFHSVWSSFVIIMITVTIDSLYMLIIHHASGLFA
MCGYQIAKATECNDRHNENELFRQCVMTHNKAYKFFEIMNKSSRNSYFLQISLTIIGISIIAVQIVMYLHKPEE
AFRISLFLIAAQFHLFIITLTGQVIADQSSKLSNNMYCTTWYRMPPNVQKIFHIIQIKSSKPCKLTAGGILELNLE
NFGIALKTCMSYFTIFLSLQD

>AmOr98

MDMFQKTGKEYSNIFDIPYYKVLKKYLQFLGQDPYQECKYRNIITIIMLISMIAIFIPTTFEIYVSIHDKNTDAVM
ECLPNLCASLSSVVKILNVHFNRENFNKLLEFVVKEWDELKLNELHILEEITIQGSKIAHLYRNTLLSFLILFLLV
PMYFPILDMIDALNQTRSRQQLLRVNYMVFNADDYFFYVYLQLAWGAIVIVMIVITVDSLYIIIIHHVCGLFAV
CSYEIQKTVKDLTVFTDIEKCSYKELKNCVIKHKKAIKFYNILNNSSQLSYLLQIGINIMGISTTAFQLAVNLDT
RPQEAIRNAVFCGANQFHLFVLSLPGQILLDHCAELSNTIYCSMWYKLPVKIQKMFNIMLMRSKKSCALTVY
GLYELNMENFGTTFKACISYFTMMLSLK

>AmOr99

MDIFQKIEKQHNEIYDIPYYKMMEKYIRFLGQDPRQKDEFRNIIVFILIISIASIVIPTTLELYISLRNKDVDGVIEC
IPHFIASSISAVKLLNLHFNRQNYNILFHFVTKKWQQLKSTYELNALDETIMQGKKMAQLYRNTLFSFLILFLV
VPLVSPILDIVHPLNQTRSRQQLLRVNYIVFDIDDYFFYVYLQLAWGSIIVVLTIIAADWFYILIIHFNSGLFAVC
GVQVLEATMNSNLISKDAFSENSSYEKFRTCVIMHNEVIEFYNILNENCQYSYLIQVGLNMLGMSTTAVQTVI
NLDRPDVAIRSAVFFGADQFHLFLLSLPGQILLDHCADFANAIYDSTWYGTSLEIQKMLYMMQIRSKKLCAL
TAGGLYDMNIENFGITFKTCMSYFTMIMSFK

>AmOr100PSE

VHIFQIYEEQCLDIFEIPYYKSLKKWLILSGLYPPKNIIIILVAISIISVTLPLMFAIYTSLHAKNIDAMFECLPSLGV
CIVAMFKLQNIYNNSENFKKLFTFVAKQWYQLKLNNEIRILEEIIMQGNKMAQIYKNTLLLSMTIFFFVPLIFPI
LDIVYPLNETRPRQQLYRVNYFIFNHEDYFFYVYFQLVWSSFVCVIVIIIFDWLYILIIHHNSGMFAVCGYQIQKI
FAEKVFSNIHIYEQFKNCLIVHSEAIQFFSILDESSRNTYLFLVGTNIMATSISAVQVVLNLDKLEVAIKSAVFLI
AAQFHLFILSIPGQILLNHYSNLKNNIFMSSWYNMPIEVQKMFYVMQIRCKKPCSLTACGLYEMNMENFGTA
LKTCMSYITMILSLK

>AmOr101

MDIFQKTKKQHNEIYDIPYYKMMEKYIRFLGQDPRQKNEFRNIIVFILVISIASILIPTTLELYISLRNKDMDGVIE
CIPHFIASSISAVKLLNLHFNRQNYNILFHFVIKKWQQLKSIYELNALDETIMQGKRMAKLYRNTLFSFLILFLL
VPLVSPILDIVHPLNQTRSRQQLLRVNYIIFDTDDYFFYIYLQLAWGSIIVVLTIIAADWFYILIIHFNSGLFAVCG
VQVLEATMNSNLVSKDAFSENSSYEKFRTCVIMHNEVIEFYNILNENCQYSYLIQVGLNMLGMSTTAVQTVI
NLDRPDVAIRSAVFFGANQFHLFLLSLPGQILLDHCADFANAIYDTTWYGTSLEIQKMLYMMQIRSKKLCAL
TAGGLYDMNIENFGITFKTCMSYFTMIMSLK

>AmOr102

MNIFQKTRRQCPDIFDIPYYKMVEKYFQLLGQDPRLKNEFRNFIVTVVVISISGNIVPTSIELYTSLCDKNMDAV
IEGLPHFIAATISAVKILNVYFYRENFDKLFQFVTNEWNKLKLNNELHILDKTIIRGNRTAHLYRSALLIALVLF
LLIPLISPMLDVFLPLNETRPRQQLLKVNYLVFNDDDYFFYVYLQLAWGSIIVVVTSVAVDSLLILIIHHCSGLF
TVCGYQVQKVISNAKSFNGTVLNNYTYEQIKNCVIMHDEAIQFYNILNESNRNSYLIQVGLNMLAISATAVQ
AVVNLDRPEEAIRSAVFCGANQFHLFVLSLPGQVLLDHCSEFSNNIYSCIWYRAPVRIQKVLYIMQIRSKKLCT
LSAGGLYEMNIENFGITFKTCMSYFTMIMSLK

>AmOr103

MDIFQKTERQYPEIFDIPYYKMVEKYFQLLGQDPRLKNEFRNFIVTVVVISISGNIVPTSIELYTSLCDKNMDAVI
EGLPHFIAATISAVKILNIYFYRENFDKLFQFVASEWDKLKLNNELHILDNTIIQGNKMAQLYRSALLTALILFL
LIPLLSPILDIVLPLNETRPRQQLLKVNYLFFNDDNYFFYVYLQLAWGSIMVVVTIVAVDSLLILIIHHCSGLFTV
CGYQVQQVTGNAKSLNKIVSNNYTYEQIRNCVITHDEAIQFYNILNESNRNSYLIQVGLNMLAISATAVQAV
VNLDRPEEAIRSAVFCGANQFHLFVLSLPGQVLLDHCSEFSNNIYNCIWYRVPVRIQKVLYVMQIRSKKLCTL
SAGGLYEMNIENFGTTFKTCMSYFTMIMSLK

>AmOr104

MDVFQKTRNKCINIFDIPYYKLLEKYMKFLGQDPRQRDGFRNIIVIVMVASISGILIPTSLELYTSLRDKNMDA
VIECLPHLIAAATSVVKLLNIHFNRENFKKLFEFITKEWEKFELNNQFHVLEEITIKGSKMAQLYRNTLLSFMV
LFLLVPLIFPFLDIVHPLNETRPRQQLFRVNYLIFNHNDYFFYIYLQLAWGSIIVVMIIVTVDSLYMIIIHHSSGMF
AMCGYKVQEATKYQNLFNDRIISENYTYEQLKNCITIHNKALQFYNILNESSRNSYLIQVGLNMMGISVTAV
QTVVNLDRPEEAIRTAVFLGAEQFHLFVISLPGQVLLDHCTELANNIYSSTWYRIPVKIQKVLHMMQIRSKKP
CSLTAGGLYEMNMENFGITFKTCMSYFTMLMSLKK

>AmOr105

MSMLQKSNEQEYNAFDIAYYKTLKLYLTICGINPYQNNSISIIIIIMIISVCMSFLCPTSIQLWEAISNKDFDNIIQ
NIPQVITVIASMIKILNIYSNKMQFKNLFYSLAQDWKLLESKEELIMLDKFTQYGSKLALLYRRTLLTFLVIFLFL
PLCNPILDVILPLNETRSRQNIFNVNYIILDNYEYFYIVYMHLSCSAVIIVIIIISVDSLYISIIYHACGLFAACGYQI
QKLTKVHTIEKNGPNISNIDYEEFKQCVIMHYKCLQLYDVLEKCCRNLYLIQMGLNIMIISVTCVEVVVFLDR
PKEAIRAIIYVIAQQFHLYAISLPGETLLNQSSKLADKIYDSEWYKIPMKVQKVLHIMQIRSNKPCILTAAGLYE
MKIESFGITIKTCMSYFMMFLSLRE

>AmOr106

MDVRLEERYLKINKIYSIIVGMWPNQKRKTIPRIFVELIAILAHLTQGGNMVLFFSLTLAMDQIPFLIAAILLMI
KYNNFIINEQKFKELFVSILNDWQKKKTHEEEMILEKYADKSLFFILIYVVNAYFCTVLFLILPLTPILLDIFIPLN
ESRPRVQMYPAYYYIENEADYYYPILIFSIVSLLTAMCVYIATDTTLVYVVQHACGLLTLAGYRFRNSLNDLYS
MRKDSKMDEKIYRRMCYAIKTHKRALAYLTKIEDFYSMNIFAQVGASILCLTVTLMKIATIKWSMETNQYYG
FVIAQVVHIFFLTAQGQFVIDSHDNVYRDMYEPYWYNVQYKIQAMFVLILRRNLNPPLLTAGGLMQLNLNT
FAQVVKTSVSYFTVLKSV

>AmOr107

MDQQAMEELYLKDNKFFGQLVGVWPDQGKFMKFLMRFIILIVMIIAFIAQISRVAVFYSVDVLSDQIPYIDLG
FALMLKQYNYILNEKKLRELLHNIISDRLVKRSKEEEEIFEIYFKRAMFFCSFYEVSIYSCGFMFLSMPSIPLIMNV
IMPLNESRSRELVYPSYYFVDEQKYYYLITGHMLAVCLGHVFVYIACDINLIHVVHHGCALLTISGYHFKHAM
DNVDLCNEKYSDELMEKTYAKVSQSIDAHKKAVEYVNKIDACHIHYFFILLGMIIVTFTGTFIKLTSMEIGGRF
FTFCTFTIGQLTHLLFLMVMGQFLIDSNEEVFKTIYDARWYYGSSKTQSLYLLVLRKCLNPPKLTGGGLIALNL
DSFVKVLKTSFSYYTVFRSS

>AmOr108

MNKQAIEDQYLRINKFFGQLVGVWPYQERFTKFCIRLTIFAIIILTLTTQIYQVIVFCTLDALSNQLPYLNALFIL
LFKQYNYILNEDKLRDLLNDIIFDRLMVRSKKELEILNMYSRRATTLCIFYEVIVIFSAIMFIMIPTIPPILNIIMPL
NESRDREFIYPTYFFIDEEKYYYPILTYMATVILIVSSVYLACDTNLVQIVHHGCALLAISGYHFKHAVDDMKFS
NGNYIDLLMDETYKKVKQSIKAHKTAVEYVDKIDACHIYYFLLIIGMIVLAFTGTFLKLSTMEIEIRFFTFCGYT
VAQLTHLFFLTIMGQFLINANDEIFNTIYEAHWYNGSSRTQSLYVLVLRKCLNPPTLTGGGLIVLNLDSFVQIL
KLSFSYYTVFRS

>AmOr109

MDERAIEDQYLKINKFFGQLVGVWPYQKKFFKTCIRFITFTIMIFSLATQISRVIVFYSLDVLSDQLPYINAGIVT
LFKQYNYILNEDKLRELLHDIVSDRLIERSKEELEILEMYSRRTTALCALYKVMVYSCAFMFLVIPTIPPILNIVA
PLNVSRSREFIYPTYYFVDEQKYYYPILTHMIAVILVLSSVYLACDTNLVQIVHHGCALLAISGYHFKHAVDDV
KFCDGYYIDASMDETYVKIRQSIKAHKTAVQYVDKIDACHIHYFLLVIGMIVLAFTGTFLKLSTMEVGIRFFTF
CAYTIAQLIHLFFLTIMGQFLINANEETFKTIYEADWYNGSSKMQSLYVLVLRKCLSPPKLTGGGFVALNLDSF
VQILKASFSYYTVFRS

>AmOr110FIX

MDKQTIENQYLKINKFFGQLVGVWPFQERFIKTCMRFIVSVIMLLDLATQISRVIVFYSFDVFSDQIPYLNAAII
CLFKEYNYVLNENKLRELLNDIISDRLIRRSKKELEILELYSRKATTLCILYKVMVYSCAFMFLVIPTIPPILNIVA
PLNVSRSREFIYPTYYFVDEQKYYYPILMHMIAAILVLSSIYLACDTNLVQVVHHGCALLAISGYHFKHAVDD
VKICDENYITLMDETYTRVRHSIKAHRTAVEYVDKIDACHIYYFLLTIGMIVLTFTGTFVKLSTMEMGIRFFTFC
AYIAAQLTHLFFLTIMGQFLINANEEIFRTIYEARWYNGSSKTQSLYVLVLRKCLTFPKLTGGGLIILNLNSFVQI
LKASFSYYTVFRS

>AmOr111

MDKRVIEDQYLKINKFFGQLVGVWPYQQRFIKFCIRFITSVIVVLTLAAQISRVIIFYSIDVLSDQLPYLDVGFVL
LFKQYNYILNEDKLRELLNEIISDRLIKRSKEELEILEIYLKRARVLSTVYEVSIFFCGFMFLLIPSIPPILNIISPLNES
RGRELIYPSYYFVDEEKYYYPILMHMIAVALILTSVYVACDTYLVYIVHHGCALLAISGYRFKHAVDDIKLCGG
DCIDPLTDETYTKVRQSIKAHKMAVEYVDKIDACHIHYFLLIIGMIVLAFTGTFVKLSSMEVNVRFFTFCAFTV
GQLTHLFFLTIMGQFLINANEEIFKTIYEARWYNGSSRTQSLYILVLRKCLSPPKLTGGGLVALNLDSFLQILKA
SFSYYTVFRS

>AmOr112

MDARTVEKNFLKVNKIFGLITGVWPYQNYRSKMAERFISVTVMMSGFVTQFAYLVLNPTMDKIATNLPYSIA
SFGTFVKMGNYFLDETKLTTILNHIFEDWATIKSKEEYEIMYKYSRRGLFITISYFLHIGVTETFMLILPMVPPILD
IIVPLNVSRKRVFLYPAYFWLDDEKYYVLLLGHMIITLLMICFIFCACDTNYVYAVQHACGLLAIAKYRFKNV
CKNLKEDHAIPLEEIKYKSICESIKAHQHALKYLKLIENSYHTYLFVSMGLLIMAISVSLLEVANGKNGSRELV
QATFLFAQLFHTFILTVQGQFVINELQDVYESIYESPWYTFSPRIRSLYVLSLRSCLNFPTLTAGGLIVLNLQSFA
EIIKAAVSYYTVMQTT

>AmOr113

MDSDILEKRFLKITKRFAKLSGIWPDQNKYLKYISWIIIYVISIPSIVVQIARIVHISTANVIVEQSGIATAIFLSLLK
EANYILNATKVKSLFNDMYMDWRMDRPKKEFEIMSTYAQRGSFLAMFYFINAYCCSLLFLQVPWTARLLYM
IKSQNTSPPMLYVIPGYYFVDDDRDYYYFIQLHMSLSIIMVANVYVAYDTCYMVFVQHVCGLLAVAGYRFKH
AIDDSASKNSEEKIKETCKKIRSSIQGHEGAIRYLKKIEDTHVNLLFISLGLIIMCFSITLLKVVTMDYCLDFYKYS
SFLIVQLMHLCYVMIQGQFVIDSCNEIYYSIYEASWYNINPKIQALYILALRRSLTPPRLTAGGLIELNMQSFSEV
IKLSISYYTVLRST

>AmOr114

MKFIGIWPEERKWNQASNYLVLIPFLMILCFICAPQTINLTIISNDFNLVIENLSMGNITITLSLLKTIAFWINGK
PLKSLLNCMANDWIKVTSKTEQETMARIASITRNTIIKSTVMCHTVVAFYVFLRYISMKYNENKLLFRAYFPY
DTTVSPNYELTILGQFVAALYAATSYTAVDTFVAMLILHVCGQLSGIKNELSRLPTYDKKDLKRRLKEIVQKH
EYVNRFAETIENCFNVMLLIQILGCTVQLCFQCFQAIMSFGGGEAQEYLFFQLMFLLVYVFYVMLQLYLYCYV
GERLSVESMEIVNAAYNTEWYTLPTNITKMLIIVMCRAKSPLTVTAGRFCSFTLQLFSEVLKTSMRYLSVLYAV
KDKIKR

>AmOr115

MDFAMGWNRFNLTLLGVYPEPRKMSRNSRLMSSLIFWFTTLVTFTFICAPQTANLILKSTSLDEVIENLSINIPI
VFALIKQIVLRYYKKALTELLGEMLADWSGPIGDQDRETMLRNARLSRAISIVCSTLTYFMLLAFVSLQVWSN
AENASETDLGGLLHPATFPYETSKSPNYEITWLGQLMGTVLTAICYSCFDTFLAVLVLHLCGQLTVLGTALED
LVNATRRNDYKTFEQRLSSIVNRHNHLSRFAVIVEDCFNITLLVQTLICTAMFCLTGYRMITSVDREDEADVPI
VGIIFFIIHVIYTMLHLFIYCYVGETLLGQSTGIGLSTYHCNWYDLPSRRAVLLMIVIRRANVSFQITAGKFSPFSL
EFFNAVLKTSAGYLSVLLAMKDRLVEGK

>AmOr116

MTNHLEKQIKLKKINSNKHLQNNLSIIYYIGLWPDRVKYKYLYNLYTICSLIFLVGIIIVSEIIYIIINWGKIEIMMT
GLTILMTNSTYAAKVIYIICRYERIKNLVDITNSEIFNRDNDKYKHIISYYNWQGIFHHIAYQGFASICIFSYSCIP
LQSAFSGKSKQLPIAGWYPYNVTSTPIFEIACLHQVLVILINCINNIAIDTLITGFIIITCCQLTILKKCIARNNNIN
IEKSPSKIYNKFYENLKHCVKHSIIIFDFTKQIQDIFGIIIFFQLFVNCIIVCLAAFNLSQIKNYITPEFFGSLLYICC
MIYQIFIYCWHGNELYLHSMKICLSAYKNNWWNNNKNFNYALLIIMIRTQIPLIIIVGKVMELSLQNFLLILRT
SYSIFTLLKTFTT

>AmOr117

MKKPFNKSIDYYILPNKIFCSIAGMWPIDEKSSIFSKIFAYVRLIFGLIIVNSFFIPQIIIIVMNWKNIKIIAGIGCVLT
TITQVLFKMIYLIARREKTYSLYYKIRNLWNSSNDSKERPYEEFAYWARIFSIIFYSSCMCNVFTFSIAAAIDYFKF
EYNANNTENNRHLPFIVWYGTDISASPSFEIVFFYQIISSSICASVISGLDTSLMTIILHVSGQFKLINIWINNIGIEI
NCNPNYMRKLKVDLIKCIRHHQQLIHVVNNVNNLFTPIIFIQLLTSGIEICLSGYAVLDNNSANADLLKFISYFI
SMGIQLLLWCWPGEILIQESQEIGHVIYLNIPWYNLPPIYQKYLYFMIVRSQQYCRITALTFQTLSICTLSNVFNT
SVSYFTLLRQMQQ

>AmOr118

MINRPLEYSLRIFGIWPDSPYPKLKIITWIIILPTFLVFQYWYCITHIKLGLIDLLDGLSLTLSNTLVFIKLIVIWFHK
RTFYEILMSMKEDLNNNKHSATENKRIIMDKSMLSSRISNFLISYFAITFFLYSGVALVIFDEDQGKFLVRMEFP
FIATISPRYEIILITQFIFESFIVYGAATSIALIAALILYVGSQIDLFCQNLTFHSYKKRESQDTIKDIIVRHQKIIQLSK
NIETIFTYISLCQFVSNMLVICFISFVLTVSLHTEQTIVLIMKCLPYYIAVNCEAFILCYTGEYITSKSENINKAVYN
FLWYNLKPRDVRIMLMIILRSQKQLTLTAGKFICLSLEAFANMLKASASYVSVLYARY

>AmOr119

MHTQRDTSEITYSHDTATSRKLFYLLVVVGGQMAHASGHEWMKSIRTLTSIKINYLKYSGLGEIDSSCSRILKY
AYFVYKVWMLVSMCILAITVFADIYTNMDNLSITTDDGCIFAGIFVVIFKAMNLQIQLESVKKIIDKYHTRNK
VMFFGFCVIGACLGFALLCFTPMENGLPIRAKYPLNTTVSPWHEISFFVETCAVSGGLLGIIVMDSMTTFKCSLI
TMLLDALSVNFENCNGETKRTICNRHGKEERNDNNNRFLDRYKKCVQFHQRLVVISRDYNKIYSLSMLVQM
ISSTSIICLTGFQAVVVGGQSSNIMKYGIYLSAAMSQLFYICWLGNELGYASSTLDKNQWFSGWCNERLTGIG
QVFTLSTVFTRKSIILRASVFYVLSLETFIAIIKRSYSFFTLLNNMDLTDH

>AmOr120

MSNQTNTMNIRNYIFINQLVLKFVGFYPINILRYVICISCIMFIVIPQIIMIYINWNDLNIVMETGSTLLTILLAAL
KSIVWIFNRKKLEFFIEFMLTDYWKIIETNVFEYLQEYAIYAKNITKGYFFSMCNALLFFFSLPIIETLTKNENLN
NFTIKNFPFAASYPITFYKFPFYEIAYISQILATSICCLMMLAIDSLIATALLHTCGHFTVLKENLKNLDTYIYDLT
KTNLKTNSKYINKNLYEIKTQIIYIIKHHQLVLWFCDNMEKNFHLILFLQAITSSLIICFVGFQISIALTERSKFLE
SFSHLIVSLFQLLLFCFPGDILIRQSFNISIAAYSMQWYQLPTFIKDEICMIILRSQRPSFITAGKLYIMHLENFTAI
LSTAFSYFMMLQSFNTEA

>AmOr121

MHTSESKKYSKDYEWAVRLNRFSLNVICLWPVEEQNMRKQSWTKLHIMTCFMLITFVCTIPCLCALKQCNN
LMEVTDNLAYSIPLIITTIKFIVVSSKKKVLSLIVNMVAKDWAKLKTDHEKDIMIRRARIARIINIFGYILICILIW
LLMILPRFGITIRYVTNETDAKKLFPLPSYYIFDVSETPYFEIMYALQSISLLIAAFCYAGVDNFFGILILHICGQLT
NLRFQLANIKESEASNFILIAIVKDHIRLIRVIELLKMFVEQIINLIITIFKYFFKCLKMYIEEEQFSLFRIIYLICNFT
NTFLQTFLYFMAGQMLVTQSEEVHNAAYECEWVSLKYTKAKSLIIIMARSKKPLYLTAGKLFPVTMLTFCNIL
KISLSYISFLLTIL

>AmOr122

MNMDVFDKQYRIYRIILKIVGLWPYDKSIYVWIQRICLSMYFLIGVIFQIILLVKSEITLRNYIVTLSAIFPLLLFFIR
YIYYITMFPYVEILFDNIRTEENLLQDTTEIQIQTKYLDISSHIIYIFCCMTFAFIVAAIIFLVNPVILDLRNPLNESRI
FYFDLLFLLDDQSAYIKIFLILNFMLNILFGLLSITSTESFTNIFSYYICRQFNIVNYRIRKIIEDLSTRNLSKIDLKIK
DIHRVVDIHCHAIELLYKALITMDNRIEIFGSTLIVIYHLMIAFYNNHCGQLIIDSNLGIFNELFASTWYRIPLKA
QKLLLFMILRSSMGCEICLSGLFTPSYAGLTSMMSSSFSYCTVIYSIQ

>AmOr123

MNVFDNQYRTYRIILKIVGLWPYDNSIYVRIQRICVLIYFLIGILIQIFSLVKSEISLRNCIVTFSTTFPIVLFCLRYIY
CLTLFSYAKVLFDDICIEEHLLQDTTEIQIQTKYLDISSHIIYIFCWLSFICVASTWIFILNPVILDVIMPLNKFRLH
YSVIFLSNDRRKFIDIFLVLNSIIIFTFGLLSLICSELFTNIVSYYICRQFHIVCYRIRKIITDLSMPNLPKTDLKLRDI

HRVVDIHCHAIELLYKALVTTDNRMEILGSTLIVIYHVMMALYNNHYGQLIINSNHGIFNELCASTWYRIPLK
AQKLLLFMILRSSMGCEICLSGLFTPSYAGLTSMMSSSFSYCAVIYSIQ

>AmOr125

MNMDVFDKQYRIYRIILKIVGLWPYDKSIYVWIQRICLSMYFLIGVIFQIIVLVKSEITLRNYIVTLSAIFPLLLFFI
RYIYYITMFPYAKLLFDDIRTEEYLLEDETEIQIQTRYLDISSHIIYIFCCMTFAFIAAAIIFLVNLIILDLRNSLNEFR
FYFDLLFFFDDQSAYIKIFLILNFMLNTLFGLLSITSTESLTNIFSYYVCRQFNIVNYRIRKIIEDLSTPNLSKIDLKI
KDIHRVVDIHCHAIELLYKALTAMDDRMEILGSTLIVIYHLMIAFYNNHCGQLIIDSNLGIFNELFASTWYRIP
LKAQKLLLFMILRSSMDCELRLSGLFTPSYAGLTSMMSSSFSYCTVIYSIQ

>AmOr126

MNVFDNQYRTYRIILKIVGLWPYDNSIYVRIQRICVLIYFLIGVLVQIFSFVKSEISLRNCIVTFSMTFPTVLFCLRY
IYCLTLFSYAKLLFDDICTEEHLLQDTTEIQIQTKYLDISSHIIYIFCWLSFICAAASCILILNPVILDVIMPLNKFRL
HYSLIFLSNDRRKCIDIFLVLNSIIIFTFGLLSLICSELLTNIFSYYICRQFHIVCYRIRKIITDLSTPNLPKTDLKLRDI
HRVVDIHCHAIELLYKALITMDNRMEILGSILIVIYHLMMALYNNHYGQLIINSNHGIFNELCASTWYRIPLK
AQKLLLFMILRSSMGCEICLSGLFTPSYAGLTSMMSSSFSYCAVIYSIQ

>AmOr127

MNVFDNQYRTYRIILKIVGLWPYDNSIYVRIQRICVLIYFLIGILIQIFSFVKSEISLRNCIVTFSTTFPTLLFCLRYIY
CLTLFSYAKLLFDDICTEEHLLQDTTEIQIQTKYLDISSHIIYIFCWLSFICAAASCIFIVNPVILDVIMPLNKFRLH
YSVIFLSNDRRKCIDIFLVLNSIIIFIFGLLSLICSELFTNIVSYYICRQFHIVSYRIRKIITNLSMSNLPQIDLKLRDIH
RVVDIHCHAIELLYNALITMDNRVEIFCSTIVVTYHLMTALYNNHYGQLIINSNHDIFNELCASTWYRIPLKA
QKLLLFMILRSSMGCEICLSGLFTPSYAGLTSMMSSSFSYCAVIYSIQ

>AmOr128

MNVFDNQYRTYRTVLKIVGLWPYDNSIYVRIQRICVLIYFLIVVLVQIFSLVKSEISLRNCIVTFSTTFPTLLFCLR
YIYCLTLFSYAELLFDNVHTEEHLLEDTTEIQIQTKYLDISSHIIDIFCWMSFICVASTCIFMLNPVILDVIMPLNK
FRLHFSLIFLSNDRRTYIDIFMVLNLIIILIFGLLSIVCSESLTNIFSYYIYRQFDIVSYRIQKIIADLSMPNLPKTDLKF
RDIHRVVDIHCHAIELLYKALITMDNRMEIFGCILVVAYHLMIAFYSNYCGQLIIDSNLGIFNELYASTWYRIPL
KAQKLLLLMMLRSTVGCELHLSGLFTPSYAGFTSMMSSSFSYCAVIYSIQ

>AmOr129FIX

MNVFDNQYRTYRIILKIIGLWPYDNSIYVWIYRLCLLIYFLVVVLVQIFSLAKSEISLRNCIVTLSTTFPTLLYCLR
YIYCLTLFSYTELLFDNIRTEEHILQDMTEIQIQTKYLDISSHIIDIFCWLSFICVAATWIFILNPVTLDVIMPLNKS
RIHFSLIFLSNDRRTYIDIFMVLNLIIILIFGLLSLICSESLTNIFSYYVCRQFDIVSYRMRKIIVNLSMPNLPKTDLK
LRDIHRVVDIHCRTIELLYNALIIMNNRMEIFGSALMVMYHLMIAFYNNHCGQLIIDSNFGIFKELYASTWYRI
PLKAQKLLLFMMFKSSVGCELRLCGLFTASYAGFTSMMSSSFSYCAVIYSIQ

>AmOr130

MNVFDNQYRIYRIILKIIGLWPYDNSIYVWIQRLCLLSYFFANIIFQIVSLLRSEITLQNSILILSITCPLVLFLLRYIG
SIACFPTIKIVFKHIRTEENIVQDSIESQIRMKLIDDSHHIINIFFWMTYTTIVIFIIYVSYPIILDFMIPLNESRTHFIY
YITTFSHNQSIYLDILDFNFMFTGIFGLLSVACSESITGIYSYYICILLKIVSYRIQKIIMYLAMFKLSPKQIDSKLIEL
YRVVDIHNQTIELLVNATLIKKNQLEMLFCFTLVAIHLVIIFLNNYNGQIVMNSSQELFDELYNSMWYFMPLK
AQKILLLIMLQSTTKHAFNILGLFTPCYAGFSTMLSSSFSYFTLMYSIQ

>AmOr131

MDVFDKYYHTYRIVLKIIGLWPYNNSVYVWIQRLCISALFLGNIIFQILSLIRSEITLRNCILILSTTCPLIIILLRYIS
FIIFFPMVKLLFHHICVEENAVQDLIEIQIRMKYIGNSRHMIEILLRVTFLTITLFSIFLLYFVTMDFIMPLNEFHR
HILLYVTLFSVNRTIYFYILYLNFLFVITFGLLSLICTESIVGLYSYHTGMLFKIISYRIRKIITYLTMFNVSSKQIDSK

LAELHRVVDIHNQAIGLVVNAITIKKDQLEILITLIIFANHLMIMFLCNYNGQILINSNEEFFHELYIPVWYFVP
LKVQKILLLIMIRSSMACIFHIFGVFIPCYVGFTTMLSTSFSYFTLIYSIQ

>AmOr132

MDVFDKYYHSYRTVLKIIGLWPYNNSIYVWIQRLLLLTLFLGNIIFQIMSLLRSEITLRNCILILSTTCPLIIISLRYI
CFILFFPMIKYLFHHMRMEENIVQDSIETRIRTKCINDSCHMIDIFLWMIYAIFAFCIILLLCPIILDFIMPLNESRI
YIAHYITIFSDKRIIYVDILCLNYMFLMIFVVLSIMSTESILGLYSYHTSMLFKIISYRIQKIITYLTIVNLSSKQIDSKL
AELYHVVDIHNQAIQLLENAIIVTKDHLEILICLMLFVKQLMIMFLCNYNGQILIDNSEELFDELYFSIWYFVPL
KVQKILLLIMTRSSTTCMFHILGVFVPCYTGFTTMLSTSFSYFTLMYSIQ

>AmOr133CTE

MEENIIQDSIEAQIRTKYISDSRHMIEILLWMAYATITLYSILGLCPIIFIILLNESPIRMLHYVTLLSVNGTIYFYIL
CLDFLFIIIFGLLSMICTETIVGIYIYHTSILFKIISHRIQKIIAYLNMFNLLSNQIESKLAELYCVVDIHNQAIQLLV
NAITIKKDQLEILISLIIFVNHLVIMFLCNHTAQILINNNEEFFHELYISVWYSVPLKVQKILLLIMIRSSMACIFHI
CGVFVPCHAGFTTMLSTSFSYFTLMYSIQ

>AmOr134

MDIFDKHYYSYRTVLKIIGLWPYNNSIYVWIQRLWISALFLGNIIFQIVLLLRSKITVRNCILILSTTCPLIIISLRYI
CFILFFPMIKYLFHHMRMEENIIQDSIEAQIRMKYIGDSRHMIEIFLWMAYANITLYSILGLYLIIFIMPLNESPIR
MLHYVTLFSVNGTIYFYILCLDFLFVIIFGFLSIICTETIIGIYIYHTGVLFKIISHRIQKIITYLTIIDLSSKQIDSKLAE
LYRVVDIHNQAIQLLVNAITIKKDQLEILISLIIFTNQLVFIFLCNHTAQILINNSEEFFYELYISVWYFVPLKIQKI
LLLIMIRSSTACMFHIFGVFVSCHAGFTTMLSTSFSYFTLMYSIQ

>AmOr135

MDVFDKQYHSYRTVMKIVGLWPYNNSIYIWIQRLLLLTFFLGNVIFQIVSLLKSEITLRNCILILSITCPFIIVSLRY
VCFIVFFPTIKLLFHHMRVEENIVQDLIEIQIRTKYINDSCHIIDIFFWVACTNITLSSISLLYFITLNFIMPLNEFRII
HYITLFSVNRTMYFNILCLDFIFVVIFALLSVICTESIIGLYSYHISVLFKIINHRIQKIITYLTIVNLSSKQIETKLAEL
YRVVDMHNQAIELLVNAIIIKKDQLEISISFIFFVNQLIIMFLCNHSGQILIDNSQKLFNELYISIWYFVPLKVQKI
LLLIMIRSSTRCMFHILDIFTPCYAGFSKMLSTSFSYFTLIYSMQ

>AmOr136

MNVFDKHYHTYRTLMKIVGLWPYNNSIYVWIQRLWFLMFFFGNIIFQIMSLLTSAITLQNCVLIFSTTCPLIIVL
FRYIGLILFFPTIKLLFHHMCMEEAMIQDSIEAQIRRKYIDDSCYMIDIFFWMTYVGIALCSILLLCPITLDFIMPL
NESRTRIVHYVTIFSDKSIIYMDILCLNYMLLAILVILSATCTESILGLYSYHTSIMFKIIGHRIQKIVKYLTMFNLS
SKQIDSKLAELYRIVDIHNQAIELLLNAIVIKKDELEILISFIFFTTQLVITFLNNNCNQILIDNSQELFIELYISMW
YFVPLKVQKILLLIMIRSSTACMINILGVFTPCYIGFSKMLSTSFSYFTLMHSIQ

>AmOr137CTE

MDIFDKRYCTYRTMLKIVGLWPYNNSIYVWIQRLWLLIFFLGNIIFQVVSLSSEITLRNCILILSLIFPLTIILVRYV
SCVIFFSMIKLLFHHMRMGGNIIQDSTEIKIRKKYINDSCHMMNIFFWIIYGIAALSIIFILYPMTLDFIMPLNRTR
IRIIHYITIFPYNRTMYLDILSLNFMFVGIFGSLSLACTESIFGLYCFHASILFKIIIYRIQKIVTYLTMFNLSSKQIDT
KLTELYRAVDIHNQTIGL

>AmOr138NTE

VVSLSSEITLRNCILILSLIFPLTIILVRYISCIIFFSMIKLLFHHMRMERNMIQDSTEIKIRKKYINDSCHMINIFFZII
YGIAVLSIIFILYPMTLDFIMPLNKTRIHIIHYITIFPYNRTIYLDILSLNFMFVGIFGSLSLACTESIFGLYCFHANIL
FKIISYRIQKIVTYLTMFNLSSKQIDMKLTELYRAVDIHNQAIGLLVNAIIVKKDQLEMLISFMILMAQLIITFLC
NYNNQILIDNSQELLDELYISAWYFVPLKVQKILLLIMIRSSTSCTFHILGVFIPCYTGFSKILSTSFSYFTMIYSIQ

>AmOr139PSE

MDIFDKHQSYHSYRTIMKIIGLWPYNNSIYVYIQKLZLLIFFLGQIIFQXDAVIAFFSISLLYPIILDFINSLNESRTR
IIHYFTIFFHSRIIYIDILCLNYIFLAIISLLSIICIESMIGLYTIVTTSLFFKIIGYRIQKIITXLTIFNLSSKQINSKLVELY
HVVDFHNQVIELLVNMILIRKDQLEIFMFFIFLVSQMMIMFICNYSSQILIDNSQELLYDLYISMWYFVPLKVZK
ILLLIMIQSSITYMISILGVFILCHIGFSTMLNTSFSYFTLIXSTQ

>AmOr140NTE

LRGLYSVWGVNYDAVIECMPPIISIFQSASMYFNGIFNTKKIKNILLFIKNDHKYYINRPENIILQKYDLQGKKI
TFYYILYVYTTLFVYLLLPTIPLIIDFITSSNHSQKRNFLFELDYGMDKQQYFYYISIHSYIGTAIVANLIASCDTM
YMLYAQHAYALFAIVSYELKTIHILNTNNLINVTDHHLLEKYKNITLLSKDEKKVYRKLFICIKNHQNAIKYS
NLLESLFTKSILVQLFFNVLCLSITGVETVIKLGNLSEMMRFGSFTFAQAVHIFFLCLPGQRLLNHSEELHVSAC
EVTWYIFPKKYQNLYKFLLARSLIFSKLTAFKVTTLSMQTFLAIIQTAMSYFTVLLSTT

>AmOr141

MIDEKTKREFDKTIDLNLFLLKLCGIVPCGDGFARNILAWLAFSCLTIYSISYVHEFITNTTNLTTALESVAMIISI
VGGHARYTILLWFRDICQTMLNVCEIFWSNLKPHEKKIVQSYTRKTTRLTRWYLASCVLTIAFYAFLVLFGSLF
DQSKDFEHMRNDSSLVPSEAGNILESMSKRHLPYAFFLDVQKTPWYEIVYAVQLIGMFNVGFTCVGVDTVG
ALFILIICGYFDTIQSRIENLHSFDTSLSSSLLNILSRKITTAKMSDIKTEASNSVQMRNLRMCVIHHQLLLNRFC
EDIEHLTSGMFFIQVIASTYNISLVGFKLLEDTPDKFKYITQLIILIIQLFLCNWPADLLLSKSIDISRATYSMPWY
GYSYNLQKITNILMIRSQKAVRLTAGKFIGLSLETFASMISTAASFFTMVRSMN

>AmOr142

MKNRLTPEKAILFTKLSVALTCSWPPSPLATKAQHLFFNALWCIAFLTSVMLFLPLLAAIYVYRKHPVILGKTV
SLTAAVAQVTIKMIICRLQQKRFQMLYSEMENFCKQATNEEKIILQRYVDRYKYFHSFYILWSFLTTIFVICGPL
YTVQTFPTHAIYPFSVRRHLYKGLIFFHQSLVGFQVSSGMAIDTQIALLLRYATARFEILGIQFNNAKSDGEFD
ACIKKHDELLRYSREIRQSIKFLILATNGTTVIAVIFGSLNLIANQPLILKALYAIVVFSASVELFMYAWPADSLM
HMTMKMATKVYNMDWYGKDIRTQRKILFIILRSQKYESFGINGIVPALSLSYYGKYLYTSLSYFNALRIMVED
TVN

>AmOr143

MNIRQILYILELIGTFTCTWPINPNISKRRIIFRNIFWIFSILNVILLMTSLMLAVVYFRNDILMSLKTASEMAALL
EVVLDLILCKWNNSEFQVLIEEVKSFVEMANEYEIKILQGYVNRYKKFFSTVSMGYISTAISFSLMPLFSAQKLP
ADGWLPFSTEPFGIYCIIYFNHVYCILQTAFCIFVDFTIVILFSFPAAKLDVLRSKLRHVNNYDTLVSCIKEHQKII
GFVEDTKATVETLLFKTNVTMGSTVMCGAFPLLNNQSLAAISQFLPLVLSGILHLYVIAWPADDLRESSVQFS
NSISDIQWLGQSNKMKSCVIFMMMRSQKAFLIRMSNLLPPLSLEYCSNFITTVSSYFMAMRTMIES

>AmOr144

MGMLNMDIRQVLHILELTGTFTCTWPINPKDSKKYIIIRNILWTFTILNVIFLTISLMLAIFHFRSNIPKSMKTAS
EMAALLEVVLDLVLCKWNNSELQVLIEEVKSFLEMASEYEIKILQGYINRYKKFFSTVSMGYILPASSFILMPLF
SAQELPAEGWLPFSIEPLGIYCVVYVNHIYCILQTSFCIFVDFTIVILFSFPAAKLDVLRSKLRHVNNYDMLVSCI
KEHQKILGFVEDTNATVETLLFKTNVTMGSTVICGAFPLLNNQSLDVVTQFLPLVLSGMLHLFVISWPADDL
RESSIQFAESINDIQWLGQSKKMKSCVIFMMIRSQKLFLIRMSSLLPPLSLEYCSNFVTTVSSYFMAMRTMIES

>AmOr145

MGMLNMNIRQVFYILELTGTFTCAWPINPNDSKTYIIIRNILWIFTILNVIFLAISMIFAIFHFRSDIPKSMKTASE
MAALLEVALDLALFKWNNSELQILIEEVKSFLEIADEYEIKILQGYINRYKKFFSTVSMGYILPASSFILTPLLSDK
ELPTEGWLPFSIEPLGIYCAVYVNHVYCILQTLSFCIFVDFTIVILFSFPAAKLDVLGSKLQNVNNYDMLVSCIK

EHQKILGFVENSNATVETLIFKTNITMGSIVICGAFPLLNNQSLDVVTQFLPLILTGMLHLFVIAWPADDLRESS
IQFAESINDIQWLGQLKKMKSCVIFMMIRSQKLFLIRMSSLLPPLSLEYCSNFVTTISSYFMAMRTMIES

>AmOr146

MFRNATPEKAIAFTQFIVSLSCCWPLPSTATKLQTRCFKIIRSLLFLNSLLLFFPLLYFVYVNRNDNTTFCKAMS
LSLAVVQVPLLSSFCITQYDRFQRLIKEMKFCCENANSYERQVFQGYAKSYATFYGVSAIWFYWCALIVVVGT
LFISDPFPTNAEYPFPVHFEPVRSIVFVQQALVGFQCSAHLCVNIFCALLLLFAAARFEILMNELRAVENIESLI
KCIEKYYAIRRYAEEVVNSARYTTLITLCICGVESVFGGIIFIGRQPFTVKLQFLTLSATTLLAVFMCAWPADYL
MDVSENTMRAVYESEWYKRSLKLQKFVLFATIPQTPVILKVRCIIPAFSLNYYCSFITNVLSMFTALRVLMYKD
EN

>AmOr147FIX

MLKQVSPEKGIYIIWLSVALSLCWPLPINSTRKQIVCMKILQIGAIISAFMILLPLIYTIYLNLDNLNIFFKSICLLM
GVFQHIVQTITCFIKYDSLQRVVEEMMICIKEMQLNEIMCAYVAKCNIFYGGTIVLIYTTATVFILGPTFLPITFP
WETEYPFQVNYTSRNFIIYMHQFFFTYQCAAHICVSMFVALLLWFTSARFECLVKELQKTTNIEMLIVCLKKQ
LLLRRYAEDVVNCIRFIIFYTMAVSTIVLTLSGIILITTSSLLVKIQFLTICISILLEIYMYAWPADHMYDMSITVLQ
SVYDSMWYGQTLNMQKLVLITLIYQKPVTISINVVLPQLTLHYHCLYVSNAFSIFTAIRAIIYDR

>AmOr148

MLKYVTPEKGIYIVWLSVALSLCWPLPASSTRKQIVCIKILQIGAIISAFMVLLPLIYAIHLNIHNLINLFKCICLLI
CVFQNIIQTIICFIKYDVLQRVVEEMMTCVKEEQLYKVLCIYVKKCNIFYGGTIVLTYGAATVFVLGPTFLPISFP
WETEYPFQINDTSRNIIYIHQFFFTYQCAAHICLSLFGALLLWFAAARFECLVEELQKITNIDMLIVCFKKLLLL
RRYAEEVVSCIRFLVFYAIAVGTFMLTLSGIIMIINSPILVKIQFIIICMSSLMEIYMYAWPADHMQDASINILRSA
YNSIWYEQSLDMQKDLLIILMYQRPVILSINVLLPELTLRYYCSYVANAFSVFTALRAVVEDK

>AmOr149FIX

MLKQMTFKKIINIIWFSVALTFCWPLSANSTKIQIFVFRILQIISIINAFMLIVPLSYSIYLHYDDIAIIFQSLAILVGL
SQMIIQTVILCFIKYNFLQRVVEEMIICIKEAQQYERKIFCKYIENCNIFYGSSLTITYLVVIIYIMGPIVLPTPFPVD
TEYPFHVNSTIIKIIIYLQQSLLIFQCAGHLCISIFCALLLWFTAARFECLIVELQKITNIGMLIICIKKQLRLRRYA
RNVVNSFRFMIVYAIGVSTFALILYGIIMIVKAPLIMKIESVTLSFVLLLQIYIYAWPADHMKDMSINVSKSVYNI
IWYKQTLRMQKDLLNVLIYQRPITFSVDCILPELSLRYYCSYVSNIFSIFTAVRVIIEDN

>AmOr150FIX

MLKQLTPEKAIHITWISVAITFCWPLPANSTKIQVFMFKTLQIISIINAFILLLPLLYSVYLHFDDIVIVFKSIALCV
GLSQMIIQTAICFVKYNTLQRVIEEMITYVKEAQQYERKIFHKYIKKCYTFYGCSIICMYLTGLAFIIGPAFSPASF
PADAEYPFQINYKSIKVIIYLQQTLVGFQCTAHICLSVFGALLLWFTAARFECLIVELKKITNISMLIVCIKKQLH
IRRYAKKVVIGFRFIILCAIGISTFALTLGGVIMIKKAPFIVKVQFITLILTLLTEIYIYTWPANHMKDMSINVSQSI
YNITWYKQTLRMQKDVLTVLMYQQPIILSINCILPELTLHYYCSYLSNAFSIFTAIRVMIEDDP

>AmOr151

MLKQLTPEKVIYITWVSVALTLCWPLPANNGKIQVFMFKALQIISIINAFILLLPLLYSVYLHFDDVIIVSKCVA
VSIGLTQVITQTIICFAKYDSLQHVIEEMIICIKAAQQYEEKIFHKYIEKCYTFYACSITCMYLTATAFIIGPAFSPA
SFPIDAEYPFQINYTSVKIIIYLQQTLVGFQCAAHVCLSIFGALLLWFTAARFECLAVELQKITNIGMLIACVKK
QLRIRRYAKKVVISFRFIILYAIAVSTFVLILDGIIMIMKVSLIVKVQFITLSLTMLTEIYIYAWPADYMKDMSTNV
SKSVYNITWYKQTLRMQKDVLNILVYQQPIIFSVNCILPELSLRYYCSYLSNAFSIFTAIRVMIEDDP

>AmOr152

MLKQIISEKTIQIIWFSVAITFCWPISLNSSKTQVFIFKILQIISIINVFMLLLPLLYSVYLHFNDIIIVSKSIALSVGLI
QVIVQTIICFIKYDSLQHVVEEMIIYVKEAQQYEKKIFHKYIEKCHIFYGCSIACIYLTATVFVIGPVFSSASFPAD

AEYPFQVNSTSMKIIIYLQQSLIAFQCAGHACLSIFGALLLWFVSARFECLAVELQKTTDIGMLIVCVKKQLHIR
RYARRVVISFRFIILCAMGVSIFSLTLGGIIMITKSPFIVKVQFITLILTLLTEIYMYAWPADHMKDMSINVSKSVY
NTIWYEQTLRMQKNLLNILMYQQPIILSINCILPELSLRYYCSYLSNAFSIFTAIRVIIENNPS

>AmOr153PSE

MVKEMIPEKTIHITWLSVALCWPLSVNSGKTQVFIFKMLQIISIVSACMLLLLSSYSIYFXHGQCRIFKNYHRFID
VAQNIIQTVICFYIIKEMKICIKETQEYEIEIFQKYIAKFKTVWGCNITCMYLTALAFTIGSVFISTSLSCDAEYPFQ
LNYTLVFAIRYQSFLSYQCAYACADHXLLWFTAPRFECLCVELQNVTNINMLIVCXYAKKMINWFRFIIFNAI
GLSILVFTLASIILIMISICMYIVVCSCMYNFINKNYYMYIWPADYMTDKSINVSRKIYDSMXYKQMLKMQKNL
LKXLIFQRPVXIYRLZLLSKLILRYYCLYLSNVFSIFTALHVLEDNI

>AmOr154

MLKKVTPENVIYIIRLSVAICCCWPRPFNSTKNQIFAFKVLQISTIISAFMVFLPLLYSIYLHNDNIIHVFKCICLSI
GITQLIVQTLICFIKHNSLQRVVEEMVNCVKQAQQSEIEIFYKYIEKCKIFYGSSIAFSYLAATAFMLGPAILPISF
PLEAEYPFHVNESLITIIIYMHQSLVSYQCSANVCVSIFGALLLWFTVARFECLIEEFQKCSNIDMMIACIKKQL
QLKRYAEEIINCFRYIVLYGIAVTTFALILCGIILLMNIPLIVKIQFVIICITIMTEVYMYAWPADYVKNMSINISRS
VYELSWYEQTIEMQKNFLNVLVYQKPVIFSISCIVPELSLRYYCSYLSNVFSIFTTLRVLLEDTSA

>AmOr155JOI

MLKKATPEKIIDIIRFSVAICFCWPYPLNSSRNQIFGFKVLQISTMVSACIMLLPLLYSIYLHNDDVIHISKCICISI
GVTQLIVQTLVCFIKHNSLQRVVGEMMKCVKEAQQNEIEIFSKYIEKCKIFYGSSIIFSYLTSTAFMLGPIILPISFP
FDAEYPFHVNHSLVTIIIYIHQSLVGYQCSANVCASVFGALLLWFTVARFECLIVEFQKCTDIDMVIACVKKQV
QLRSYAKEVIKCFRYIVLYYITITTFALIISCIILLMNVPLIVKMQFIIICVTIMTEIYIYAWPADYVKNMSINISKSV
YELSWYEQTLEMRKYLLNVLIYQKPITFSISCIVPELTLRYYCSYLSNAFSIFTTLRILLEDNST

>AmOr156

MIEQVMLKRVIYITWLSVALCFCWPVSANTSRNQIIVFRFFQIFTIISSCLGSLPMFHSIYLHQDDIVIVAKSISIM
VVLIQLIVQTTICAIKHDTLQHIIEEMITYMKEAKQYEKKIIQKYVSKCYILYGSAIIISYLTTTIFILGPIFLPISLPFY
TEFPLSLNNTAVYIIIYFHQCFFAYQCSATVCLSIFGALLLWFVVIKFECLIMKIQNISNKDMMVICIKKQLQIRR
YAKEIANCFRHIIFYTIIATSFNMILAGIILIMNPLLVIKIQFMITCFTALIEVYLYAWPAQYMDDMSKNVSISAY
NLKWYEQTSEMQQNILIMLIFQKPISLSINFLMPKLSLRSYCAYLSNAFSIFTALRVILKDNSI

>AmOr157

MRRARPEKSVYLVWLSVAMTFCWPLPPDTARKRIVGMKVLLIISIVNGCAVILPMLYWIHLHLDDIISLFKCIC
VALCLVQYVAQTIVCLVKYDTLQRVVDEMMGLIEERRMYEILRAYASKCNTLYGASIASIYVCGTSFIFAPLFL
PNPFPFETEYPFHVNTTTRIFIIYASHVLVIFQGTAHMCLCMFGALLLWFTTARFECLIGELRGVTSVDTLVVCL
EKHSRLKRYAEEVVSCIRFLVFHAILLGTFVLTLCGIVLIINSPLIVKAQFIIICVCILLEIYLYALPADYMYDMSM
NISRSVYDSIWYEQRLDLQKALLTVLAFQKPIAVSINVLLPELTIRYYCSYVSNALSIFAALRTVVE

>AmOr158FIX

MLKQITPEKSIYIIWLSVALSFCWPLHINSTRKQIMYIKILQISAVVNAFMVLLPLIYTIHLNMHNLINLFQCICL
LICIFKHIIQTVICFIKYNALQRVVEEMMICVKEEQLYDILCMYVKKCNIFYGGTIVLIYGTATVFVLGPIFLPISF
PWGTEYPFQVNYTTINVIIYAHQFFLVYQCAAHTCLSLFGALLLWFATARFECLIKELQKITSIDMLIVCLKKL
LFLRRYAEEVVSSIRFLVFYAITISTFTLTLSGIIMIINCPLFVKMEFITISISLLVQIYIYAWPADYMQDMSINVLRS
AYNSIWYEQTLDMQKTLLIMMAYQKPVTFSINVLLPELTLRYCCSYVSNALSIFTALRAVVEVT

>AmOr160

MRRPISSYVELFYDKNVISWSKRLLGLSGLWPDNRNDVRFFLYITYVVIFTWLEIVTLVQNIHDLEKTLKNITLS
FPTILIVLKAVMFRMNMHLVLPLLTVVKRDVNEGLYRSAEERRTVVWYNVAATLFSTSSALSLFFVPTLFYAK

PIIGCLLSKYNNCTLPFELPMKVNNVYEITKLQTYALFCVYLIPTSTLLTIGATGADSLLVTLTFHLCSQLSIVAY
RMRNVNIEPKIYFPKMKALVERHTELLRLANILANTFSSLMFVQTLGLIFSLCIVVYQLLMTSESGEDMNTIHFI
IYSCAVILLAFCYCFLGECLINESSEVQMACYFTNWYDLPEQYTRSLIFCIARAQKPLYLTAGKFYVFSLETFAVI
VKASMAYLSVLKSII

>AmOr161

MGEFRNEEYDQLIKPIMITGKIISIWPLAENSSRITITFRRFHLFCMFFLVIVMSVAVTADVVHNIDDLDEATEC
ALICTAFYLCVVRLLVYSFHQKDMLYVVNTMKEDWLSSSDQDRLIYAEKTMFAFRLAKYFITTVAITIVMFMS
VPILEIYVIGNSDKVLPFRGYFFINQTVSPIFEFLYLFNVTAGGFGGSMIAGATSFNLVVIIHGSGKFAVLRRRME
ALNGADPNSAAIMGDNVIRHQQAIKFADTLERIINLLALGQFVISTGLICFAGFQITSMMEDKGRLMKYSTFL
NSAILELFMFSFSGNGLIDESEGIGESAYNSGWIGSRFCRSVQIMMMRSKIPSKITAAKFYSMSLESFSAVLSTSFS
YFTVLTATKNE

>AmOr162PSE

MKRLMDIMQEDWKFHARLRNEYEILCEHYAIARKITTSFVAFLLGLTTPFGAMPLLLNIGDALGLCNISDDRP
LAFRVEYFVDVDKYYYLLLVHSSIGTLGYTVIVLAINSIIIVYVLHECGLCEILRESNNMICKIFLKDSLDIYTLDK
IMNQFIIVQGETRKLCGNGCNGYRITPPQZKGZMVSKCQGLCTLAQTHNRVSIKLITKFQLILTYLFFFLLIFQI
SRFLSLRFAKILEDTNTTSYLLQLGFNMICISFTQFQAIINIEDTPKVLRYVSITIALLCDLLFVSWTGQQLSNSTE
RIFEYTTNGKWYQSSISCRKLLAIMLSKSIAPLRLTACKLYTLNLESFTTIAKTSVSYTMVLCSLQ

>AmOr163

MFKTIITYPVEVCLRLIGVWPYSSYRIMQRIFWTIIMGNSTVFQLWYCISYFKTADLFDLLDGITLTLSNTVTFFK
LIILWFNYRTIHNILTIVFEDWNNRALTDKKKQLMVDNTRLSSRISNFLFGIYSVTCILYSASIALISDDIDNTNN
ELILNNKKLLLKMKLPFDFTIFPLYEFVIVAQFVFECFVALTAGMLMAFSAALVLHIGSQIDITCQELIEIPRHK
GKTSYILKNIIVKHQRILRLSENVKYLFLYTSLIQFLSNILVICFLGFILVNALGTEQESTIFIKCFPYYIAANCEAFI
LCYTGEYLMFKNESIVHAAYDTLWYNLNPRDSRIVLLILIQAQRKLILSAGNFVTLSVQTFASMQKVSASYISIL
MTIY

>AmOr164FIX

MAIKSIINRPVEISLRLIGAWPNSSCQILKYIMWTIVMSIFLIFQYSYCIIHIKTATLIDILDCLSITCSNTLLLLKFIII
WFHKRVLFESLIIIAEDWDNCKFEWNMEIMMQKAILSYRIAKLMLIIFICSIFMYAVSTFFGPDIGASHSDQKKF
LLKMEFPFEATVSPLYEIIITIQLVMQFMFATMAGMFMTIIATFVLHIASQLDIICDRLSEILDEHKEQELRIRIIK
KLIAKHQRTLNLSENIENIFTFISLSQFFFNILVICFVNFILVTSIGTEQAPTVISKCFPYYIALNFEALILCYTGEYL
SSKSENISWIAYNSNWYELSIYEIRVLLLLIMRSQKPLTLTIGKYMKLSLETFANMLKISASYASVLYALE

>AmOr165FIX

MLVLNTLSPSVKFGLHFAGIWPGTPFPYLHKLGWLAAIAALQSYQYRYIVMHYKSDNLMSIIDNLSIAMPFSL
VFIKLIVTWINYGVFCDILSTMEKDCQKYAVIDINNLISKTGQISFYTTTIVMSSYLVSAAFYITGTLAFQRTNSSI
SRELLFKMDLPFETNESPNYEFVVTSQLLIHVSAAFTFGTFSALLLMMVLHIGCQIDILCQNLLDIPHISTSHLK
FFIIRYQEIITFAERVEKLFTYIALSQLVSNTLITCCVGFLIVIAIHEDNGLPLLLKSVLFYMVICLEAFIYCFAGEYL
RIKNESIIEAAYDMLWYNLNPQDSRIVLLILIQAQRQLTLSAGNFVTLSAETFASMQKISASYISILMAMY

>AmOr166JOI

MTSINTISRSVKYGLYFAASWPGASFSILHKFFWTIIFCTLHISQYSYLIMHYKYDALTEIIDNISICLPHSLVCIKL
FTAWTQNTLIRNILLSMEEECQKYAIMDTDNLISKTAYLSYRLTSTIICTCVASTVCYAIGIFSHQEVNVTSSREL
LLKMNLPFDTNKSPIYEFVVIIQYFYQVSAAFVFGVFAAFLLMIVLHVGCQIDIMCQTLMKTTHRDQKKLKFFI
KRHQEIILLAEKIEKFFTYIALSQLISNTLITCCLGYLIVITLHLGNNIILIKYIMFYVAVCSEAFIYCFAGEYLSIKS
KLIADTAYEFLWYNMNPNESRLLIPIILRAQRGFTFTFGKFATLSMESFTAIMKASGSYMSVLLAMT

>AmOr167JOI

MIPIRSISHPIVIGLRLIGIWPKSSYEIIVRFMWVIIMMCAQIFQYQYIINHIGFDNLADLIDSVSTTLPYSLLCFKLI
SFWTKREIFENILIGMYHDWTNAFATDFIVEDMIKKTELAYYCSNLILSIYAIAVFLYVGVFLELSHDHDQENR
SNLSPELLIKMDLPFTYDESPIYEYVFIVQFIQLFFIASSIAVLDALIITLIFHIGGQIEILHKTLKNISINDEKPESSRII
IKSLIDRHYRIIIGSEYIESLFSYIALMQLICNTLIICCIGFLIVVALNSNLKLLIRISFFYIAITLEAFIFSIAGEYLSNKS
LSVSISAYESPWYLLSPKNRGVMILLMVRSQRRLTITAGKFMDLSMQGFANVLKASVSYVSILYAMY

>AmOr168

MNFQNLNRLNALANVVSGNFLPMTNINEKSSVISKIYFVIVWIIQLMYLASCTLGLFNVSWERALKDGTVNM
VLLLEVIILNVYLHSRKKLLRELIGKLNQILINEDEIFRNVTISTTKMLEKPSRIYIIVNVISIIVWISSPLIKLFQKDE
FYHEDFVMPAVFSNQPFSTGVFISGVFLQLFGGEYLLFRKISLDLYTMHLNLLITSQYKYLRIKFATILKENGES
AKDNDKTIRQEMKLLIRHFETVIEMTGILKKLLSPNIGILYLNYVFRFCFLSFMFATTSLSEKLTYTIIVSYTTGAL
IQFYILCYCIQDLFEASTSIADDVVYEKWYSYDVRFQRVILMISLANELKCKISNFQNIDLTLPSFMSILNQAYSI
CLLFLKTKQD

>AmOr169

MKMNFQNLNRLNTFVNAVSGNILPITDMKKRLSIVLKIYSILVWTIELSYLAACILGLFNVSRERALKDSTVNI
VISLEVFVLIVYLHNRENLLRELIGKLNCLLIVDDETLRDVTIGTVKPLEKPLRVYIIASVGSLMIWASLPLAKIFR
KSEFYYTDYQVPAVISNEPFPIGVFIGGVALQIFGSAYTLLRKVSLDLYTMHLILLITAQYKYLRIKFAAILEQET
PKDFFYGGIIWQNVPCEYDKMVKQEMKLLTRHFEIVVEMTVMLKKLLSPNIGILYINYVFRFCFLSFMLATSSG
MHFEKCLLVSYTIGALIQFYILCYCIQQLLEASTTVADDVVHEKWYLHDVKFQHIILMITLANKLKCKLSSFRN
IDLTLPSFMSILNQAYSVCLLFLKARQS

>AmOr170

MNFQNLNRLNAFANMVSGNFLPMTNINEKLSTILKIYFVVAWIIELIYVAASFLGLFNVSGEKALKDGTVNIA
ISFEVIVFNIYLHSRKKLLHKLIGKLNHLLITEDEIFRSVIIDTVKPLEMPLKIYVIASVASLMIWILSPLIKLFQKDE
FYYEDFIMPAVFSKQQPFSNDVFICGIFLQLLGGEDTIIRKISLDIYTIYLCLLITAQYKYLRIKFAIILKEEREITKD
HYKNIIWRNDNVRQEMKLVTRHFETVIETTTILKKLISPNIGFLYLSYVFRFCFLSFMFAMTTAKYFEKCLLASY
TIGALIQFYILCYCIQRLFEASSSIADDVVYEKWYYYDVRFQRVILMISLSNELKCKISNFQNIDLTLPTFMSILN
QAYSVCLLFLKARQD

>AmOr171

MSKELRLYKKYASFVKLFLLIGGICPITRELNVIYRYIPIWAIFSCFIELCAVGNSSLQNVENIPLLTASLILIGTILN
VITKTSCFFIHRKKLHQVNDIFNSILEEILNEIYIKSIVLFYLQIIYRLIYVQTVLMFVTTIIYSMKPMIIKLFHDANI
TNVQYPLPLFGTFPWKINSMLIWQLHYFFDVNILWFIFSVSVSVDAFFGFCMFRICVILRFLSFEFRSSIDDKNK
RNKEKSYQQIFRECVEKHVLLLKCRNIIQEVYGPIILLVTITNALSMCSIIFQLFQVNGINIYKIGTFMIYLMLKLI
QTFLYSWPGDVIFTESEFLRRNVYCSCWYDKNTSFAKYFLLVLAQRPIVLKACSLVQVTMDLLAKIMNTTISY
YFLLETMNDDK

>AmOr172PSE

MDFEGSQYYNINRILMISVGLWPYERTIYSKMLNILIFFFLSTAIFIQVMSFIMLKMDINLTLNSLSYTSCTCICIM
KYFNCLFHIKDIKNFLDEIKNDWNSLRNIEELRIIHEYSKTIKKITICFVIIIVPLQLVFFLNVFGNNILDILIPLNH
TRPRTVPIEIYYFIDQQKFFFFFGMHLNIITSFGGLVYIAIETISMGMIQHLCGLLKITSFRISHTFVANIPKISSTER
SIIIRKKVMSIVYLHVKIKK

>AmOr173PSE

MDFEGSQYYNINRILMISVGLWPYERTIYSKMLNILIFFFLXTAIFIQIMSFIMLKMDINLTLNSLSYISCTCICIMK
YFNCLFHIKDIKNFLDEIKNDWNSLRNIEELRIIHEYSKTIKKITICFVIIIVPLQLVFFLNVFGNNILDILIPLNHT
RPRTVPIEIYYFIDQQKFFFFFGMHLNIITSFGGLVYIAIETIYMGMIQHLCGLLK

>AmOr174PSE

MDFEGSQYYNINRILMICVGLWPFERTIYSKLLNIFIFLLMSIAIFVQTMSFMMLKVDMSLMLTSLSYILCTCIFS
MKYFSFLFHIKDVKEYEELQIIHEYSKTTKGITICFVXIIVPLQLVSFLSIFGNDILDILIPLNYTRSRTIPIVIFVDQQ
KFFYIFGTYLNLITSFGGLGIIATETTSMAVMQHLCGLLKILS

>DmOr83b

MTTSMQPSKYTGLVADLMPNIRAMKYSGLFMHNFTGGSAFMKKVYSSVHLVFLLMQFTFILVNMALNAEE
VNELSGNTITTLFFTHCITKFIYLAVNQKNFYRTLNIWNQVNTHPLFAESDARYHSIALAKMRKLFFLVMLTT
VASATAWTTITFFGDSVKMVVDHETNSSIPVEIPRLPIKSFYPWNASHGMFYMISFAFQIYYVLFSMIHSNLCD
VMFCSWLIFACEQLQHLKGIMKPLMELSASLDTYRPNSAALFRSLSANSKSELIHNEEKDPGTDMDMSGIYSS
KADWGAQFRAPSTLQSFGGNGGGGNGLVNGANPNGLTKKQEMMVRSAIKYWVERHKHVVRLVAAIGDT
YGAALLLHMLTSTIKLTLLAYQATKINGVNVYAFTVVGYLGYALAQVFHFCIFGNRLIEESSSVMEAAYSCH
WYDGSEEAKTFVQIVCQQCQKAMSISGAKFFTVSLDLFASVLGAVVTYFMVLVQLK
